# Comparison of bivalent and monovalent SARS-CoV-2 variant vaccines: the phase 2 randomized open-label COVAIL trial

**DOI:** 10.1038/s41591-023-02503-4

**Published:** 2023-08-28

**Authors:** Angela R. Branche, Nadine G. Rouphael, David J. Diemert, Ann R. Falsey, Cecilia Losada, Lindsey R. Baden, Sharon E. Frey, Jennifer A. Whitaker, Susan J. Little, Evan J. Anderson, Emmanuel B. Walter, Richard M. Novak, Richard Rupp, Lisa A. Jackson, Tara M. Babu, Angelica C. Kottkamp, Anne F. Luetkemeyer, Lilly C. Immergluck, Rachel M. Presti, Martín Bäcker, Patricia L. Winokur, Siham M. Mahgoub, Paul A. Goepfert, Dahlene N. Fusco, Elissa Malkin, Jeffrey M. Bethony, Edward E. Walsh, Daniel S. Graciaa, Hady Samaha, Amy C. Sherman, Stephen R. Walsh, Getahun Abate, Zacharoula Oikonomopoulou, Hana M. El Sahly, Thomas C. S. Martin, Satoshi Kamidani, Michael J. Smith, Benjamin G. Ladner, Laura Porterfield, Maya Dunstan, Anna Wald, Tamia Davis, Robert L. Atmar, Mark J. Mulligan, Kirsten E. Lyke, Christine M. Posavad, Megan A. Meagher, David S. Stephens, Kathleen M. Neuzil, Kuleni Abebe, Heather Hill, Jim Albert, Kalyani Telu, Jinjian Mu, Teri C. Lewis, Lisa A. Giebeig, Amanda Eaton, Antonia Netzl, Samuel H. Wilks, Sina Türeli, Mamodikoe Makhene, Sonja Crandon, David C. Montefiori, Mat Makowski, Derek J. Smith, Seema U. Nayak, Paul C. Roberts, John H. Beigel, the COVAIL Study Group, Angela R. Branche, Nadine G. Rouphael, David J. Diemert, Ann R. Falsey, Cecilia Losada, Lindsey R. Baden, Sharon E. Frey, Jennifer A. Whitaker, Susan J. Little, Evan J. Anderson, Emmanuel B. Walter, Richard M. Novak, Richard Rupp, Lisa A. Jackson, Tara M. Babu, Angelica C. Kottkamp, Anne F. Luetkemeyer, Lilly C. Immergluck, Rachel M. Presti, Martín Bäcker, Patricia L. Winokur, Siham M. Mahgoub, Paul A. Goepfert, Dahlene N. Fusco, Elissa Malkin, Jeffrey M. Bethony, Daniel S. Graciaa, Hady Samaha, Amy C. Sherman, Stephen R. Walsh, Getahun Abate, Zacharoula Oikonomopoulou, Hana M. El Sahly, Thomas C. S. Martin, Satoshi Kamidani, Michael J. Smith, Benjamin G. Ladner, Laura Porterfield, Maya Dunstan, Anna Wald, Tamia Davis, Robert L. Atmar, Kirsten E. Lyke, Christine M. Posavad, Megan A. Meagher, David S. Stephens, Kathleen M. Neuzil, Kuleni Abebe, Heather Hill, Jim Albert, Kalyani Telu, Jinjian Mu, Teri C. Lewis, Lisa A. Giebeig, Amanda Eaton, Antonia Netzl, Samuel H. Wilks, Sina Türeli, Mamodikoe Makhene, Sonja Crandon, David C. Montefiori, Mat Makowski, Derek J. Smith, Seema U. Nayak, Paul C. Roberts, John H. Beigel, Edward Walsh, Patrick Kingsley, Kari Steinmetz, Michael Peasley, Cassie Grimsley Ackerley, Kristen E. Unterberger, Aimee Desrosiers, Marc Siegel, Alexandra Tong, Rebecca Rooks, Daniel F. Hoft, Irene Graham, Wendy A. Keitel, C. Mary Healy, Nicole Carter, Steven Hendrickx, Christina A. Rostad, Etza Peters, Lauren Nolan, M. Anthony Moody, Kenneth E. Schmader, Andrea Wendrow, Jessica Herrick, Rebecca Lau, Barbara Carste, Taylor Krause, Kirsten Hauge, Celia Engelson, Vijaya Soma, Chloe Harris, Azquena Munoz Lopez, Erica Johnson, Austin Chan, Fatima Ali, Trisha Parker, Jane A. O’Halloran, Ryley M. Thompson, Kimberly Byrnes, Asif Noor, Jeffery Meier, Jack Stapleton, Celia Maxwell, Sarah Shami, Arnaud C. Drouin, Florice K. Numbi, Julie McElrath, Mike Gale, Holly Baughman, Lisa McQuarrie, Theresa M. Engel, Caleb J. Griffith, Wendi L. McDonald, Alissa E. Burkey, Lisa B. Hoopengardner, Jessica E. Linton, Nikki L. Gettinger, Marina Lee, Mohamed Elsafy, Rhonda Pikaart-Tautges, Janice Arega, Binh Hoang, Dan Curtin, Hyung Koo, Elisa Sindall, Marciela M. DeGrace, Diane J. Post, David S. Stephens, Kathleen M. Neuzil, Monica M. Farley, Jeanne Marrazzo, Sidnee Paschal Young, Jeffery Lennox, Robert L. Atmar, Linda McNeil, Elizabeth Brown

**Affiliations:** 1 Department of Medicine, Division of Infectious Diseases, University of Rochester, Rochester, NY USA; 2 Hope Clinic, Emory University, Decatur, GA USA; 3 George Washington Vaccine Research Unit, George Washington University, Washington D.C., WA USA; 4 Brigham and Women’s Hospital, Harvard Medical School, Boston, MA USA; 5 Center for Vaccine Development, Saint Louis University, St. Louis, MO USA; 6 Departments of Molecular Virology and Microbiology and Medicine, Baylor College of Medicine, Houston, TX USA; 7 Division of Infectious Diseases and Global Public Health, Department of Medicine, University of California San Diego, La Jolla, CA USA; 8 Center for Childhood Infections and Vaccines (CCIV) of Children’s Healthcare of Atlanta and Emory University Department of Pediatrics, Atlanta, GA USA; 9 Duke Human Vaccine Institute, Duke University School of Medicine, Durham, NC USA; 10 Project WISH, University of Illinois at Chicago, Chicago, IL USA; 11 University of Texas Medical Branch, Galveston, TX USA; 12 Kaiser Permanente Washington Health Research Institute, Seattle, WA USA; 13 Departments of Medicine, Epidemiology and Laboratory Medicine and Pathology, University of Washington, Vaccines and Infectious Diseases Division, Fred Hutchinson Cancer Center, Seattle, WA USA; 14 NYU VTEU Manhattan Research Clinic, NYU Grossman School of Medicine, New York, NY USA; 15 Zuckerberg San Francisco General, University of California San Francisco, San Francisco, CA USA; 16 Department of Microbiology, Biochemistry and Immunology, and Clinical Research Center, Morehouse School of Medicine, Atlanta, GA USA; 17 Washington University School of Medicine, St. Louis, MO USA; 18 NYU VTEU Long Island Research Clinic, NYU Long Island School of Medicine, Mineola, NY USA; 19 College of Medicine, University of Iowa, Iowa City, IA USA; 20 Howard University College of Medicine, Howard University Hospital, Washington D.C., WA USA; 21 Department of Medicine, University of Alabama at Birmingham, Birmingham, AL USA; 22 Tulane University School of Medicine, New Orleans, LA USA; 23 Center for Vaccine Development and Global Health, University of Maryland School of Medicine Baltimore, Baltimore, MD USA; 24 IDCRC Laboratory Operations Unit, Fred Hutchinson Cancer Center, Seattle, WA USA; 25 Department of Medicine and Woodruff Health Sciences Center, Emory University, Atlanta, GA USA; 26 FHI 360, Durham, NC USA; 27 The Emmes Company, LLC, Rockville, MD USA; 28 Clinical Monitoring Research Program Directorate, Frederick National Laboratory for Cancer Research, Frederick, MD USA; 29 Department of Surgery, Duke University Medical Center, Durham, NC USA; 30 Center for Pathogen Evolution, Department of Zoology, University of Cambridge, Cambridge, UK; 31 Division of Microbiology and Infectious Diseases, National Institute of Allergy and Infectious Diseases, National Institutes of Health, Bethesda, MD USA; 32 IDCRC Principal Investigators, Atlanta, GA USA; 33 IDCRC Leadership Operations Center, Atlanta, GA USA; 34 IDCRC Clinical Operations Unit, Atlanta, GA USA; 35 IDCRC Statistical and Data Science Unit, Fred Hutchinson Cancer Center, Seattle, WA USA

**Keywords:** Translational research, RNA vaccines, Randomized controlled trials

## Abstract

Vaccine protection against severe acute respiratory syndrome coronavirus 2 (SARS-CoV-2) infection wanes over time, requiring updated boosters. In a phase 2, open-label, randomized clinical trial with sequentially enrolled stages at 22 US sites, we assessed safety and immunogenicity of a second boost with monovalent or bivalent variant vaccines from mRNA and protein-based platforms targeting wild-type, Beta, Delta and Omicron BA.1 spike antigens. The primary outcome was pseudovirus neutralization titers at 50% inhibitory dilution (ID_50_ titers) with 95% confidence intervals against different SARS-CoV-2 strains. The secondary outcome assessed safety by solicited local and systemic adverse events (AEs), unsolicited AEs, serious AEs and AEs of special interest. Boosting with prototype/wild-type vaccines produced numerically lower ID_50_ titers than any variant-containing vaccine against all variants. Conversely, boosting with a variant vaccine excluding prototype was not associated with decreased neutralization against D614G. Omicron BA.1 or Beta monovalent vaccines were nearly equivalent to Omicron BA.1 + prototype or Beta + prototype bivalent vaccines for neutralization of Beta, Omicron BA.1 and Omicron BA.4/5, although they were lower for contemporaneous Omicron subvariants. Safety was similar across arms and stages and comparable to previous reports. Our study shows that updated vaccines targeting Beta or Omicron BA.1 provide broadly crossprotective neutralizing antibody responses against diverse SARS-CoV-2 variants without sacrificing immunity to the ancestral strain. ClinicalTrials.gov registration: NCT05289037.

## Abstract (web summary)

Analysis of antibody responses to COVID-19 vaccines encoding variant-specific spike, with or without ancestral spike, suggests no loss of neutralization of the ancestral virus with variant-only vaccines, which may simplify future vaccine updates.

## Main

Severe acute respiratory syndrome coronavirus 2 (SARS-CoV-2) has infected over 750 million people worldwide and resulted in nearly 7 million deaths, including more than 1 million deaths in the United States^1,2^. Coronavirus disease 2019 (COVID-19) prototype/wildtype vaccines authorized for emergency use or fully approved in the United States are safe and highly effective against severe disease and death^3–6^. However, vaccine protection against infection wanes over time^7–10^. In addition, new variants of concern (VOCs) have emerged, including B.1.351 (Beta), B.1.617.2 (Delta), B.1.1.529 (Omicron BA.1) and Omicron subvariants, all with mutations in the spike protein receptor binding domain (RBD) that result in diminished viral neutralization by antibodies^11–13^, leading to increased rates of infections but maintaining efficacy against severe COVID-19. Although additional booster doses of prototype/wildtype vaccines based on the ancestral strain improve vaccine effectiveness (VE) against infection by VOCs in the short term^14–20^, variant-specific boosters may optimize vaccine immunogenicity against current and future VOCs.

In this phase 2 clinical trial, we evaluated boosting with ancestral and variant SARS-CoV-2 spike protein(s) (Beta, Delta and Omicron BA.1), alone or in combination, using two mRNA vaccines and a recombinant protein vaccine, and across three sequentially enrolled stages, to assess the breadth, magnitude and durability of neutralizing antibody responses.

## Results

### Study population

**Fig. 1:**
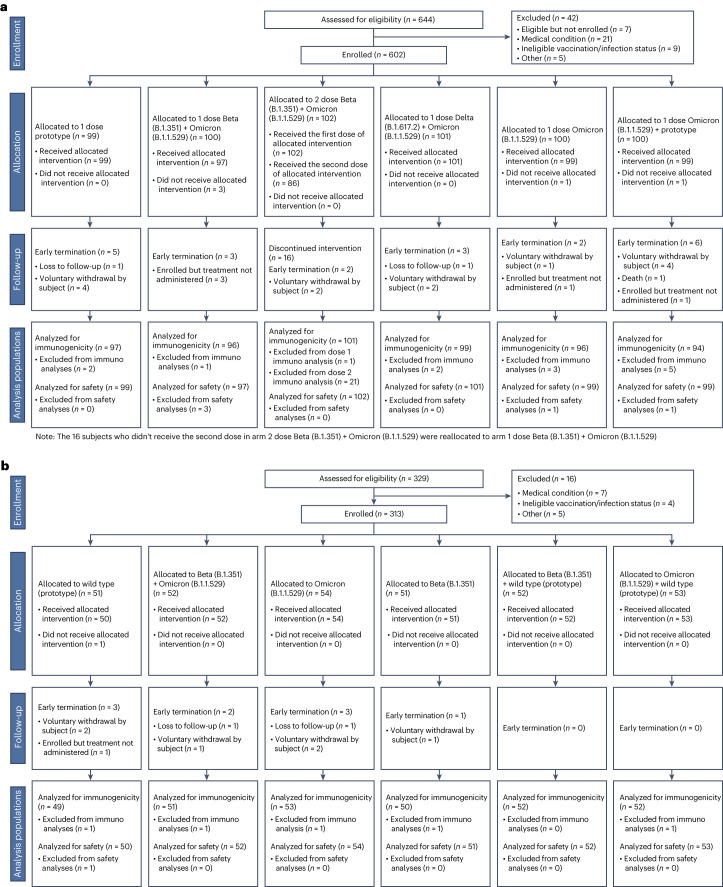

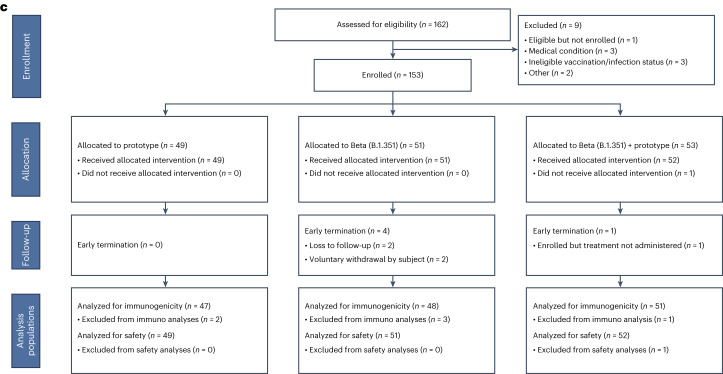
Consort diagram for the study. **a**–**c**, Shown are the consort diagrams for stage 1 (**a**), stage 2 (**b**) and stage 3 (**c**) of the study. A description of the number of participants screened for eligibility, enrolled, allocated to a vaccine arm and the number vaccinated is included for each stage. Additional details are provided on the follow-up of participants at the time of data cut-off and the analysis populations. Immuno, immunogenicity.

From 30 March to 6 May 2022, 602 participants were randomized and 597 received a mRNA-1273 vaccine (Moderna) in stage 1 (Table 1 and Fig. 1)^21^. From 12 to 27 May 2022, 313 participants were randomized and 312 received a BNT162b2 vaccine (Pfizer–BioNTech) in stage 2. From 8 to 17 June 2022, 153 participants were randomized and 152 received a pre-S DTM AS03 protein vaccine (Sanofi) in stage 3. This study was conducted whereby each stage was designed independently and sequentially based on the availability of new products and platforms. Vaccine selection in previous stages also informed the design of subsequent stages.

**Table 1:** Summary of demographic and baseline characteristics by stage and vaccination arm

	Stage 1: mRNA-1273 (50 µg) Enrollment 30 March–6 May 2022							Stage 2: BNT162b2 (30 µg) Enrollment 12–27 May 2022							Stage 3: pre-S DTM ASO3 (5 µg) Enrollment 8–17 June 2022			
	Arm 1: 1 dose prototype (*n* = 99)	Arm 2: 1 dose Beta + Omicron BA.1 (*n* = 100)	Arm 3: 2 dose Beta + Omicron BA.1 (*n* = 102)	Arm 4: 1 dose Delta + Omicron BA.1 (*n* = 101)	Arm 5: 1 dose Omicron BA.1 (*n* = 100)	Arm 6: 1 dose Omicron BA.1 + prototype (*n* = 100)	Total (*n* = 602)	Arm 7: 1 dose wild type (*n* = 51)	Arm 8: 1 dose Beta + Omicron BA.1 (*n* = 52)	Arm 9: 1 dose Omicron BA.1 (*n* = 54)	Arm 10: 1 dose Beta (*n* = 51)	Arm 11: 1 dose Beta + wild type (*n* = 52)	Arm 12: 1 dose Omicron BA.1 + wild type (*n* = 53)	Total (*n* = 313)	Arm 13: 1 dose prototype (*n* = 49)	Arm 14: 1 dose Beta (*n* = 51)	Arm 15: 1 dose Beta + wild type (*n* = 53)	Total (*n* = 153)
**Age**																		
Median age, yr (range)	55 (22–79)	54.5 (19–81)	53.5 (24–80)	53 (19–76)	51 (21–85)	52.5 (18–78)	53 (18–85)	49 (22–76)	47 (20–81)	45 (21–83)	52 (22–77)	41.5 (25–81)	48 (20–82)	47 (20–83)	38 (18–76)	47 (26–79)	43 (19–79)	45 (18–79)
18–64 years, *n* (%)	64 (65)	65 (65)	66 (65)	65 (64)	65 (65)	66 (66)	391 (65)	36 (71)	36 (69)	38 (70)	36 (71)	35 (67)	38 (72)	219 (70)	39 (80)	40 (78)	43 (81)	122 (80)
≥65 years, *n* (%)	35 (35)	35 (35)	36 (35)	36 (36)	35 (35)	34 (34)	211 (35)	15 (29)	16 (31)	16 (30)	15 (29)	17 (33)	15 (28)	94 (30)	10 (20)	11 (22)	10 (19)	31 (20)
**Sex,** ***n*** **(%)**																		
Men	49 (49)	43 (43)	45 (44)	49 (49)	46 (46)	51 (51)	283 (47)	27 (53)	25 (48)	24 (44)	23 (45)	24 (46)	22 (42)	145 (46)	16 (33)	21 (41)	26 (49)	63 (41)
Women	50 (51)	57 (57)	57 (56)	52 (51)	54 (54)	49 (49)	319 (53)	24 (47)	27 (52)	30 (56)	28 (55)	28 (54)	31 (58)	168 (54)	33 (67)	30 (59)	27 (51)	90 (59)
**Ethnicity,** ***n*** **(%)**																		
Not Hispanic or Latino	94 (95)	90 (90)	100 (98)	94 (93)	93 (93)	95 (95)	566 (94)	46 (90)	46 (88)	45 (83)	49 (96)	49 (94)	52 (98)	287 (92)	41 (84)	46 (90)	49 (92)	136 (89)
Hispanic or Latino	5 (5)	10 (10)	2 (2)	7 (7)	7 (7)	5 (5)	36 (6)	4 (8)	6 (12)	9 (17)	2 (4)	3 (6)	1 (2)	25 (8)	8 (16)	5 (10)	4 (8)	17 (11)
Not reported	0 (0)	0 (0)	0 (0)	0 (0)	0 (0)	0 (0)	0 (0)	1 (2)	0 (0)	0 (0)	0 (0)	0 (0)	0 (0)	1 (0)	0 (0)	0 (0)	0 (0)	0 (0)
**Race,** ***n*** **(%)**																		
American Indian or Alaska Native	0 (0)	2 (2)	0 (0)	0 (0)	0 (0)	0 (0)	2 (0)	1 (2)	0 (0)	0 (0)	0 (0)	0 (0)	0 (0)	1 (0)	0 (0)	0 (0)	0 (0)	0 (0)
Asian	5 (5)	6 (6)	8 (8)	6 (6)	7 (7)	9 (9)	41 (7)	5 (10)	9 (17)	9 (17)	5 (10)	6 (12)	6 (11)	40 (13)	9 (18)	4 (8)	4 (8)	17 (11)
Native Hawaiian or other Pacific Islander	0 (0)	0 (0)	0 (0)	0 (0)	0 (0)	0 (0)	0 (0)	0 (0)	0 (0)	0 (0)	0 (0)	0 (0)	0 (0)	0 (0)	0 (0)	0 (0)	0 (0)	0 (0)
Black	12 (12)	8 (8)	6 (6)	6 (6)	4 (4)	10 (10)	46 (8)	6 (12)	2 (4)	3 (6)	5 (10)	5 (10)	2 (4)	23 (7)	3 (6)	9 (18)	4 (8)	16 (10)
White	79 (80)	82 (82)	87 (85)	81 (80)	86 (86)	79 (79)	494 (82)	38 (75)	39 (75)	39 (72)	39 (76)	38 (73)	44 (83)	237 (76)	35 (71)	38 (75)	38 (72)	111 (73)
Multiracial	2 (2)	1 (1)	1 (1)	8 (8)	2 (2)	1 (1)	15 (2)	1 (2)	2 (4)	3 (6)	2 (4)	3 (6)	1 (2)	12 (4)	0 (0)	0 (0)	6 (11)	6 (4)
Unknown race	1 (1)	1 (1)	0 (0)	0 (0)	1 (1)	1 (1)	4 (1)	0 (0)	0 (0)	0 (0)	0 (0)	0 (0)	0 (0)	0 (0)	2 (4)	0 (0)	1 (2)	3 (2)
**History of prior infection,** ***n*** **(%)**																		
Self-report	15 (15)	14 (15)	15 (15)	15 (15)	15 (15)	15 (15)	89 (15)	11 (22)	12 (23)	13 (24)	12 (24)	12 (23)	13 (25)	73 (23)	15 (31)	15 (29)	15 (28)	45 (29)
Positive nucleocapsid antibody	20 (20)	16 (16)	19 (19)	15 (15)	23 (23)	18 (18)	111 (18)	17 (33)	18 (35)	18 (33)	16 (31)	14 (27)	16 (30)	99 (32)	22 (45)	18 (35)	19 (36)	59 (39)
Self-report or positive nucleocapsid antibody	21 (21)	18 (18)	21 (21)	17 (17)	23 (23)	20 (20)	120 (20)	17 (33)	18 (35)	18 (33)	18 (35)	15 (29)	18 (34)	104 (33)	22 (45)	20 (39)	20 (38)	62 (41)
**Prior SARS-CoV-2 vaccination regimen,** ***n*** **(%)**																		
mRNA primary, mRNA boost	95 (96)	93 (95)	97 (95)	95 (94)	95 (96)	96 (97)	571 (95)	51 (100)	52 (100)	54 (100)	50 (98)	52 (100)	51 (96)	310 (99)	46 (94)	50 (98)	51 (96)	147 (96)
Ad26 primary, mRNA boost	4 (4)	5 (5)	4 (4)	5 (5)	4 (4)	1 (1)	23 (4)	0 (0)	0 (0)	0 (0)	1 (2)	0 (0)	2 (4)	3 (1)	2 (4)	0 (0)	2 (4)	4 (3)
Ad26 primary, Ad26 boost	0 (0)	0 (0)	1 (1)	1 (1)	0 (0)	2 (2)	4 (1)	0 (0)	0 (0)	0 (0)	0 (0)	0 (0)	0 (0)	0 (0)	1 (2)	1 (2)	0 (0)	2 (1)
**Days since most recent known SARS-CoV-2 antigenic exposure, median days (range)**																		
Most recent COVID-19 vaccine	170.0 (113–333)	164.0 (112–244)	176.5 (120–239)	167.0 (114–258)	174.0 (114–261)	170.0 (114–238)	170.0 (112–333)	198.0 (125–262)	200.0 (110–284)	203.0 (106–333)	209.0 (131–269)	206.0 (139–276)	202.0 (115–267)	202.0 (106–333)	209.0 (123–295)	202.0 (79–253)	211.0 (108–359)	209.0 (79–359)
Most recent self-reported COVID-19 infection	475.0 (118–719)	250.5 (117–675)	227.0 (116–770)	218.0 (119–744)	493.0 (115–773)	243.0 (110–760)	395.0 (110–773)	170.0 (122–641)	246.5 (116–873)	163.0 (130–526)	156.0 (113–531)	141.5 (121–782)	141.0 (117–732)	148.0 (113–873)	156.0 (123–723)	211.0 (130–461)	152.0 (117–817)	156.5 (117–817)
Most recent COVID-19 vaccine or self-reported COVID-19 infection	164.0 (113–333)	162.0 (112–244)	173.5 (116–239)	165.0 (114–258)	172.0 (114–261)	169.0 (110–238)	168.0 (110–333)	192.0 (122–262)	193.0 (110–284)	194.5 (106–333)	201.0 (113–269)	199.5 (121–276)	202.0 (115–267)	198.0 (106–333)	199.0 (123–295)	188.0 (79–253)	201.0 (108–359)	197.0 (79–359)

Baseline demographics were similar across study arms within each stage (Table 1). The median age (range) was 53 (18–85) years for stage 1, 47 (20–83) years for stage 2 and 45 (18–79) years for stage 3; 35%, 30% and 20% were ≥65 years for each stage, respectively. The majority of participants were women (53–59%); 6–11% were Hispanic and 73–82% were white. All participants had received a primary series and initial boost vaccination at enrollment, the majority with an mRNA vaccine (94–100% per arm). In stages 1, 2 and 3, 20%, 33% and 41%, respectively, were defined as previously infected based on anti-nucleocapsid (anti-N) antibody seropositivity at baseline and/or by self-reported past positive SARS-CoV-2 PCR or antigen testing. Median duration (range) between study vaccination and the last previous vaccination or infection was 168 (110–333) days, 198 (106–333) days and 197 (79–359) days for stages 1, 2 and 3, respectively. Median follow-up duration at data cut-off was 228 days, 193 days and 176 days for stages 1, 2 and 3, respectively.

### Safety

**Extended Data Fig. 1:**
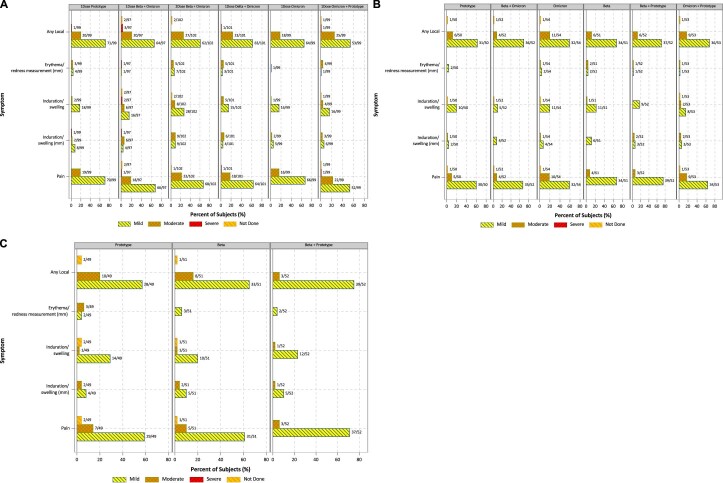
Frequency and severity of local solicited adverse events by stage and vaccine arm. Maximum severity of local solicited events for stage 1 (A), stage 2 (B) and stage 3 (C).

**Extended Data Fig. 2:**
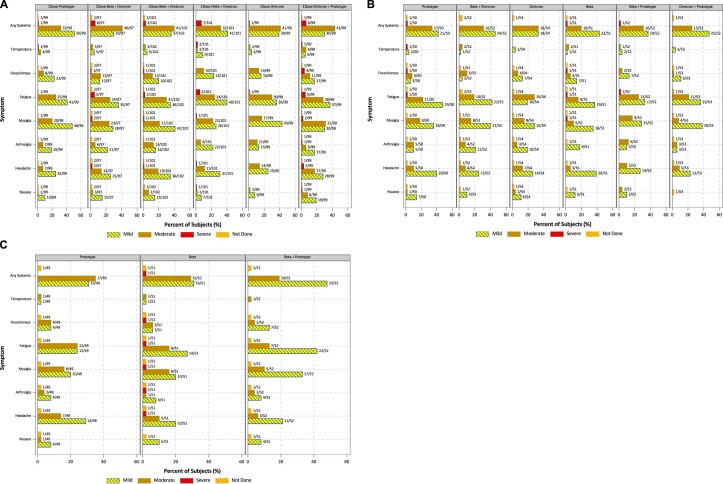
Frequency and severity of systemic solicited adverse events by stage and vaccine arm. Maximum severity of systemic solicited events for stage 1 (A), stage 2 (B) and stage 3 (C).

**Extended Data Fig. 3:**
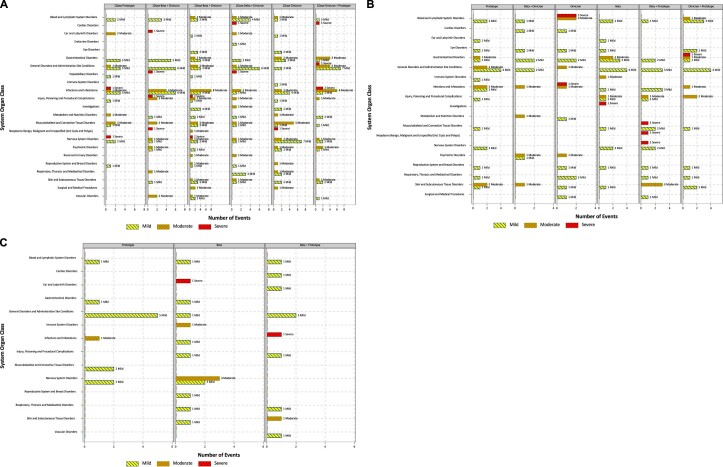
Frequency and severity of all adverse events by stage and vaccine arm. Number of severity of all adverse events by MedDRA® System Organ Class and vaccination group for stage 1 (A), stage 2 (B) and stage 3 (C).

The frequency and severity of solicited local and systemic adverse effects (AEs) after vaccination were similar to other booster trials^22^ and did not differ between arms in each stage (Extended Data Figs. 1 and 2). Multiple AEs could occur in a single participant. The most frequently reported solicited local AE was injection-site pain (83% of participants for stage 1, 77% for stage 2 and 74% for stage 3). The most common solicited systemic AEs were fatigue (50–67%) and myalgia (39–57%). Most solicited AEs were mild to moderate, with only 0–1% severe local AEs and 0.7–4% severe systemic AEs. A summary of all AEs is presented in Extended Data Figs. 1–3. As of the data cut-off, 13 participants in stage 1, 4 participants in stage 2 and 1 participant in stage 3 had a serious AE; all were deemed unrelated to study product. There was one related AE of special interest in stage 1 of a young man who reported chest pain 1 day after vaccination that was initially evaluated as possible myocarditis, which was ultimately excluded due to a normal troponin I level and normal cardiac magnetic resonance imaging. There was one death unrelated to study product due to cardiac arrest from advanced coronary artery disease.

### Neutralizing antibody responses for stage 1

Stage 1 participants were boosted with either the mRNA-1273 ancestral (prototype) vaccine or one of four different variant-targeting vaccine products including monovalent BA.1, and bivalent vaccines comprising BA.1 and either B.1.351 (Beta), B.1.617.2 (Delta) or ancestral (prototype) spike (Table 1). BA.1 was the Omicron variant vaccine available at the start of this trial. Neutralizing antibodies (pseudovirus-neutralizing antibodies (PsVN Abs)) were assessed against pseudoviruses expressing the spike proteins of ancestral (D614G) SARS-CoV-2 and variants B.1.617.2, B.1.351, BA.1 and BA.4/5 at baseline and on days 15, 29 and 91, and geometric mean titers (GMTs) were estimated with 95% confidence intervals (CIs) calculated at each time point. Although no prespecified hypothesis tests were planned, comparison of estimates using CIs allowed for numerical comparisons.

**Fig. 2:**
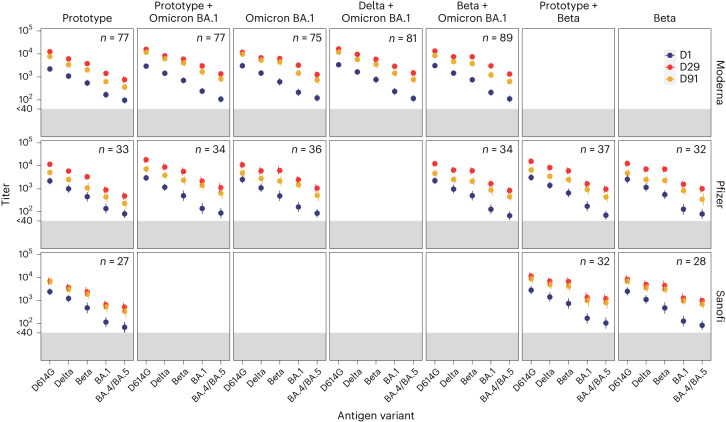
Pseudovirus neutralization ID_50_ titers by time point and variant in uninfected participants by vaccine arm and platform. Time points were Day 1 (D1), day 29 (D29) and day 91 (D91). Variants were D614G, Delta, Beta, Omicron BA.1 (B.1.1.529) and Omicron BA.4/BA.5. Circles denote GMT, with 95% CI. GMTs at prevaccination baseline, obtained on day 1, are shown in blue and postvaccination day 29 GMTs and day 91 GMTs are shown in red and yellow, respectively.

**Extended Data Fig. 4:**
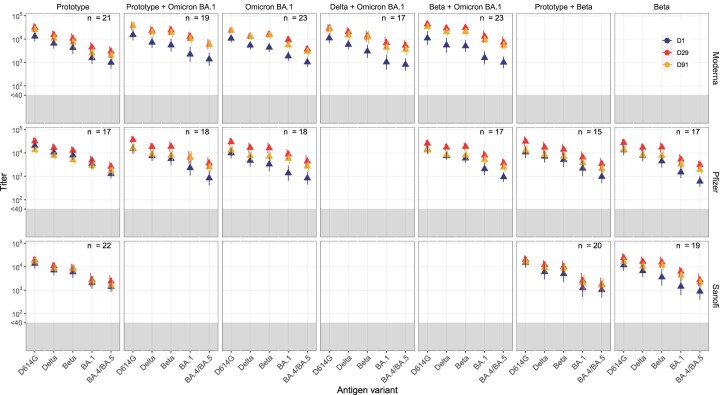
Pseudovirus neutralization ID_50_ titers by timepoint (D1, D29 and Day 91) and variant (D614G, Delta, Beta, Omicron BA.1 and Omicron BA.4/5) in infected participants by vaccine arm and platform.

**Extended Data Fig. 5:**
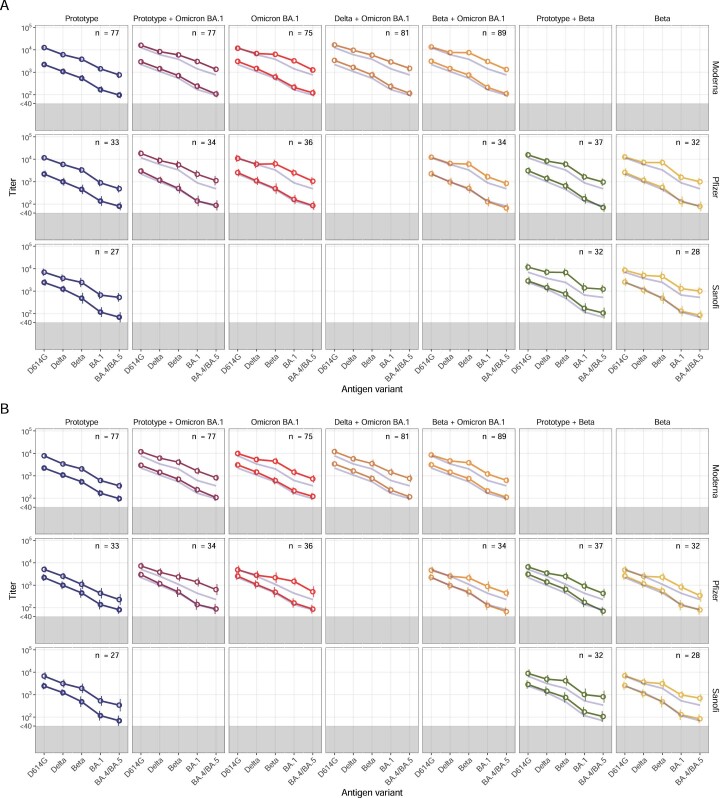
Comparison of pseudovirus neutralization ID_50_ titers at D1 and D29 (A) and D1 and D90 (B) as well as by variant (D614G, Delta, Beta, Omicron BA.1 and Omicron BA.4/5) in uninfected participants by vaccine arm and platform.

**Extended Data Fig. 6:**
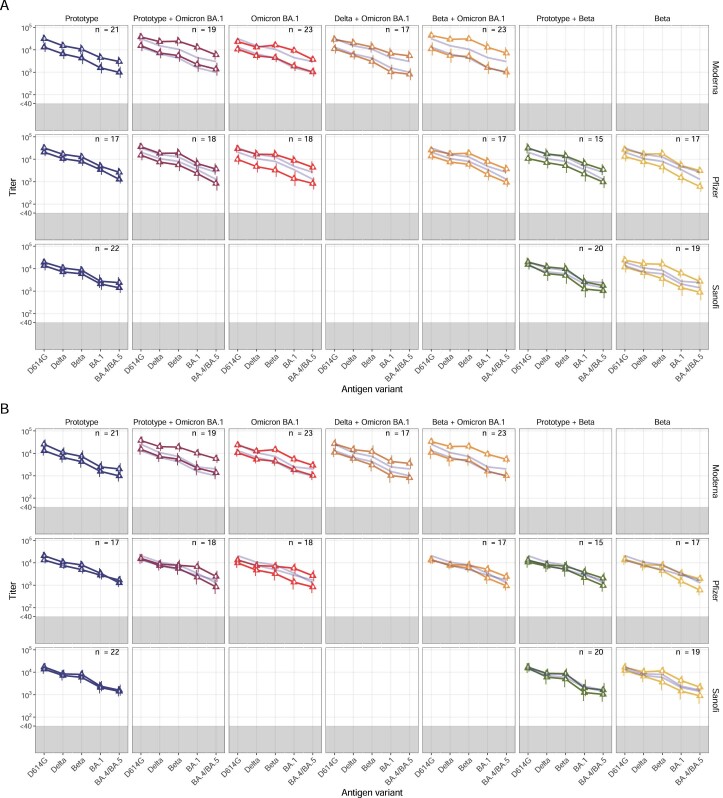
Comparison of pseudovirus neutralization ID_50_ titers at D1e and D29 (A) and D1 and D91 (B) as well as by variant (D614G, Delta, Beta, Omicron BA.1 and Omicron BA.4/5) in previously infected participants by vaccine arm and platform.

For stage 1, PsVN Ab responses peaked at day 15 after vaccination, remained relatively stable at day 29, were similar between older (≥65 years) and younger adults, and were 2–3 times higher in participants who were previously infected compared with those who were uninfected (Supplementary Tables 6 and 7). PsVN Ab GMTs against all variants declined from day 29 to day 91 by a factor of 1.74 (95% CI, 1.69, 1.80) in participants who were previously uninfected and by a factor of 1.34 (95% CI, 1.25, 1.44) in those who were previously infected (Fig. 2 and Extended Data Figs. 4–6).

For participants who were uninfected, all Omicron BA.1-containing vaccines (day 29 GMT_D614G_ of between 11,963 and 16,001) boosted PsVN Abs to D614G similarly to the prototype vaccine (day 29 GMT_D614G_ = 12,600) (Fig. 2). The prototype vaccine was less effective in boosting against all variants, based on numerically higher point estimates (GMT_B.1.617.2_ = 6,181, GMT_B.1.351_ = 3,535, GMT_BA.1_ = 1,343 and GMT_BA.4/5_ = 722 at day 29) when compared with variant vaccines (GMT_B.1.617.2_ between 6,902 and 9,342, GMT_B.1.351_ between 5,744 and 7,016, GMT_BA.1_ between 2,684 and 3,005 and GMT_BA.4/5_ between 1,190 and 1,384 at day 29). In particular, monovalent or bivalent Omicron BA.1 vaccines did not differ numerically in the point estimates of their ability to neutralize all variants tested (Fig. 2, Extended Data Figure 5 and Supplementary Table 6). The geometric mean fold rises (GMFR) at day 29 in all Omicron BA.1-containing vaccines against Omicron variants (GMFR_BA.1_ = 11.6 to 14.6 and GMFR_BA.4/5_ = 10.6 to 12.7) and B.1.351 (GMFR_B.1.351_ = 7.7 to 10.1) were higher when compared with B.1.617.2 (GMFR_B.1.617.2_ = 5.0 to 6.7) or D614G (GMFR_D614G_ = 4.3 to 5.7), suggesting either differences in antibody maturation for antigenically distant variants or a ceiling with D614G.

The antibody responses with Omicron BA.1-containing vaccines demonstrated a trend of greater durability, with a smaller estimate of geometric mean fold decline (GMFD) from day 29 to day 91 for B.1.351 (GMFD_B.1.351_ = 1.4 to 1.7) and Omicron subvariants (GMFD_BA.1_ = 2.0 to 2.2 and GMFD_BA.4/5_ = 1.8 to 2.0) when compared with the prototype vaccine (GMFD_B.1.351_ = 1.8, GMFD_BA.1_ = 2.3 and GMFD_BA.4/5_ = 2.1). Within each study arm, the ratio in geometric mean neutralization titer against variant pseudoviruses compared with the ancestral D614G pseudovirus (geometric mean ratio against D614G (GMR_D614G_)) was used as a measure of boosting effect, where lower values correspond to stronger responses of variant vaccines to variants other than D614G. GMR_D614G_ values also reflect the extent of neutralization escape, where higher values correspond to greater escape. In stage 1, less reduction in neutralization titers against Omicron variants was observed for Omicron BA.1-containing vaccines (GMR_D614G_ = 7.13 to 8.72 for BA.1 and 13.40 to 16.13 for BA.4/5) than with the prototype vaccine (GMR_D614G_ = 12.0 for BA.1 and 20.6 for BA.4/5) at day 91 (Extended Data Figure 5 and Supplementary Table 6).

### Neutralizing antibody against additional Omicron subvariants

**Fig. 3:**
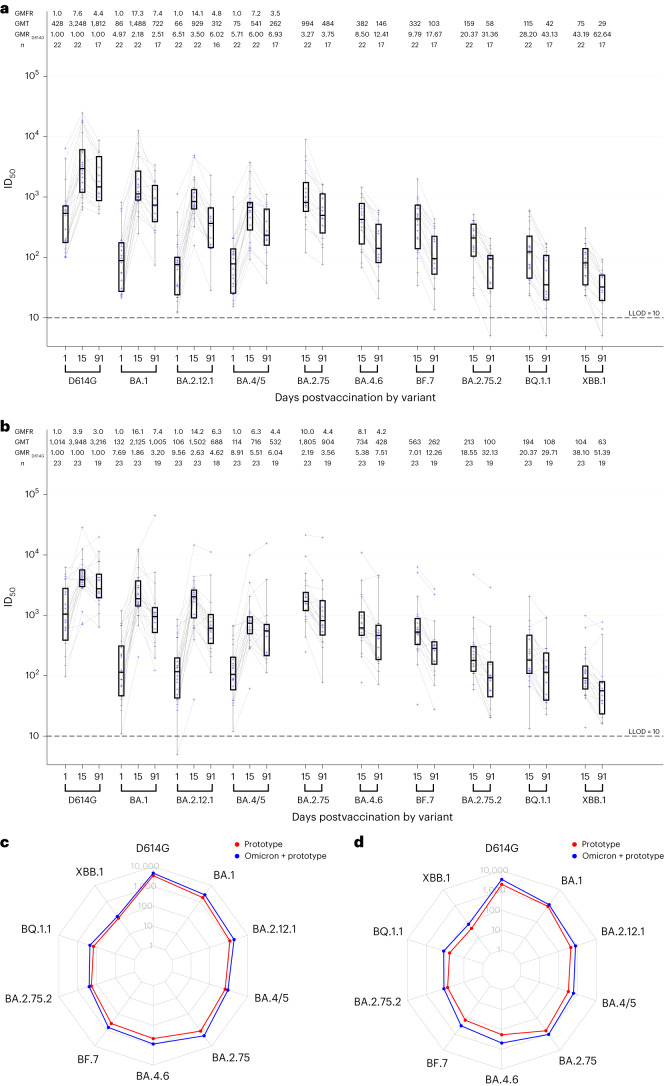
Pseudovirus neutralization ID_50_ titers by time point and variant in a subset (*n* = 22–23) of participants who were uninfected. Time points were days 1, 15 and 91. Variants were D614G and Omicron BA.1, BA.2.12.1, BA.4/BA.5, BA.2.75, BA.4.6, BF.7, BA.2.75.2, BQ.1.1 and XBB.1. **a**, Stage 1 mRNA-1273 prototype monovalent vaccine. **b**, Stage 1 mRNA-1273 Omicron BA.1 + prototype bivalent vaccine. In **a** and **b**, boxes and horizontal bars denote interquartile range and median ID_50_, respectively; whiskers denote 95% CI; and *n* represents the number of samples tested. **c**,**d**, Radar plots of the pseudovirus neutralization GMTs at day 15 (**c**) and day 91 (**d**) for the two vaccine arms in stage 1 mRNA-1273 prototype monovalent vaccine (red) and mRNA-1273 Omicron BA.1 + prototype bivalent vaccine (blue). Circles are GMT estimates for each variant. In the radar plots, each variant is represented by its own vertical line or spoke, and the spokes are evenly distributed around the circle. Each horizontal line along a vertical spoke represents the GMT at a ten-fold dilution, with the value closest to the center being 1 and farthest from the center being 10,000 or 10^4^. A line is drawn connecting the GMT data values for vaccine arm at the individual variants represented by its vertical spoke.

Serum samples from a subset of uninfected participants in stage 1 who were boosted with either the mRNA-1273 monovalent prototype vaccine (*n* = 22) or the mRNA-1273 bivalent Omicron BA.1 + prototype vaccine (*n* = 23) were tested at day 15 and day 91 for PsVN Abs to D614G and Omicron subvariants BA.1, BA.2.75, BA.2.12.1, BA.4/5, BA.4.6, BF.7, BA.2.75.2, BQ.1.1 and XBB.1 (Fig. 3 and Supplementary Table 12). The assays were performed in a separate laboratory using a pseudovirus platform that resembles but is not identical to the one used for the other datasets in this study.

PsVN Ab GMT estimates were highest against the ancestral D614G variant in both groups. Higher GMT estimates against all Omicron subvariants were observed at day 15 with the Omicron BA.1 + prototype bivalent vaccine when compared with the prototype. More pronounced reduction in neutralization titers was seen with the recent variants (BQ.1.1 and XBB.1; Fig. 3). The PsVN Ab response demonstrated a trend of modest improvement in durability with the bivalent compared with the prototype vaccine at day 91 relative to day 15 with a GMFD of 2.8 (95% CI, 2.2–3.5) and 2.7 (95%CI, 2.1–3.3) for the prototype vaccine compared with a decline of 2.1 (95%CI, 1.5–2.9) and 1.9 (95%CI, 1.4–2.6) for the Omicron BA.1 + prototype vaccine against BQ.1.1 and XBB.1, respectively (Fig. 3 and Supplementary Table 12).

These results highlight the remarkable speed at which the Omicron lineage evolved to evade vaccine-elicited neutralizing antibodies, where recent subvariants (for example, BQ.1.1 and XBB.1) are substantially more resistant to neutralization than earlier subvariants (for example, BA.1 and BA.2.75), regardless of whether the BA.1 spike was present in the vaccine boost.

### Neutralizing antibody responses for stage 2

Stage 2 participants were boosted with either the Pfizer BNT162b2 wild-type vaccine or one of five different variant-targeting versions of Pfizer BNT162b2 COVID-19 vaccine, including monovalent BA.1, monovalent B.1.351, a bivalent BA.1 + wild-type vaccine, and two additional bivalent vaccines comprising B.1.351 and either BA.1 or wild-type spike (Table 1). Neutralizing antibodies were assessed with the same assay used for the main dataset in stage 1.

Consistent with stage 1 results involving a similar mRNA vaccine, PsVN Ab GMT estimates peaked on day 15, remained relatively stable on day 29, were similar between older (≥65 years) and younger adults, and were 2–4 times higher in participants who were previously infected compared with those who were uninfected (Fig. 2, Supplementary Tables 8 and 9 and Extended Data Figs. 4–6).

For participants who were uninfected (Supplementary Table 8 and Extended Data Figure 5), all variant-containing vaccines (Beta or Omicron BA.1) boosted D614G PsVN Abs (day 29 GMT_D614G_ between 10,951 and 18,093) similarly to the wild-type vaccine (day 29 GMT_D614G_ = 11,600). As in stage 1, the wild-type vaccine was less effective in boosting against all variants (GMT_B.1.617.2_ = 5,890, GMT_B.1.351_ = 3,313, GMT_BA.1_ = 888 and GMT_BA.4/5_ = 485 at day 29) when point estimates were compared with all other variant vaccines (GMT_B.1.617.2_ between 6,002 and 8,721, GMT_B.1.351_ between 5,664 and 6,253, GMT_BA.1_ between 1,411 and 2,480, and GMT_BA.4/5_ between 839 and 1,054 at day 29).

B.1.351, BA.1 and BA.4/5 share a common set of mutations in the RBD (K417N, E484K/A and N501Y), which might account for the modestly improved neutralizing antibody responses against Omicron seen with the Beta and Beta + wild-type vaccines compared with the wild-type monovalent vaccine. However, although monovalent Omicron BA.1 and monovalent Beta vaccines similarly boosted titers to the B.1.351 variant (GMT_B.1.351_ of 6,253 and 6,247, respectively), they numerically differed in their ability to neutralize Omicron BA.1 (GMT_BA.1_ of 2,480 and 1,411, respectively).

The GMFR estimates at day 29 for all variant vaccines against Omicron (GMFR_BA.1_ = 9.5 to 17.3 and GMFR_BA.4/5_ = 11.4 to 14.2) and B.1.351 variants (GMFR_B.1.351_ = 9.1 to 13.9) were higher when compared with B.1.617.2 (GMFR_B.1.617.2_ = 5.5 to 8.3) or D614G variants (GMFR_D614G_ = 4.3 to 7.0). Of note, for the wild-type vaccine, GMFRs for the variants tested were not numerically different and ranged between 5.3 and 7.3 with overlapping CIs.

A trend of more durable antibody responses were observed with most variant-targeting vaccines, with a smaller GMFD estimate from day 29 to day 91, particularly for Omicron subvariants (GMFD_BA.1_ = 1.6 to 2.0 and GMFD_BA.4/5_ = 1.6 to 2.4) when compared with wild-type vaccine (GMFD_BA.1_ = 2.1 and GMFD_BA.4/5_ = 2.1). In addition, compared with responses against D614G, less reduction in neutralization titers to Omicron variants was observed for variant-containing vaccines (GMR_D614G_ = 3.3 to 7.1 for BA.1 and 9.5 to 15.1 for BA.4/5) than with the wild-type vaccine (GMR_D614G_ = 11.6 for BA.1 and 22.1 for BA.4/5) at day 91 (Fig. 2, Extended Data Figure 5 and Supplementary Table 8).

### Neutralizing antibody responses for stage 3

Stage 3 participants were boosted with one of three pre-S DTM AS03 protein vaccine products, including the prototype vaccine, a monovalent Beta vaccine and a bivalent Beta + prototype vaccine (Table 1). Neutralizing antibodies were assessed on days 1, 29 and 91 in the same assay used for the main datasets in stages 1 and 2. Day 15 samples were not tested for stage 3.

PsVN Ab GMT estimates at day 29 after vaccination with Sanofi variant vaccines were similar between older and younger adults and approximately 2–5 times higher in participants who were previously infected compared with those who were uninfected (Fig. 2, Supplementary Tables 10 and 11 and Extended Data Figs. 4–6). For participants who were uninfected, all Beta-containing vaccines boosted D614G antibody titers (day 29 GMT_D614G_ between 9,384 and 11,726) better than the prototype vaccine (day 29 GMT_D614G_ = 6,942) (Extended Data Figure 5 and Supplementary Table 10). The prototype vaccine was less effective in boosting against most variants (day 29 GMT_B.1.617.2_ = 3,739, GMT_B.1.351_ = 2,437 and GMT_BA.1_ = 667) when compared with the two variant vaccines (day 29 GMT_B.1.617.2_ between 5,670 and 6,996; GMT_B.1.351_ between 5,173 and 6,785 and GMT_BA.1_ between 1,169 and 1,391) based on point estimates. The GMFR estimates from baseline to day 29 in both variant vaccines against B1.617.2 (GMFR_B.1.617.2_ = 4.7 to 8.9), B.1.351 (GMFR_B.1.351_ = 8.6 to 16.3) and Omicron (GMFR_BA.1_ = 7.7 to 12.0 and GMFR_BA.4/5_ = 9.2 to 10.3) variants were numerically higher when compared with D614G variants (GMFR_D614G_ = 4.0 to 7.0).

Similar or a trend of modestly more durable antibody responses were seen in PsVN Ab titers from day 29 to day 91 with Beta-containing vaccines against Omicron subvariants (GMFD_BA.1_ = 1.5 to 2.1 and GMFD_BA.4/5_ = 1.5 to 1.7) when compared with prototype vaccine (GMFD_BA.1_ = 1.5 and GMFD_BA.4/5_ = 2.0). In addition, compared with responses against D614G, less reduction in neutralization titers for Omicron variants was observed for the Beta + prototype vaccine (GMR_D614G_ = 9.1 for BA.1 and 11.6 for BA.4/5) than with the prototype vaccine (GMR_D614G_ = 13.1 for BA.1 and 21.5 for BA.4/5) at day 91, based on point estimates, although CIs were overlapped (Fig. 2, Supplementary Table 10 and Extended Data Figure 5).

### Analysis of covariance modeling of variant vaccines to prototype/wild type

In analysis of covariance (ANCOVA) models (adjusted for baseline titers, age and baseline infection status) for each stage, the day 91 GMR comparing neutralization titers with variant-containing vaccines to first generation prototype/wild-type vaccines against the ancestral D614G variant ranged from 1.01 to 1.40 for each variant vaccine within the 3 stages.

In stage 1, all Omicron BA.1-containing Moderna vaccines led to a day 91 GMR_BA.1_ ≥ 1.88, GMR_BA.4.5_ ≥ 1.70 and GMR_B1.351_ ≥ 1.50 compared with the prototype vaccine, with unadjusted lower-bound CIs >1 (Extended Data Table 2). In stage 2, all Omicron BA.1- or Beta-containing Pfizer vaccines led to a day 91 GMR_BA.1_ ≥ 1.99, GMR_BA.4.5_ ≥ 1.8 and GMR_B.1.351_ ≥ 1.78 compared with the wild-type vaccine (Extended Data Table 3). The day 91 GMRs in stages 1 and 2 were similar or higher to those observed for day 29. In stage 3, all Beta-containing Sanofi vaccines led to a day 91 GMR of greater than 1 relative to the prototype vaccine, although the unadjusted lower-bound CI failed to exclude 1 (Extended Data Table 4).

### Antigenic cartography and antibody landscapes

**Fig. 4:**
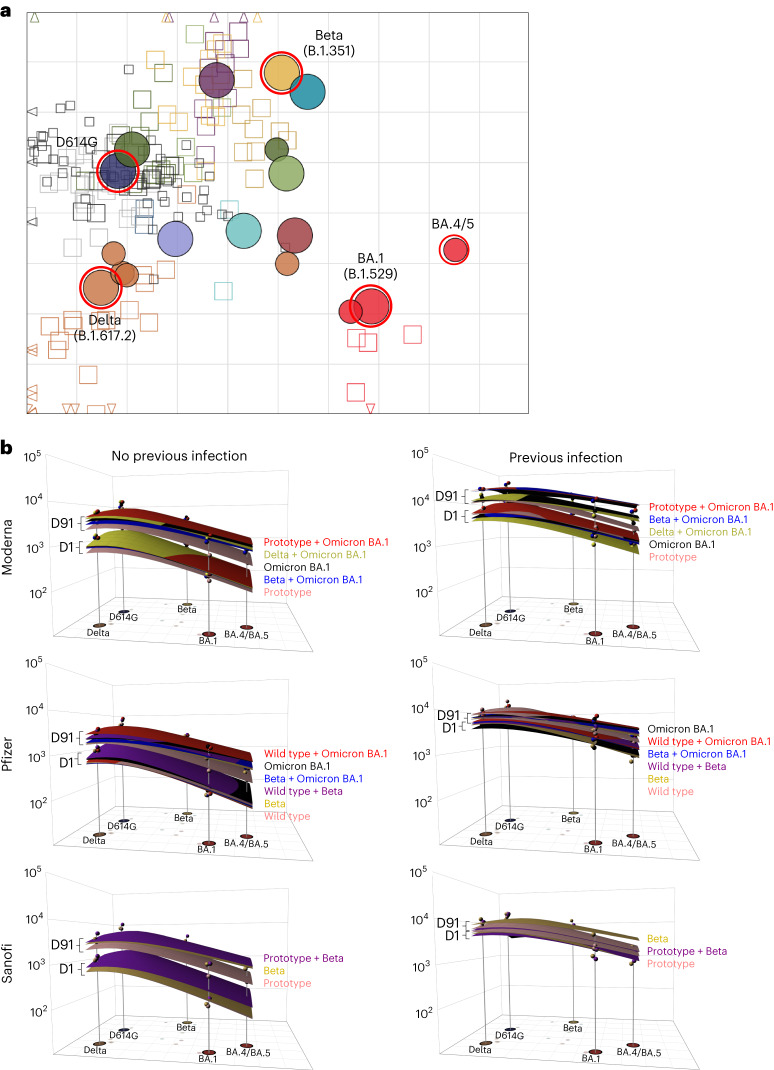
Antigenic cartography. **a**, An antigenic map by ref. ^25^ served as the base map for all antibody landscapes. Virus variants are shown as color-filled circles. Variants with additional substitutions from their root variant are shown as smaller circles. Variants associated with significant outbreaks or pandemic waves are secondarily encircled in red. Individual sera from individuals who were infected are displayed as open squares in the color of their root variant or gray for mRNA-1273 vaccinated sera; small dark squares represent clinical trial participants. One grid unit in the map corresponds to a twofold dilution in the neutralization assay. Within the *x* and *y* axes, the map orientation is free as antigenic distances are relative. Small triangles point to sera outside the shown map area. **b**, Day 1 and day 91 GMT antibody landscapes for individuals who were uninfected and infected in different arms for the three stages. Impulse lines extending from the base map to the landscapes show the GMT against the specific variant. Lower landscapes correspond to day 1 and upper landscapes to day 91 immunity. To interpret landscapes, a day 91 response where the upper landscape is flat indicates the responses to all the variants were equivalent, whereas skewing up or down indicates an uneven response across variants. The landscapes are ordered by height of their GMT against BA.4/5. The surface colors represent study arms: pink, prototype; red, prototype + Omicron BA.1; black, Omicron BA.1; light green, Delta + Omicron BA.1; blue, Beta + Omicron BA.1; purple, Beta + prototype; yellow, Beta.

Antigenic cartography is a method to visualize antigenic relationships of virus variants in a two-dimensional map, where the distance in the map corresponds to neutralization properties of the variants^23^. We constructed antibody landscapes^24^, where neutralization titers are plotted in a third dimension above the variants in an antigenic map, to visualize how immunity in the different study arms distributes across antigenic space. The base map we used here was derived from a map by ref. ^25^ (Fig. 4a). Figure 4b shows the GMT antibody landscapes for each vaccine arm in the three stages stratified by prior infection, with the corresponding neutralizing antibody titers above the variant’s map position. Lower landscapes correspond to day 1 and upper landscapes to day 91 immunity. To interpret landscapes, a day 91 response where the upper landscape is flat indicates the titers to all the variants were equivalent, whereas skewing up or down indicates titer differences across variants. The surface colors represent individual study arms.

All vaccine arms for each of their respective stages in participants who were uninfected had similar prevaccination antibody landscapes, with the apex over D614G, as expected (Fig. 4b). After vaccination, all arms, in all 3 stages, had antibody titers that raised and flattened the landscape. In uninfected cohorts for all three stages, variant-containing vaccines lifted titers against BA.1 and BA.4/5 and produced flatter landscapes in the antigenic space surrounding these variants than did the prototype or wild-type vaccines. A second booster dose raised antibody titers in participants who were uninfected to the titers observed in participants who were previously infected at baseline (Fig. 4b).

### SARS-CoV-2 infections

There were 267 self-reported COVID-19 illnesses occurring after randomization among 973 participants in single dose arms by data cut-off, 1 of which resulted in a brief hospitalization, lasting less than 24 hours, due to hypoxemia. The incidence of infections in this trial reflect the community transmission, with the majority occurring during the Omicron BA.5 wave in the United States. At any point in time, participants from different stages were in different points in follow-up, thereby preventing assessment of incidence across stages. Kaplan–Meier (KM) estimates of infections at the end of the follow-up period were similar among arms within a stage (Supplementary Tables 4–6). A higher percentage of infections, across all stages, was noted in participants with no history of prior infection (KM estimate, 37.8%; 95% CI, 31.8%, 44.6%) compared with those with a history of prior infection (KM estimate, 12.1%; 95% CI, 8.4%, 17.2%). There were also fewer infections in adults ≥65 years (KM estimate, 19.3%; 95% CI, 15.1%, 24.5%) compared with their younger counterparts (KM estimate, 36.2%; 95% CI, 29.2%, 44.4%) across all stages.

## Discussion

The continued emergence of SARS-CoV-2 VOCs led to a recommendation to update COVID-19 vaccines^26^. The strains selected in 2022 for modified vaccines covered circulating strains at the time of vaccine development, not necessarily variants that would drift antigenically from Omicron BA.1 and BA.4/5 or evolve from other distinct locations on the phylogenetic tree. Therefore, it is important to investigate not only immune responses to known variants but also the antigenic relationships among different SARS-CoV-2 VOCs^25^ and how variant vaccines may alter immunologic landscapes to cover antigenic areas where new strains may emerge. Here we described the magnitude, breadth and landscapes of the neutralizing antibody response following a second booster with investigational monovalent and bivalent variant-specific vaccines reflective of the diverse SARS-CoV-2 immunologic background seen in the general population. Our randomized study, using different vaccine platforms, offers the most comprehensive assessment of how vaccination with variants antigenically distinct from the ancestral strain compare in the ability to produce a broadly crossneutralizing antibody response and provides several insights to inform future SARS-CoV-2 vaccine policy.

First, our findings support that mRNA and adjuvanted protein variant vaccines elicit substantial crossreactive neutralizing antibodies to D614G and to B.1.351, 1.617.2, Omicron BA.1, Omicron BA.4/5 and other Omicron subvariants, regardless of prior SARS-CoV-2 infection history and age. This is probably due to ongoing antibody somatic mutation, memory B cell clonal turnover and development of antibodies that are resistant to SARS-CoV-2 spike protein RBD mutations^27,28^.

Second, our ANCOVA modeling demonstrated that the mRNA variant vaccines offered a clear serologic advantage over the wild-type/prototype vaccines against B.1.351, BA.1 and BA.4/5 that persisted up to 3 months after vaccination. Moreover, vaccine candidates without Omicron BA.1 variant, such as the Pfizer mRNA Beta vaccine, still provided superior heterologous coverage to Omicron BA.1 and BA.4/5 when compared with the wild-type vaccine, which was probably due to the common mutations in the spike RBD (K417N, E484K/A and N501Y) between B.1.351 and these Omicron variants. Although a serologic advantage to BA.1 was not seen in ANCOVA modeling with the Sanofi Beta or Beta + prototype protein vaccine candidates, perhaps due to small sample size or undetected prior infection, a similar serologic benefit of boosting with the Beta monovalent vaccine^29,30^, and superior clinical efficacy against Omicron BA.1 and BA.2, was seen in the manufacturer’s phase 3 clinical trial^31^.

The antibody landscapes visualizing the neutralization profile after vaccination further support inclusion of variants in booster vaccines. After vaccination, the antibody landscape rises with variant vaccine candidates, especially against more recent variants, and flattens the antibody landscape more than the prototype vaccine, suggesting there may be higher titers of neutralizing antibodies with variant-containing vaccines against future VOCs, especially if they emerge near B.1.351, Omicron BA.1 and BA.4/5 (ref. ^32^).

Although specific correlates of protection for infection with recent Omicron subvariants are not well understood, neutralizing antibody titers have been used to infer protection during the D614G wave of the pandemic, when the circulating virus closely matched the vaccine strain^33^, and the resulting immunologic data have served as the basis for emergency-use authorization for booster vaccines by regulatory agencies^34,35^. The improved serologic response with either Omicron BA.1 or Beta variant-containing vaccines over prototype/wild-type vaccines in our study and others^36–38^ provides evidence that broad crossprotection may be conferred without a variant-chasing approach and warrants further mechanistic exploration.

For all vaccine candidates, including vaccine products not containing prototype, the antibody titers were higher against D614G compared with the VOCs, supporting the hypothesis of back-boosting to the ancestral strain seen in previous studies^24,37,38^. This suggests that future generations of SARS-CoV-2 vaccines may be able to omit prototype or wild-type sequences without losing the ability to neutralize D614G, or other variants within close antigenic distance, in people who previously received the prototype vaccines. Furthermore, Omicron BA.1 or Beta monovalent vaccines were nearly equivalent to Omicron BA.1 + prototype or Beta + prototype bivalent vaccines for neutralization of B.1.351 and both Omicron subvariants (BA.1 and BA.4/5), further supporting the premise that monovalent variant vaccines could replace bivalent vaccines as the updated boost in the future^31^.

Notably, although variant vaccines improved neutralizing activity against Omicron subvariants, these titers decreased for more recent Omicron subvariants. Although the serum inhibitory dilution required for 50% neutralization (ID_50_) against BA.1 and BA.4/5 remained high, the neutralization titers for subvariants BQ.1.1 and XBB.1 were much lower. In addition, we noted a high rate of infections that occurred during the BA.4/5 wave and subsequent waves with XBB.1 and BQ.1.1. These infections occurred more frequently in individuals who were previously uninfected compared with those who were previously infected, highlighting the importance of hybrid immunity in protection against disease^32^. In addition, infections occurred in younger rather than older adults, probably reflecting behavioral differences affecting risk of exposure. Our study was not designed to assess VE. Although recent data suggest possibly higher VE against Omicron subvariants with bivalent vaccine boosts (prototype + Omicron BA.4/5 and prototype + Omicron BA.1) compared with the prototype vaccine^18,39^, our findings highlight concerns that variant vaccines are unlikely to keep pace with virus evolution and that other immune correlates of protection beyond antibody responses need to be explored.

Our study has several limitations. First, the sample size is small for certain subgroups of interest, such as prior infection (27%) and adults older than 65 years (31%). Second, T cell responses and antibody effector functions, which may be critical to preventing severe disease^40^, have not yet been evaluated. In addition, clonal and kinetic analyses of the memory B cell response, although underway, are not available to further differentiate the durability of the antibody response elicited by variant-containing vaccines. Finally, participants were only randomized to different arms within each stage and not between stages that enrolled sequentially at different calendar times, leading to different exposures to circulating variants before and after enrollment. This precludes head-to-head comparisons of rates of infections or neutralization titers across stages. These results may also not extend to adenovirus vector or inactivated vaccines licensed and used more frequently in other parts of the world or future next-generation vaccines.

In conclusion, these data demonstrate that updating vaccines to target recent variants provides modestly improved and broadly crossprotective neutralizing antibody responses against diverse SARS-CoV-2 variants without sacrificing boosting immunity to the ancestral strain. The precise degree to which the enhanced antibody response elicited by updated vaccines will restore protection against disease after infection with heterologous or homologous strains needs further confirmation by real-world effectiveness studies. Our study incorporating both antigenic distances and serologic landscapes serve as a framework for objectively guiding decisions for future vaccine updates.

## Methods

### Study design and eligibility criteria

This phase 2, open-label, randomized clinical trial was performed at 22 sites in the United States (Supplementary Table 1). This trial comprised multiple stages, each of which was designed independently and enrolled sequentially, taking into consideration vaccine selection in the previous stages and the availability of new variant vaccine products from different manufacturers across more than one platform.

The trial was sponsored and funded by the National Institutes of Health (NIH). The National Institute of Allergy and Infectious Diseases (NIAID) SARS-CoV-2 Assessment of Viral Evolution (SAVE) program team was consulted to inform study arm design and variant vaccine selection. The trial was reviewed and initially approved by the Advarra Central Institutional Review Board on 22 March 2022 and overseen by an independent data and safety monitoring board. There have been three subsequent amendments to the protocol to address updates to risk and benefits of the study related to myopericarditis as well as US Food and Drug Administration approval of a second booster vaccine for older adults (amendment 2), and with the design of additional stages (amendments 3 and 4). Written informed consent was obtained from all trial participants before enrollment. A stipend was provided for participation in the study, which was determined by each enrolling site.

From 30 March to 6 May 2022, 602 eligible participants were enrolled in stage 1 (Table 1 and Fig. 1). From 12 to 27 May 2022, 313 eligible participants were enrolled in stage 2. From 8 to 17 June 2022, 153 eligible participants were enrolled in stage 3. Eligible participants were healthy adults 18 years of age and older (with or without a history of prior SARS-CoV-2 infection) who had received a primary series and a single homologous or heterologous boost with an approved or emergency-use-authorized COVID-19 vaccine (Supplementary Table 2). The most recent vaccine dose and/or prior infection must have occurred at least 16 weeks before randomization. Full eligibility criteria are described at ClinicalTrials.gov (NCT05289037).

Eligible participants were stratified by age (18–64 and ≥65 years) and a self-reported history of confirmed SARS-CoV-2 infection, and randomly assigned across arms within each stage in an equal ratio using block-randomization methodology, with blocks of size 6 and 12 for stages 1 and 2 and blocks of size 3 and 6 for stage 3. Subjects were randomized using the Advantage eClinical system used by the Statistical Data Coordinating Center. As this was an unblinded study, no effort was made to conceal the assignment postrandomization. Sample size was chosen to be able to detect common AEs and estimate immunogenicity parameters with acceptable precision (see the protocol in Supplementary Information for further details). After providing informed consent, participants underwent screening, including confirmation of COVID-19 vaccination history, medical history, a targeted physical examination and a urine pregnancy test (if indicated). Safety and immunogenicity assessments were performed on days 1, 15 and 29, and at 3, 6, 9 and 12 months after last vaccination. Although the study was not designed to evaluate booster VE, we collected information on antigen or PCR-confirmed symptomatic or asymptomatic SARS-CoV-2 infection at any time after randomization. A nasal swab sample was collected for viral sequencing in persons testing positive. Immunologic data are currently available up to day 91 visit after first vaccination. The safety data cut-off was 2 December 2022.

### Trial vaccines

Trial vaccines are listed in Table 1 and Extended Data Table 1. Trial vaccines were provided by Moderna for stage 1 (50 µg per vaccine), Pfizer–BioNTech for stage 2 (30 µg per vaccine) and Sanofi for stage 3 (5 µg per vaccine). The vaccine candidates were manufactured similarly to their corresponding authorized or approved vaccines in the United States or Europe.

### Study outcomes

The primary objective was to evaluate humoral immune responses of candidate SARS-CoV-2 variant vaccines, alone or in combination. The secondary objective was to evaluate the safety of candidate SARS-CoV-2 variant vaccines assessed by solicited injection-site and systemic AEs, which were collected for 7 days after vaccination; unsolicited AEs through day 29; and serious AEs, new-onset chronic medical conditions, AEs of special interest, AEs leading to withdrawal and medically attended AEs through the duration of the trial.

Exploratory objectives included sequencing strains from infections for variant spike lineage and assessing anti-N serology. Information on antigen- or PCR-confirmed symptomatic or asymptomatic SARS-CoV-2 infection at any time after randomization was collected.

### Immunogenicity assays

SARS-CoV-2 neutralization titers, expressed as the ID_50_, were assessed using pseudotyped lentiviruses presenting SARS-CoV-2 spike mutations for the D614G (Wuhan-1 containing a single D614G spike mutation), B1.617.2 (Delta), B.1.351 (Beta) and B.1.1.529 (Omicron BA.1) variants, as described previously^22,41^. A random subset of samples (25 per selected vaccine arm, distributed equally between age strata and sites) was analyzed for neutralization titers to the Omicron BA.4/BA.5, BA.2.12.1, BA.2.75.2, BA.2.75, BA.4.6, BF.7 and BQ.1.1 subvariants in a separate laboratory^42^. Electrochemiluminescence immunoassays were used for the detection of anti-N (Elecsys Anti-SARS-CoV-2 N; Roche) at baseline^41^.

### Statistical analysis

The primary objective of this study was to evaluate the magnitude, breadth and durability of SARS-CoV-2-specific antibody titers in serum samples by estimating 95% CIs for the GMT at each time point when samples were collected. No prespecified formal hypothesis tests were planned. The GMFR was calculated as the geometric mean of titers at a time point divided by titers at day 1. The GMR_D614G_ is the geometric mean of the ratio of D614G titers against titers for a VOC. Seropositive rate was calculated as the proportion of participants with titers above the lower limit of detection (LLOD). The 95% CIs for GMT, GMFR and GMR_D614G_ were calculated using the Student’s *t*-distribution, and 95% CIs for seropositive rate were calculated using the Clopper–Pearson binomial method. Assumptions on neutralizing titers and variability were made to determine the precision with which GMTs could be estimated. Acceptable precision would then allow numeric comparison with reasonable CI width, even if power had been low for hypothesis testing.

No imputation was done for missing data. However, any values below the LLOD were imputed as half of the LLOD. Participants with a SARS-CoV-2 infection occurring between vaccination and a prespecified immunogenicity time point were excluded from immunogenicity analysis at that time point and thereafter. For the purpose of analysis, participants were defined as previously infected by self-report of a confirmed positive antigen or PCR testing or the detection of anti-SARS-CoV-2 N antibodies at enrollment.

ANCOVA models were used to estimate GMT ratios of variant vaccines compared with the prototype vaccine and included independent variables for vaccination arms, age (18–64 years and ≥65 years of age), previous infection history and baseline titers. For modeling purposes, titers were log_10_-transformed and estimated mean differences were back-transformed to generate GMT ratios between vaccination groups. Unadjusted 97.5% CIs based on the *t*-distribution are reported.

Infection rates were estimated using KM methodology.

All analyses were done in SAS v.9.4 or R v.4.2.2 or higher.

### Antigenic cartography and antibody landscapes

Antigenic cartography uses antibody neutralization data to position virus variants and sera relative to each other in an *n*-dimensional Euclidean space, in this case a two-dimensional space, as previously described^23^. The distance between variants can be understood as a measure of antigenic similarity. Briefly, for each serum–variant pair, the fold-change from the maximum titer variant in the specific serum was calculated to obtain a target distance from the serum. Serum and variant coordinates were then optimized such that difference between Euclidean map distance and this target distance was minimized, with one map unit corresponding to one twofold dilution of neutralization titers on the log_2_ scale. Here, the antigenic map published in ref. ^25^ was used as the basis for the antibody landscapes, where neutralization titers against virus variants were plotted in a third dimension above the corresponding variant in an antigenic map and a continuous surface was fitted to these titers^24^. Antibody landscapes were constructed using the ablandscape.fit function^24,43^, of the ablandscape package (v.1.1.0, R v.4.2.0) with the parameters method = ‘cone’, error.sd = 1, bandwidth = 1, degree = 1, control = list(optimise.cone.slope = TRUE). Variant coordinates from the base map were used to fit a single-cone surface to neutralization titers against D614G, B.1.351, B.1.617.2, BA.1 and BA.4/5 for each serum. Per arm, the surface slope was optimized to match prevaccine and 3 months postvaccine neutralization titers. Samples from participants who were nonresponding, defined as a titer of 20 (LLOD/2) against all variants at either time point were not included (*n* = 12 in the uninfected cohort, *n* = 3 in the infected cohort).

### Reporting summary

Further information on research design is available in the Nature Portfolio Reporting Summary linked to this article.

## Online content

Any methods, additional references, Nature Portfolio reporting summaries, source data, extended data, supplementary information, acknowledgements, peer review information; details of author contributions and competing interests; and statements of data and code availability are available at 10.1038/s41591-023-02503-4.

## Supplementary information

Supplementary Information Supplementary Tables 1–12, Eligibility criteria, and Study team and study group.

Reporting Summary

## Figures and Tables

**Extended Data Table 1:**
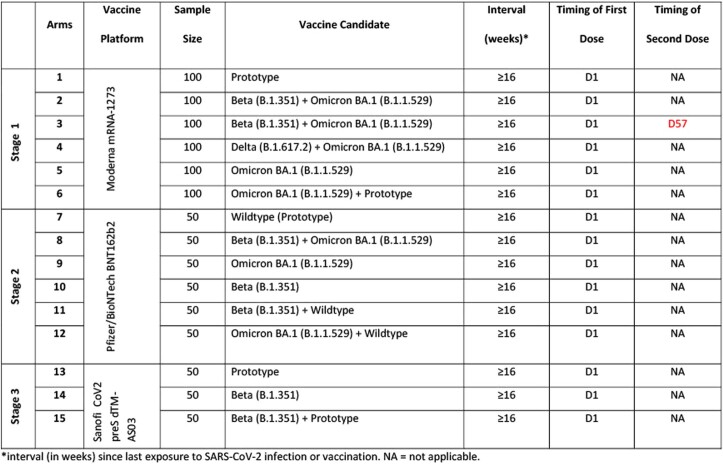
Study arms

**Extended Data Table 2:**
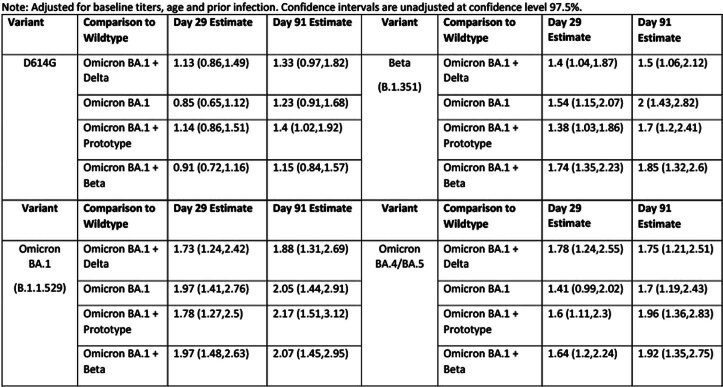
Stage 1 (Moderna) adjusted pseudovirus neutralization day 29 and day 91 GMR ANCOVA modeling

**Extended Data Table 3:**
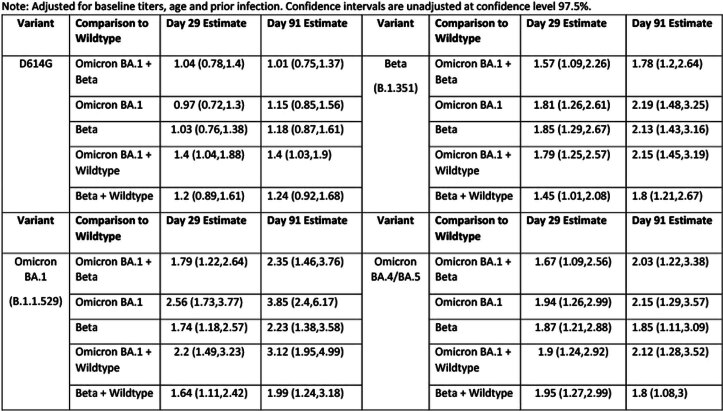
Stage 2 (Pfizer) adjusted pseudovirus neutralization day 29 and day 91 GMR ANCOVA modeling

**Extended Data Table 4:**
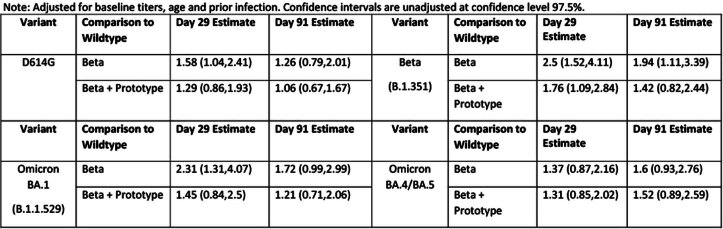
Stage 1 (Sanofi) adjusted pseudovirus neutralization day 29 and day 91 GMR ANCOVA modeling

## Data Availability

All data are included in the paper. The protocol for the study is provided in Supplementary Information.

## Code availability

The code for antibody landscapes and titer line plots is publicly available in a GitHub repository (https://github.com/acorg/branche_et_al2023)^44^.
