# Supplementary material for: Comparison of bivalent and monovalent SARS-CoV-2 variant vaccines: the phase 2 randomized open-label COVAIL trial
Source: Nat Med. 2023 Aug 28;29(9):2334–46. doi: 10.1038/s41591-023-02503-4 (PMC10504073; doi:10.1038/s41591-023-02503-4)
Supplement: Supplementary file 1 — Supplementary Tables 1–12, Eligibility criteria, and Study team and study group. [file 41591_2023_2503_MOESM1_ESM.pdf]

nature medicine

Article

https://doi.org/10.1038/s41591-023-02503-4

Comparison of bivalent and monovalent SARS-CoV-2 variant vaccines: the phase 2 randomized open-label COVAIL trial

In the format provided by the authors and unedited

Supplementary Appendix

This appendix is submitted by the authors to provide additional information about their work.

Table of Contents

COVAIL Manuscript Study Group......6

COVAIL Study Team Members......11

Supplemental Methods

Eligibility criteria......18

Supplemental Tables

Table S1. List of COVAIL US Sites......20

Table S2. Eligible Regimens for Primary Vaccination Series and Boost......21

Table S3 Breakthrough Infections During the Study by Stage and Arm through December 2, 2022...... 22

Table S4 Breakthrough Infections During the Study by Stage and Prior Infection Status......23

Table S5 Breakthrough Infections During the Study by Stage an Age at Enrollment...... 24

Table S6. Stage 1 (Moderna) Pseudovirus Neutralization Assay Summary Results by Arm, Time Point and Variant for Uninfected Participants...... 25

Table S7. Stage 1 (Moderna) Pseudovirus Neutralization Assay Summary Results by Arm, Time Point and Variant for Previously Infected Participants...... 32

Table S8. Stage 2 (Pfizer) Pseudovirus Neutralization Assay Summary Results by Arm, Time Point and Variant for Uninfected Participants......38

Table S9. Stage 2 (Pfizer) Pseudovirus Neutralization Assay Summary Results by Arm, Time Point and Variant for Previously Infected Participants......43

Table S10. Stage 3 (Sanofi) Pseudovirus Neutralization Assay Summary Results by Arm, Time Point and Variant for Uninfected Participants......48

Table S11. Stage 3 (Sanofi) Pseudovirus Neutralization Assay Summary Results by Arm, Time Point and Variant for Previously Infected Participants......51

Table S12. Stage 1 (Moderna) Pseudovirus Neutralization Assay Summary Results by Arm, Time Point and Omicron Subvariant for a Subset of Uninfected Participants. 54

Study Protocol..56

COVAIL Manuscript Study Group

George Washington Vaccine Research Unit, George Washington University, Washington D.C.

David J. Diemert, MD; Elissa Malkin, DO; Jeffrey M. Bethony, PhD; Aimee Desrosiers, PA-C; Marc Siegel, MD

University of Rochester VTEU, Rochester, NY

Angela R. Branche, MD, Ann R. Falsey, MD, Edward Walsh, MD, Patrick Kingsley, BS, Michael Peasley, BS

Emory University Hope Clinic, Decatur, GA

Nadine G. Rouphael, MD; Cecilia Losada, MD; Daniel S. Graciaa, MD; Hady Samaha, MD; Cassie Grimsley Ackerley, MD; Kristen E. Unterberger, PA

Brigham and Women's Hospital, Harvard Medical School, Boston, MA

Lindsey R. Baden, MD; Amy C. Sherman, MD; Stephen R. Walsh, MD; Alexandra Tong, BS; Rebecca Rooks, BS

Center for Vaccine Development, Saint Louis University, St. Louis, MO

Sharon E. Frey, MD; Getahun Abate, MD, PhD; Zacharoula Oikonomopoulou, MD; Daniel F. Hoft, MD, PhD; Irene Graham, MD

Departments of Molecular Virology and Microbiology and Medicine, Baylor College of Medicine, Houston, TX

Jennifer A. Whitaker, MD; Hana M. El Sahly, MD; Wendy A. Keitel, MD; C. Mary Healy, MD; Robert L. Atmar, MD

Department of Medicine, Division of Infectious Diseases and Global Public Health, University of California San Diego, La Jolla, CA

Susan J. Little, MD; Thomas C.S. Martin, MD; Nicole Carter, MPH; Steven Hendrickx, RN

Center for Childhood Infections and Vaccines (CCIV) of Children's Healthcare of Atlanta and Emory University Department of Pediatrics, Atlanta, GA

Evan J. Anderson, MD; Christina A. Rostad, MD; Satoshi Kamidani, MD PhD; Etza Peters, RN; Lauren Nolan, PA

Duke Human Vaccine Institute, Duke University School of Medicine, Durham, NC

Emmanuel B. Walter MD, MPH; Michael J. Smith MD, MSCE; M. Anthony Moody, MD; Kenneth E. Schmader, MD

University of Illinois at Chicago-Project WISH, Chicago, IL

Richard M. Novak, MD; Benjamin G. Ladner, MD; Andrea Wendrow, RPh; Jessica Herrick, MD

University of Texas Medical Branch, Galveston, TX

Richard Rupp, MD; Laura Porterfield, MD

Kaiser Permanente Washington Health Research Institute, Seattle, WA

Lisa A. Jackson, MD, MPH; Maya Dunstan, MS, RN; Rebecca Lau, PharmD; Barbara Carste, MPH

Departments of Medicine, Epidemiology, and Laboratory Medicine & Pathology, University of Washington, Vaccines and Infectious Diseases Division, Fred Hutchinson Cancer Center, Seattle, WA

Tara M. Babu, MD, MSCI; Anna Wald, MD, MPH; Taylor Krause, BA; Kirsten Hauge, MPH

NYU VTEU Manhattan Research Clinic at NYU Grossman School of Medicine, New York, NY

Angelica C. Kottkamp, MD; Tamia Davis, NP; Celia Engelson, NP; Vijaya Soma, MD

Zuckerberg San Francisco General, University of California San Francisco, San Francisco, CA

Anne F. Luetkemeyer, MD; Chloe Harris, BA; Azquena Munoz Lopez, BS

Morehouse School of Medicine, Clinical Research Center's Vaccine Trial Unit, Atlanta, GA

Lilly C. Immergluck, MD; Erica Johnson, PhD; Austin Chan, MD; Fatima Ali, MPH; and Trisha Parker, MPH

Washington University School of Medicine, St. Louis, MO

Rachel M. Presti, MD, PhD; Jane A. O'Halloran, MD, PhD; Ryley M. Thompson

NYU VTEU Long Island Research Clinic at NYU Long Island School of Medicine, Mineola, NY

Martín Bäcker, MD; Kimberly Byrnes, RN; Asif Noor, MD

University of Iowa College of Medicine, Iowa City, IA

Patricia L. Winokur, MD; Jeffery Meier, MD; Jack Stapleton, MD

Howard University College of Medicine, Howard University Hospital, Washington D.C.

Siham M. Mahgoub, MD; Celia Maxwell, MD; Sarah Shami, PharmD

Department of Medicine, University of Alabama at Birmingham, Birmingham, AL

Paul A. Goepfert, MD

Tulane University School of Medicine, New Orleans, LA

Dahlene N. Fusco, MD; Arnaud C. Drouin, MD; Florice K. Numbi, MD

IDCRC Principal Investigators

David S. Stephens, MD; Kathleen M. Neuzil, MD

IDCRC Leadership Operations Center

Monica M. Farley, MD; Jeanne Marrazzo, MD; Sidnee Paschal Young

IDCRC Clinical Operations Unit

Jeffery Lennox, MD; Robert L. Atmar, MD; Linda McNeil FHI360

IDCRC Statistical and Data Science Unit

Elizabeth Brown, PhD

IDCRC Laboratory Operations Unit – Fred Hutchinson Cancer Center, Seattle, WA

Christine M. Posavad, PhD; Megan A. Meagher, BS; Julie McElrath, MD; Mike Gale, PhD

The Emmes Company, LLC, Rockville, MD

Mat Makowski, PhD; Heather Hill, MS; Jim Albert, MS, Holly Baughman; Lisa McQuarrie, MS; Kalyani Telu, MS; Jinjian Mu, PhD

Clinical Monitoring Research Program Directorate, Frederick National Laboratory for Cancer Research, Frederick, MD

Teri C. Lewis, BS; Lisa A. Giebeig, MS; Theresa M. Engel, MFS; Caleb J. Griffith, MPH; Wendi L. McDonald, BSN; Alissa E. Burkey, MS; Lisa B. Hoopengardner, MS; Jessica E. Linton, MS; Nikki L. Gettinger, MPH

Division of Microbiology and Infectious Diseases, National Institute of Allergy and Infectious Diseases, National Institutes of Health, Bethesda, MD.

Marina Lee, PhD; Mamodikoe Makhene, MD; Mohamed Elsafy, MD; Rhonda Pikaart-Tautges, BS; Janice Arega, MS; Binh Hoang, RPh; Dan Curtin; Hyung Koo, BSN; Elisa Sindall, BSN; Sonja Crandon, BSN; Seema U. Nayak, MD; Marciela M DeGrace, PhD; Diane J Post, PhD; Paul C Roberts, PhD; John H Beigel, MD.

COVAIL Manuscript Study Team Members

George Washington Vaccine Research Unit, George Washington University, Washington D.C.

David J. Diemert, MD; Elissa Malkin, DO; Jeffrey M. Bethony, PhD; Aimee Desrosiers, PA-C; Marc Siegel, MD; Nikita Schroll-McLaughlin, MS; Jonathan Manning, BA; Jane Ryu, MS; Hanna-Grace Rabanes, MPH; Khadija Khan, MPH; Laura Vasquez, MPH; Caroline Thoreson, PA-C; Larissa Scholte, PhD; Rafaela Thur, DVM; Peyton St. John, BS; Dorinne Mettle-Amuah, PharmD

University of Rochester VTEU, Rochester, NY

Angela R. Branche, MD, Ann R. Falsey, MD, Edward Walsh, MD, Patrick Kingsley, BS, Arthur Zemanek, BSN, MS Katherine Elena, BSN, Spencer Obrecht, BSN, Ian Shannon, BSN, Amy Kaychalo, BS, MS, Erin Nowicki, Sharon Moorehead, Kari Steinmetz, BA, Doreen Francis, RN, Tanya Smith, BS, William Hamilton, BS, Jeanne Holden-Wiltse, MPH, MBA, Christopher Lane, MS, Michael Peasley, BS, Samuel Diehl, BS, Kyle Richards, PharmD, Stephen Bean, PharmD, Nicole Dornbush, PharmD, Carol Cole, PharmD

Emory University Hope Clinic, Decatur, GA

Nadine G. Rouphael, MD; Cecilia Losada, MD; Daniel S. Graciaa, MD; Hady Samaha, MD; Cassie Grimsley Ackerley, MD; Kristen E. Unterberger, PA; Amy Anderson, BSN; Mary Atha, ACNP; Kareem Bechnak, BSN; Sarah Bechnak, BSN; Mary Bower, BSN; Laura Clegg, RN; Matthew Collins, MD, PhD; Francine Dyer, RN; Srilatha Edupuganti, MD; Rebecca Fineman, BS; Tigisty Girmay, MSN; Rebecca Gonzalez, PharmD; Natalie Gray, BS; Evan Gutter, MPH; Lisa Harewood; Chris Huerta, MSc; Brandi Johnson, BS; Lauren Johnson, MPH; Colleen Kelley, MD; Alexandra Koumanelis, BA; Deborah Laryea, BSN; Hollie Macenczak, BSN; Nour Makkaoui, MD; Michele McCullough, MPH; Tuong-Vy Ngo,

PharmD; Eileen Osinski, BS; Julia Paine, BS; Bernadine Panganiban, BS; Rose Pope, RN; Paulina Rebolledo, MD; Susan Rogers, RPh; Erin Scherer, PhD; Veronica Smith, NP-C; Andre Stringer, BS; Jessica Traenkner, PA; Dongli Wang, BS; Alahna Watson, BA; Stacey Wheeler, RN; Jean Winter; Jianguo Xu, PhD

Brigham and Women's Hospital, Harvard Medical School, Boston, MA

Lindsey R. Baden, MD; Amy C. Sherman, MD; Stephen R. Walsh, MD; Alexandra Tong, BS; Rebecca Rooks, BS; Jane A. Kleinjan, NP; Jon A. Gothing, NP; Andres A. Avila Paz, BA; Muneerah M. Aleissa, PharmD, MPH; Bethany Evans, BA, August Heithoff, BS; Natalie E. Izaguirre, MS; Hannah Jin, MPH; Urwah Kanwal, BS; Austin Kim, BS; Julia E. Klopfer, BS; Christina Montesano, BS; John Almeida, BA; Emily S. Koleske, BS; Hannah Levine, BS; Nicholas P. Morreale, BS; Omolola Ometoruwa, BS; Jun Bai Park Chang, BS; Anna F. Piermattei, BA; Djenane M. Pierre, BS; Megan Powell, BA; Kevin Zinchuk, PharmD; Stephanie Pickford, PharmD; Charles M. Kelly III, PharmD; Xiaofang Li, PhD; John Kupelian, BS ; Kimberly Dufresne, BS; Xiaoguang Fan, MD, PhD; Xi Zhang, PhD; Esther Arbona-Haddad, MD; José Humberto Licona, MD

Center for Vaccine Development, Saint Louis University, St. Louis, MO

Sharon E. Frey, MD; Getahun Abate, MD, PhD; Zacharoula Oikonomopoulou, MD; Daniel F. Hoft, PhD, MD; Irene Graham, MD; Azra Blazevic, DVM, MPH; Tamara Blevins, MS; Kathleen Chirco, BSN; Sabrina M. DiPiazza, BSN, MA; Stanley Doublin; Heather Hoertel Douds, MSNS, BSN; Carol G. Duane, PhD, RN; Eric Eggemeyer, BA; Linda M. Eggemeyer-Sharpe, BSN; Lauren Nicole Foreman, BSN; Sarah Louise George, MD; Geoffrey J. Gorse, MD; Michelle Harris, PharmD; Helay Hassas, PharmD; Rong Hou, MD; Ryan Clark Kerr, BSN; Kate Elizabeth Liefer, BSN; Melissa J. Loyet, RN; Lainey Mejia-Jauregui, BS; Keith Meyer, BS; Tracy Renee Montauk, BSN; Karla J. Mosby, RN; Amanda Nethington, BS; Huan Ning, MD;

Nicole Purcell; Joan M. Siegner, BSN, MA; Janice M. Tennant, BSN, MPH; Mei Xia, PhD; Kiana Wilder, BA; Yinyi Yu, BS; Cassandra Nicole Zehenny, BSN

Departments of Molecular Virology and Microbiology and Medicine, Baylor College of Medicine, Houston, TX

Jennifer A. Whitaker, MD; Hana M. El Sahly, MD; Wendy A. Keitel, MD; C. Mary Healy, MD; Robert L. Atmar, MD; Pedro A. Piedra, MD; Jesus Banay; Kathy Bosworth; Janet Brown, RPh; Kayla Burrell; Jeremy Castro; Tykel Eddy; Marcena Eubanks; Cathy Faw, RPh; Rachel Froebe; Alix Halter, RN; Janey John, MSN, APRN, FNP-C; Chanei Henry, AAS; Vanessa Martinez; Carol Mundell, RN; Brandie Phillips, RN; Alicia Prevost-Barthe, RN; Connie Rangel, RN; Yolanda Rayford, MS; Yvette Rugeley; Maria Shlyapobersky; Tina Sierra; Elizabeth Silguero; Lisreina Toro; Dawn Turner, RN; Chianti Wade-Bowers, RN; Jessica Woods, RN

Department of Medicine, Division of Infectious Diseases and Global Public Health, University of California San Diego, La Jolla, CA

Susan J. Little, MD; Thomas C.S. Martin, MD; Nicole Carter, MPH; Steven Hendrickx, RN; Ajay Bharti, MD; Alyssa Phillips; Aurora Verduzco Gonzalez, NP; Cheryl Dullano; Chris Houston; Dawn Rosenblum, RN; DeeDee Pacheco; DeLys Brooks; Fang Wan; Helene Le, CPhiT; JC Alcantar; Jill Blumenthal, MD; Joseph Lencioni, MABMH; Kory Hess; Letty Muttera, PharmD; Marlene Arredondo; Megan Smyth; Megan Taylor; Melinda Stafford, PharmD; Michelle Orsburn, MD; Michelle Truong; Niamh Higgins, PharmD, MSc, AAHIVP; Nimish Patel, PharmD, PhD, AAHIVP; Rebecca Gonzalez; Vivian Maldonado

Center for Childhood Infections and Vaccines (CCIV) of Children's Healthcare of Atlanta and Emory University Department of Pediatrics, Atlanta, GA

Evan J. Anderson, MD; Christina A. Rostad, MD; Satoshi Kamidani, MD, PhD; Etza Peters, RN; Larry Anderson, MD; Julia Bartol; Leisa Bower RN; Natsuko Campbell RN; Lisa Harewood, Hui-Mien Hsiao, Laila Hussaini MPH, Inara Jooma, Gidget Kettle RN; Marcia Lewis RN; Wensheng Li; Cindy Lubbers RN; Lisa Macoy RN; Molly Morrison, Heather Nurse RN; Anna Siaw-Anim; Kathleen Stephens RN; Madeline Taylor; Ashley Tippett MPH; Lauren Nolan PA

Duke Human Vaccine Institute, Duke University School of Medicine, Durham, NC

Emmanuel B. Walter MD, MPH; Michael J. Smith MD, MSCE; M. Anthony Moody, MD; Kenneth E. Schmader, MD; Susan Doyle; Lynn S Harrington BSN; Lori Hendrickson BSN; Amy O'Berry MSN; Sherry Huber BSN; Janet Wootton RN, RSCN; Kelly Clark BA; Lani Banez; Stephanie Smith BA; Byron Hauser BS; Ally Odom BA; Emily Randolph BA; Krystina Yoder BA; Kathlene Chmielewski; Luis Ballon BA; Aubree Latorre; Breana Montgomery; Antony Tritz MS; Thad Gurley, MS; Margaret Pendzich

University of Illinois at Chicago-Project WISH, Chicago, IL

Richard M. Novak, MD; Benjamin G. Ladner, MD; Andrea Wendrow, RPh; Jesica Herrick, MD; Alfredo J. Mena Lora, MD; Scott A. Borgetti, MD; Diana L Bahena, APRN; Regina Harden, BA; Renyce Powell; David C. M. Chan, PharmD; Rebeca F. Gasari, PharmD; Michael Pacini, PharmD; Margarita M. Villarreal, CPhT; Rodrigo Reyes, ADN; Samuel M. Rene, MPH; Shannon M Whitted, BSN; Habiba Sultana, MBBS; Nanu Kunwar, BS; Tasmin Sultana, MBBS; Md R. Amin, PhD; Mahmood Ghassemi, PhD, Liam Morrissy, BS; Nia O'Neal, BS; Chasity Serrano, BS; Charlie Peterson, BA

University of Texas Medical Branch, Galveston, TX

Richard Rupp, MD; Laura Porterfield, MD; Amber Stanford, PA-C; Robert Cox, RN; Kristin Pollock, RN; Diane Barrett, MS; Gerrianne Casey, RN; Amy McMahan, LVN; Cori Burkett, PA-C; Essie Cox

Kaiser Permanente Washington Health Research Institute, Seattle, WA

Lisa A. Jackson, MD, MPH; Maya Dunstan, MS, RN; Rebecca Lau, PharmD; Barbara Carste, MPH; Wesley A. Andersen, RPh, MHA, MA; Lee Barr, RN; Cassandra Bryant, BS; Joe Choe, BS; Lynn Gross, PA-C; Erika Kiniry, MPH; Bonnie Y Lam, PharmD; Stella Lee, BA; Paula J Lins, PA-C, MPH; Amy Mohelnitzky, PA-C; Marilyn Nguyen, BS; Matthew Nguyen, MPH; Hallie Phillips, MEd; Melissa Resendiz Rivas, BA; Melissa Boothe Scheer, PA-C; Janice Suyehira, MD; Stacie Wellwood, LPN; Maryann K Woodford, PA-C

Departments of Medicine, Epidemiology, and Laboratory Medicine & Pathology, University of Washington, Vaccines and Infectious Diseases Division, Fred Hutchinson Cancer Center, Seattle, WA

Tara M. Babu, MD, MSCI; Anna Wald, MD, MPH; Taylor Krause, BA; Kirsten Hauge, MPH; Jina Taub, ARNP, Dana Varon, ARNP, Britt Murphy, ARNP, Morissa Pertik, PA-C, T. Nui Pholsena, ARNP, Alyssa Braun, BS, Mark Drummond, BS, Jessica Heimonen, MPH, Amy Link, BS, Lindsey McClellan, BS, Jessica Moreno, BS, Chloe Wilkens, BS, Matt Seymour, MPH, Lawrence Hemingway, BS, Jean Mernaugh, BS, Chris McClurkan, BS, Kerry Laing, PhD, Meredith Potochnic, PharmD, Joong Kim, PharmD, Bao-Chao Vo, PhT, Dil Singh, BS

NYU VTEU Manhattan Research Clinic at NYU Grossman School of Medicine, New York, NY

Angelica C. Kottkamp, MD; Tamia Davis, NP; Celia Engelson, NP; Vijaya Soma, MD; Abdulwahab Abdulai; Ashanay Allen; Natella Aronova, NP; Philip Aziz, PharmD; Emily Beato; Samuel Bliss, PharmD; Jacqueline Callahan, RN; Ellie Carmody, MD; Amanda Dontino, BS; Aimee Edwin, RN; Shelby Goins;

Sarah Haiken; Ramin Herati, MD; Abdonnie Holder; Janice Hong; Trishala Karmacharya; Manpreet Kaur, PharmD; Hye-Youn Kim; Alexander McMeeking, MD; Mark Mulligan, MD; Wai Ng; Edward Nirenberg; Irma Noriega, NP; Samuel Nweke; Lalitha Parameswaran, MD; Levonne Phillip, MPH; Stephanie Rettig, MPH; Marie Samanovic-Golden, PhD; Madalyn Saporito; Pamela Suman; Meron Tasissa; Michael Tuen; Julia Wagner, MPH; James Wilson; Doris Wong, PharmD; Grace Yip, BS; Samantha Yip, RN; Heekoung Youn, RN; Lisa Zhao

Zuckerberg San Francisco General, University of California San Francisco, San Francisco, CA

Anne F. Luetkemeyer, MD; Chloe Harris, BA; Azquena Munoz Lopez, BS; Daniel Berrner; Dennis Dentoni-Lasofsky, MSN; John Dwyer, RN; Suzanne Hendler, BSN; Elvira Gomez, MPH; WeyLing Phuah, PharmD; Jaime Velasco, BA; Veronica Viar, MS

Morehouse School of Medicine, Atlanta, GA

Lilly C. Immergluck, MD; Erica Johnson, PhD; Austin Chan, MD; Fatima Ali, MPH; Sonja Jackson; Noor Mohamed, PharmD; LaKesha Tables, MD; Norberto Fas, MD; Kay Woodson, PharmD; Saadia Khizer, MD; Jacquelyn Ali, MSA; Abdullah Warsama; Eric Gaines; Sierra Jordan Thompson; Cristina Wilson; Trisha Parker, MPH; Xiting Lin; LaTeshia Thomas Seaton, APRN, Derrick Wilson

Washington University School of Medicine, St. Louis, MO

Rachel M. Presti, MD, PhD; Jane A. O'Halloran, MD, PhD; Michael Klebert, RN, PhD Ryley M. Thompson; Alem Haile; Kim Gray, NP; Chapelle Ayres; Delaney Carani, RN; Michael Royal; John Tran; Laura Blair; Anita Afghanzada; Natalie Schodl

NYU VTEU Long Island Research Clinic at NYU Long Island School of Medicine, Mineola, NY

Martín Bäcker, MD, Kimberly Byrnes, RN, Asif Noor, MD, Andrew B. Fleming, MD, Sigridh A. Muñoz-Gómez, MD, Diana Badillo, MD, Steven E. Carsons, MD, Sajumon K. Joseph, FNP, Sarah J. Pastolero,

RN, Sophie Danziger, Monica Benitez, Maung Aung, Louis Ragolia, PhD, Alicia Vasile, RPh, April Correll, RPh, Christopher Hall, Thomas Palaia, Miloni H Thakker, MD, Lavern Harvey, Lisa Zhao

University of Iowa College of Medicine, Iowa City, IA

Patricia L. Winokur, MD; Jeffery Meier, MD; Jack Stapleton, MD; Laura Stulken, PA; Theresa Hegmann, PA; Deb Pfab, RN; Elizabeth Morgan, RN; Susan Herman, RN; Angel Peguero, CMA; Michelle Rodenburg; Alfred J. Carr; Delilah Johnson

Howard University College of Medicine, Howard University Hospital, Washington D.C.

Siham M. Mahgoub, MD; Celia Maxwell, MD; Sarah Shami, PharmD; Edward Bauer, BS; Yuanxiu Chen, MD, PhD; Megan Ware-Pressley, MHA; Debra Ordor, RN; Linda Fletcher, RN; Emmanuel Baidoo, BS; David Jaspan, RPh, MBA; Adetokunbo Adedokun, PharmD, MPH, BCPS; Michelle Strobeck, BS; Michael A. Riga; Ashley Karen Bautista, BS

Department of Medicine, University of Alabama at Birmingham, Birmingham, AL

Paul A. Goepfert, MD; Jenna Weber, RN; Savannah Spaulding, RN; Heather Logan, CRNP; Faye Heard; Foreamben Patel; Michelle Chambers

Tulane University School of Medicine, New Orleans, LA

Dahlene N. Fusco, MD; Arnaud C. Drouin, MD; Florice K. Numbi, MD; Hamada F. Rady, PhD; Crystal A. Ward, MSN; Quinn M. Powers, MS; William E. Casey, BS; Brian P. Logarbo, MD; Shae P. Williams, BS; Emily Callegari, MSN

IDCRC Principal Investigators

David S. Stephens, MD; Kathleen M. Neuzil, MD

IDCRC Leadership Operations Center

Monica M. Farley, MD; Jeanne Marrazzo, MD; Sidnee Paschal Young

IDCRC Clinical Operations Unit

Jeffery Lennox, MD; Robert L. Atmar, MD; Linda McNeil FHI360

IDCRC Laboratory Operations Unit – Fred Hutchinson Cancer Center and University of Washington, Seattle, WA

Christine M. Posavad, PhD; Megan A. Meagher, BS; Michael Stirewalt, MBA; John Hural, PhD; Weston Lawler, BA; Lexi Tanser, MA, Julie McElrath, MD, PhD; Mike Gale, PhD

IDCRC Statistical and Data Science Unit

Elizabeth Brown, PhD

The Emmes Company, LLC, Rockville, MD

Mat Makowski, PhD; Heather Hill, MS; Jim Albert, MS, Holly Baughman; Lisa McQuarrie, MS; Kalyani Telu, MS; Jinjian Mu, PhD

Clinical Monitoring Research Program Directorate, Frederick National Laboratory for Cancer Research, Frederick, MD

Teri C. Lewis, BS; Lisa A. Giebeig, MS; Theresa M. Engel, MFS.; Caleb J. Griffith, MPH; Wendi L. McDonald, BSN; Alissa E. Burkey, MS; Lisa B. Hoopengardner, MS; Jessica E. Linton, MS; Nikki L. Gettinger, MPH; Aroussiak Bowen; Beth R. Baseler, MS; Vanessa S. Eccard-Koons, MS; Charles W. R. Hofsommer, JD; Thomas C. Sova, JD; Gary A. Krauss

Duke Laboratory, NC

David C Montefiori, MD, Amanda Eaton, MBA, Francesca Suman, MS.

Smith's Laboratory, Cambridge, UK

Derek J Smith, PhD; Antonia Netzl; Samuel H Wilks, PhD; Sina Tureli, PhD; Ana Mosterín Höpping, PhD; Samuel Turner; Sarah James, MD; Poppy Roth

Division of Microbiology and Infectious Diseases, National Institute of Allergy and Infectious Diseases, National Institutes of Health, Bethesda, MD.

Marina Lee, PhD; Mamodikoe Makhene, MD; Mohamed Elsafy, MD; Rhonda Pikaart-Tautges, BS; Janice Arega, MS: Binh Hoang, RPh; Dan Curtin; Hyung Koo, BSN; Elisa Sindall, BSN; Aya Nakamura, RN, MS; Audria Crowder, BS; Guinevere Chun, RN, BSN, MSHS; Frank Kenny, PhD MPH; Seemi Patel, RHP, PharmD; Sonia Gales, MS; Ahsen Khan, JD; Walla Dempsey, PhD; Robert Jurao- RN, BSN; Sonja Crandon, BSN; Seema U. Nayak, MD; Marciela M DeGrace, PhD; Diane J Post, PhD; Paul C Roberts, PhD, John H Beigel, MD SAVE Program

Supplemental Methods

<u>Eligibility Criteria for Stages 1-3</u>

Inclusion Criteria

Participants must meet all of the following criteria to be eligible to participate in this study:

1. Individuals > / = 18 years of age at the time of consent.
2. Confirmed receipt of a complete primary and booster COVID-19 vaccine series, either homologous or heterologous, with an FDA authorized/approved vaccine at least 16 weeks prior to study vaccine dose 1.
3. Willing and able to comply with all scheduled visits, vaccination plan, laboratory tests and other study procedures.
4. Determined by medical history, targeted physical examination and clinical judgement of the investigator to be in stable state of health.

Note: Participants with pre-existing stable chronic medical conditions defined as condition not requiring significant change in therapy or hospitalization for worsening disease within 4 weeks from enrollment, can be included at the discretion of the investigator.

Exclusion Criteria:

Participants meeting any of the following criteria will be excluded from the study:

1. Confirmed SARS-CoV-2 infection < 16 weeks prior to any study vaccine dose.
2. Pregnant and breastfeeding participants.
3. Prior administration of an investigational coronavirus vaccine at any time or SARS-CoV-2 immunoglobulin, monoclonal antibody or plasma antibody therapy in the preceding 3 months.

Note: subjects that participated in clinical trials of products that are now FDA approved/authorized are allowed to participate.

4. Current/planned simultaneous participation in another interventional study or receipt of any investigational study product within 28 days prior to vaccine study dose(s).
5. A history of anaphylaxis, urticaria, or other significant adverse reaction requiring medical intervention after receipt of a vaccine, polyethylene glycol (PEG), polysorbate or nanolipid particles.
6. A history of myocarditis or pericarditis at any time prior to enrollment (for subjects in stages 1 and 2).
7. Received or plans to receive a vaccine within 28 days prior to or after any dose of study vaccine.

Note: Receipt of seasonal influenza vaccine is allowed at any time.

8. Bleeding disorder diagnosed by a healthcare provider (e.g., factor deficiency, coagulopathy, or platelet disorder requiring special precautions) or bleeding difficulties with intramuscular injections or blood draws.
9. Current or previous diagnosis of an immunocompromising condition or other immunosuppressive condition.
10. Advanced liver or kidney diseases.
11. Advanced (CD4 count < 200) and/or untreated HIV, untreated Hepatitis B or C.
12. Received oral, intramuscular or intravenous systemic immunosuppressants, or immune-modifying drugs for >14 days in total within 6 months prior to any study vaccine dose (for corticosteroids ≥ 20 mg/day of prednisone equivalent). *Note: Topical medications are allowed.*

13. Received immunoglobulin or blood-derived products, within 3 months prior to any study vaccine dose.
14. Received chemotherapy, immunotherapy or radiation therapy within 6 months prior to any study vaccine dose.
15. Study personnel or an immediate family member or household member of study personnel.
16. Is acutely ill or febrile 72 hours prior to or at vaccine dosing (fever defined as ≥ 38.0 degrees Celsius/100.4 degrees Fahrenheit). Participants meeting this criterion may be rescheduled within the relevant window periods.

Note: Afebrile participants with minor illnesses can be enrolled at the discretion of the Investigator, as long as the illness is not suggestive of COVID-19.

TABLE S1: List of COVAIL US Sites

George Washington Vaccine Research Unit, George Washington University

University of Rochester Medical Center

Hope Clinic of the Emory Vaccine Center

Brigham and Women's Hospital, Harvard Medical School

Center for Vaccine Development, Saint Louis University

Baylor College of Medicine

University of California San Diego

Emory Center for Childhood Infections and Vaccines of Children's Healthcare of Atlanta

Duke Human Vaccine Institute, Duke University School of Medicine

University of Illinois at Chicago, Project WISH

University of Texas Medical Branch

Kaiser Permanente Washington Health Research Institute

University of Washington

New York University Manhattan Research Clinic, NYU Grossman School of Medicine

Zuckerberg San Francisco General Hospital, University of California at San Francisco

Morehouse School of Medicine

Washington University School of Medicine

NYU Long Island Research Clinic, NYU Long Island School of Medicine

University of Iowa College of Medicine

Howard University Hospital, Howard University Hospital

University of Alabama at Birmingham

Tulane University School of Medicine

TABLE S2: Eligible Regimens for Primary Vaccination Series and Boost as of June 2022

Primary series vaccine manufacturer	Number of doses in primary series	Primary series dose	Interval between 1st and 2nd dose	Number of booster doses	Booster dose	Allowed interval between primary series and booster dose*
Pfizer BioNTech	2	30 µg	At least 17 days	1	30 µg	≥ 5 months
Moderna	2	100 µg	At least 24 days	1	50 µg	≥ 5 months
Janssen	1	$5{\times}10^{10}$ viral particles	Not applicable	1	$5{\times}10^{10}$ viral particles	≥ 2 months

*homologous and heterologous boosters are acceptable

Table S3: Breakthrough Infections During the Study by Stage and Arm through cut-off Date of December 2, 2022

Stage	Treatment Arm	At Risk (Day 1)	Breakthrough Infection Count	Breakthrough Infection Rate	95% CI
**Moderna**	1 Dose Prototype	99	31	32.3%	(23.9%, 42.7%)
	1 Dose Beta+Omicron	112	45	41.4%	(32.7%, 51.3%)
	1 Dose Delta+Omicron	101	33	33.1%	(24.8%, 43.4%)
	1 Dose Omicron	99	29	30.4%	(22.2%, 40.8%)
	1 Dose Omicron+Prototype	99	34	42.6%	(27.6%, 61.4%)
**Pfizer**	Wildtype (Prototype)	50	17	36.4%	(24.3%, 52.1%)
	Beta+Omicron	52	7	13.6%	(6.7%, 26.4%)
	Omicron	54	10	19.1%	(10.7%, 32.6%)
	Beta	51	11	22.2%	(12.9%, 36.5%)
	Beta+Wildtype (Prototype)	52	12	23.8%	(14.2%, 38.1%)
	Omicron+Wildtype (Prototype)	53	10	18.9%	(10.6%, 32.2%)
**Sanofi**	Prototype	49	6	12.3%	(5.7%, 25.3%)
	Beta	50	13	26.4%	(16.2%, 41.1%)
	Beta+Prototype	52	9	17.4%	(9.5%, 30.8%)

Table S4: Breakthrough Infections in the Study by Stage and Prior Infection Status at Enrollment

Stage	Baseline Infection Status	At Risk (Day 1)	Breakthrough Infection Count	Breakthrough Infection Rate	95% CI
**Moderna**	No	406	155	41.9%	(35.4%,49.1%)
	Yes	104	17	16.7%	(10.7%,25.5%)
**Pfizer**	No	208	57	28.4%	(22.7%,35.3%)
	Yes	104	10	9.7%	(5.4%,17.3%)
**Sanofi**	No	90	25	28.1%	(19.9%,38.7%)
	Yes	61	3	5.0%	(1.6%,14.6%)
**Overall**	No	704	237	37.8%	(31.8%,44.6%)
	Yes	269	30	12.1%	(8.4%,17.2%)

Table S5: Breakthrough Infections During the Study by Stage and Age at Enrollment

Stage	Age Group	At Risk (Day 1)	Breakthrough Infection Count	Breakthrough Infection Rate	95% CI
**Moderna**	≥ 65 years	178	39	23.1%	(17.4%, 30.3%)
	18-64 years	332	133	43.6%	(36.2%, 51.9%)
**Pfizer**	≥ 65 years	94	11	11.8%	(6.7%, 20.3%)
	18-64 years	218	56	26.6%	(21.1%, 33.2%)
**Sanofi**	≥ 65 years	30	5	16.8%	(7.4%, 35.8%)
	18-64 years	121	23	19.2%	(13.2%, 27.4%)
**Overall**	≥ 65 years	302	55	19.3%	(15.1%,24.5%)
	18-64 years	671	212	36.2%	(29.2%,44.4%)

Table S6: Stage 1 (Moderna) Pseudovirus Neutralization Assay Summary Results by Arm, Time Point and Variant for Uninfected Participants

Time Point	Statistic	D614G	BA.1	BA.2.12.1	BA.4/5	B.1.351	B.1.617.2
**Arm 1: 1 Dose Prototype**							
Day 1	n	78	78	22	78	78	78
	GMT (95% CI)	2228 (1680, 2956)	164 (115, 232)	129 (73, 226)	94 (67, 133)	519 (371, 725)	1095 (799, 1502)
	Median (Min, Max)	2161 (139,33802)	195 (20,3809)	154 (20,1130)	124 (20,3079)	642 (20,9119)	1140 (20,14380)
	GMR $_{D614G}$ (95% CI)	1.0 (NE)	13.6 (11.1, 16.7)	17.9 (12.6, 25.4)	23.6 (18.9, 29.6)	4.3 (3.6, 5.2)	2.0 (1.8, 2.3)
	Seropositive (95% CI)	1.00 (0.95, 1.00)	0.74 (0.63, 0.84)	0.77 (0.55, 0.92)	0.56 (0.45, 0.68)	0.92 (0.84, 0.97)	0.97 (0.91, 1.00)
Day 15	n	75	75	22	22	75	75
	GMT (95% CI)	16976 (13851, 20806)	1997 (1482, 2692)	1588 (1119, 2253)	1007 (676, 1501)	5982 (4632, 7724)	9327 (7517, 11574)
	Median (Min, Max)	17534 (2205,68899)	2103 (20,28781)	1559 (323,8880)	996 (202,6398)	5942 (239,65076)	9038 (1091,66807)
	GMR $_{D614G}$ (95% CI)	1.0 (NE)	8.5 (6.9, 10.5)	9.8 (7.6, 12.7)	15.5 (12.3, 19.4)	2.8 (2.5, 3.2)	1.8 (1.7, 1.9)
	GMFR (95% CI)	7.4 (6.1, 9.0)	11.9 (9.0, 15.9)	12.3 (8.4, 18.0)	13.2 (9.3, 18.9)	11.1 (8.8, 14.1)	8.3 (6.6, 10.4)
	Seropositive (95% CI)	1.00 (0.95, 1.00)	0.97 (0.91, 1.00)	1.00 (0.85, 1.00)	1.00 (0.85, 1.00)	1.00 (0.95, 1.00)	1.00 (0.95, 1.00)
Day 29	n	73	73	73	73	73	73
	GMT (95% CI)	12600 (10243, 15500)	1343 (1037, 1739)	ND	722 (509, 1024)	3535 (2664, 4691)	6181 (4933, 7745)
	Median (Min, Max)	13098 (1632,77641)	1354 (20,15656)	ND	943 (20,8496)	3731 (20,33994)	6057 (644,42604)
	GMR $_{D614G}$ (95% CI)	1.0 (NE)	9.4 (7.8, 11.3)	ND	17.4 (13.6, 22.4)	3.6 (2.9, 4.4)	2.0 (1.9, 2.2)
	GMFR (95% CI)	5.5 (4.5, 6.7)	8.2 (6.3, 10.6)	ND	7.7 (5.8, 10.2)	6.6 (5.3, 8.2)	5.5 (4.4, 6.8)
	Seropositive (95% CI)	1.00 (0.95, 1.00)	0.99 (0.93, 1.00)	ND	0.92 (0.83, 0.97)	0.99 (0.93, 1.00)	1.00 (0.95, 1.00)
Day 91	n	58	58	58	58	58	58

	GMT (95% CI)	7020 (5034, 9791)	583 (437, 778)	ND	341 (234, 496)	1871 (1346, 2601)	3090 (2271, 4204)
	Median (Min, Max)	7961 (20,47117)	620 (20,3960)	ND	348 (20,3849)	1999 (20,17591)	3342 (20,23974)
	GMR D614G (95% CI)	1.0 (NE)	12.0 (9.9, 14.6)	ND	20.6 (16.0, 26.5)	3.8 (3.2, 4.4)	2.3 (2.1, 2.5)
	GMFR (95% CI)	2.7 (2.0, 3.7)	3.4 (2.6, 4.4)	ND	3.3 (2.5, 4.3)	3.4 (2.7, 4.3)	2.4 (1.8, 3.2)
	GMFD (95% CI)	1.9 (1.4, 2.4)	2.3 (2.0, 2.6)	ND	2.1 (1.7, 2.6)	1.8 (1.6, 2.1)	2.1 (1.6, 2.7)
	Seropositive (95% CI)	0.98 (0.91, 1.00)	0.97 (0.88, 1.00)	ND	0.88 (0.77, 0.95)	0.98 (0.91, 1.00)	0.98 (0.91, 1.00)
**Arm 2 and Arm 3: 1 and 2 Dose Beta (B.1.351)+Omicron (BA1)[a]**							
Day 1	n	160	160	22	160	160	160
	GMT (95% CI)	2872 (2352, 3507)	199 (155, 255)	154 (81, 294)	105 (82, 135)	694 (555, 867)	1354 (1101, 1667)
	Median (Min, Max)	2949 (20,57670)	237 (20,8791)	180 (20,1752)	123 (20,6241)	720 (20,34082)	1465 (20,40606)
	GMR D614G (95% CI)	1.0 (NE)	14.5 (12.3, 17.0)	22.4 (15.3, 32.9)	27.4 (22.8, 32.9)	4.1 (3.7, 4.6)	2.1 (2.0, 2.3)
	Seropositive (95% CI)	0.99 (0.97, 1.00)	0.77 (0.70, 0.83)	0.73 (0.50, 0.89)	0.58 (0.50, 0.66)	0.96 (0.91, 0.98)	0.98 (0.94, 0.99)
Day 15	n	157	157	22	22	157	157
	GMT (95% CI)	16115 (13445, 19316)	3742 (3020, 4635)	1539 (844, 2804)	1106 (604, 2025)	9591 (7897, 11648)	9824 (8202, 11767)
	Median (Min, Max)	15900 (20,330760)	3833 (20,126625)	1410 (20,11939)	1336 (20,9977)	10328 (20,160941)	10248 (20,148989)
	GMR D614G (95% CI)	1.0 (NE)	4.3 (3.9, 4.8)	7.7 (5.0, 11.7)	10.7 (7.1, 16.2)	1.7 (1.6, 1.8)	1.6 (1.6, 1.7)
	GMFR (95% CI)	5.6 (4.8, 6.5)	18.9 (15.4, 23.1)	10.0 (5.7, 17.3)	11.8 (7.6, 18.5)	13.8 (11.8, 16.1)	7.3 (6.2, 8.5)
	Seropositive (95% CI)	0.99 (0.97, 1.00)	0.99 (0.95, 1.00)	0.95 (0.77, 1.00)	0.95 (0.77, 1.00)	0.99 (0.97, 1.00)	0.99 (0.97, 1.00)
Day 29	n	154	154	154	154	154	154
	GMT (95% CI)	12277 (10341, 14577)	2827 (2295, 3481)	ND	1240 (999, 1538)	7016 (5757, 8549)	7076 (5946, 8421)
	Median (Min, Max)	11568 (20,142897)	2876 (20,61692)	ND	1388 (20,26413)	6572 (20,130621)	6669 (20,66743)
	GMR D614G (95% CI)	1.0 (NE)	4.3 (3.9, 4.8)	ND	9.9 (8.7, 11.3)	1.7 (1.6, 1.9)	1.7 (1.7, 1.8)

	GMFR (95% CI)	4.3 (3.7, 4.9)	14.2 (11.7, 17.2)	ND	11.7 (9.5, 14.4)	10.1 (8.7, 11.8)	5.2 (4.5, 6.1)
	Seropositive (95% CI)	0.99 (0.96, 1.00)	0.98 (0.94, 1.00)	ND	0.96 (0.92, 0.99)	0.99 (0.96, 1.00)	0.99 (0.96, 1.00)
Day 91	n	62	62	62	62	62	62
	GMT (95% CI)	8449 (6636, 10757)	1206 (884, 1644)	ND	629 (458, 864)	3820 (2794, 5222)	4611 (3587, 5927)
	Median (Min, Max)	7084 (1235,59187)	1368 (20,18784)	ND	823 (20,7653)	3996 (148,27580)	4263 (571,36607)
	GMR $_{D614G}$ (95% CI)	1.0 (NE)	7.0 (5.7, 8.6)	ND	13.4 (10.6, 17.0)	2.2 (1.9, 2.6)	1.8 (1.7, 2.0)
	GMFR (95% CI)	3.3 (2.7, 4.1)	6.7 (5.1, 8.8)	ND	6.9 (5.1, 9.3)	5.8 (4.7, 7.3)	3.7 (3.0, 4.4)
	GMFD (95% CI)	1.4 (1.2, 1.6)	2.2 (1.9, 2.5)	ND	1.8 (1.5, 2.2)	1.7 (1.5, 2.0)	1.5 (1.3, 1.6)
	Seropositive (95% CI)	1.00 (0.94, 1.00)	0.98 (0.91, 1.00)	ND	0.95 (0.87, 0.99)	1.00 (0.94, 1.00)	1.00 (0.94, 1.00)
**Arm 4: 1 Dose Delta (B.1.617.2)+Omicron (BA1)**							
Day 1	n	84	84	24	84	84	84
	GMT (95% CI)	2812 (2030, 3894)	213 (147, 310)	192 (97, 381)	108 (77, 153)	670 (462, 971)	1403 (1008, 1954)
	Median (Min, Max)	3730 (20,61227)	272 (20,7767)	211 (20,9305)	142 (20,4312)	790 (20,28796)	1778 (20,38544)
	GMR $_{D614G}$ (95% CI)	1.0 (NE)	13.2 (10.5, 16.5)	18.8 (10.7, 32.9)	26.0 (19.9, 33.9)	4.2 (3.5, 5.1)	2.0 (1.9, 2.2)
	Seropositive (95% CI)	0.96 (0.90, 0.99)	0.74 (0.63, 0.83)	0.79 (0.58, 0.93)	0.58 (0.47, 0.69)	0.92 (0.84, 0.97)	0.96 (0.90, 0.99)
Day 15	n	83	83	24	24	83	83
	GMT (95% CI)	21972 (16761, 28802)	3808 (2716, 5337)	2494 (1455, 4275)	1584 (932, 2693)	8608 (6196, 11959)	12773 (9703, 16813)
	Median (Min, Max)	28136 (20,272880)	4338 (20,106363)	2204 (409,72253)	1475 (259,48395)	10877 (20,163492)	15205 (20,229105)
	GMR $_{D614G}$ (95% CI)	1.0 (NE)	5.8 (4.7, 7.1)	8.3 (6.0, 11.5)	13.1 (9.4, 18.1)	2.6 (2.2, 2.9)	1.7 (1.6, 1.8)
	GMFR (95% CI)	7.9 (5.8, 10.7)	18.3 (13.1, 25.6)	13.0 (6.3, 26.6)	17.8 (8.3, 38.1)	13.1 (9.5, 18.0)	9.3 (6.8, 12.7)
	Seropositive (95% CI)	0.99 (0.93, 1.00)	0.98 (0.92, 1.00)	1.00 (0.86, 1.00)	1.00 (0.86, 1.00)	0.99 (0.93, 1.00)	0.99 (0.93, 1.00)
Day 29	n	79	79	79	79	79	79
	GMT (95% CI)	16001 (12150, 21073)	2684 (1899, 3794)	ND	1384 (956, 2004)	5783 (4084, 8188)	9342 (7077, 12332)
	Median (Min, Max)	19156 (20,402704)	3091 (20,173396)	ND	1496 (20,51474)	7995 (20,206284)	10918 (20,252540)

	GMR $_{D614G}$ (95% CI)	1.0 (NE)	6.0 (4.8, 7.4)	ND	11.6 (9.1, 14.7)	2.8 (2.4, 3.2)	1.7 (1.6, 1.8)
	GMFR (95% CI)	5.7 (4.1, 7.9)	12.2 (8.6, 17.1)	ND	12.7 (9.3, 17.4)	8.3 (5.9, 11.5)	6.7 (4.8, 9.2)
	Seropositive (95% CI)	0.99 (0.93, 1.00)	0.97 (0.91, 1.00)	ND	0.95 (0.88, 0.99)	0.99 (0.93, 1.00)	0.99 (0.93, 1.00)
Day 91	n	62	62	62	62	62	62
	GMT (95% CI)	10973 (7582, 15881)	1258 (821, 1927)	ND	680 (438, 1057)	3328 (2102, 5269)	5348 (3691, 7750)
	Median (Min, Max)	14069 (20,242000)	1356 (20,51127)	ND	833 (20,15236)	3778 (20,104299)	5806 (20,72666)
	GMR $_{D614G}$ (95% CI)	1.0 (NE)	8.7 (6.7, 11.4)	ND	16.1 (12.2, 21.3)	3.3 (2.7, 4.0)	2.1 (1.9, 2.2)
	GMFR (95% CI)	3.7 (2.8, 5.0)	5.6 (4.1, 7.7)	ND	5.4 (4.0, 7.4)	4.6 (3.3, 6.4)	3.6 (2.7, 5.0)
	GMFD (95% CI)	1.4 (1.3, 1.6)	2.0 (1.8, 2.3)	ND	2.0 (1.8, 2.3)	1.7 (1.5, 2.0)	1.7 (1.5, 2.0)
	Seropositive (95% CI)	0.98 (0.91, 1.00)	0.95 (0.87, 0.99)	ND	0.90 (0.80, 0.96)	0.97 (0.89, 1.00)	0.98 (0.91, 1.00)
**Arm 5: 1 Dose Omicron (BA1)**							
Day 1	n	76	76	19	76	76	76
	GMT (95% CI)	2853 (2087, 3900)	206 (140, 304)	233 (125, 436)	118 (81, 172)	583 (392, 868)	1382 (1011, 1890)
	Median (Min, Max)	3016 (20,69373)	234 (20,7754)	222 (20,2075)	148 (20,4429)	737 (20,17683)	1542 (20,38445)
	GMR $_{D614G}$ (95% CI)	1.0 (NE)	13.8 (11.3, 17.0)	18.5 (13.3, 25.8)	24.2 (19.3, 30.3)	4.9 (4.0, 6.0)	2.1 (1.9, 2.2)
	Seropositive (95% CI)	0.99 (0.93, 1.00)	0.75 (0.64, 0.84)	0.89 (0.67, 0.99)	0.59 (0.47, 0.70)	0.91 (0.82, 0.96)	0.99 (0.93, 1.00)
Day 15	n	73	73	18	18	73	73
	GMT (95% CI)	16467 (13087, 20719)	4561 (3379, 6157)	3032 (1693, 5429)	2036 (1137, 3647)	8642 (6240, 11968)	9490 (7490, 12023)
	Median (Min, Max)	17732 (1552,389237)	5552 (20,86464)	2713 (309,55314)	1747 (298,50989)	10519 (20,390573)	10466 (1340,240645)
	GMR $_{D614G}$ (95% CI)	1.0 (NE)	3.6 (3.0, 4.4)	5.6 (4.2, 7.4)	8.3 (6.7, 10.4)	1.9 (1.6, 2.3)	1.7 (1.6, 1.9)
	GMFR (95% CI)	5.7 (4.5, 7.4)	21.7 (16.2, 29.0)	12.6 (7.4, 21.3)	12.7 (7.4, 22.0)	14.6 (11.1, 19.3)	6.7 (5.2, 8.7)
	Seropositive (95% CI)	1.00 (0.95, 1.00)	0.99 (0.93, 1.00)	1.00 (0.81, 1.00)	1.00 (0.81, 1.00)	0.99 (0.93, 1.00)	1.00 (0.95, 1.00)
Day 29	n	72	72	72	72	72	72
	GMT (95% CI)	11963 (9464, 15122)	3005 (2211, 4085)	ND	1190 (853, 1661)	5792 (4161, 8063)	6902 (5439, 8758)

	Median (Min, Max)	13539 (1027,281094)	3278 (20,78111)	ND	1631 (20,61002)	6510 (20,259673)	6692 (559,210702)
	GMR D614G (95% CI)	1.0 (NE)	4.0 (3.2, 4.9)	ND	10.1 (8.0, 12.6)	2.1 (1.6, 2.6)	1.7 (1.6, 1.9)
	GMFR (95% CI)	4.3 (3.3, 5.5)	14.6 (11.2, 19.1)	ND	10.6 (7.9, 14.1)	10.1 (7.9, 12.9)	5.0 (3.9, 6.5)
	Seropositive (95% CI)	1.00 (0.95, 1.00)	0.99 (0.93, 1.00)	ND	0.96 (0.88, 0.99)	0.99 (0.93, 1.00)	1.00 (0.95, 1.00)
Day 91	n	60	60	60	60	60	60
	GMT (95% CI)	9941 (7399, 13357)	1351 (907, 2013)	ND	689 (450, 1052)	4082 (2755, 6048)	4842 (3386, 6923)
	Median (Min, Max)	8713 (584,161949)	1699 (20,26323)	ND	983 (20,22203)	4742 (20,123017)	5160 (20,169340)
	GMR D614G (95% CI)	1.0 (NE)	7.4 (5.6, 9.7)	ND	14.4 (10.9, 19.0)	2.4 (1.9, 3.1)	2.1 (1.7, 2.6)
	GMFR (95% CI)	3.5 (2.6, 4.7)	6.6 (4.9, 9.0)	ND	5.8 (4.2, 8.0)	7.0 (5.2, 9.3)	3.5 (2.8, 4.5)
	GMFD (95% CI)	1.3 (1.1, 1.5)	2.2 (1.9, 2.6)	ND	1.8 (1.6, 2.1)	1.4 (1.2, 1.7)	1.5 (1.2, 2.0)
	Seropositive (95% CI)	1.00 (0.94, 1.00)	0.95 (0.86, 0.99)	ND	0.88 (0.77, 0.95)	0.98 (0.91, 1.00)	0.98 (0.91, 1.00)
**Arm 6: 1 Dose Omicron (BA1)+Prototype**							
Day 1	n	79	79	23	79	79	79
	GMT (95% CI)	2586 (1897, 3525)	225 (161, 314)	264 (158, 440)	103 (73, 146)	640 (466, 881)	1289 (936, 1775)
	Median (Min, Max)	3222 (20,41622)	256 (20,18442)	270 (20,2120)	134 (20,10419)	690 (20,36239)	1490 (20,37699)
	GMR D614G (95% CI)	1.0 (NE)	11.5 (9.2, 14.4)	15.0 (10.1, 22.3)	25.1 (19.2, 32.8)	4.0 (3.5, 4.7)	2.0 (1.8, 2.2)
	Seropositive (95% CI)	0.97 (0.91, 1.00)	0.82 (0.72, 0.90)	0.91 (0.72, 0.99)	0.59 (0.48, 0.70)	0.94 (0.86, 0.98)	0.96 (0.89, 0.99)
Day 15	n	73	73	23	23	73	73
	GMT (95% CI)	21096 (17398, 25580)	3747 (2773, 5061)	2519 (1708, 3715)	1576 (933, 2663)	7814 (5879, 10386)	10798 (8331, 13995)
	Median (Min, Max)	23575 (2771,168564)	3471 (20,82878)	2642 (89,13552)	1781 (20,10239)	8501 (20,101435)	12003 (20,85750)
	GMR D614G (95% CI)	1.0 (NE)	5.6 (4.6, 6.9)	7.2 (5.5, 9.4)	11.5 (7.9, 16.7)	2.7 (2.2, 3.3)	2.0 (1.6, 2.4)
	GMFR (95% CI)	7.8 (6.0, 10.2)	15.8 (12.1, 20.8)	9.6 (6.0, 15.2)	14.3 (7.6, 26.9)	11.7 (9.1, 15.1)	8.1 (6.2, 10.5)
	Seropositive (95% CI)	1.00 (0.95, 1.00)	0.99 (0.93, 1.00)	1.00 (0.85, 1.00)	0.96 (0.78, 1.00)	0.99 (0.93, 1.00)	0.99 (0.93, 1.00)
Day 29	n	70	70	70	70	70	70

	GMT (95% CI)	15635<br>(12686, 19269)	2928<br>(2166, 3957)	ND	1322<br>(984, 1776)	5744<br>(4404, 7493)	8175<br>(6513, 10261)
	Median<br>(Min, Max)	15131<br>(1648,178407)	3125<br>(20,69319)	ND	1387<br>(20,33137)	5335<br>(516,160251)	7450<br>(848,127131)
	GMR $_{D614G}$<br>(95% CI)	1.0<br>(NE)	5.3<br>(4.5, 6.3)	ND	11.8<br>(9.8, 14.3)	2.7 (2.4, 3.1)	1.9 (1.8, 2.1)
	GMFR (95% CI)	5.1<br>(4.2, 6.3)	11.6<br>(9.1, 14.9)	ND	11.6<br>(8.5, 15.8)	7.7 (6.2, 9.6)	5.4 (4.3, 6.6)
	Seropositive (95% CI)	1.00 (0.95, 1.00)	0.99 (0.92, 1.00)	ND	0.97 (0.90, 1.00)	1.00 (0.95, 1.00)	1.00 (0.95, 1.00)
Day 91	n	58	58	58	58	58	58
	GMT (95% CI)	11775<br>(9106, 15227)	1652<br>(1142, 2390)	ND	818<br>(588, 1139)	4050<br>(2848, 5758)	6143<br>(4653, 8110)
	Median<br>(Min, Max)	11323<br>(966,115427)	1579<br>(20,43028)	ND	816<br>(20,25058)	4219<br>(20,76354)	5534<br>(472,78091)
	GMR $_{D614G}$<br>(95% CI)	1.0<br>(NE)	7.1<br>(5.7, 9.0)	ND	14.4<br>(11.7, 17.7)	2.9<br>(2.4, 3.5)	1.9<br>(1.8, 2.1)
	GMFR (95% CI)	3.4<br>(2.8, 4.1)	5.9<br>(4.5, 7.8)	ND	6.5<br>(4.8, 9.0)	4.7<br>(3.8, 5.9)	3.5<br>(2.8, 4.4)
	GMFD (95% CI)	1.4 (1.2, 1.7)	2.0 (1.7, 2.3)	ND	1.9 (1.6, 2.1)	1.6 (1.3, 1.9)	1.4 (1.2, 1.7)
	Seropositive (95% CI)	1.00 (0.94, 1.00)	0.97 (0.88, 1.00)	ND	0.97 (0.88, 1.00)	0.98 (0.91, 1.00)	1.00 (0.94, 1.00)

n=Number of subjects with results available at time point. ND= not done. Confidence intervals of the geometric means were calculated with the Student's t distribution on log-transformed data. [a]Arm 2 and 3 combined for all timepoints prior to subjects in arm 2 received second dose and excluded thereafter.

Table S7: Stage 1 (Moderna) Pseudovirus Neutralization Assay Summary Results by Arm, Time Point and Variant for Previously Infected Participants

Time Point	Statistic	D614G	BA.1	BA.2.12.1	BA.4/5	B.1.351	B.1.617.2
**Arm 1: 1 Dose Prototype**							
Day 1	n	21	21	3	21	21	21
	GMT (95% CI)	13117 (7565, 22745)	1573 (812, 3045)	1555 (39, 61761)	990 (511, 1920)	4262 (2117, 8584)	6586 (3661, 11848)
	Median (Min, Max)	14669 (887,108216)	1557 (97,27671)	3159 (283,4203)	1079 (20,12119)	5555 (166,43346)	6208 (446,71062)
	GMR $_{D614G}$ (95% CI)	1.0 (NE)	8.3 (5.6, 12.4)	4.7 (0.4, 59.7)	13.2 (9.3, 18.8)	3.1 (2.1, 4.5)	2.0 (1.6, 2.5)
	Seropositive (95% CI)	1.00 (0.84, 1.00)	1.00 (0.84, 1.00)	1.00 (0.29, 1.00)	0.95 (0.76, 1.00)	1.00 (0.84, 1.00)	1.00 (0.84, 1.00)
Day 15	n	21	21	3	3	21	21
	GMT (95% CI)	44587 (30177, 65877)	7906 (4584, 13638)	6496 (66, 635796)	3560 (81, 157042)	18863 (11081, 32110)	25152 (16488, 38369)
	Median (Min, Max)	52942 (13305,373292)	7566 (1190,78414)	7779 (944,37321)	5351 (659,12793)	24343 (1405,207240)	28815 (3972,173543)
	GMR $_{D614G}$ (95% CI)	1.0 (NE)	5.6 (3.9, 8.2)	4.9 (0.4, 57.0)	8.9 (1.4, 56.3)	2.4 (1.8, 3.1)	1.8 (1.6, 2.0)
	GMFR (95% CI)	3.4 (2.4, 4.9)	5.0 (3.4, 7.5)	4.2 (0.8, 22.1)	4.9 (1.1, 22.1)	4.4 (3.0, 6.4)	3.8 (2.7, 5.5)
	Seropositive (95% CI)	1.00 (0.84, 1.00)	1.00 (0.84, 1.00)	1.00 (0.29, 1.00)	1.00 (0.29, 1.00)	1.00 (0.84, 1.00)	1.00 (0.84, 1.00)
Day 29	n	21	21	21	21	21	21
	GMT (95% CI)	31104 (19694, 49125)	4486 (2566, 7844)	ND	2997 (1679, 5348)	10588 (5964, 18799)	15165 (9397, 24471)
	Median (Min, Max)	34689 (5523,184152)	4445 (629,34612)	ND	3354 (322,71430)	11409 (615,83849)	19420 (2148,71188)
	GMR $_{D614G}$ (95% CI)	1.0 (NE)	6.9 (4.9, 9.9)	ND	10.4 (7.1, 15.2)	2.9 (2.1, 4.1)	2.1 (1.7, 2.5)
	GMFR (95% CI)	2.4 (1.7, 3.2)	2.9 (2.1, 3.8)	ND	3.0 (2.0, 4.5)	2.5 (1.8, 3.4)	2.3 (1.7, 3.1)
	Seropositive (95% CI)	1.00 (0.84, 1.00)	1.00 (0.84, 1.00)	ND	1.00 (0.84, 1.00)	1.00 (0.84, 1.00)	1.00 (0.84, 1.00)
Day 91	n	19	19	19	19	19	19
	GMT (95% CI)	25356 (14478, 44406)	2488 (1285, 4816)	ND	2015 (1014, 4007)	7256 (3536, 14888)	10795 (5854, 19909)
	Median (Min, Max)	20712 (4877,420375)	1878 (426,69848)	ND	1677 (111,49088)	5220 (423,224762)	10798 (1310,274071)
	GMR $_{D614G}$ (95% CI)	1.0 (NE)	10.2 (7.0, 14.8)	ND	12.6 (9.6, 16.5)	3.5 (2.6, 4.7)	2.3 (2.0, 2.8)
	GMFR	2.2 (1.5, 3.2)	1.6 (1.2, 2.3)	ND	2.2 (1.4, 3.3)	2.0 (1.4, 3.0)	1.9 (1.4, 2.6)

	(95% CI)						
	GMFD (95% CI)	1.2 (0.9, 1.5)	1.8 (1.4, 2.3)	ND	1.5 (1.1, 2.0)	1.4 (1.0, 1.8)	1.3 (1.0, 1.8)
	Seropositive (95% CI)	1.00 (0.82, 1.00)	1.00 (0.82, 1.00)	ND	1.00 (0.82, 1.00)	1.00 (0.82, 1.00)	1.00 (0.82, 1.00)
**Arm 2 and Arm 3: 1 and 2 Dose Beta (B.1.351)+Omicron (BA1)[a]**							
Day 1	n	38	38	3	38	38	38
	GMT (95% CI)	10923 (6562, 18183)	1348 (720, 2524)	1921 (54, 68319)	791 (447, 1401)	4327 (2464, 7597)	4819 (2565, 9053)
	Median (Min , Max)	13847 (20,121332)	2112 (20,15942)	1006 (706,9976)	1278 (20,14592)	3957 (20,41317)	7087 (20,69083)
	GMR $_{D614G}$ (95% CI)	1.0 (NE)	8.1 (5.6, 11.8)	25.5 (2.5, 262.3)	13.8 (9.3, 20.4)	2.5 (1.5, 4.3)	2.3 (1.6, 3.2)
	Seropositive (95% CI)	0.97 (0.86, 1.00)	0.89 (0.75, 0.97)	1.00 (0.29, 1.00)	0.87 (0.72, 0.96)	0.95 (0.82, 0.99)	0.92 (0.79, 0.98)
Day 15	n	38	38	3	3	38	38
	GMT (95% CI)	46135 (33789, 62993)	13558 (7262, 25314)	11218 (4109, 30624)	5825 (3651, 9292)	35903 (25629, 50295)	25847 (15711, 42524)
	Median (Min , Max)	51178 (1800,208690)	20339 (20,94116)	11953 (7281,16221)	5604 (4935,7145)	43917 (789,177469)	35166 (20,148746)
	GMR $_{D614G}$ (95% CI)	1.0 (NE)	3.4 (2.0, 5.8)	6.2 (1.0, 38.5)	12.0 (3.0, 47.9)	1.3 (1.2, 1.4)	1.8 (1.2, 2.6)
	GMFR (95% CI)	4.2 (2.8, 6.4)	10.1 (5.6, 18.1)	5.8 (0.1, 512.6)	3.1 (0.2, 47.8)	8.3 (5.3, 12.9)	5.4 (3.5, 8.3)
	Seropositive (95% CI)	1.00 (0.91, 1.00)	0.95 (0.82, 0.99)	1.00 (0.29, 1.00)	1.00 (0.29, 1.00)	1.00 (0.91, 1.00)	0.97 (0.86, 1.00)
Day 29	n	37	37	37	37	37	37
	GMT (95% CI)	31745 (19361, 52049)	10055 (5455, 18536)	ND	5074 (2830, 9097)	22727 (14017, 36850)	19793 (12233, 32027)
	Median (Min , Max)	37579 (20,164753)	13045 (20,72318)	ND	6604 (20,53017)	27785 (20,136949)	26265 (20,134673)
	GMR $_{D614G}$ (95% CI)	1.0 (NE)	3.2 (2.1, 4.8)	ND	6.3 (4.1, 9.5)	1.4 (1.2, 1.6)	1.6 (1.4, 1.8)
	GMFR (95% CI)	2.7 (1.4, 5.0)	6.7 (3.6, 12.1)	ND	5.8 (3.4, 10.1)	4.5 (3.3, 6.2)	3.5 (2.2, 5.7)
	Seropositive (95% CI)	0.97 (0.86, 1.00)	0.95 (0.82, 0.99)	ND	0.95 (0.82, 0.99)	0.97 (0.86, 1.00)	0.97 (0.86, 1.00)
Day 91	n	19	19	19	19	19	19

	GMT (95% CI)	33376 (24176, 46076)	9161 (6654, 12613)	ND	5401 (3530, 8263)	20590 (13873, 30557)	20257 (13924, 29470)
	Median (Min , Max)	29024 (11116,107592)	7642 (3002,41232)	ND	4926 (1366,37440)	24009 (3759,111469)	21965 (5486,71835)
	GMR $_{D614G}$ (95% CI)	1.0 (NE)	3.6 (2.8, 4.8)	ND	6.2 (4.8, 8.0)	1.6 (1.3, 2.0)	1.6 (1.5, 1.9)
	GMFR (95% CI)	1.8 (1.2, 2.6)	3.4 (2.0, 5.9)	ND	3.5 (2.5, 4.8)	3.1 (1.8, 5.1)	2.0 (1.4, 3.0)
	GMFD (95% CI)	1.3 (1.0, 1.8)	1.4 (0.6, 3.3)	ND	1.2 (0.6, 2.7)	1.5 (1.2, 1.9)	1.5 (1.2, 1.8)
	Seropositive (95% CI)	1.00 (0.82, 1.00)	1.00 (0.82, 1.00)	ND	1.00 (0.82, 1.00)	1.00 (0.82, 1.00)	1.00 (0.82, 1.00)
**Arm 4: 1 Dose Delta (B.1.617.2)+Omicron (BA1)**							
Day 1	n	17	17	1	17	17	17
	GMT (95% CI)	10999 (6446, 18767)	1032 (425, 2506)	4099 (NE)	813 (400, 1652)	3013 (1409, 6443)	5831 (3539, 9610)
	Median (Min, Max)	14171 (2144,47968)	996 (20,10985)	4099 (4099,4099)	1217 (20,6358)	2860 (185,25082)	6985 (802,31905)
	GMR $_{D614G}$ (95% CI)	1.0 (NE)	10.7 (6.1, 18.6)	9.9 (NE)	13.5 (9.3, 19.7)	3.7 (2.6, 5.1)	1.9 (1.6, 2.3)
	Seropositive (95% CI)	1.00 (0.80, 1.00)	0.94 (0.71, 1.00)	1.00 (0.03, 1.00)	0.94 (0.71, 1.00)	1.00 (0.80, 1.00)	1.00 (0.80, 1.00)
Day 15	n	15	15	1	1	15	15
	GMT (95% CI)	42845 (23664, 77574)	11385 (5224, 24813)	5252 (NE)	3818 (NE)	21095 (10542, 42212)	29734 (15156, 58334)
	Median (Min, Max)	29877 (8693,463855)	11101 (583,107557)	5252 (5252,5252)	3818 (3818,3818)	18783 (1645,110955)	21370 (5137,222246)
	GMR $_{D614G}$ (95% CI)	1.0 (NE)	3.8 (2.6, 5.5)	9.7 (NE)	13.3 (NE)	2.0 (1.5, 2.7)	1.4 (1.2, 1.8)
	GMFR (95% CI)	3.7 (2.4, 5.6)	11.8 (6.6, 21.1)	1.3 (NE)	1.7 (NE)	7.1 (4.5, 11.2)	4.8 (3.1, 7.2)
	Seropositive (95% CI)	1.00 (0.78, 1.00)	1.00 (0.78, 1.00)	1.00 (0.03, 1.00)	1.00 (0.03, 1.00)	1.00 (0.78, 1.00)	1.00 (0.78, 1.00)
Day 29	n	16	16	16	16	16	16
	GMT (95% CI)	28982 (18595, 45172)	6909 (3440, 13877)	ND	5355 (2915, 9835)	13357 (6912, 25813)	20227 (11976, 34162)
	Median (Min, Max)	27900 (9909,113361)	6538 (516,41083)	ND	4620 (610,38641)	17610 (1416,57189)	21628 (4974,75769)
	GMR $_{D614G}$ (95% CI)	1.0 (NE)	4.2 (3.0, 5.9)	ND	5.4 (4.0, 7.4)	2.2 (1.6, 3.0)	1.4 (1.2, 1.7)
	GMFR (95% CI)	2.4	6.2	ND	6.0	3.9	3.2

		(1.6, 3.5)	(3.7, 10.4)		(4.0, 9.1)	(2.5, 6.1)	(2.2, 4.7)
	Seropositive (95% CI)	1.00 (0.79, 1.00)	1.00 (0.79, 1.00)	ND	1.00 (0.79, 1.00)	1.00 (0.79, 1.00)	1.00 (0.79, 1.00)
Day 91	n	15	15	15	15	15	15
	GMT (95% CI)	26513 (14567, 48257)	4358 (2089, 9089)	ND	3569 (1782, 7148)	11861 (5429, 25912)	14636 (8664, 24723)
	Median (Min, Max)	18526 (6421,170951)	3555 (246,39388)	ND	2910 (183,55316)	11173 (660,88832)	13997 (4014,77082)
	GMR $_{D614G}$ (95% CI)	1.0 (NE)	6.1 (4.1, 9.0)	ND	7.4 (4.8, 11.4)	2.2 (1.6, 3.1)	1.8 (1.4, 2.3)
	GMFR (95% CI)	2.0 (1.2, 3.1)	3.6 (1.9, 6.6)	ND	3.7 (2.6, 5.4)	3.1 (1.8, 5.1)	2.0 (1.3, 3.1)
	GMFD (95% CI)	1.2 (0.9, 1.5)	1.7 (1.3, 2.3)	ND	1.7 (1.2, 2.3)	1.3 (0.9, 1.7)	1.5 (1.2, 2.0)
	Seropositive (95% CI)	1.00 (0.78, 1.00)	1.00 (0.78, 1.00)	ND	1.00 (0.78, 1.00)	1.00 (0.78, 1.00)	1.00 (0.78, 1.00)
**Arm 5: 1 Dose Omicron (BA1)**							
Day 1	n	23	23	6	23	23	23
	GMT (95% CI)	10645 (6972, 16252)	1860 (1086, 3186)	1311 (390, 4410)	1031 (617, 1723)	4416 (2690, 7249)	5421 (3448, 8525)
	Median (Min, Max)	10597 (1388,69507)	2173 (132,19859)	2028 (149,3299)	1444 (20,6388)	4958 (352,37305)	6003 (492,26178)
	GMR $_{D614G}$ (95% CI)	1.0 (NE)	5.7 (4.4, 7.5)	11.3 (4.4, 28.7)	10.3 (7.4, 14.4)	2.4 (1.9, 3.1)	2.0 (1.7, 2.3)
	Seropositive (95% CI)	1.00 (0.85, 1.00)	1.00 (0.85, 1.00)	1.00 (0.54, 1.00)	0.96 (0.78, 1.00)	1.00 (0.85, 1.00)	1.00 (0.85, 1.00)
Day 15	n	23	23	6	6	23	23
	GMT (95% CI)	31039 (24607, 39154)	14441 (10744, 19411)	4294 (1741, 10593)	2349 (927, 5950)	25132 (19186, 32921)	21107 (16386, 27189)
	Median (Min, Max)	31796 (10186,87762)	14334 (2454,56100)	5299 (847,10183)	2688 (612,7213)	27235 (4387,73729)	22131 (5417,55396)
	GMR $_{D614G}$ (95% CI)	1.0 (NE)	2.1 (1.8, 2.6)	5.5 (3.0, 9.9)	10.0 (6.1, 16.4)	1.2 (1.1, 1.4)	1.5 (1.3, 1.6)
	GMFR (95% CI)	2.9 (1.8, 4.6)	7.8 (4.3, 14.0)	3.3 (0.7, 14.7)	3.4 (0.5, 21.1)	5.7 (3.4, 9.5)	3.9 (2.4, 6.3)
	Seropositive (95% CI)	1.00 (0.85, 1.00)	1.00 (0.85, 1.00)	1.00 (0.54, 1.00)	1.00 (0.54, 1.00)	1.00 (0.85, 1.00)	1.00 (0.85, 1.00)
Day 29	n	23	22	23	23	23	23
	GMT (95% CI)	23170 (17851, 30075)	9359 (6695, 13082)	ND	3691 (2654, 5135)	16174 (12010, 21783)	13243 (10163, 17258)

	Median (Min, Max)	24694 (8174,70560)	11171 (2068,37021)	ND	4262 (464,11342)	15274 (3972,67552)	14989 (4545,54412)
	GMR $_{D614G}$ (95% CI)	1.0 (NE)	2.4 (1.9, 3.0)	ND	6.3 (5.0, 7.9)	1.4 (1.2, 1.7)	1.7 (1.6, 1.9)
	GMFR (95% CI)	2.2 (1.5, 3.2)	5.2 (3.0, 9.2)	ND	3.6 (2.3, 5.5)	3.7 (2.2, 6.0)	2.4 (1.6, 3.8)
	Seropositive (95% CI)	1.00 (0.85, 1.00)	1.00 (0.85, 1.00)	ND	1.00 (0.85, 1.00)	1.00 (0.85, 1.00)	1.00 (0.85, 1.00)
Day 91	n	22	22	22	22	22	22
	GMT (95% CI)	23587 (17653, 31516)	5499 (3754, 8055)	ND	2923 (1881, 4543)	14719 (10483, 20668)	12555 (9069, 17380)
	Median (Min, Max)	23162 (6298,74410)	6437 (299,12610)	ND	3946 (180,8533)	15823 (1551,44205)	11994 (1896,38034)
	GMR $_{D614G}$ (95% CI)	1.0 (NE)	4.3 (3.3, 5.6)	ND	8.1 (6.2, 10.4)	1.6 (1.3, 1.9)	1.9 (1.7, 2.1)
	GMFR (95% CI)	2.2 (1.3, 3.7)	3.0 (1.5, 5.8)	ND	2.9 (1.7, 4.8)	3.4 (1.9, 6.0)	2.3 (1.3, 4.2)
	GMFD (95% CI)	1.0 (0.8, 1.2)	1.8 (1.3, 2.4)		1.3 (1.0, 1.6)	1.1 (0.9, 1.4)	1.1 (0.9, 1.4)
	Seropositive (95% CI)	1.00 (0.85, 1.00)	1.00 (0.85, 1.00)	ND	1.00 (0.85, 1.00)	1.00 (0.85, 1.00)	1.00 (0.85, 1.00)
**Arm 6: 1 Dose Omicron (BA1)+Prototype**							
Day 1	n	20	20	2	20	20	20
	GMT (95% CI)	10902 (4390, 27076)	1763 (673, 4619)	7716 (0, 202812307)	1111 (477, 2584)	4165 (1674, 10361)	5370 (2290, 12596)
	Median (Min, Max)	21719 (20,82159)	2882 (20,34602)	7716 (3464,17189)	1376 (20,24191)	6470 (20,75039)	9123 (20,52883)
	GMR $_{D614G}$ (95% CI)	1.0 (NE)	6.2 (4.1, 9.3)	6.6 (0.1, 388.6)	9.8 (6.4, 15.2)	2.6 (2.1, 3.3)	2.0 (1.7, 2.4)
	Seropositive (95% CI)	0.95 (0.75, 1.00)	0.90 (0.68, 0.99)	1.00 (0.16, 1.00)	0.90 (0.68, 0.99)	0.95 (0.75, 1.00)	0.95 (0.75, 1.00)
Day 15	n	20	20	2	2	20	20
	GMT (95% CI)	57011 (38113, 85280)	19160 (11875, 30914)	21146 (1, 577554179)	13507 (1, 324268602)	34324 (20763, 56743)	33893 (22070, 52050)
	Median (Min, Max)	62715 (7241,183288)	21110 (2345,79174)	21146 (9464,47247)	13507 (6107,29875)	33056 (3226,214521)	34458 (5231,144352)
	GMR $_{D614G}$ (95% CI)	1.0 (NE)	3.0 (2.4, 3.7)	5.2 (0.1, 511.4)	8.2 (0.1, 703.7)	1.7 (1.3, 2.1)	1.7 (1.4, 2.0)
	GMFR (95% CI)	5.2 (2.3, 12.1)	10.9 (4.1, 28.6)	2.7 (2.6, 2.8)	3.0 (0.1, 178.0)	8.2 (3.5, 19.4)	6.3 (2.8, 14.1)

	Seropositive (95% CI)	1.00 (0.83, 1.00)	1.00 (0.83, 1.00)	1.00 (0.16, 1.00)	1.00 (0.16, 1.00)	1.00 (0.83, 1.00)	1.00 (0.83, 1.00)
Day 29	n	19	19	19	19	19	19
	GMT (95% CI)	37270 (25040, 55472)	9201 (3915, 21622)	ND	4419 (1950, 10012)	16575 (6555, 41909)	23677 (15111, 37099)
	Median (Min, Max)	40291 (5968,160182)	14381 (20,66771)	ND	4623 (20,39022)	19300 (20,124525)	19547 (2623,79451)
	GMR $_{D614G}$ (95% CI)	1.0 (NE)	4.1 (2.0, 8.4)	ND	8.4 (4.3, 16.5)	2.2 (1.0, 5.0)	1.6 (1.3, 1.8)
	GMFR (95% CI)	3.6 (1.5, 8.5)	5.6 (2.5, 12.4)	ND	4.4 (2.4, 7.9)	4.2 (2.3, 7.6)	4.6 (1.9, 10.8)
	Seropositive (95% CI)	1.00 (0.82, 1.00)	0.95 (0.74, 1.00)	ND	0.95 (0.74, 1.00)	0.95 (0.74, 1.00)	1.00 (0.82, 1.00)
Day 91	n	17	17	17	17	17	17
	GMT (95% CI)	34868 (22395, 54288)	7026 (2841, 17375)	ND	4146 (1811, 9495)	18303 (11223, 29850)	18987 (11972, 30113)
	Median (Min, Max)	38238 (9105,216537)	11732 (20,40505)	ND	5070 (20,23421)	18728 (4192,147217)	22583 (4298,87468)
	GMR $_{D614G}$ (95% CI)	1.0 (NE)	5.0 (2.3, 10.7)	ND	8.4 (4.4, 16.1)	1.9 (1.6, 2.3)	1.8 (1.5, 2.2)
	GMFR (95% CI)	2.9 (1.2, 7.0)	3.6 (1.5, 8.6)	ND	2.9 (1.7, 5.1)	3.8 (1.5, 9.3)	3.3 (1.5, 7.5)
	GMFD (95% CI)	1.2 (1.0, 1.5)	1.5 (1.1, 2.1)	ND	1.2 (0.9, 1.6)	1.0 (0.4, 2.6)	1.5 (1.1, 1.9)
	Seropositive (95% CI)	1.00 (0.80, 1.00)	0.94 (0.71, 1.00)	ND	0.94 (0.71, 1.00)	1.00 (0.80, 1.00)	1.00 (0.80, 1.00)

n=Number of subjects with results available at time point. ND = not done.
Confidence intervals of the geometric means were calculated with the Student's t distribution on log-transformed data
[a]Arm 2 and 3 combined for all timepoints prior to subjects in arm 2 received second dose and excluded thereafter.

Table S8: Stage 2 (Pfizer) Pseudovirus Neutralization Assay Summary Results by Arm, Time Point and Variant for Uninfected Participants

Time Point	Statistic	D614G	BA1	BA.4/5	B.1.351	B.1.617.2
**Arm 7: 1 Dose Wildtype**						
Day 1	n	33	33	33	33	33
	GMT (95% CI)	2195 (1515, 3181)	137 (80, 234)	81 (51, 128)	452 (267, 766)	995 (646, 1531)
	Median (Min, Max)	2463 (353,20214)	177 (20,2638)	116 (20,840)	662 (20,5990)	1056 (20,7926)
	GMR $_{D614G}$ (95% CI)	1.0 (NE)	16.0 (11.2, 22.8)	27.2 (18.6, 39.7)	4.9 (3.6, 6.5)	2.2 (1.8, 2.6)
	Seropositive (95% CI)	1.00 (0.89, 1.00)	0.70 (0.51, 0.84)	0.58 (0.39, 0.75)	0.88 (0.72, 0.97)	0.97 (0.84, 1.00)
Day 15	n	30	30	30	30	30
	GMT (95% CI)	14381 (11061, 18697)	1428 (954, 2136)	ND	4410 (3185, 6106)	6883 (5272, 8987)
	Median (Min, Max)	13363 (4662,55971)	1432 (20,5983)	ND	4844 (528,20627)	6131 (1753,28573)
	GMR $_{D614G}$ (95% CI)	1.0 (NE)	10.1 (7.4, 13.8)	ND	3.3 (2.6, 4.0)	2.1 (1.8, 2.4)
	GMFR (95% CI)	6.3 (4.7, 8.5)	10.1 (6.5, 15.5)	ND	9.1 (6.3, 13.2)	6.6 (4.6, 9.4)
	Seropositive (95% CI)	1.00 (0.88, 1.00)	0.97 (0.83, 1.00)	ND	1.00 (0.88, 1.00)	1.00 (0.88, 1.00)
Day 29	n	32	32	32	32	32
	GMT (95% CI)	11600 (9059, 14852)	888 (610, 1295)	485 (322, 730)	3313 (2403, 4568)	5890 (4632, 7489)
	Median (Min, Max)	12485 (2191,49554)	874 (20,4064)	595 (20,3157)	3356 (234,13614)	5485 (830,20766)
	GMR $_{D614G}$ (95% CI)	1.0 (NE)	13.1 (10.0, 17.1)	23.9 (16.8, 34.1)	3.5 (2.8, 4.4)	2.0 (1.8, 2.2)
	GMFR (95% CI)	5.3 (3.9, 7.2)	6.4 (4.3, 9.6)	6.2 (4.4, 8.6)	7.3 (5.1, 10.6)	5.9 (4.2, 8.3)
	Seropositive (95% CI)	1.00 (0.89, 1.00)	0.97 (0.84, 1.00)	0.94 (0.79, 0.99)	1.00 (0.89, 1.00)	1.00 (0.89, 1.00)
Day 91	n	23	23	23	23	23
	GMT (95% CI)	5086 (3718, 6959)	439 (241, 799)	230 (122, 434)	1098 (647, 1862)	2520 (1774, 3578)
	Median (Min, Max)	4751 (680,23039)	755 (20,2063)	360 (20,1654)	1344 (20,5062)	2650 (225,9340)
	GMR $_{D614G}$ (95% CI)	1.0 (NE)	11.6 (7.5, 17.8)	22.1 (13.5, 36.2)	4.6 (3.4, 6.4)	2.0 (1.8, 2.2)
	GMFR (95% CI)	2.1 (1.5, 2.9)	2.4 (1.6, 3.6)	2.7 (1.8, 4.0)	2.3 (1.6, 3.4)	2.3 (1.6, 3.3)
	GMFD (95% CI)	2.5 (2.0, 3.2)	2.1 (1.6, 2.9)	2.1 (1.5, 3.0)	3.2 (2.5, 4.2)	2.5 (2.0, 3.1)
	Seropositive (95% CI)	1.00 (0.85, 1.00)	0.87 (0.66, 0.97)	0.78 (0.56, 0.93)	0.96 (0.78, 1.00)	1.00 (0.85, 1.00)
**Arm 8: 1 Dose Beta (B.1.351)+Omicron (BA1)**						
Day 1	n	34	34	34	34	34
	GMT (95% CI)	2246 (1569, 3215)	128 (76, 216)	67 (41, 109)	502 (297, 850)	966 (602, 1549)
	Median (Min, Max)	2491 (117,13492)	213 (20,1133)	20 (20,1248)	828 (20,3499)	1240 (20,6078)
	GMR $_{D614G}$ (95% CI)	1.0 (NE)	17.5 (12.7, 24.3)	33.4 (23.4, 47.7)	4.5 (3.3, 6.1)	2.3 (1.8, 2.9)
	Seropositive (95% CI)	1.00 (0.90, 1.00)	0.65 (0.46, 0.80)	0.47 (0.30, 0.65)	0.88 (0.73, 0.97)	0.94 (0.80, 0.99)

Day 15	n	33	33	33	33	33
	GMT (95% CI)	12810 (9782, 16774)	2574 (1830, 3622)	ND	6363 (4635, 8736)	6852 (5217, 9000)
	Median (Min, Max)	15923 (1923,44676)	3693 (449,10214)	ND	7965 (903,28525)	8769 (1251,26208)
	GMR $_{D614G}$ (95% CI)	1.0 (NE)	5.0 (4.1, 6.0)	ND	2.0 (1.7, 2.3)	1.9 (1.7, 2.0)
	GMFR (95% CI)	5.2 (4.1, 6.6)	19.0 (12.9, 28.0)	ND	11.9 (8.1, 17.4)	6.6 (4.8, 9.1)
	Seropositive (95% CI)	1.00 (0.89, 1.00)	1.00 (0.89, 1.00)	ND	1.00 (0.89, 1.00)	1.00 (0.89, 1.00)
Day 29	n	32	32	32	32	32
	GMT (95% CI)	12284 (9266, 16286)	1653 (1138, 2400)	839 (583, 1208)	6071 (4157, 8868)	6492 (4769, 8838)
	Median (Min, Max)	11236 (2032,40071)	1648 (158,6763)	918 (147,6891)	6656 (750,41709)	6253 (788,25300)
	GMR $_{D614G}$ (95% CI)	1.0 (NE)	7.4 (6.0, 9.2)	14.6 (11.5, 18.6)	2.0 (1.7, 2.4)	1.9 (1.7, 2.1)
	GMFR (95% CI)	4.9 (4.0, 6.0)	11.5 (7.9, 16.8)	11.6 (7.7, 17.3)	11.2 (7.7, 16.4)	6.3 (4.6, 8.5)
	Seropositive (95% CI)	1.00 (0.89, 1.00)	1.00 (0.89, 1.00)	1.00 (0.89, 1.00)	1.00 (0.89, 1.00)	1.00 (0.89, 1.00)
Day 91	n	26	26	26	26	26
	GMT (95% CI)	4652 (3194, 6776)	881 (490, 1586)	447 (288, 693)	2117 (1344, 3333)	2535 (1728, 3719)
	Median (Min, Max)	3980 (747,29485)	970 (20,9171)	492 (20,3925)	2161 (343,21517)	2677 (274,14171)
	GMR $_{D614G}$ (95% CI)	1.0 (NE)	5.3 (3.9, 7.1)	10.4 (7.9, 13.6)	2.2 (1.8, 2.7)	1.8 (1.6, 2.1)
	GMFR (95% CI)	1.9 (1.6, 2.3)	6.7 (4.3, 10.5)	7.2 (4.6, 11.1)	4.4 (3.0, 6.3)	2.7 (1.9, 3.7)
	GMFD (95% CI)	2.4 (2.0, 2.8)	1.7 (1.3, 2.3)	1.6 (1.3, 2.0)	2.5 (2.1, 2.9)	2.3 (2.0, 2.6)
	Seropositive (95% CI)	1.00 (0.87, 1.00)	0.92 (0.75, 0.99)	0.96 (0.80, 1.00)	1.00 (0.87, 1.00)	1.00 (0.87, 1.00)
**Arm 9: 1 Dose Omicron (BA1)**						
Day 1	n	36	36	36	36	36
	GMT (95% CI)	2533 (1688, 3803)	162 (97, 269)	86 (54, 139)	489 (275, 870)	1090 (691, 1719)
	Median (Min, Max)	2736 (165,25184)	198 (20,2047)	102 (20,1442)	716 (20,7052)	1406 (20,9096)
	GMR $_{D614G}$ (95% CI)	1.0 (NE)	15.7 (11.9, 20.7)	29.4 (21.6, 39.9)	5.2 (3.9, 6.9)	2.3 (1.9, 2.8)
	Seropositive (95% CI)	1.00 (0.90, 1.00)	0.72 (0.55, 0.86)	0.58 (0.41, 0.74)	0.86 (0.71, 0.95)	0.97 (0.85, 1.00)
Day 15	n	34	34	34	34	34
	GMT (95% CI)	12646 (8461, 18903)	3429 (2191, 5367)	ND	6873 (4498, 10501)	6835 (4468, 10454)
	Median (Min, Max)	10810 (668,131426)	3098 (97,43522)	ND	7301 (442,76207)	5470 (142,55500)
	GMR $_{D614G}$ (95% CI)	1.0 (NE)	3.7 (3.1, 4.4)	ND	1.8 (1.6, 2.1)	1.9 (1.6, 2.1)
	GMFR (95% CI)	4.8 (3.3, 6.9)	20.4 (12.8, 32.5)	ND	13.7 (8.6, 21.9)	6.1 (4.0, 9.4)
	Seropositive (95% CI)	1.00 (0.90, 1.00)	1.00 (0.90, 1.00)	ND	1.00 (0.90, 1.00)	1.00 (0.90, 1.00)
Day 29	n	33	33	33	33	33

	GMT (95% CI)	10951 (7406, 16192)	2480 (1660, 3705)	1054 (674, 1649)	6253 (3979, 9829)	6002 (3914, 9203)
	Median (Min, Max)	10820 (715,69205)	2766 (142,22292)	1052 (20,12292)	7411 (362,47557)	6031 (185,42274)
	GMR $_{D614G}$ (95% CI)	1.0 (NE)	4.4 (3.6, 5.4)	10.4 (7.9, 13.7)	1.8 (1.5, 2.1)	1.8 (1.6, 2.1)
	GMFR (95% CI)	4.3 (3.3, 5.7)	15.0 (9.7, 23.2)	11.4 (7.0, 18.4)	12.8 (8.5, 19.2)	5.5 (3.7, 8.4)
	Seropositive (95% CI)	1.00 (0.89, 1.00)	1.00 (0.89, 1.00)	0.97 (0.84, 1.00)	1.00 (0.89, 1.00)	1.00 (0.89, 1.00)
Day 91	n	27	27	27	27	27
	GMT (95% CI)	4860 (3028, 7802)	1494 (973, 2294)	513 (276, 954)	2178 (1211, 3916)	2783 (1697, 4564)
	Median (Min, Max)	6277 (194,22743)	1928 (146,11544)	754 (20,10916)	2998 (20,21607)	2946 (85,21986)
	GMR $_{D614G}$ (95% CI)	1.0 (NE)	3.3 (2.6, 4.1)	9.5 (6.4, 14.0)	2.2 (1.9, 2.7)	1.7 (1.6, 2.0)
	GMFR (95% CI)	2.0 (1.5, 2.6)	11.1 (7.1, 17.3)	6.0 (3.5, 10.2)	5.3 (3.4, 8.1)	2.7 (1.8, 4.1)
	GMFD (95% CI)	2.2 (1.8, 2.8)	1.6 (1.3, 1.8)	1.8 (1.2, 2.6)	2.6 (2.1, 3.3)	2.1 (1.7, 2.5)
	Seropositive (95% CI)	1.00 (0.87, 1.00)	1.00 (0.87, 1.00)	0.89 (0.71, 0.98)	0.96 (0.81, 1.00)	1.00 (0.87, 1.00)
**Arm 10: 1 Dose Beta (B.1.351)**						
Day 1	n	33	33	33	33	33
	GMT (95% CI)	2231 (1317, 3782)	122 (68, 216)	77 (45, 131)	502 (302, 836)	1015 (618, 1667)
	Median (Min, Max)	2245 (20,37778)	134 (20,3289)	20 (20,2072)	595 (20,7413)	870 (20,21254)
	GMR $_{D614G}$ (95% CI)	1.0 (NE)	18.3 (12.4, 27.1)	29.2 (19.3, 44.0)	4.4 (3.4, 5.8)	2.2 (2.0, 2.5)
	Seropositive (95% CI)	0.97 (0.84, 1.00)	0.64 (0.45, 0.80)	0.48 (0.31, 0.66)	0.91 (0.76, 0.98)	0.97 (0.84, 1.00)
Day 15	n	32	32	1	32	32
	GMT (95% CI)	13936 (9297, 20889)	2198 (1385, 3489)	169 (NE)	7226 (4658, 11211)	6636 (4448, 9902)
	Median (Min, Max)	14876 (537,211875)	1698 (20,22764)	169 (169,169)	7315 (178,64198)	5960 (299,61700)
	GMR $_{D614G}$ (95% CI)	1.0 (NE)	6.3 (5.3, 7.6)	9.4 (NE)	1.9 (1.6, 2.3)	2.1 (1.9, 2.3)
	GMFR (95% CI)	6.6 (4.9, 8.8)	19.8 (12.8, 30.5)	8.5 (NE)	15.2 (10.4, 22.3)	6.9 (5.2, 9.2)
	Seropositive (95% CI)	1.00 (0.89, 1.00)	0.97 (0.84, 1.00)	1.00 (0.03, 1.00)	1.00 (0.89, 1.00)	1.00 (0.89, 1.00)
Day 29	n	31	31	31	31	31
	GMT (95% CI)	11335 (7725, 16631)	1411 (925, 2154)	914 (588, 1420)	6247 (4050, 9635)	6355 (4238, 9529)
	Median (Min, Max)	11212 (531,70530)	1072 (63,10618)	1081 (66,12048)	5720 (115,33960)	6404 (187,44039)
	GMR $_{D614G}$ (95% CI)	1.0 (NE)	8.0 (6.5, 9.9)	12.4 (9.7, 15.9)	1.8 (1.6, 2.1)	1.8 (1.6, 2.0)
	GMFR (95% CI)	5.7 (4.1, 7.8)	13.8 (9.3, 20.5)	13.6 (9.2, 20.2)	13.9 (9.5, 20.3)	6.9 (5.0, 9.6)
	Seropositive (95% CI)	1.00 (0.89, 1.00)	1.00 (0.89, 1.00)	1.00 (0.89, 1.00)	1.00 (0.89, 1.00)	1.00 (0.89, 1.00)
Day 91	n	25	25	25	25	25
	GMT (95% CI)	4283 (2671, 6867)	717 (419, 1225)	309 (150, 636)	2057 (1252, 3382)	2244 (1384, 3636)
	Median (Min, Max)	3458 (355,79254)	683 (20,8699)	563 (20,3718)	1810 (157,32804)	2141 (155,38235)
	GMR $_{D614G}$ (95% CI)	1.0 (NE)	6.0 (4.5, 7.9)	13.9 (8.9, 21.7)	2.1 (1.7, 2.5)	1.9 (1.8, 2.1)

	GMFR (95% CI)	2.4 (1.7, 3.4)	8.7 (5.6, 13.5)	6.2 (3.5, 10.9)	5.5 (3.7, 8.1)	2.8 (2.0, 4.0)
	GMFD (95% CI)	2.4 (1.9, 2.9)	1.7 (1.3, 2.1)	2.4 (1.6, 3.4)	2.6 (2.1, 3.3)	2.4 (1.9, 3.0)
	Seropositive (95% CI)	1.00 (0.86, 1.00)	0.96 (0.80, 1.00)	0.76 (0.55, 0.91)	1.00 (0.86, 1.00)	1.00 (0.86, 1.00)
**Arm 11: 1 Dose Beta (1.351)+Wildtype**						
Day 1	n	37	37	37	37	37
	GMT (95% CI)	3088 (2140, 4455)	174 (101, 297)	71 (44, 113)	653 (409, 1043)	1400 (964, 2032)
	Median (Min, Max)	3091 (390,36403)	205 (20,11225)	20 (20,1674)	648 (20,34731)	1379 (209,25437)
	GMR $_{D614G}$ (95% CI)	1.0 (NE)	17.8 (12.6, 25.2)	43.8 (32.0, 59.9)	4.7 (3.7, 6.0)	2.2 (2.0, 2.5)
	Seropositive (95% CI)	1.00 (0.91, 1.00)	0.73 (0.56, 0.86)	0.49 (0.32, 0.66)	0.95 (0.82, 0.99)	1.00 (0.91, 1.00)
Day 15	n	36	36	36	36	36
	GMT (95% CI)	18958 (14244, 25232)	2445 (1683, 3551)	ND	7324 (5109, 10498)	9779 (7285, 13127)
	Median (Min, Max)	19108 (3589,68797)	2808 (217,19002)	ND	8753 (563,38156)	10368 (1746,40258)
	GMR $_{D614G}$ (95% CI)	1.0 (NE)	7.8 (6.4, 9.3)	ND	2.6 (2.2, 3.0)	1.9 (1.8, 2.1)
	GMFR (95% CI)	5.9 (4.5, 7.7)	13.3 (9.0, 19.5)	ND	10.9 (7.9, 15.0)	6.6 (4.8, 9.2)
	Seropositive (95% CI)	1.00 (0.90, 1.00)	1.00 (0.90, 1.00)	ND	1.00 (0.90, 1.00)	1.00 (0.90, 1.00)
Day 29	n	35	35	35	35	35
	GMT (95% CI)	15659 (11411, 21490)	1635 (1125, 2376)	959 (634, 1450)	6018 (4156, 8715)	8335 (6029, 11523)
	Median (Min, Max)	17291 (2427,67164)	1842 (109,10650)	1010 (20,9520)	6227 (277,31571)	9151 (938,43160)
	GMR $_{D614G}$ (95% CI)	1.0 (NE)	9.6 (7.6, 12.1)	16.3 (12.4, 21.5)	2.6 (2.2, 3.1)	1.9 (1.7, 2.1)
	GMFR (95% CI)	5.0 (3.8, 6.7)	9.5 (6.4, 14.2)	14.2 (9.9, 20.4)	9.1 (6.5, 12.7)	6.0 (4.4, 8.3)
	Seropositive (95% CI)	1.00 (0.90, 1.00)	1.00 (0.90, 1.00)	0.97 (0.85, 1.00)	1.00 (0.90, 1.00)	1.00 (0.90, 1.00)
Day 91	n	29	29	29	29	29
	GMT (95% CI)	6546 (4660, 9194)	918 (547, 1540)	434 (260, 724)	2486 (1602, 3857)	3477 (2576, 4693)
	Median (Min, Max)	5914 (818,33306)	1049 (20,13320)	448 (20,4364)	2334 (116,19328)	3293 (424,22160)
	GMR $_{D614G}$ (95% CI)	1.0 (NE)	7.1 (5.3, 9.6)	15.1 (10.9, 21.0)	2.6 (2.2, 3.1)	1.9 (1.7, 2.1)
	GMFR (95% CI)	2.2 (1.7, 3.0)	5.5 (3.6, 8.6)	6.1 (4.2, 8.9)	3.8 (2.6, 5.5)	2.5 (1.8, 3.5)
	GMFD (95% CI)	2.3 (2.0, 2.7)	1.7 (1.4, 2.1)	2.1 (1.7, 2.7)	2.4 (2.0, 2.8)	2.3 (1.9, 2.8)
	Seropositive (95% CI)	1.00 (0.88, 1.00)	0.97 (0.82, 1.00)	0.93 (0.77, 0.99)	1.00 (0.88, 1.00)	1.00 (0.88, 1.00)
**Arm 12: 1 Dose Omicron (BA1)+Wildtype**						
Day 1	n	35	35	35	35	35
	GMT (95% CI)	2572 (1672, 3957)	131 (73, 235)	84 (48, 148)	454 (259, 798)	1041 (674, 1608)
	Median (Min, Max)	2533 (20,22390)	155 (20,3094)	20 (20,1696)	591 (20,9464)	1089 (20,17718)
	GMR $_{D614G}$ (95% CI)	1.0 (NE)	19.6 (12.7, 30.4)	30.5 (19.7, 47.0)	5.7 (3.9, 8.1)	2.5 (2.1, 2.9)
	Seropositive (95% CI)	0.97 (0.85, 1.00)	0.63 (0.45, 0.79)	0.49 (0.31, 0.66)	0.89 (0.73, 0.97)	0.97 (0.85, 1.00)

Day 15	n	33	33	33	33	33
	GMT (95% CI)	22430 (16634, 30245)	3764 (2394, 5919)	ND	7826 (5033, 12171)	9992 (6821, 14636)
	Median (Min, Max)	22327 (4612,119854)	2967 (376,55280)	ND	6294 (965,86968)	7430 (1114,72042)
	GMR $_{D614G}$ (95% CI)	1.0 (NE)	6.0 (4.7, 7.6)	ND	2.9 (2.2, 3.7)	2.2 (1.9, 2.7)
	GMFR (95% CI)	9.0 (6.1, 13.5)	27.6 (16.2, 47.1)	ND	17.3 (10.4, 28.7)	9.8 (6.6, 14.4)
	Seropositive (95% CI)	1.00 (0.89, 1.00)	1.00 (0.89, 1.00)	ND	1.00 (0.89, 1.00)	1.00 (0.89, 1.00)
Day 29	n	32	32	32	32	32
	GMT (95% CI)	18093 (13021, 25143)	2171 (1372, 3434)	984 (557, 1738)	5664 (3687, 8703)	8721 (6143, 12379)
	Median (Min, Max)	15200 (3763,120251)	1434 (238,25138)	890 (20,11413)	3987 (657,62332)	6944 (1748,60607)
	GMR $_{D614G}$ (95% CI)	1.0 (NE)	8.3 (6.4, 10.8)	18.4 (12.0, 28.1)	3.2 (2.5, 4.0)	2.1 (1.8, 2.3)
	GMFR (95% CI)	7.0 (4.7, 10.3)	17.3 (10.4, 28.8)	11.9 (7.2, 19.6)	13.3 (8.2, 21.6)	8.3 (5.8, 12.0)
	Seropositive (95% CI)	1.00 (0.89, 1.00)	1.00 (0.89, 1.00)	0.94 (0.79, 0.99)	1.00 (0.89, 1.00)	1.00 (0.89, 1.00)
Day 91	n	28	28	28	28	28
	GMT (95% CI)	7140 (5059, 10077)	1201 (672, 2145)	565 (293, 1090)	2378 (1480, 3824)	3807 (2545, 5697)
	Median (Min, Max)	6031 (1796,58477)	1100 (20,10419)	511 (20,10213)	1955 (269,31869)	3483 (453,25785)
	GMR $_{D614G}$ (95% CI)	1.0 (NE)	5.9 (4.0, 8.9)	12.6 (8.1, 19.7)	3.0 (2.3, 3.8)	1.9 (1.6, 2.2)
	GMFR (95% CI)	2.8 (1.8, 4.3)	9.4 (5.3, 16.9)	6.8 (3.9, 11.7)	6.0 (3.4, 10.5)	3.6 (2.4, 5.4)
	GMFD (95% CI)	2.6 (2.3, 3.0)	2.0 (1.4, 2.9)	2.0 (1.6, 2.6)	2.6 (2.2, 3.0)	2.5 (2.1, 3.0)
	Seropositive (95% CI)	1.00 (0.88, 1.00)	0.96 (0.82, 1.00)	0.89 (0.72, 0.98)	1.00 (0.88, 1.00)	1.00 (0.88, 1.00)

n=Number of subjects with results available at time point. ND = not done.
Confidence intervals of the geometric means were calculated with the Student's t distribution on log-transformed data

Table S9: Stage 2 (Pfizer) Pseudovirus Neutralization Assay Summary Results by Arm, Time Point and Variant for Previously Infected Participants

Time Point	Statistic	D614G	BA1	BA.4/5	B.1.351	B.1.617.2
**Arm 7: 1 Dose Wildtype**						
Day 1	n	17	17	17	17	17
	GMT (95% CI)	20596 (13576, 31247)	3428 (1881, 6246)	1276 (798, 2040)	7981 (4883, 13045)	10635 (6973, 16221)
	Median (Min, Max)	26516 (3973,98812)	4320 (109,15673)	1525 (205,5422)	8679 (425,40669)	11162 (1497,61193)
	GMR $_{D614G}$ (95% CI)	1.0 (NE)	6.0 (4.1, 8.8)	16.1 (10.5, 24.8)	2.6 (1.9, 3.5)	1.9 (1.6, 2.3)
	Seropositive (95% CI)	1.00 (0.80, 1.00)	1.00 (0.80, 1.00)	1.00 (0.80, 1.00)	1.00 (0.80, 1.00)	1.00 (0.80, 1.00)
Day 15	n	17	17	17	17	17
	GMT (95% CI)	44093 (29847, 65139)	7996 (4918, 13001)	ND	19945 (11845, 33583)	24333 (15959, 37101)
	Median (Min, Max)	48972 (12073,323043)	10084 (1009,51371)	ND	15808 (2012,195472)	20065 (5937,155530)
	GMR $_{D614G}$ (95% CI)	1.0 (NE)	5.5 (4.2, 7.3)	ND	2.2 (1.7, 2.8)	1.8 (1.6, 2.1)
	GMFR (95% CI)	2.1 (1.5, 3.0)	2.3 (1.6, 3.3)	ND	2.5 (1.8, 3.5)	2.3 (1.6, 3.2)
	Seropositive (95% CI)	1.00 (0.80, 1.00)	1.00 (0.80, 1.00)	ND	1.00 (0.80, 1.00)	1.00 (0.80, 1.00)
Day 29	n	17	17	17	17	17
	GMT (95% CI)	31701 (21635, 46449)	4860 (2905, 8130)	2621 (1553, 4423)	12601 (7870, 20176)	16637 (11026, 25105)
	Median (Min, Max)	32528 (5333,106005)	4228 (841,34236)	2512 (477,17191)	15643 (1143,51719)	19385 (3157,63358)
	GMR $_{D614G}$ (95% CI)	1.0 (NE)	6.5 (4.5, 9.5)	12.1 (8.5, 17.3)	2.5 (2.1, 3.0)	1.9 (1.6, 2.3)
	GMFR (95% CI)	1.5 (1.1, 2.2)	1.4 (1.0, 2.1)	2.1 (1.5, 2.7)	1.6 (1.2, 2.2)	1.6 (1.1, 2.2)
	Seropositive (95% CI)	1.00 (0.80, 1.00)	1.00 (0.80, 1.00)	1.00 (0.80, 1.00)	1.00 (0.80, 1.00)	1.00 (0.80, 1.00)
Day 91	n	15	15	15	15	15
	GMT (95% CI)	13278 (9558, 18444)	2804 (1743, 4511)	1692 (1057, 2707)	4948 (3261, 7508)	7594 (5340, 10800)
	Median (Min, Max)	14039 (4461,34007)	2878 (316,8531)	1718 (313,7131)	5726 (851,16858)	7852 (2448,18709)
	GMR $_{D614G}$ (95% CI)	1.0 (NE)	4.7 (3.3, 6.8)	7.8 (5.8, 10.6)	2.7 (2.1, 3.5)	1.7 (1.4, 2.1)
	GMFR (95% CI)	0.6 (0.5, 0.8)	0.8 (0.5, 1.1)	1.4 (1.0, 1.8)	0.6 (0.5, 0.8)	0.7 (0.5, 0.9)
	GMFD (95% CI)	2.3 (1.6, 3.2)	1.8 (1.3, 2.5)	1.6 (1.1, 2.3)	2.5 (1.7, 3.5)	2.1 (1.5, 3.0)
	Seropositive (95% CI)	1.00 (0.78, 1.00)	1.00 (0.78, 1.00)	1.00 (0.78, 1.00)	1.00 (0.78, 1.00)	1.00 (0.78, 1.00)

**Arm 8: 1 Dose Beta (B.1.351)+Omicron (BA1)**						
Day 1	n	18	18	18	18	18
	GMT (95% CI)	9650 (4037, 23064)	1599 (682, 3753)	755 (385, 1482)	4310 (1895, 9799)	5412 (2448, 11966)
	Median (Min, Max)	12634 (20,54417)	2000 (20,12893)	1046 (20,7994)	4731 (20,39231)	6002 (20,24064)
	GMR $_{D614G}$ (95% CI)	1.0 (NE)	6.0 (3.8, 9.6)	12.8 (8.3, 19.7)	2.2 (1.8, 2.9)	1.8 (1.6, 2.0)
	Seropositive (95% CI)	0.94 (0.73, 1.00)	0.94 (0.73, 1.00)	0.94 (0.73, 1.00)	0.94 (0.73, 1.00)	0.94 (0.73, 1.00)
Day 15	n	17	17	17	17	17
	GMT (95% CI)	21357 (8042, 56718)	8466 (3468, 20668)	ND	15883 (6023, 41887)	14547 (5689, 37198)
	Median (Min, Max)	28082 (20,112745)	10440 (20,43584)	ND	28354 (20,80142)	21161 (20,56644)
	GMR $_{D614G}$ (95% CI)	1.0 (NE)	2.5 (1.9, 3.3)	ND	1.3 (1.1, 1.6)	1.5 (1.3, 1.7)
	GMFR (95% CI)	2.0 (1.6, 2.7)	4.6 (2.8, 7.6)	ND	3.4 (2.3, 5.0)	2.5 (1.9, 3.3)
	Seropositive (95% CI)	0.94 (0.71, 1.00)	0.94 (0.71, 1.00)	ND	0.94 (0.71, 1.00)	0.94 (0.71, 1.00)
Day 29	n	18	18	18	18	18
	GMT (95% CI)	24962 (18477, 33722)	5684 (2395, 13492)	2743 (1250, 6022)	12711 (5223, 30934)	16584 (11647, 23612)
	Median (Min, Max)	22819 (10327,86893)	6486 (20,130644)	3036 (20,29054)	17140 (20,74162)	14903 (6702,62538)
	GMR $_{D614G}$ (95% CI)	1.0 (NE)	4.4 (2.1, 9.1)	9.1 (4.9, 17.1)	2.0 (0.9, 4.3)	1.5 (1.3, 1.8)
	GMFR (95% CI)	2.6 (1.2, 5.6)	3.6 (2.1, 6.0)	3.6 (2.2, 6.0)	2.9 (2.1, 4.2)	3.1 (1.6, 5.9)
	Seropositive (95% CI)	1.00 (0.81, 1.00)	0.94 (0.73, 1.00)	0.94 (0.73, 1.00)	0.94 (0.73, 1.00)	1.00 (0.81, 1.00)
Day 91	n	17	17	17	17	17
	GMT (95% CI)	8895 (3659, 21622)	3672 (1587, 8499)	1825 (858, 3883)	5514 (2272, 13381)	5826 (2512, 13514)
	Median (Min, Max)	12496 (20,58294)	4096 (20,34770)	2071 (20,12591)	6326 (20,50319)	7340 (20,28582)
	GMR $_{D614G}$ (95% CI)	1.0 (NE)	2.4 (1.8, 3.2)	4.9 (3.4, 6.9)	1.6 (1.3, 2.1)	1.5 (1.3, 1.8)
	GMFR (95% CI)	1.0 (0.7, 1.3)	2.4 (1.3, 4.4)	2.5 (1.5, 4.0)	1.3 (0.8, 2.0)	1.1 (0.8, 1.5)
	GMFD (95% CI)	2.9 (1.3, 6.4)	1.7 (1.2, 2.2)	1.6 (1.3, 2.0)	2.3 (1.8, 3.1)	2.8 (1.4, 5.7)
	Seropositive (95% CI)	0.94 (0.71, 1.00)	0.94 (0.71, 1.00)	0.94 (0.71, 1.00)	0.94 (0.71, 1.00)	0.94 (0.71, 1.00)
**Arm 9: 1 Dose Omicron (BA1)**						
Day 1	n	18	18	18	18	18
	GMT (95% CI)	9839 (5483, 17655)	1372 (567, 3321)	833 (407, 1707)	3284 (1451, 7433)	4713 (2331, 9528)
	Median (Min, Max)	12856 (1211,64282)	1482 (20,32004)	884 (126,18901)	3973 (137,82541)	7169 (414,51642)
	GMR $_{D614G}$ (95% CI)	1.0 (NE)	7.2 (4.5, 11.4)	11.8 (7.9, 17.7)	3.0 (2.1, 4.3)	2.1 (1.6, 2.7)
	Seropositive (95% CI)	1.00 (0.81, 1.00)	0.94 (0.73, 1.00)	1.00 (0.81, 1.00)	1.00 (0.81, 1.00)	1.00 (0.81, 1.00)
Day 15	n	18	18	18	18	18

	GMT (95% CI)	25267 (15949, 40028)	9261 (4944, 17349)	ND	14772 (7909, 27592)	14154 (8361, 23960)
	Median (Min, Max)	24964 (5625,147461)	12611 (1454,74058)	ND	20046 (2010,134074)	14958 (2635,71908)
	GMR $_{D614G}$ (95% CI)	1.0 (NE)	2.7 (2.0, 3.7)	ND	1.7 (1.3, 2.3)	1.8 (1.4, 2.3)
	GMFR (95% CI)	2.6 (2.1, 3.2)	6.7 (4.2, 10.8)	ND	4.5 (3.0, 6.6)	3.0 (2.1, 4.3)
	Seropositive (95% CI)	1.00 (0.81, 1.00)	1.00 (0.81, 1.00)	ND	1.00 (0.81, 1.00)	1.00 (0.81, 1.00)
Day 29	n	16	16	16	16	16
	GMT (95% CI)	29365 (19538, 44135)	8787 (4883, 15812)	4356 (2154, 8808)	16215 (8794, 29899)	16722 (9363, 29866)
	Median (Min, Max)	26661 (9415,93730)	10518 (1656,48232)	6545 (168,23781)	22116 (2455,71092)	18768 (2525,75938)
	GMR $_{D614G}$ (95% CI)	1.0 (NE)	3.3 (2.4, 4.6)	6.7 (4.2, 10.9)	1.8 (1.4, 2.4)	1.8 (1.4, 2.3)
	GMFR (95% CI)	2.3 (1.7, 3.2)	5.1 (2.7, 9.9)	4.3 (2.6, 7.1)	3.8 (2.2, 6.4)	2.7 (1.9, 3.7)
	Seropositive (95% CI)	1.00 (0.79, 1.00)	1.00 (0.79, 1.00)	1.00 (0.79, 1.00)	1.00 (0.79, 1.00)	1.00 (0.79, 1.00)
Day 91	n	16	16	16	16	16
	GMT (95% CI)	13261 (8959, 19627)	5702 (3141, 10350)	2653 (1354, 5196)	7011 (4158, 11823)	7475 (4492, 12437)
	Median (Min, Max)	12356 (4290,40651)	5522 (1038,21685)	3551 (172,13202)	7687 (1464,38915)	6192 (1591,30235)
	GMR $_{D614G}$ (95% CI)	1.0 (NE)	2.3 (1.7, 3.2)	5.0 (3.1, 8.0)	1.9 (1.5, 2.4)	1.8 (1.4, 2.3)
	GMFR (95% CI)	1.2 (0.8, 1.7)	3.8 (1.9, 7.8)	3.1 (1.8, 5.1)	1.9 (1.1, 3.2)	1.4 (1.0, 2.1)
	GMFD (95% CI)	2.0 (1.7, 2.3)	1.4 (1.2, 1.6)	1.4 (1.1, 1.8)	2.0 (1.6, 2.3)	1.9 (1.6, 2.3)
	Seropositive (95% CI)	1.00 (0.79, 1.00)	1.00 (0.79, 1.00)	1.00 (0.79, 1.00)	1.00 (0.79, 1.00)	1.00 (0.79, 1.00)
**Arm 10: 1 Dose Beta (B.1.351)**						
Day 1	n	18	18	18	18	18
	GMT (95% CI)	9262 (3620, 23698)	1178 (524, 2645)	502 (248, 1014)	3308 (1295, 8446)	5554 (2187, 14107)
	Median (Min, Max)	12050 (20,63201)	1455 (20,9526)	791 (20,2404)	3323 (20,34326)	6986 (20,50163)
	GMR $_{D614G}$ (95% CI)	1.0 (NE)	7.9 (4.9, 12.5)	18.5 (10.6, 32.1)	2.8 (1.8, 4.3)	1.7 (1.4, 2.0)
	Seropositive (95% CI)	0.94 (0.73, 1.00)	0.94 (0.73, 1.00)	0.89 (0.65, 0.99)	0.94 (0.73, 1.00)	0.94 (0.73, 1.00)
Day 15	n	18	18	18	18	18
	GMT (95% CI)	30370 (16391, 56273)	6415 (2614, 15739)	ND	18833 (9060, 39146)	17162 (9196, 32029)
	Median (Min, Max)	37369 (530,126055)	7968 (20,55606)	ND	20796 (311,127298)	21997 (342,95021)
	GMR $_{D614G}$ (95% CI)	1.0 (NE)	4.7 (3.2, 6.9)	ND	1.6 (1.2, 2.2)	1.8 (1.5, 2.0)

	GMFR (95% CI)	3.3 (2.0, 5.4)	5.4 (3.2, 9.4)	ND	5.7 (3.2, 10.3)	3.1 (1.8, 5.2)
	Seropositive (95% CI)	1.00 (0.81, 1.00)	0.94 (0.73, 1.00)	ND	1.00 (0.81, 1.00)	1.00 (0.81, 1.00)
Day 29	n	18	18	18	18	18
	GMT (95% CI)	22096 (12272, 39783)	4232 (2055, 8715)	2334 (1146, 4753)	13736 (6630, 28455)	13432 (7134, 25287)
	Median (Min, Max)	28939 (575,103863)	5377 (96,34567)	3479 (20,11945)	18736 (224,72221)	19325 (251,52781)
	GMR $_{D614G}$ (95% CI)	1.0 (NE)	5.2 (3.6, 7.5)	9.5 (6.8, 13.1)	1.6 (1.2, 2.2)	1.6 (1.3, 2.0)
	GMFR (95% CI)	2.4 (1.4, 4.1)	3.6 (2.2, 5.9)	4.7 (2.8, 7.8)	4.2 (2.4, 7.1)	2.4 (1.5, 3.8)
	Seropositive (95% CI)	1.00 (0.81, 1.00)	1.00 (0.81, 1.00)	0.94 (0.73, 1.00)	1.00 (0.81, 1.00)	1.00 (0.81, 1.00)
Day 91	n	18	18	18	18	18
	GMT (95% CI)	11035 (5755, 21157)	2459 (1107, 5462)	1496 (756, 2960)	6071 (3013, 12236)	6510 (3491, 12139)
	Median (Min, Max)	14411 (180,56677)	3220 (20,22758)	2412 (20,6754)	8052 (112,45678)	7809 (125,26702)
	GMR $_{D614G}$ (95% CI)	1.0 (NE)	4.5 (3.2, 6.3)	7.4 (5.8, 9.4)	1.8 (1.4, 2.4)	1.7 (1.5, 2.0)
	GMFR (95% CI)	1.2 (0.8, 1.8)	2.1 (1.3, 3.3)	3.0 (1.9, 4.6)	1.8 (1.1, 3.0)	1.2 (0.8, 1.8)
	GMFD (95% CI)	2.0 (1.5, 2.7)	1.7 (1.2, 2.4)	1.6 (1.2, 2.0)	2.3 (1.6, 3.1)	2.1 (1.7, 2.6)
	Seropositive (95% CI)	1.00 (0.81, 1.00)	0.94 (0.73, 1.00)	0.94 (0.73, 1.00)	1.00 (0.81, 1.00)	1.00 (0.81, 1.00)
**Arm 11: 1 Dose Beta (1.351)+Wildtype**						
Day 1	n	15	15	15	15	15
	GMT (95% CI)	10732 (5552, 20744)	2183 (865, 5506)	966 (464, 2009)	5119 (2090, 12538)	7062 (3518, 14176)
	Median (Min, Max)	9164 (1716,47184)	5711 (155,14787)	1258 (169,6255)	7651 (522,54215)	5296 (1086,35361)
	GMR $_{D614G}$ (95% CI)	1.0 (NE)	4.9 (3.2, 7.5)	11.1 (7.9, 15.6)	2.1 (1.4, 3.2)	1.5 (1.3, 1.8)
	Seropositive (95% CI)	1.00 (0.78, 1.00)	1.00 (0.78, 1.00)	1.00 (0.78, 1.00)	1.00 (0.78, 1.00)	1.00 (0.78, 1.00)
Day 15	n	15	15	15	15	15
	GMT (95% CI)	33730 (18649, 61006)	8860 (4667, 16820)	ND	20415 (10950, 38062)	19638 (11167, 34536)
	Median (Min, Max)	37902 (4615,145376)	10527 (1280,53600)	ND	22806 (3128,134162)	26828 (2437,80311)
	GMR $_{D614G}$ (95% CI)	1.0 (NE)	3.8 (3.2, 4.6)	ND	1.7 (1.3, 2.0)	1.7 (1.4, 2.1)
	GMFR (95% CI)	3.1 (2.3, 4.2)	4.1 (2.5, 6.7)	ND	4.0 (2.4, 6.7)	2.8 (2.0, 3.9)
	Seropositive (95% CI)	1.00 (0.78, 1.00)	1.00 (0.78, 1.00)	ND	1.00 (0.78, 1.00)	1.00 (0.78, 1.00)
Day 29	n	15	15	15	15	15
	GMT (95% CI)	30962 (16533, 57981)	6499 (3336, 12661)	3448 (1783, 6665)	14002 (7309, 26824)	17177 (8810, 33488)

	Median (Min, Max)	22920 (3961,280235)	6684 (1093,42631)	2872 (686,51972)	17111 (2213,87399)	15893 (2622,160039)
	GMR $_{D614G}$ (95% CI)	1.0 (NE)	4.8 (3.9, 5.8)	9.0 (5.7, 14.0)	2.2 (1.8, 2.7)	1.8 (1.5, 2.1)
	GMFR (95% CI)	2.9 (2.0, 4.1)	3.0 (1.8, 5.1)	3.6 (2.2, 5.7)	2.7 (1.5, 5.0)	2.4 (1.7, 3.5)
	Seropositive (95% CI)	1.00 (0.78, 1.00)	1.00 (0.78, 1.00)	1.00 (0.78, 1.00)	1.00 (0.78, 1.00)	1.00 (0.78, 1.00)
Day 91	n	14	14	14	14	14
	GMT (95% CI)	12922 (7407, 22542)	3808 (1896, 7646)	2052 (1031, 4084)	7055 (3724, 13365)	8180 (4700, 14234)
	Median (Min, Max)	11500 (2705,50281)	4018 (421,27598)	2240 (343,29812)	8044 (971,33925)	8718 (2203,28112)
	GMR $_{D614G}$ (95% CI)	1.0 (NE)	3.4 (2.6, 4.5)	6.3 (4.3, 9.2)	1.8 (1.5, 2.3)	1.6 (1.4, 1.8)
	GMFR (95% CI)	1.1 (0.8, 1.4)	1.4 (0.8, 2.5)	1.9 (1.1, 3.1)	1.2 (0.7, 2.0)	1.0 (0.8, 1.4)
	GMFD (95% CI)	2.8 (2.0, 3.8)	1.9 (1.5, 2.4)	1.9 (1.7, 2.1)	2.3 (1.6, 3.1)	2.4 (1.9, 3.1)
	Seropositive (95% CI)	1.00 (0.77, 1.00)	1.00 (0.77, 1.00)	1.00 (0.77, 1.00)	1.00 (0.77, 1.00)	1.00 (0.77, 1.00)
**Arm 12: 1 Dose Omicron (BA1)+Wildtype**						
Day 1	n	18	18	18	18	18
	GMT (95% CI)	15053 (8479, 26724)	2295 (944, 5580)	838 (359, 1957)	5585 (2656, 11744)	7463 (4227, 13177)
	Median (Min, Max)	16381 (1111,108658)	2325 (20,45341)	1128 (20,12235)	5952 (158,73695)	8072 (480,59195)
	GMR $_{D614G}$ (95% CI)	1.0 (NE)	6.6 (4.0, 10.8)	18.0 (11.6, 27.7)	2.7 (1.9, 3.8)	2.0 (1.7, 2.4)
	Seropositive (95% CI)	1.00 (0.81, 1.00)	0.94 (0.73, 1.00)	0.89 (0.65, 0.99)	1.00 (0.81, 1.00)	1.00 (0.81, 1.00)
Day 15	n	18	18	18	18	18
	GMT (95% CI)	39480 (25202, 61847)	10593 (5504, 20387)	ND	20903 (12076, 36183)	21511 (13961, 33143)
	Median (Min, Max)	32555 (9262,227629)	9844 (1473,126146)	ND	14855 (4370,176367)	18361 (6224,109198)
	GMR $_{D614G}$ (95% CI)	1.0 (NE)	3.7 (2.6, 5.3)	ND	1.9 (1.4, 2.5)	1.8 (1.5, 2.3)
	GMFR (95% CI)	2.6 (1.7, 4.1)	4.6 (2.6, 8.3)	ND	3.7 (2.2, 6.5)	2.9 (1.8, 4.6)
	Seropositive (95% CI)	1.00 (0.81, 1.00)	1.00 (0.81, 1.00)	ND	1.00 (0.81, 1.00)	1.00 (0.81, 1.00)
Day 29	n	18	18	18	18	18
	GMT (95% CI)	36133 (24409, 53488)	6456 (3152, 13220)	3651 (1886, 7070)	18712 (10670, 32818)	18391 (12326, 27438)
	Median (Min, Max)	27268 (13803,167814)	4741 (968,124365)	2179 (797,80699)	15496 (3218,183071)	15738 (6739,85391)
	GMR $_{D614G}$ (95% CI)	1.0 (NE)	5.6 (3.3, 9.6)	9.9 (6.3, 15.5)	1.9 (1.4, 2.6)	2.0 (1.7, 2.3)
	GMFR (95% CI)	2.4 (1.5, 3.9)	2.8 (1.6, 5.1)	4.4 (2.2, 8.7)	3.4 (1.9, 5.8)	2.5 (1.5, 4.0)
	Seropositive (95% CI)	1.00 (0.81, 1.00)	1.00 (0.81, 1.00)	1.00 (0.81, 1.00)	1.00 (0.81, 1.00)	1.00 (0.81, 1.00)

Day 91	n	15	15	15	15	15
	GMT (95% CI)	15647 (9190, 26642)	6678 (3369, 13237)	2473 (1258, 4860)	7752 (3997, 15037)	8679 (4971, 15155)
	Median (Min, Max)	14160 (3927,90408)	4277 (909,51818)	1740 (394,20938)	4700 (1734,58324)	8944 (2034,40208)
	GMR $_{D614G}$ (95% CI)	1.0 (NE)	2.3 (1.5, 3.6)	6.3 (4.3, 9.4)	2.0 (1.4, 2.9)	1.8 (1.6, 2.1)
	GMFR (95% CI)	0.9 (0.6, 1.5)	2.2 (1.1, 4.5)	2.2 (1.2, 4.0)	1.1 (0.6, 2.1)	1.0 (0.6, 1.8)
	GMFD (95% CI)	2.7 (2.1, 3.4)	1.3 (0.8, 2.0)	1.8 (1.2, 2.6)	3.0 (2.1, 4.3)	2.5 (2.0, 3.1)
	Seropositive (95% CI)	1.00 (0.78, 1.00)	1.00 (0.78, 1.00)	1.00 (0.78, 1.00)	1.00 (0.78, 1.00)	1.00 (0.78, 1.00)

n=Number of subjects with results available at time point. ND = not done.
Confidence intervals of the geometric means were calculated with the Student’s t distribution on log-transformed data

Table S10: Stage 3 (Sanofi) Pseudovirus Neutralization Assay Summary Results by Arm, Time Point and Variant for Uninfected Participants

Time Point	Statistic	D614G	BA1	BA.4/5	B.1.351	B.1.617.2
**Arm 13: 1 Dose Prototype**						
Day 1	n	27	27	27	27	27
	GMT (95% CI)	2438 (1694, 3509)	116 (67, 202)	69 (39, 123)	484 (264, 887)	1236 (857, 1783)
	Median (Min , Max)	2747 (405,13861)	172 (20,1429)	20 (20,2743)	437 (20,4383)	1322 (254,7389)
	GMR $_{D614G}$ (95% CI)	1.0 (NE)	21.0 (14.6, 30.2)	35.3 (23.1, 54.0)	5.0 (3.3, 7.6)	2.0 (1.7, 2.2)
	Seropositive (95% CI)	1.00 (0.87, 1.00)	0.67 (0.46, 0.83)	0.48 (0.29, 0.68)	0.89 (0.71, 0.98)	1.00 (0.87, 1.00)
Day 29	n	25	25	25	25	25
	GMT (95% CI)	6942 (4680, 10299)	667 (430, 1032)	525 (323, 854)	2437 (1447, 4103)	3739 (2455, 5696)
	Median (Min , Max)	5608 (1951,95264)	588 (136,21523)	509 (63,6818)	2271 (282,66253)	3006 (902,81333)
	GMR $_{D614G}$ (95% CI)	1.0 (NE)	10.4 (8.2, 13.3)	13.2 (9.9, 17.6)	2.8 (2.2, 3.6)	1.9 (1.7, 2.0)
	GMFR (95% CI)	2.7 (2.1, 3.4)	5.6 (3.5, 8.8)	7.4 (4.9, 11.1)	4.7 (3.2, 6.9)	2.9 (2.2, 3.8)
	Seropositive (95% CI)	1.00 (0.86, 1.00)	1.00 (0.86, 1.00)	1.00 (0.86, 1.00)	1.00 (0.86, 1.00)	1.00 (0.86, 1.00)
Day 91	n	22	22	22	22	22
	GMT (95% CI)	6062 (4108, 8947)	462 (280, 761)	282 (148, 539)	1723 (1031, 2881)	2834 (1873, 4290)
	Median (Min, Max)	6500 (1772,80096)	382 (85,15039)	296 (20,4176)	2036 (241,29448)	2969 (838,43236)
	GMR $_{D614G}$ (95% CI)	1.0 (NE)	13.1 (10.0, 17.3)	21.5 (14.0, 32.8)	3.5 (2.8, 4.4)	2.1 (1.9, 2.4)
	GMFR (95% CI)	2.4 (1.9, 3.0)	3.7 (2.4, 5.7)	3.3 (2.2, 5.0)	3.2 (2.2, 4.7)	2.2 (1.7, 3.0)
	GMFD (95% CI)	1.2 (1.0, 1.5)	1.5 (1.3, 1.8)	2.0 (1.5, 2.8)	1.5 (1.2, 1.9)	1.4 (1.1, 1.8)
	Seropositive (95% CI)	1.00 (0.85, 1.00)	1.00 (0.85, 1.00)	0.86 (0.65, 0.97)	1.00 (0.85, 1.00)	1.00 (0.85, 1.00)
**Arm 14: 1 Dose Beta (B.1.351)**						
Day 1	n	31	31	31	31	31
	GMT (95% CI)	1587 (816, 3085)	107 (61, 189)	73 (45, 119)	355 (181, 695)	756 (411, 1394)
	Median (Min, Max)	2544 (20,20310)	155 (20,1417)	79 (20,1471)	452 (20,7622)	1217 (20,7234)
	GMR $_{D614G}$ (95% CI)	1.0 (NE)	14.8 (9.1, 24.1)	21.6 (13.0, 35.9)	4.5 (3.1, 6.4)	2.1 (1.8, 2.5)
	Seropositive (95% CI)	0.90 (0.74, 0.98)	0.58 (0.39, 0.75)	0.55 (0.36, 0.73)	0.77 (0.59, 0.90)	0.87 (0.70, 0.96)
Day 29	N	25	25	25	25	25
	GMT (95% CI)	9384 (5670, 15532)	1169 (597, 2289)	681 (375, 1237)	5173 (2796, 9572)	5670 (3311, 9709)
	Median (Min, Max)	7512 (921,141489)	1557 (20,24813)	777 (20,13413)	3330 (490,104984)	4572 (577,108622)
	GMR $_{D614G}$ (95% CI)	1.0 (NE)	8.0 (4.0, 16.1)	13.8 (6.6, 29.0)	1.8 (1.5, 2.2)	1.7 (1.5, 1.9)
	GMFR (95% CI)	7.0 (2.8, 17.5)	12.0 (5.3, 27.3)	9.2 (5.4, 15.5)	16.3 (6.8, 39.1)	8.9 (3.6, 22.0)

	Seropositive (95% CI)	1.00 (0.86, 1.00)	0.92 (0.74, 0.99)	0.92 (0.74, 0.99)	1.00 (0.86, 1.00)	1.00 (0.86, 1.00)
Day 91	n	21	21	21	21	21
	GMT (95% CI)	8078 (4874, 13387)	604 (318, 1146)	426 (231, 786)	3551 (1947, 6477)	4320 (2516, 7418)
	Median (Min, Max)	6410 (950,100232)	669 (20,3790)	423 (20,4032)	3324 (441,55411)	4034 (483,65385)
	GMR $_{D614G}$ (95% CI)	1.0 (NE)	13.4 (5.5, 32.5)	18.9 (8.1, 44.2)	2.3 (1.8, 2.9)	1.9 (1.7, 2.1)
	GMFR (95% CI)	4.8 (1.6, 14.4)	5.1 (2.6, 10.2)	4.9 (2.6, 9.0)	8.4 (3.0, 23.7)	5.4 (1.8, 15.9)
	GMFD (95% CI)	1.4 (1.1, 1.9)	2.1 (0.9, 5.1)	1.7 (1.5, 2.1)	1.9 (1.4, 2.7)	1.7 (1.2, 2.3)
	Seropositive (95% CI)	1.00 (0.84, 1.00)	0.90 (0.70, 0.99)	0.90 (0.70, 0.99)	1.00 (0.84, 1.00)	1.00 (0.84, 1.00)
**Arm 15: 1 Dose Beta (1.351)+Prototype**						
Day 1	n	32	32	32	32	32
	GMT (95% CI)	2839 (1798, 4483)	171 (97, 301)	106 (58, 195)	751 (421, 1339)	1436 (853, 2415)
	Median (Min, Max)	3728 (114,30099)	165 (20,3105)	102 (20,2091)	638 (20,14701)	2387 (20,19850)
	GMR $_{D614G}$ (95% CI)	1.0 (NE)	16.6 (12.2, 22.7)	26.7 (18.4, 38.8)	3.8 (3.0, 4.7)	2.0 (1.8, 2.2)
	Seropositive (95% CI)	1.00 (0.89, 1.00)	0.75 (0.57, 0.89)	0.56 (0.38, 0.74)	0.94 (0.79, 0.99)	0.97 (0.84, 1.00)
Day 29	n	30	30	30	30	30
	GMT (95% CI)	11726 (7890, 17428)	1391 (802, 2413)	1224 (762, 1964)	6785 (4369, 10537)	6996 (4750, 10304)
	Median (Min, Max)	13751 (1006,58467)	2137 (20,25964)	1487 (151,10863)	7217 (671,74317)	7587 (685,34901)
	GMR $_{D614G}$ (95% CI)	1.0 (NE)	8.4 (6.0, 11.8)	9.6 (7.6, 12.1)	1.7 (1.5, 2.0)	1.7 (1.6, 1.8)
	GMFR (95% CI)	4.0 (2.8, 5.6)	7.7 (4.6, 12.8)	10.3 (6.5, 16.4)	8.6 (5.2, 14.1)	4.7 (3.1, 7.2)
	Seropositive (95% CI)	1.00 (0.88, 1.00)	0.97 (0.83, 1.00)	1.00 (0.88, 1.00)	1.00 (0.88, 1.00)	1.00 (0.88, 1.00)
Day 91	n	25	25	25	25	25
	GMT (95% CI)	7914 (5087, 12311)	867 (428, 1754)	680 (343, 1349)	3665 (2094, 6418)	4382 (2750, 6983)
	Median (Min, Max)	11513 (1097,34915)	1044 (20,31076)	891 (20,12838)	3245 (296,40505)	5118 (374,23579)
	GMR $_{D614G}$ (95% CI)	1.0 (NE)	9.1 (6.0, 13.8)	11.6 (7.4, 18.2)	2.2 (1.7, 2.7)	1.8 (1.6, 2.0)
	GMFR (95% CI)	2.6 (1.7, 4.1)	4.5 (2.6, 7.9)	6.0 (3.4, 10.5)	4.3 (2.4, 7.7)	2.8 (1.6, 4.9)
	GMFD (95% CI)	1.6 (1.3, 2.0)	1.5 (0.9, 2.5)	1.5 (1.1, 2.0)	1.8 (1.3, 2.5)	1.6 (1.2, 2.1)
	Seropositive (95% CI)	1.00 (0.86, 1.00)	0.92 (0.74, 0.99)	0.92 (0.74, 0.99)	1.00 (0.86, 1.00)	1.00 (0.86, 1.00)
n=Number of subjects with results available at time point<br>Confidence intervals of the geometric means were calculated with the Student's t distribution on log-transformed data						

Table S11: Stage 3 (Sanofi) Pseudovirus Neutralization Assay Summary Results by Arm, Time Point and Variant for Previously Infected Participants

Time Point	Statistic	D614G	BA1	BA.4/5	B.1.351	B.1.617.2
**Arm 13: 1 Dose Prototype**						
Day 1	n	22	22	22	22	22
	GMT (95% CI)	13696 (8072, 23240)	2086 (1054, 4129)	1410 (785, 2533)	5912 (3003, 11638)	7160 (4025, 12735)
	Median (Min, Max)	17834 (456,77402)	2276 (20,33493)	1677 (20,13936)	8470 (20,41412)	8524 (196,78168)
	GMR $_{D614G}$ (95% CI)	1.0 (NE)	6.6 (4.8, 9.0)	9.7 (7.7, 12.2)	2.3 (1.6, 3.3)	1.9 (1.7, 2.2)
	Seropositive (95% CI)	1.00 (0.85, 1.00)	0.95 (0.77, 1.00)	0.95 (0.77, 1.00)	0.95 (0.77, 1.00)	1.00 (0.85, 1.00)
Day 29	n	21	21	21	21	21
	GMT (95% CI)	19141 (12996, 28192)	2756 (1241, 6120)	2389 (1242, 4596)	8448 (5205, 13712)	10781 (7354, 15806)
	Median (Min, Max)	19029 (4295,125062)	4309 (20,73349)	2656 (20,32164)	9427 (380,95151)	10573 (3252,68172)
	GMR $_{D614G}$ (95% CI)	1.0 (NE)	6.9 (3.7, 13.1)	8.0 (5.2, 12.4)	2.3 (1.7, 3.0)	1.8 (1.6, 2.0)
	GMFR (95% CI)	1.4 (1.0, 1.9)	1.3 (0.7, 2.6)	1.7 (1.2, 2.5)	1.5 (1.0, 2.1)	1.5 (1.0, 2.2)
	Seropositive (95% CI)	1.00 (0.84, 1.00)	0.95 (0.76, 1.00)	0.95 (0.76, 1.00)	1.00 (0.84, 1.00)	1.00 (0.84, 1.00)
Day 91	n	21	21	21	21	21
	GMT (95% CI)	17078 (11134, 26195)	2396 (1328, 4323)	1588 (832, 3030)	7972 (4689, 13554)	8400 (5169, 13652)
	Median (Min, Max)	20346 (2563,67272)	2502 (213,25327)	1704 (20,13871)	8683 (521,40445)	8786 (955,37319)
	GMR $_{D614G}$ (95% CI)	1.0 (NE)	7.1 (4.8, 10.5)	10.8 (7.2, 16.1)	2.1 (1.7, 2.7)	2.0 (1.8, 2.3)
	GMFR (95% CI)	1.2 (0.8, 2.0)	1.1 (0.6, 2.2)	1.1 (0.6, 2.3)	1.4 (0.7, 2.8)	1.2 (0.7, 2.0)
	GMFD (95% CI)	1.1 (0.8, 1.5)	1.2 (0.5, 2.5)	1.5 (0.8, 3.0)	1.1 (0.6, 1.8)	1.3 (0.9, 1.8)
	Seropositive (95% CI)	1.00 (0.84, 1.00)	1.00 (0.84, 1.00)	0.95 (0.76, 1.00)	1.00 (0.84, 1.00)	1.00 (0.84, 1.00)
**Arm 14: 1 Dose Beta (B.1.351)**						
Day 1	n	19	19	19	19	19
	GMT (95% CI)	11815 (6252, 22328)	1439 (522, 3965)	885 (340, 2302)	3595 (1351, 9569)	6648 (3257, 13568)

	Median (Min, Max)	13511 (463,99331)	2018 (20,48003)	1405 (20,13664)	4624 (20,51627)	7277 (226,65923)
	GMR $_{D614G}$ (95% CI)	1.0 (NE)	8.2 (4.9, 13.9)	13.3 (8.5, 20.9)	3.3 (1.9, 5.6)	1.8 (1.6, 2.0)
	Seropositive (95% CI)	1.00 (0.82, 1.00)	0.95 (0.74, 1.00)	0.84 (0.60, 0.97)	0.95 (0.74, 1.00)	1.00 (0.82, 1.00)
Day 29	n	18	18	18	18	18
	GMT (95% CI)	23922 (14351, 39876)	6302 (3809, 10425)	2716 (1301, 5669)	15773 (8345, 29814)	16500 (9484, 28707)
	Median (Min, Max)	19503 (2499,123066)	5779 (1060,32852)	3484 (20,16984)	15141 (914,136728)	14176 (1435,79376)
	GMR $_{D614G}$ (95% CI)	1.0 (NE)	3.8 (2.7, 5.4)	8.8 (5.8, 13.5)	1.5 (1.2, 1.9)	1.4 (1.3, 1.6)
	GMFR (95% CI)	2.0 (1.3, 3.2)	4.5 (2.2, 9.4)	3.2 (1.7, 6.1)	4.5 (2.3, 8.9)	2.5 (1.6, 3.9)
	Seropositive (95% CI)	1.00 (0.81, 1.00)	1.00 (0.81, 1.00)	0.94 (0.73, 1.00)	1.00 (0.81, 1.00)	1.00 (0.81, 1.00)
Day 91	n	18	18	18	18	18
	GMT (95% CI)	16501 (9873, 27581)	4254 (2337, 7744)	2163 (1317, 3553)	11453 (7132, 18394)	10337 (6200, 17232)
	Median (Min, Max)	16262 (3196,83723)	3515 (347,40416)	1858 (356,10066)	12506 (1489,52212)	12500 (1606,59269)
	GMR $_{D614G}$ (95% CI)	1.0 (NE)	3.9 (2.7, 5.6)	7.6 (5.6, 10.4)	1.4 (1.2, 1.8)	1.6 (1.4, 1.8)
	GMFR (95% CI)	1.4 (0.8, 2.4)	3.0 (1.5, 6.2)	2.5 (1.3, 4.9)	3.3 (1.4, 7.5)	1.6 (0.9, 2.7)
	GMFD (95% CI)	1.4 (1.0, 2.2)	1.5 (1.1, 1.9)	1.3 (0.7, 2.2)	1.4 (0.8, 2.4)	1.6 (1.0, 2.5)
	Seropositive (95% CI)	1.00 (0.81, 1.00)	1.00 (0.81, 1.00)	1.00 (0.81, 1.00)	1.00 (0.81, 1.00)	1.00 (0.81, 1.00)
**Arm 15: 1 Dose Beta (1.351)+Prototype**						
Day 1	n	20	20	20	20	20
	GMT (95% CI)	15252 (8677, 26808)	1243 (451, 3423)	1033 (427, 2503)	4988 (1854, 13415)	6034 (2558, 14235)
	Median (Min, Max)	15546 (1142,113184)	1844 (20,21366)	1077 (20,24266)	7087 (20,65808)	7287 (20,44079)
	GMR $_{D614G}$ (95% CI)	1.0 (NE)	12.3 (5.8, 25.9)	14.8 (8.2, 26.4)	3.1 (1.5, 6.4)	2.5 (1.3, 5.1)
	Seropositive (95% CI)	1.00 (0.83, 1.00)	0.85 (0.62, 0.97)	0.90 (0.68, 0.99)	0.95 (0.75, 1.00)	0.95 (0.75, 1.00)
Day 29	n	20	20	20	20	20
	GMT (95% CI)	19765 (11963, 32654)	2585 (1089, 6135)	1760 (812, 3814)	9961 (5034, 19708)	12004 (6978, 20648)
	Median (Min, Max)	20184 (3246,156596)	4264 (20,39155)	2931 (20,17731)	11829 (609,112669)	12437 (1787,102210)

	GMR $_{D614G}$ (95% CI)	1.0 (NE)	7.6 (3.8, 15.3)	11.2 (6.5, 19.5)	2.0 (1.5, 2.5)	1.6 (1.4, 1.9)
	GMFR (95% CI)	1.3 (1.0, 1.6)	2.1 (1.4, 3.0)	1.7 (1.2, 2.3)	2.0 (1.0, 3.9)	2.0 (1.0, 4.0)
	Seropositive (95% CI)	1.00 (0.83, 1.00)	0.95 (0.75, 1.00)	0.95 (0.75, 1.00)	1.00 (0.83, 1.00)	1.00 (0.83, 1.00)
Day 91	n	18	18	18	18	18
	GMT (95% CI)	16653 (10049, 27598)	1995 (723, 5503)	1626 (703, 3764)	8604 (4594, 16114)	8894 (5097, 15518)
	Median (Min, Max)	21543 (2410,65700)	3385 (20,45205)	2327 (20,23519)	11197 (535,42319)	10573 (1631,48274)
	GMR $_{D614G}$ (95% CI)	1.0 (NE)	8.3 (3.6, 19.2)	10.2 (5.2, 20.2)	1.9 (1.6, 2.4)	1.9 (1.6, 2.3)
	GMFR (95% CI)	0.9 (0.7, 1.2)	1.2 (0.9, 1.5)	1.2 (0.9, 1.6)	1.3 (0.5, 3.2)	1.2 (0.5, 3.0)
	GMFD (95% CI)	1.4 (1.1, 1.9)	1.6 (1.2, 2.2)	1.3 (1.1, 1.5)	1.5 (1.1, 2.0)	1.6 (1.2, 2.2)
	Seropositive (95% CI)	1.00 (0.81, 1.00)	0.89 (0.65, 0.99)	0.94 (0.73, 1.00)	1.00 (0.81, 1.00)	1.00 (0.81, 1.00)

n=Number of subjects with results available at time point.
Confidence intervals of the geometric means were calculated with the Student’s t distribution on log-transformed data

Table S12: Stage 1 (Moderna) Pseudovirus Neutralization Assay Summary Results by Arm, Time Point and Omicron Subvariant for a Subset of Uninfected Participants

Time Point	Statistic	D614G	BA.1	BA.2.12.1	BA.4/5	BA.2.75	BA.4.6	BF.7	BA.2.75.2	BQ.1.1	XBB.1
**Arm 1: 1 Dose Prototype Vaccine**											
Day 1	n	22	22	22	22	22	22	22	22	22	22
	GMT (95% CI)	428 (255, 719)	86 (52, 143)	66 (39, 110)	75 (45, 124)	ND	ND	ND	ND	ND	ND
	Median (Min , Max)	535 (99,6336)	88 (21,807)	76 (12,1118)	78 15,995)	ND	ND	ND	ND	ND	ND
	GMR $_{D614G}$ (95% CI)	1.00 (NE)	4.97 (3.38, 7.31)	6.51 (4.90, 8.64)	5.71 (4.41, 7.39)	ND	ND	ND	ND	ND	ND
	Seropositive (95%CI)	1.00 (0.85, 1.00)	1.00 (0.85, 1.00)	1.00 (0.85, 1.00)	1.00 (0.85, 1.00)	ND	ND	ND	ND	ND	ND
Day 15	N	22	22	22	22	22	22	22	22	22	22
	GMT (95% CI)	3248 (1948, 5414)	1488 (983, 2252)	929 (657, 1313)	541 (353, 829)	994 (641, 1541)	382 (255, 573)	332 (211, 523)	159 (105, 241)	115 (74, 179)	75 (53, 106)
	Median (Min,Max)	2949 (611,24523)	1125 249,12473)	839 (155,4895)	675 (93,3773)	814 (120,8802)	425 (68,1437)	431 (34,1987)	209 (24,512)	123 (23,604)	81 (23,306)
	GMR $_{D614G}$ (95% CI)	1.00 (NE)	2.18 (1.55, 3.07)	3.50 (2.50, 4.89)	6.00 (4.42, 8.15)	3.27 (2.33, 4.59)	8.50 (6.44,11.23)	9.79 7.14,13.41)	20.37 (14.81, 28.02)	28.20 (21.76, 36.56)	43.19 (30.75, 60.66)
	GMFR (95% CI)	7.6 (5.5, 10.5)	17.3 (11.9, 25.1)	14.1 (9.9, 20.1)	7.2 (5.3, 9.8)	0.0 (NE)	0.0 (NE)	0.0 (NE)	0.0 (NE)	0.0 (NE)	0.0 (NE)
	Seropositive (95% CI)	1.00 (0.85, 1.00)	1.00 (0.85, 1.00)	1.00 (0.85, 1.00)	1.00 (0.85, 1.00)	1.00 (0.85, 1.00)	1.00 (0.85,1.00)	1.00 (0.85, 1.00)	1.00 (0.85, 1.00)	1.00 (0.85, 1.00)	1.00 (0.85, 1.00)
Day 91	n	17	17	16	17	17	17	17	17	17	17
	GMT (95% CI)	1812 (1148, 2858)	722 (433, 1202)	312 (179, 545)	262 (158, 433)	484 (304, 769)	146 (87, 245)	103 (61,172)	58 (35, 95)	42 (24, 73)	29 (19, 45)
	Median (Min , Max)	1470 (527,8531)	734 (74,3362)	364 (28,2283)	233 (38,1093)	495 (77,1615)	141 (21,598)	95 (14,432)	94 (5,204)	35 (5,169)	32 (5,93)
	GMR $_{D614G}$ (95% CI)	1.00 (NE)	2.51 (1.83, 3.44)	6.02 (4.38, 8.29)	6.93 (5.01, 9.57)	3.75 (2.73, 5.14)	12.41 (8.63,17.83)	17.67 (12.34,25.2 9)	31.36 (21.06, 46.70)	43.13 (30.30, 61.41)	62.64 (45.02, 87.16)

	GMFR (95% CI)	4.4 (3.0, 6.4)	7.4 (5.2, 10.4)	4.8 (3.2, 7.1)	3.5 (2.5, 4.8)	0.0 (NE)	0.0 (NE)	0.0 (NE)	0.0 (NE)	0.0 (NE)	0.0 (NE)
	GMFD (95% CI)	2.0 (1.5, 2.6)	2.2 (1.7, 2.9)	3.2 (2.4, 4.2)	2.3 (1.9, 2.7)	2.2 (1.7, 2.9)	2.9 (2.3, 3.5)	3.7 (3.1, 4.5)	2.8 (2.2, 3.6)	2.8 (2.2, 3.5)	2.7 (2.1, 3.3)
	Seropositive (95% CI)	1.00 (0.80, 1.00)	1.00 (0.80, 1.00)	1.00 (0.79, 1.00)	1.00 (0.80, 1.00)	1.00 (0.80, 1.00)	1.00 (0.80, 1.00)	1.00 (0.80, 1.00)	0.94 (0.71, 1.00)	0.94 (0.71, 1.00)	0.88 (0.64, 0.99)
**Arm 6: 1 Dose Omicron (B.1.1.529)+Prototype**											
Day 1	n	23	23	23	23	23	23	23	23	23	23
	GMT (95% CI)	1014 (612, 1681)	132 (78, 222)	106 (63, 178)	114 (73, 177)	180 (102, 317)	ND	ND	ND	ND	ND
	Median (Min , Max)	1042 (98,6231)	114 (11,1188)	117 (5,849)	105 (12,668)	157 (16,1852)	ND	ND	ND	ND	ND
	GMR D614G (95%CI)	1.00 (NE)	7.69 (5.50, 10.75)	9.56 (6.54, 13.96)	8.91 (6.15, 2.91)	5.64 (4.02, 7.90)	ND	ND	ND	ND	ND
	Seropositive (95% CI)	1.00 (0.85, 1.00)	1.00 (0.85, 1.00)	0.96 (0.78, 1.00)	1.00 (0.85, 1.00)	1.00 (0.85, 1.00)	ND	ND	ND	ND	ND
Day 15	n	23	23	23	23	23	23	23	23	23	23
	GMT (95% CI)	3948 (2790,5587)	2125 (1444, 3127)	1502 (930, 2427)	716 (472, 1086)	1805 1207,2700)	734 (476, 1133)	563 (354, 895)	213 (142, 319)	194 (122, 307)	104 (70, 153)
	Median (Min , Max)	3890 (703,28360)	1886 203,12383)	2026 (40,14586)	739 (61,9913)	1700 (247,21398)	618 (77,10875)	528 (33,6286)	178 (58,4740)	182 (13,2080)	91 (14,979)
	GMR D614G (95%CI)	1.00 (NE)	1.86 (1.42, 2.43)	2.63 (1.90, 3.64)	5.51 (4.13, 7.36)	2.19 (1.59, 3.02)	5.38 (3.85, 7.51)	7.01 (4.94, 9.96)	18.55 (13.33, 25.81)	20.37 (14.76, 28.12)	38.10 (27.43, 52.92)
	GMFR (95% CI)	3.9 (2.6, 5.8)	16.1 (9.9, 26.1)	14.2 (9.3, 21.6)	6.3 (4.3, 9.2)	10.0 (6.9, 14.6)	8.1 (5.7, 11.5)	0.0 (NE)	0.0 NE)	0.0 (NE)	0.0 (NE)
	Seropositive (95% CI)	1.00 (0.85, 1.00)	1.00 (0.85, 1.00)	1.00 (0.85, 1.00)	1.00 (0.85, 1.00)	1.00 (0.85, 1.00)	1.00 (0.85, 1.00)	1.00 (0.85, 1.00)	1.00 (0.85, 1.00)	1.00 (0.85, 1.00)	1.00 0.85, 1.00)
Day 91	n	19	19	18	19	19	19	19	19	19	19
	GMT (95% CI)	3216 (2158, 4793)	1005 (543, 1861)	688 (401, 1181)	532 (305, 929)	904 (519, 1573)	428 (258, 712)	262 (156, 441)	100 (55, 183)	108 64, 183)	63 (36, 109)
	Median (Min , Max)	2752 (639,19735)	953 (123,44979)	610 (115,11179)	552 (113,15533)	818 (78,19344)	462 (72,4587)	283 (28,2741)	92 (20,2879)	114 (23,920)	56 (16,776)

	GMR $_{D614G}$ (95%CI)	1.00 (NE)	3.20 (2.01, 5.09)	4.62 (3.00, 7.13)	6.04 (3.97, 9.20)	3.56 (2.30, 5.50)	7.51 (4.91, 1.48)	12.26 (7.97, 18.84)	32.13 (20.46, 50.46)	29.71 (19.17, 46.04)	51.39 (32.06, 82.37)
	GMFR (95% CI)	3.0 (2.2, 4.0)	7.4 (4.4, 12.3)	6.3 (4.0, 10.1)	4.4 (2.9, 6.7)	4.4 (3.2, 6.2)	4.2 (3.0, 5.9)	0.0 (NE)	0.0 (NE)	0.0 (NE)	0.0 (NE)
	GMFD (95% CI)	1.3 (1.0, 1.7)	2.3 (1.6, 3.5)	2.4 (1.8, 3.4)	1.5 (1.1, 2.1)	2.1 (1.6, 2.8)	1.9 (1.5, 2.4)	2.5 (1.9, 3.3)	2.4 (1.8, 3.3)	2.1 (1.5, 2.9)	1.9 (1.4, 2.6)
	Seropositive (95% CI)	1.00 (0.82, 1.00)	1.00 (0.82, 1.00)	1.00 (0.81, 1.00)	1.00 (0.82, 1.00)	1.00 (0.82, 1.00)	1.00 (0.82, 1.00)	1.00 (0.82, 1.00)	1.00 (0.82, 1.00)	1.00 (0.82, 1.00)	1.00 (0.82, 1.00)

Phase 2 Clinical Trial to Optimize Immune Coverage of SARS-CoV-2 Existing and Emerging Variants

COVID-19 VAriant Immunologic Landscape Trial
(COVAIL Trial)

DMID Protocol Number: 22-0004

IND Sponsor: Division of Microbiology and Infectious Diseases (DMID)

Version Number: 4.0

12 May2022

STATEMENT OF COMPLIANCE

Each institution engaged in this research will hold a current Federal wide Assurance (FWA) issued by the Office of Human Research Protection (OHRP) for federally funded research. The Institutional Review Board (IRB)/Independent or Institutional Ethics Committee (IEC) must be registered with OHRP as applicable to the research.

The study will be carried out in accordance with the following as applicable:

- United States (US) Code of Federal Regulations (CFR) 45 CFR Part 46: Protection of Human Subjects
- Food and Drug Administration (FDA) Regulations: 21 CFR Part 50 (Protection of Human Subjects), 21 CFR Part 54 (Financial Disclosure by Clinical Investigators), 21 CFR Part 56 (IRBs), 21 CFR Part 11, and 21 CFR Part 312 (Investigational New Drug Application), and/or 21 CFR 812 (Investigational Device Exemptions)
- The International Council for Harmonisation (ICH) of Technical Requirements for Registration of Pharmaceuticals for Human Use (ICH) E6(R2) Good Clinical Practice (GCP), and the Belmont Report: Ethical Principles and Guidelines for the Protection of Human Subjects of Research, Report of the National Commission for the Protection of Human Subjects of Biomedical and Behavioral Research
- The policies and procedures of National Institutes of Health (NIH) Office of Extramural Research and Division of Microbiology and Infectious Diseases (DMID)
- The National Institute of Allergy and Infectious Diseases (NIAID) Terms of Award
- Any additional Federal, State, and Local Regulations and Guidance

The signature below provides the necessary assurance that this study will be conducted according to all stipulations of the protocol, including statements regarding confidentiality, and according to local legal and regulatory requirements, US federal regulations, and ICH E6(R2) GCP guidelines.

Site Investigator Signature:

Signed: ______________________________ Date: ______________

Name, Credentials

Title

TABLE OF CONTENTS

STATEMENT OF COMPLIANCE 2
TABLE OF CONTENTS 3
LIST OF TABLES 8
LIST OF FIGURES 8
1. PROTOCOL SUMMARY 9
1.1 Synopsis 9
1.2 Schedule of Activities (SOA) 15
2. INTRODUCTION 20
2.1 Background and Study Rationale 20
2.1.1 Public Readiness and Emergency Preparedness Act 23
2.2 Risk/Benefit Assessment 24
2.2.1 Known Potential Risks 24
2.2.2 Known Potential Benefits 28
3. OBJECTIVES AND ENDPOINTS 29
4. STUDY DESIGN 31
4.1 Overall Design 31
4.2 Scientific Rationale for Study Design 31
4.3 Justification for Doses 32
5. STUDY POPULATION 33
5.1 Inclusion Criteria 33
5.2 Exclusion Criteria 33
5.2.1 Exclusion of specific populations 33
5.3 Inclusion of Vulnerable Subjects 33
5.4 Lifestyle Considerations 33
5.5 Screen Failures 34
5.5.1 Strategies for Recruitment and Retention 34
5.5.2 Retention 34
5.5.3 Compensation Plan for Subjects 34

5.5.4 Costs ... 34
6. STUDY PRODUCT ... 35
6.1 Study Product(s) and Administration ... 35
6.1.1 Study Product Description ... 35
6.1.2 Dosing and Administration ... 36
6.1.3 Dose Modifications ... 37
6.2 Accountability/Handling/Storage/Preparation ... 37
6.2.1 Acquisition and Accountability ... 37
6.2.2 Formulation and Appearance ... 38
6.2.3 Product Storage and Stability ... 38
6.2.4 Preparation ... 39
6.3 Measures to Minimize Bias: Randomization and Blinding ... 39
6.3.1 Treatment Assignment Procedures ... 39
6.3.2 Randomization and Blinding ... 39
6.3.3 Blinding and Masking Procedures ... 39
6.4 Study Intervention Compliance ... 39
6.5 Concomitant Therapy ... 39
6.5.1 Rescue Medicine ... 40
6.5.2 Non-Research Standard of Care ... 40
7. STUDY INTERVENTION DISCONTINUATION AND SUBJECT DISCONTINUATION/WITHDRAWAL ... 41
7.1 Halting Criteria and Discontinuation of Study Intervention ... 41
7.1.1 Halting Criteria ... 41
7.1.2 Criteria for Continuation of Dosing and Redosing ... 41
7.1.3 Discontinuation of Study Intervention ... 41
7.1.3.1 Delay of Study Vaccination ... 42
7.1.4 Follow-up for Subjects that Discontinued Study Intervention ... 42
7.1.5 Follow up for Subjects with a Suspicion for Myocarditis and/or Pericarditis ... 42
7.1.5.1 Probable Case of Acute Myocarditis ... 43
7.1.5.2 Confirmed Case of Acute Myocarditis ... 43
7.1.5.3 Acute Pericarditis Case Definition ... 44

7.1.5.4 Myopericarditis Case Definition 44
7.2 Subject Withdrawal from the Study and Replacement 44
7.3 Lost to Follow-Up 45
8. STUDY ASSESSMENTS AND PROCEDURES 46
8.1 Screening and Immunogenicity Assessments 46
8.1.1 Screening or Enrollment/Baseline Procedures 46
8.1.2 Immunogenicity Evaluations 47
8.1.3 Samples for Suspected SARS-CoV-2 infection 48
8.1.4 Samples for Genetic/Genomic Analysis 48
8.1.4.1 Genetic/Genomic Analysis 48
8.1.4.2 Genetic Privacy and Confidentiality 48
8.1.4.3 Management of Results 48
8.1.5 SARS-CoV-2 infection 48
8.1.6. Retained Samples for Biomarker Testing 49
8.2 Safety and Other Assessments 49
8.3 Adverse Events and Serious Adverse Events 49
8.3.1 Definition of Adverse Event (AE) 49
8.3.1.1 Solicited Adverse Events 50
8.3.1.2 Unsolicited Adverse Events 51
8.3.2 Definition of Serious Adverse Event (SAE) 51
8.3.3 Suspected Unexpected Serious Adverse Reactions (SUSAR) 51
8.3.4 Classification of an Adverse Event 52
8.3.4.1 Severity of Adverse Events 52
8.3.4.2 Relationship to Study Intervention 52
8.3.5 Time Period and Frequency for Event Assessment and Follow-Up 53
8.3.6 Adverse Event Reporting 53
8.3.6.1 Investigators Reporting of AEs 53
8.3.7 Serious Adverse Event Reporting 53
8.3.7.1 Investigators Reporting of SAEs 53
8.3.7.2 Regulatory Reporting of SAEs 54
8.3.8 Reporting Events to Subjects 54

8.3.9 Adverse Events of Special Interest (AESIs), New Onset of Chronic Medical Conditions (NOCMCs) and Medically Attended Adverse Events (MAAEs) 54
8.3.10 Reporting of Pregnancy 55
8.4 Unanticipated Problems 55
8.4.1 Definition of Unanticipated Problems (UPs) 55
8.4.2 Unanticipated Problem Reporting 56
8.4.3 Reporting Unanticipated Problems to Subjects 56
9. STATISTICAL CONSIDERATIONS 57
9.1 Statistical Hypotheses 57
9.2 Sample Size Determination 57
9.2.1 Sample Size Calculation for the Safety Endpoint 57
9.2.2 Sample Size Calculation for the Immunogenicity Endpoints 58
9.3 Populations for Analyses 59
9.4 Statistical Analyses 59
9.4.1 General Approach 59
9.4.2 Analysis of the Primary Endpoint(s) 60
9.4.3 Analysis of the Secondary Endpoints 60
9.4.4 Safety Analyses 60
9.4.5 Baseline Descriptive Statistics 60
9.4.6 Planned Interim and Early Analyses 60
9.4.6.1 Interim Safety Analyses 61
9.4.6.2 Interim Immunogenicity Review 61
9.4.6.3 Interim Immunogenicity and Safety Review 61
9.4.7 Sub-Group Analyses 61
9.4.8 Tabulation of Individual Subject Data 61
9.4.9 Exploratory Analyses 61
10. SUPPORTING DOCUMENTATION AND OPERATIONAL CONSIDERATIONS 62
10.1 Regulatory, Ethical, and Study Oversight Considerations 62
10.1.1 Informed Consent Process 62
10.1.1.1 Requirements for Permission by Parents/Guardians and Assent by Children (in case of a minor) 63

10.1.1.2 Other Informed Consent Procedures 63
10.1.2 Study Termination and Closure 65
10.1.3 Confidentiality and Privacy 65
10.1.4 Secondary Use of Stored Specimens and Data 66
10.1.4.1 Samples for Secondary Research 67
10.1.4.2 Data Sharing for Secondary Research 67
10.1.5 Key Roles and Study Governance 67
10.1.6 Safety Oversight 68
10.1.6.1 Data Safety Monitoring Board (DSMB) 68
10.1.7 Clinical Monitoring 68
10.1.8 Quality Control (QC) and Quality Assurance (QA) 68
10.1.9 Data Handling and Record Keeping 69
10.1.9.1 Data Collection and Management Responsibilities 69
10.1.9.2 Study Record Retention 69
10.1.9.3 Source Records 70
10.1.9.4 Protocol Deviations 70
10.1.9.5 Data Sharing Plan 70
10.1.9.6 Genomic Data Sharing (GDS) Plan 70
10.1.9.7 Publication 71
10.1.9.8 Conflict of Interest Policy 71
10.2 Additional Considerations 71
10.2.1 Research Related Injuries 71
10.3 Abbreviations 71
10.4 Protocol Amendment History 77
11. REFERENCES 82
12. APPENDIX A: Adverse Events of Special Interest (AESIs) Terms 87

LIST OF TABLES

Table 1: Study Arms 11
Table 2: SOA for single dose 15
Table 3: SOA for 2 doses 17
Table 4: Objectives and Endpoints (Outcome Measures) 29
Table 5: Probability of Observing one or more Adverse Event for Various Event Rates in one vaccine group (or strata) 57
Table 6: Two-sided 95% confidence intervals based on observing a particular average $\log_e$-antibody titer in subjects' vaccine groups and potential strata sizes 58
Table 7: Abbreviations 71

LIST OF FIGURES

No table of figures entries found.

1. PROTOCOL SUMMARY

1.1 Synopsis

Title: Phase 2 Clinical Trial to Optimize Immune Coverage of SARS-CoV-2 Existing and Emerging Variants

Short title: COVID-19 Adaptive VAriant Immunologic Landscape Trial (COVAIL Trial)

Phase: Phase 2

Population: Approximately 1,500 healthy individuals aged ≥ 18 years

Sites: Up to 30 US clinical research sites

Rationale:

The Severe Acute Respiratory Syndrome Coronavirus 2 (SARS-CoV-2), causative agent of the coronavirus disease of 2019 (COVID-19) pandemic, has infected over 500 million people worldwide and resulted in more than 6 million deaths, including close to 1 million deaths in the United States (April 18, 2022 WHO; www.who.int). As of April 2022, there are three approved or authorized vaccines in the U.S.: two using mRNA-based technology (Pfizer BioNTech [BNT162b2] and Moderna [mRNA-1273]), and one using a non-replicating viral vector vaccine (Johnson and Johnson adenovirus Ad26), all encoding the spike protein of the ancestral strain. A fourth manufacturer, Novavax, has received World Health Organization (WHO) pre-qualification for their adjuvanted protein formulation, and has filed for Emergency Use Authorization in the United States; a fifth manufacturer Sanofi Pasteur has another adjuvanted protein vaccine that showed efficacy in a phase 3 trial. These and other WHO authorized vaccines have been an essential countermeasure against the pandemic.

However, the emergence of many variants of concern with different degrees of immune escape challenge the effectiveness of these vaccines. The persistent evolution of the virus leads to the consideration of adapting vaccines to antigenically match circulating variants as well as expanding and optimizing immune coverage to existing and emerging antigenic variants. Current efforts are directed towards modifying the existing vaccines to include the current circulating variant Omicron. However, Omicron may not be the circulating variant during the next wave of COVID-19. It is important to look beyond Omicron and understand the immunologic landscape and how we can use prototype and variant vaccines alone or in combination to address emerging variants.

This phase 2 clinical trial will evaluate the safety and immunogenicity of additional doses of prototype and variant (alone or in combination) vaccine candidates in previously vaccinated participants with or without prior SARS-CoV-2 infection, and will evaluate innate, cellular, and humoral immune responses to inform on how to shift the immune response to cover new variants as they emerge.

Objectives:

Primary:

1. To evaluate humoral immune responses of candidate SARS-CoV-2 variant vaccines, alone or in combination.

Secondary

1. To evaluate the safety of candidate SARS-CoV-2 variant vaccines, as assessed by:
   - Local and systemic solicited Adverse Events for 7 days following each vaccine dose.
   - Unsolicited Adverse Events from Dose 1 to 28 days following each vaccine dose.
   - SAEs, MAAEs, AESIs, NOCMCs, and AEs leading to withdrawal from the study from Dose 1 to 12 months after last vaccine dose.

Exploratory:

1. To assess, in at least a subset of samples, the B cell immune response of candidate SARS-CoV-2 variant vaccines.
2. To assess, in at least a subset of samples, the SARS-CoV-2 spike protein-specific T cell responses of candidate SARS-CoV-2 variant vaccines.
3. To assess, in at least a subset of samples, the magnitude, phenotype, and percentage of innate immune cells with candidate SARS-CoV-2 variant vaccines.
4. To assess, in at least a subset of samples, the functional potential of SARS-CoV-2 specific antibodies to mediate Fc-effector functions across candidate SARS-CoV-2 variant vaccines.
5. To evaluate breakthrough SARS-CoV-2 infection by sequencing strains for variant spike lineage and assessing anti- nucleocapsid serology.

Study Design:

This is a Phase 2 randomized open label, non-placebo controlled, multi-site, multi-stage clinical trial in individuals, 18 years of age and older, who are in stable state of health, have received a complete authorized/approved vaccine series (primary series + booster either with homologous or heterologous vaccine products) ≥ 16 weeks prior to enrollment.

Subjects will be stratified by i) age (18-64 years and ≥ 65 years of age) and ii) history of confirmed prior SARS-CoV-2 infection, and randomly assigned to receive one of several variant vaccines. Enrollment will target a goal of approximately 45% of each of the variant vaccine arms to be in older adults (≥ 65 years of age) and approximately 20% to have had confirmed COVID-19.

This is an adaptive design and may add arms of new vaccine platforms and/or variant lineage spike vaccines as needed. The study arms will be conducted in different stages (that could overlap) depending on public health needs and availability of study products (starting with the available mRNA vaccines).

Table 1: Study Arms

	Arms	Vaccine Platform	Sample Size	Vaccine Candidate	Interval (weeks)*	Timing of First Dose	Timing of Second Dose
Stage 1	1	Moderna mRNA-1273	100	Prototype	≥16	D1	NA
	2		100	Beta + Omicron	≥16	D1	NA
	3		100	Beta + Omicron	≥16	D1	D57
	4		100	Delta + Omicron	≥16	D1	NA
	5		100	Omicron	≥16	D1	NA
	6		100	Omicron + Prototype	≥16	D1	NA
Stage 2	7	Pfizer/ BioNTech BNT162b2	50	Wildtype (Prototype)	≥16	D1	NA
	8		50	Beta + Omicron	≥16	D1	NA
	9		50	Omicron	≥16	D1	NA
	10		50	Beta	≥16	D1	NA
	11		50	Beta + Wildtype	≥16	D1	NA
	12		50	Omicron + Wildtype	≥16	D1	NA
Stage 3	13	Sanofi CoV2 preS dTM-AS03	50	Prototype	≥16	D1	NA
	14		50	Beta	≥16	D1	NA
	15		50	Beta+ Prototype	≥16	D1	NA

Two age strata:

- 18-64 years
- ≥ 65 years (~45% in ≥ 65 years).

Two infection strata:

- Confirmed prior SARS-CoV-2 infection (~20%)
- No known history of prior infection.

*interval (in weeks) since last exposure to SARS-CoV-2 infection or vaccination

Duration of Study: Up to 4 years

Duration of participation per subject: Up to 2 years (approximately 12 months after last inoculation)

Criteria for Inclusion/Exclusion:

Inclusion Criteria:

Participants must meet all of the following criteria to be eligible to participate in this study:

1. Individuals ≥ 18 years of age at the time of consent.
2. Confirmed receipt of a complete primary and booster COVID-19 vaccine series, either homologous or heterologous, with an FDA authorized/approved vaccine at least 16 weeks prior to study vaccine dose 1.
3. Willing and able to comply with all scheduled visits, vaccination plan, laboratory tests and other study procedures.
4. Determined by medical history, targeted physical examination and clinical judgement of the investigator to be in stable state of health.

   Note: Participants with pre-existing stable chronic medical conditions defined as condition not requiring significant change in therapy or hospitalization for worsening disease within 4 weeks from enrollment, can be included at the discretion of the investigator.

Exclusion Criteria:

Participants meeting any of the following criteria will be excluded from the study:

1. Confirmed SARS-CoV-2 infection < 16 weeks prior to any study vaccine dose.
2. Pregnant and breastfeeding participants.
3. Prior administration of an investigational coronavirus vaccine at any time or SARS-CoV-2 immunoglobulin, monoclonal antibody or plasma antibody therapy in the preceding 3 months.

   Note: subjects that participated in clinical trials of products that are now FDA approved/authorized are allowed to participate.
4. Current/planned simultaneous participation in another interventional study or receipt of any investigational study product within 28 days prior to vaccine study dose(s).
5. A history of anaphylaxis, urticaria, or other significant adverse reaction requiring medical intervention after receipt of a vaccine, polyethylene glycol (PEG), polysorbate or nanolipid particles.

6. A history of myocarditis or pericarditis at any time prior to enrollment (for subjects in stages 1 and 2).
7. Received or plans to receive a vaccine within 28 days prior to or after any dose of study vaccine.

   Note: Receipt of seasonal influenza vaccine is allowed at any time.
8. Bleeding disorder diagnosed by a healthcare provider (e.g., factor deficiency, coagulopathy, or platelet disorder requiring special precautions) or bleeding difficulties with intramuscular injections or blood draws.
9. Current or previous diagnosis of an immunocompromising condition or other immunosuppressive condition.
10. Advanced liver or kidney diseases.
11. Advanced (CD4 count < 200) and/or untreated HIV, untreated Hepatitis B or untreated Hepatitis C.
12. Received oral, intramuscular or intravenous systemic immunosuppressants, or immune-modifying drugs for >14 days in total within 6 months prior to any study vaccine dose (for corticosteroids ≥ 20 mg/day of prednisone equivalent).

    Note: Topical medications are allowed.
13. Received immunoglobulin or blood-derived products, within 3 months prior any study vaccine dose.
14. Received chemotherapy, immunotherapy or radiation therapy within 6 months prior to any study vaccine dose.
15. Study personnel or an immediate family member or household member of study personnel.
16. Is acutely ill or febrile 72 hours prior to or at vaccine dosing (fever defined as ≥ 38.0°C/100.4°F). Participants meeting this criterion may be rescheduled within the relevant window periods.

    Note: Afebrile participants with minor illnesses can be enrolled at the discretion of the Investigator, as long as the illness is not suggestive of COVID-19.

Safety

- Solicited Adverse Events will be collected for 7 days following each vaccine dose.
- Unsolicited Adverse Events will be collected from Dose 1 to 28 days following each vaccine dose.
- SAEs, MAAEs, AESIs, NOCMCs, and AEs leading to withdrawal from the study will be collected from Dose 1 to 12 months after last vaccine dose.

- The study will use halting rules for vaccination. See Section 7.1 for details.

1.2 Schedule of Activities (SOA)

Table 2: SOA for single dose

Study Day	D-28 to D-1	1	4	8[b]	15	29	91	181	271	366	Illness/ Unscheduled Visit[k]	Early Termination Visit
**Visit Number**	**00[a]**	**1**	**1A**	**2**	**3**	**4**	**5**	**6**	**7**	**8**		
**Window (+/-)**		**0**	**1**	**1**	**2**	**2**	**7**	**14**	**14**	**28**		
Informed Consent[a]	X											
Eligibility Criteria	X											
Medical History	X											
Vaccination		X										
Concomitant Meds	X	X	X[j]	X[j]	X[j]	X[j]	X[j]	X[j]	X[j]	X[j]	X[j]	X[j]
Interim History		X	X	X	X	X	X	X	X	X	X	X
Physical Exam[c] - Targeted	X	X									(X)	
Vital Signs[c]	X	X									(X)	
Height/Weight (BMI)[a]	X											
Urine β-HCG[d]	X	X										
Memory Aid[e]		X	X	X								
Solicited AEs		X	X	X								
Unsolicited AEs		X	X	X	X	X						
MAAEs, SAEs, NOCMCs, AEs leading to withdrawal from the study and AESIs		X	X	X	X	X	X	X	X	X	X	X
Nasal swab for PCR & Sequencing[f]											(X)	
**Assays**												
Serum for Serological Immunogenicity Assays[g] and Biomarker Testing[l]		34[l]	4[l]		34	34	34	34	34	34	4[l]	34
PBMC Cellular Assays & Plasma[g,h]		64-70			64-70		64-70	64-70	64-70			64-70

(select sites only)												
Daily Volume (mL)[g,i]		98-104	4		98-104	34	98-104	98-104	98-104	34		98-104
Cumulative Volume (mL) over 56 day-period[g,i]		98-104	102-108		200-212	234-246	132-138	98-104	98-104	132-138		
Total Cumulative Volume (mL)[g,i]		98-104	102-108		200-212	234-246	332-350	430-454	528-558	562-592		

[a] Optional screening visit – informed consent and screening activities can be a separate visit or combined with Day 1.

[b] Telephone visit

[c] Vital signs and targeted physical exam will be performed on screening and before vaccination otherwise, only as clinically indicated or based on interim medical history.

[d] For subjects of childbearing potential, a urine pregnancy will be performed at screening. If enrollment occurs on a separate day, a repeat urine pregnancy test will be done within 24 hours of study vaccine administration and negative results confirmed prior to dosing.

[e] The memory aid will be distributed at the Day 1 visit, queried for completion at the Day 8 telephone call and reviewed during an interview with the subject at the D15 visit and confirmed data recorded on the DCF.

[f]Collect nasal swab for PCR (1-2). Swabs can be self-collected and visit done by phone. Sequencing will be performed on all Illness visit-confirmed SARS-CoV-2 specimens.

[g] Inability (e.g., failure of venipuncture) to collect all baseline samples on Day 1 will not exclude the subject from further participation in this study as long as a minimum of baseline blood volume is collected (refer to the MOP for the protocol defined minimum number of required aliquots). Blood volume for PBMC varies depending on the type of tubes used for blood collection (refer to the MOP).

[h] Selected sites will perform PBMC separation on selected subjects (refer to MOP for more guidance).

[i] Blood volumes for early termination visit are not included in the blood volume totals.

[j] After vaccination on Day 1, immunosuppressant drugs, COVID-19 related prophylaxis and therapies as well as vaccines will be recorded. For any SAE occurring at any time during the study, all medications will be recorded.

[k] If an unscheduled visit is performed to evaluate an adverse event, vital signs and targeted physical exam will be performed, as indicated. If, in the judgement of the site investigator, the event, occurring within 4 weeks after vaccination, is a case of suspected myocarditis and/or pericarditis, the site will coordinate an appropriate diagnostic workup to make a determination of probable or confirmed myocarditis and/or pericarditis which may include, but is not limited to, an ECG, cardiac troponin testing and referral to a cardiologist (directly, or through the emergency department or primary care clinic). The suspected myocarditis and/or pericarditis case should be reported to the DMID Medical Monitor within 24 hours of site awareness.

[l]Serum for biomarker testing will be collected on Day 4 and stored for potential biomarker testing at a central laboratory and compared to testing of an aliquot from serum obtained on Day 1. If an unscheduled visit is related to a concern for myocarditis and/or pericarditis within 4 weeks after vaccination, a 4 mL SST will be collected and stored for potential biomarker testing at a central laboratory.

Table 3: SOA for 2 doses

Study Day	D-28 to D-1	1	4	8[b]	15	29	57	60	64[b]	71	85	147	237	327	422	Illness/ Unscheduled Visit[k]	Early Termination Visit
**Visit Number**	00[a]	1	1A	2	3	4	5	5A	6	7	8	9	10	11	12		
**Window (+/-)**		0	1	1	2	2	3	1	1	2	2	7	14	14	28		
Informed Consent[a]	X																
Eligibility Criteria	X						X										
Medical History	X						X										
Vaccination		X					X										
Concomitant Meds	X	X	X[j]	X[j]	X[j]	X[j]	X[j]	X[j]	X[j]	X[j]	X[j]	X[j]	X[j]	X[j]	X[j]	X[j]	X[j]
Interim History		X	X	X	X	X	X	X	X	X	X	X	X	X	X	X	X
Physical Exam[c] – Targeted	X	X					X									(X)	
Vital Signs[c]	X	X					X									(X)	
Height/Weight (BMI)[a]	X																
Urine β-HCG[d]	X	X					X										
Memory Aid[e]		X	X	X			X	X	X								
Solicited AEs		X	X	X			X	X	X								
Unsolicited AEs		X	X	X	X	X	X	X	X	X	X						
MAAEs, SAEs, NOCMCs, AEs leading to withdrawal from the study and AESIs		X	X	X	X	X	X	X	X	X	X	X	X	X	X	X	X
Nasal swab for PCR & Sequencing[f]																(X)	
**Assays**																	

Serum for Serological Immunogenicity Assays[g] and Biomarker Testing[l]		34[l]	4[l]		34	34	34[l]	4[l]		34	34	34	34	34	34	4[l]	34
PBMC Cellular Assays & Plasma[g,h] *(select sites only)*		64-70			64-70		64-70			64-70		64-70	64-70	64-70			64-70
Daily Volume (mL)[g,i]		98-104	4		98-104	34	98-104	4		98-104	34	98-104	98-104	98-104	34		98-104
Cumulative Volume (mL) over 56 day-period[g,i]		98-104	102-108		200-212	234-246	332-350	238-250		336-354	268-280	132-138	98-104	98-104	132-138		
Total Cumulative Volume (mL)[g,i]		98-104	102-108		200-212	234-246	332-350	336-354		434-458	468-492	566-596	664-700	762-804	796-838		

[a] Optional screening visit – informed consent and screening activities can be a separate visit or combined with Day 1.
[b] Telephone visit
[c] Vital signs and targeted physical exam will be performed on screening and before vaccination otherwise, only as clinically indicated or based on interim medical history.
[d] For subjects of childbearing potential, a urine pregnancy test will be performed at screening. If enrollment occurs on a separate day as screening, a repeat urine pregnancy test will be done within 24 hours of first study vaccine administration and repeated on day 57 prior to second study vaccine administration. Negative results must be confirmed prior to each dosing.
[e] The memory aid will be distributed at the Day 1 and Day 57 visits, queried for completion at the Day 8 and Day 64 telephone calls and reviewed during an interview with the subjectat the D15 and Day 71 visits and confirmed data recorded on the DCF.
[f] Collect nasal swab for PCR (1-2). Swabs can be self-collected and visit done by phone. Sequencing will be performed on all Illness visit-confirmed SARS-CoV-2 specimens.
[g] Inability (e.g., failure of venipuncture) to collect all baseline samples on Day 1 will not exclude the subject from further participation in this study as long as a minimum of baseline blood volume is collected (refer to the MOP for the protocol defined minimum number of required aliquots). Blood volume for PBMC varies depending on the type of tubes used for the blood collection (refer to the MOP).
[h] Selected sites will perform PBMC separation on selected subjects (refer to MOP for more guidance).
[i] Blood volumes for early termination visit are not included in the blood volume totals.
[j] After vaccination on Day 1, immunosuppressant drugs, COVID-19 related prophylaxis and therapies as well as vaccines will be recorded. For any SAE occurring at any time during the study, all medications will be recorded.

[k] If an unscheduled visit is performed to evaluate an adverse event, vital signs and targeted physical exam will be performed, as indicated. If, in the judgement of the site investigator, the event, occurring within 4 weeks after each vaccination, is a case of suspected myocarditis and/or pericarditis, the site will coordinate an appropriate diagnostic workup to make a determination of probable or confirmed myocarditis and/or pericarditis which may include, but is not limited to, an ECG, cardiac troponin testing and referral to a cardiologist (directly, or through the emergency department or primary care clinic). The suspected myocarditis and/or pericarditis case should be reported to the DMID Medical Monitor within 24 hours of site awareness.
[l] Serum for biomarker testing will be collected on Days 4 and 60 and stored for potential biomarker testing at a central laboratory and compared to testing of an aliquot of serum obtained on Days 1 and 57, respectively. If an unscheduled visit is related to a concern for myocarditis and/or pericarditis within 4 weeks after vaccination, a 4 mL SST will be collected and stored for potential biomarker testing at a central laboratory.

Note Windows for subsequent visits should be based off the preceding vaccination visit.

If intercurrent SARS- CoV-2 infection occurs between the first and prior to the planned second study dose or if a COVID-19 vaccine is given outside the study between the first and prior to the planned second study dose, no second vaccination will be given. Participants who do not receive a second dose will revert to the schedule of activities for single dose participants.

2. INTRODUCTION

2.1 Background and Study Rationale

The Severe Acute Respiratory Syndrome Coronavirus 2 (SARS-CoV-2) was first detected in Wuhan, Hubei Province, China in December 2019. The corresponding illness designation, coronavirus disease 2019 (COVID-19), was declared as a pandemic respiratory illness in March 2020.[1] As of 18 April 2022, it has infected over 500 million people worldwide and resulted in more than 6 million deaths, including close to 1 million in the United States.[1,2]

Six Phase 3 efficacy trials of SARS-CoV-2 vaccine constructs were conducted and are in long-term follow-up in the U.S. The ModernaTX, Inc mRNA-1273 and Pfizer/BioNTech BNT162b2 mRNA platforms encode for the full-length spike (S) protein of SARS-CoV-2, modified to introduce 2 proline residues to stabilize the S protein (S-2P) in a prefusion conformation, derived from the Wuhan-Hu-1 strain (prototype).[3] The Janssen Pharmaceutical/Johnson & Johnson COVID-19 Vaccine (Ad26.COV.2) is composed of recombinant, replication-incompetent human adenovirus type 26, encoding a prefusion-stabilized SARS-CoV-2 spike antigen.[4] Studies of the Pfizer and Moderna mRNA vaccines demonstrated high efficacy against all symptomatic and severe disease and received Emergency Use Authorization (EUA) on December 12 and 18, 2020, respectively and full FDA approval on August 23, 2021 and January 31, 2022. Similarly, Janssen Pharmaceuticals reported 66% vaccine efficacy with a single dose and high-level protection against severe disease and death. FDA EUA was issued on February 26, 2021. AstraZeneca/Oxford's ChAdOx-2, a recombinant, replication-incompetent chimpanzee adenovirus vector that expresses the spike protein, Novavax's NVX-CoV2373, a recombinant nanoparticle vaccine containing the full-length spike protein, and Sanofi/GSK's adjuvanted recombinant spike protein vaccine are expected to undergo FDA review for EUA shortly.[5-7]

Despite widespread vaccine distribution and availability, and high coverage among adults in the United States (87.4% of the US population ≥ 18 years of age have received at least one dose)[8], ongoing waves of the pandemic continue to cause significant disease burden, notable for increasing incidence of breakthrough infections in fully vaccinated individuals. Studies have suggested that vaccine protection against symptomatic SARS-CoV-2 infection wanes over time though protection against severe COVID-19 outcomes remains relatively intact.[9-12] However, the evolution of variant strains may favor immune escape or reinfection among previously infected or vaccinated individuals. A variant first identified in late 2020 (B.1.351), is associated with increased transmission, higher viral burden, and possibly increased mortality in infected persons.[13] Another variant identified in December 2020 (B.1.617.2) is associated with increased transmission, higher risk of severe disease and hospitalization than the ancestral strain.[14] B.1.617.2 was the dominant strain worldwide before being replaced by B.1.1.529 in November 2021 which is characterized by a replication advantage, higher re-infection rates and evasion of humoral immunity mediated by more than 30 mutations in the spike protein.[15]

Recent data suggest that a booster dose given as either a third dose for most vaccines (mRNA, protein and ChAdOx-2) or a second dose for Janssen improved vaccine effectiveness against new variants in the short term.[16-18] Data on immunogenicity with booster doses of BNT162b2, mRNA-

1273, and AD26.COV2.S demonstrated higher antibody titers to D614G after either homologous and heterologous prime-boost combinations.[19] The result is better neutralizing activity against B.1.351, B.1.617.2 and B.1.1.529.[20-22] In an open label phase study of participants who received a single 50 mcg booster dose of mRNA-1273 ≥ 6 months after a primary series, a 13 fold rise in antibody titers against D614G were noted from pre-boost levels as well as a 17-fold rise in in titers against the B.1.617.2 variant.[20] In another study, participants boosted with 50 mcg of mRNA-1273 demonstrated ID_{50} GMTs against the omicron variant 20 times higher than those described 1 month after the second vaccination of the primary series; these titers were 2.9 times lower than titers for the D614G variant.[21]

The Sanofi/GSK adjuvanted recombinant prefusion spike protein vaccine (CoV2 preS dTM-AS03 [D614]) was developed from an established, safe platform used for influenza vaccines. A phase 2 study demonstrated the safety and immunogenicity of a two-dose primary series of CoV2 preS dTM-AS03 [D614] in adults who were SARS-CoV-2 naïve and those who were previously infected.[23] The dose-finding results informed selection of the 10 mcg primary dose and 5 mcg boost dose for further evaluation. Neutralizing antibody titers were higher in non-naïve participants after one dose than in naïve participants after two doses. In addition, 5 mcg booster dose increased neutralizing antibody titers when used as a heterologous (18-30 times) or homologous (≥ 84 times) boost.[24] Interim Results from an international phase 3 trial showed 57.9% (95% confidence interval 26.5% to 76.7%) efficacy of the two-dose primary series against any symptomatic disease and 75% efficacy against moderate or severe COVID-19 with a good safety profile.[24]

FDA EUA was subsequently issued for a booster 30 ug dose of Pfizer BNT162b2 on September 24, 2021, 50 ug booster dose of Moderna mRNA-1273 on October 21, 2021, and a 5×10^{10} viral particles booster dose of J&J AD26.COV2 on October 21, 2021. This was recommended for adults 18 years of age and older who received a FDA authorized and CDC recommended primary booster series with an mRNA vaccine at least 5 months prior or a primary series with AD26.COV2 at least 2 months prior. The Sanofi/GSK vaccine is currently undergoing regulatory review for approval of their vaccine candidate for the prevention of COVID-19.

Published reports have also provided some early evidence for the safety and immunogenicity of a fourth dose of an mRNA vaccine. An open-label study of healthcare workers in Israel suggests that a fourth dose was well-tolerated and restored neutralizing antibody titers for variants to levels similar to those after a third dose.[25] Notably, this study did not include older adults, and rates of infection with B.1.1.529 (omicron) were similar for the control and Pfizer or Moderna 4th dose groups. In a retrospective study conducted by the Israeli Ministry of Health, of 1,138,681 adults aged 60 to 100 years of age, a fourth dose of mRNA was associated with 2 times lower rate of confirmed infection 12 or more days after vaccination compared to those who had only received 3 doses and the rate of severe illness was lower by a factor of 4.3.[26] However, the added protection of an additional booster shot is small in absolute terms since less than 0.1% mortality was noted after a 3rd dose. Additionally, a separate study of adults age 60 or older suggests that vaccine effectiveness of a fourth dose against infection wanes quickly declining from a peak of 64% to 29% within 2 months though effectiveness of >73% was maintained against severe disease.[27]

On March 29, 2022, the FDA authorized an additional booster dose of an mRNA vaccine for adults 50 years of age or older, or for those with immunocompromising conditions (12 years and older with immunocompromising conditions could receive a second booster with the Pfizer vaccine and 18 years and older could receive a second booster with the Moderna vaccine). Subsequent to this authorization, the CDC updated vaccination guidance to permit an additional booster dose for these groups in recognition of the increased and ongoing risk for severe disease in these populations.

The emergence of variant strains has raised concerns about the breadth and duration of immunity and protection achieved by the current vaccines. Studies of vaccine immunogenicity for early variants of concern demonstrated those vaccinated to prototype vaccine may have reduced neutralizing activity to the variants.[28] For variants like B.1.351, pivotal studies testing both viral vector and adjuvanted protein technologies had lower efficacy in regions where B.1.351 was known to be circulating.[29,30] Moreover, sera from individuals vaccinated with mRNA-based vaccines had a 6-to-9-fold reduction in neutralizing activity against a B.1.351-matched pseudovirion relative to a Wuhan-matched pseudovirion.[10,11] Similarly, sera from individuals vaccinated with mRNA-1273 had an 8-fold reduction in neutralizing activity against the B.1.617.2 variant compared to titers against the D614G variant.[21]

Studies have also demonstrated 6.7 fold reduction in neutralizing activity for Pfizer BNT162b2 against B.1.351 with two doses of Pfizer BNT162b2.[31] Additionally, in one assessment of sera obtained from individuals vaccinated with Pfizer BNT162b2, obtained one month after the second dose, a 25 fold reduction in neutralizing antibody geometric mean titers against B.1.1.529 was noted compared to the wild-type virus.[32] Neutralizing activity has been shown to be partially restored by vaccination with a third dose of Pfizer BNT162b2. However, even with three doses, neutralizing activity was 4 times lower against B.1.1.529 and 3 times lower against B.1.351 compared to B.1.617.2 and 7-8 times lower compared to wild-type virus.[33] Thus, additional vaccinations, higher doses or variant specific vaccines may improve protection against current and emerging variants of concern.

Vaccine manufacturers have performed some trials of candidate variant vaccines. For example, a phase 2a open label clinical trial to examine the immunological benefit of boosting subjects previously vaccinated 6 months prior with mRNA-1273 with mRNA-1273.351, a B.1.351 strain-specific mRNA construct (50 mcg), or mRNA-1273.211 (50 mcg) showed 61.6 and 33.7 times higher titers against B.1.351 compared to titers from participants one month after the second dose of the primary series, with similar titers against B.1.617.2.[22] Similar findings were seen from a study of a boost with a bivalent mRNA-1273.213 vaccine (a 1:1 mix of beta and delta variant mRNA).[21]

Similarly, several BNT162b2-based variant vaccines that target these variants are also being developed including BNT162b2 (B.1.617.2), BNT162b2 (B.1.351) and BNT162b2 (B.1.1.529). In addition, Sanofi Pasteur has developed a SARS-CoV2 prefusion Spike delta TM with AS03 adjuvant (CoV2 preS dTM-AS03 [B.1.351]) monovalent vaccine and a SARS-CoV2 prefusion Spike delta TM with AS03 adjuvant, bivalent vaccine (D614/B.1.351 (CoV2 preS dTM-AS03

[D614 + B.1.351]). In a nonhuman primate model, these vaccines used as boosters induced cross-neutralizing antibodies against variants of concern including Delta and Omicron.[34]

However, current efforts are directed towards modifying the existing vaccines to include the currently circulating Omicron variant, though this may not be the circulating variant during the next wave of COVID-19. It is therefore important to look beyond Omicron, and understand the immunologic landscape and how we can use prototype and variant vaccines alone or in combination to address emerging variants.

For seasonal influenza viruses, the antigenic, viral genetic and epidemiological data guide annual updates of the seasonal influenza virus vaccines. Antigenic cartography is a visualization tool aimed at understanding the relationship among SARS-CoV-2 variants of concern (VOCs) in terms of their ability to induce cross-neutralizing antibodies. A recent study using the antigenic mapping approach showed that Omicron represents the first widely circulating new major SARS-CoV-2 variant.[35] Though the dimensions of the cartography, representing new VOCs that could circulate in the future, remain largely unknown, a better understanding of diverse immune landscape induced by vaccination and natural infection could lead, along with vaccine modification, to more durable and broadly protective immunity. The update in vaccine formulations proposed in this study will still rely on similar platforms, route of administration and dosing, though will include an update of immunogens with VOCs alone or in combination that could result in broadly cross reactive serological and cellular immune responses especially in groups with high risk for mortality (e.g., older adults).

This phase 2 clinical trial will evaluate the safety and immunogenicity of additional doses of vaccine to expand and optimize immune coverage of the existing and emerging antigenic space. Utilizing several prototype and variant specific COVID-19 vaccines based on mRNA and other approved platforms, we propose to evaluate innate, cellular, and humoral immune responses elicited from different vaccine candidates. As part of an adaptive design, we anticipate adding groups with other variant-lineage spike proteins and other vaccine platforms, subject to availability.

2.1.1 Public Readiness and Emergency Preparedness Act

The study vaccines and the efforts for this clinical trial are covered under the Public Readiness and Emergency Preparedness Act (PREP Act) and the Declaration issued by the Secretary of the U.S. Department of Health and Human Services under that Act. Under the PREP Act and the Declaration, covered persons (such as manufacturers, distributers, program planners, and other qualified persons who prescribe, administer, or dispense study product) are immune from liability from the administration, or use of a covered countermeasure. The PREP Act provides immunity for covered persons from liability unless the injury was caused by willful misconduct. The Declaration invoking the PREP Act for COVID-19 covered countermeasures was made on March 10, 2020 and is retroactively effective from February 4, 2020.

The PREP Act also established the Countermeasures Injury Compensation Program (CICP) to provide compensation for serious injuries or death that occur as the direct result of the

administration or use of certain countermeasures. Any requests for compensation must be filed within one year of the administration or use of the covered countermeasure. Requests for Benefits must be made to the Health Resources and Services Administration's (HRSA) Countermeasures Injury Compensation Program (http://www.hrsa.gov/cicp/) by filing a Request for Benefits Form and all required medical records and supporting documentation. Additional information on filing a Request for Benefits is available on the CICP's website at http://www.hrsa.gov/cicp/. Compensation may then be available for reasonable and necessary medical benefits, lost wages and/or death benefits to eligible individuals for certain injuries in accordance with regulations published by the Secretary of HHS (found at 42 CFR part 110).

If an individual suffers a serious physical injury or death from the administration or use of a covered countermeasure in this study, the individual, the individual's legal or personal representative, the administrator/executor of a deceased individual's estate, or certain survivors may request benefits from the CICP. A serious physical injury means an injury that warranted hospitalization (whether or not the person was actually hospitalized) or that led to a significant loss of function or disability. The CICP is the payer of last resort. This means that it only covers expenses or provides benefits that other third-party payers (such as health insurance, the Department of Veterans Affairs, or Workers' Compensation programs) do not have an obligation to pay.

If the Secretary of DHHS does not make a final determination on the individual's request within 240 days, or if the individual decides not to accept the compensation, the injured individual or his representative may pursue a tort claim in the US District Court for the District of Columbia, but only if the claim involves willful misconduct and meets the other requirements for suit under the PREP Act. Any award is reduced by any public or private insurance or worker's compensation available to the injured individual. Awards for non-economic damages, such as pain, suffering, physical impairment, mental anguish, and loss of consortium are also limited. If the individual accepts compensation, or if there is no willful misconduct, then the individual does not have a tort claim that can be filed in a US Federal or a State court.

2.2 Risk/Benefit Assessment

2.2.1 Known Potential Risks

The potential risks of participating in this trial are those associated with having blood drawn, IM injection, possible reactions to mRNA vaccines and breach of confidentiality.

Risks of blood draw and IM injection

Drawing blood may cause transient discomfort and fainting. Bruising at the blood draw site may occur but can be prevented or lessened by applying pressure to the blood draw site for a few minutes after the blood is taken. IM injection may also cause transient discomfort and fainting.

Vasovagal syncope (fainting) can occur before or after vaccination or blood draw, is usually triggered by the pain or anxiety caused by the needlestick and is not related to the substance injected. It is important that standard precautions and procedures be followed to avoid injury from

fainting. Fainting is usually transient and managed by having the subject lie down and elevate his/her legs.

Drawing blood and IM injection may cause infection. The use of aseptic (sterile) technique will make infection at the site where blood will be drawn or where the vaccination will be given extremely unlikely.

Risks of mRNA vaccines

- **mRNA-1273, mRNA-1273.351, mRNA-1273.617.2 and mRNA-1273.529**

mRNA-1273 was granted full FDA approval in 2022 and more than 200 million doses of mRNA-1273 have been administered in the US alone as of early 2022.[36] While the other SARS-CoV-2 mRNA vaccine candidates manufactured by Moderna have been given to a small number of individuals in the setting of clinical trials, the safety profile of these vaccine candidates is believed to be largely similar to the approved prototype vaccine. The total amount of immunogens within any study vaccine will be 50 mcg, similar to the authorized boost dose of mRNA-1273.

Immediate systemic allergic reactions (e.g., anaphylaxis) can occur following any vaccines. For the mRNA-1273 vaccines, the rate of anaphylaxis is around 2.5 cases per million doses administered.[37] Most of these reactions have onset within 30 minutes of vaccination and most of these events occurred in persons with a prior history of allergy. As a precaution, all subjects will remain under observation at the study site for at least 30 minutes after injection. Risk management also includes exclusion of participants with history of anaphylaxis, urticaria, or other significant adverse reaction requiring medical intervention after receipt of a vaccine or nanolipid particle.

Infrequently, people who have received dermal fillers might experience swelling at or near the site of filler injection (usually face or lips) following administration of a dose of an mRNA-1273 vaccine. The swelling appears to be temporary and resolves with medical treatment, including corticosteroid therapy. COVID-19 vaccines can be administered to people who have received injectable dermal fillers who have no contraindications or precautions for vaccination.[38]

Reactogenicity has been noted after mRNA-1273 vaccination. Local (pain, redness, swelling and pruritus) and systemic adverse effects (headache, myalgia, fatigue, joint pain, chills, and fever) are relatively common, most are of mild or moderate severity and are limited to the first few days after vaccination.[39] A small percentage of participants may experience late local inflammatory reactions, with onset seven or more days after, usually the first vaccination, and characterized by redness in the deltoid area of the upper arm and/or pain or itching.[39] These reactions are self-limited and are not a contraindication to subsequent vaccinations in the vaccination series.

Laboratory abnormalities (including increases in liver function tests and serum lipase levels) following injection were observed in clinical studies with similar mRNA-based vaccines. These abnormalities were without clinical symptoms or signs and returned toward baseline, pre-vaccination (Day 1) values over time. The clinical significance of these observations is unknown.

Myocarditis and pericarditis have been reported following mRNA vaccines in a younger population (mostly in adolescents and young adults), and are more common in males especially after the second dose of mRNA-1273 and within a few days following receipt of the vaccines.[40] The rate per million second doses given in a 7-day risk period in individuals between the ages of 18-39 years of age is estimated to be around 33 per million.[41] Symptoms can include chest pain,

shortness of breath, or palpitations. Whilst some severe cases have been reported, most cases have been associated with full resolution of symptoms in the short term; however, long-term follow-up is limited.[42] It is not known whether the risk of myocarditis or pericarditis is increased following additional doses of the vaccine, though data after a third dose of mRNA vaccine are reassuring especially with the extended interval between primary series and booster doses compared to the doses given within the primary series.[43-45]

There is limited experience with administration of an additional dose of the mRNA COVID-19 vaccines, and it is possible that this additional dose may be associated with more frequent or more severe adverse events.

Further details are provided in the FDA-approved fact sheet and current IB.

- **BNT162b2, BNT162b2 (B.1.351) BNT162b2 (B.1.1.529)**

BNT162b2 was granted full FDA approval in 2021 and more than 300 million doses of BNT162b2 have been administered in the US alone as of early 2022. While the other SARS-CoV-2 mRNA vaccine candidates manufactured by Pfizer have been given to a small number of individuals in the setting of clinical trials, the safety profile of these vaccine candidates is believed to be largely similar to the approved wildtype (prototype) vaccine. The total amount of immunogens within any study vaccine will be 30 mcg, similar to the authorized boost dose of BNT162b2.

Immediate systemic allergic reactions (e.g., anaphylaxis) can occur following any vaccines. For the BNT162b2 vaccine, the rate of anaphylaxis is around 11 cases per million doses administered.[46] Most of these reactions have onset within 15 minutes of vaccination and most of these events occurred in persons with a prior history of allergy. As a precaution, all subjects will remain under observation at the study site for at least 30 minutes after injection. Risk management also includes exclusion of participants with history of anaphylaxis, urticaria, or other significant adverse reaction requiring medical intervention after receipt of a vaccine or nanolipid particle.

Reactogenicity has been noted after BNT162b2 vaccination. Local (pain, redness, swelling and pruritus) and systemic adverse effects (headache, myalgia, fatigue, lethargy, decreased appetite, night sweats, malaise, joint pain, diarrhea, chills, and fever) are relatively common, most are of mild or moderate severity and are limited to the first few days after vaccination.[47] A subset from Study C4591001 Phase2/3 participants of 306 adults at least 18 through 55 years of age who completed the primary BNT162b22-dose course, received a booster dose (third dose) of BNT162b2 ~6 months (range of 4.8 to 8.0 months) after receiving Dose 2. The most frequent reactogenicity in participants 18 through 55 years of age were injection site pain (>80%), fatigue (>60%), headache (>40%), myalgia (>30%), chills and arthralgia (>20%).

Myocarditis and pericarditis have been reported following BNT162b2 vaccines in a younger population (mostly in adolescents and young adults), and are more common in males especially after the second dose of BNT162b2 and within a few days following receipt of the vaccines.[40] According to one study, the rate in individuals 18-24 years of age is 5 times lower after BNT162b2 second dose compared to mRNA-1273 second dose. Symptoms can include chest pain, shortness of breath, or palpitations. Whilst some severe cases have been reported, most cases have been associated with full resolution of symptoms in the short term; however, long-term follow-up is limited.[40] It is not known whether the risk of myocarditis or pericarditis is increased following

additional doses of the vaccine, though data after a third dose of mRNA vaccine are reassuring especially with the extended interval between primary series and booster doses compared to the doses given within the primary series.[40]

There is limited experience with administration of an additional dose of the mRNA COVID-19 vaccines, and it is possible that this additional dose may be associated with more frequent or more severe adverse events.

Further details are provided in the FDA-approved fact sheet and current IB.

- **CoV2 preS dTM-AS03 [B.1.351]) and CoV2 preS dTM-AS03 [D614 + B.1.351].**

CoV2 preS dTM-AS03 has been found to have an acceptable safety profile in phase 2 and phase 3 trials totaling 25,000 participants including more than 13,000 receiving the bivalent vaccine with the plan to offer beta vaccine boosting in the current phase 3 trial.[24] The total amount of immunogens within any Sanofi-manufactured study vaccine candidates will be 5 mcg used in the company booster strategy for the phase 3 trial.

Immediate systemic allergic reactions (e.g., anaphylaxis) can occur following any vaccine. In the phase 2 trial involving 721 participants who received at least one injection, no anaphylaxis was reported.[23] In the phase 2 booster cohorts, one participant reported a possible anaphylactic reaction among 1276 participants. As a precaution, all subjects will remain under observation at the study site for at least 30 minutes after injection. Risk management also includes exclusion of participants with history of anaphylaxis, urticaria, or other significant adverse reaction requiring medical intervention after receipt of a vaccine.

Reactogenicity has been noted after CoV2 preS dTM-AS03 vaccination. Local (pain, redness, swelling and pruritus) and systemic adverse effects (headache, myalgia, fatigue, lethargy, decreased appetite, night sweats, malaise, joint pain, diarrhea, chills, and fever) are relatively common, most are of mild or moderate severity and are limited to the first few days after vaccination.[23] Per the current IB, a similar reactogenicity profile was observed in participants who received a booster dose. Grade 3 solicited reactions in a heterologous prime/boost strategy were 5.5% with BNT162b2 and 10.7% with mRNA-1273 prime dose.

An increased risk of narcolepsy was noted in some participants after vaccination with Pandemrix, a different AS03-adjuvanted vaccine for H1N1 influenza, but has not been observed with other AS03-adjuvanted vaccines. The European Medicines Agency (EMA) stated that “a hypothesis that takes into account the potential role of antigen is more likely to explain the increased risk of narcolepsy observed with Pandemrix than hypotheses that are based on a direct role for the AS03 adjuvant”.[48] Safety concerns regarding narcolepsy have not been identified in trials of AS03-adjuvanted SARS-CoV-2 vaccine candidates to date.[23,24]

There is limited experience with administration of an additional dose of COVID-19 vaccines, and it is possible that an additional dose may be associated with more frequent or more severe adverse events.

Further details are provided in the current IB.

Risks to Privacy

Subjects will be asked to provide personal health information (PHI). All attempts will be made to keep this PHI confidential within the limits of the law. However, there is a chance that unauthorized persons will see the subject's PHI. All study records will be kept in a locked file cabinet or maintained in a locked room at the participating site. Electronic files will be password protected. Only people who are involved in the conduct, oversight, monitoring, or auditing of this trial will be allowed access to the PHI that is collected. Any publications from this trial will not use information that will identify subjects by name. Organizations that may inspect and/or copy research records maintained at the participating site for quality assurance (QA) and data analysis include groups such as the IRB, NIAID and the FDA.

A description of this clinical trial will be available on http://www.ClinicalTrials.gov, as required by US Law. This web site will not include information that can identify subjects.

There may be other risks, discomforts or side effects that are unknown at this time.

Risks of Genetic Testing

Any genetic data generated will be kept private. There may be a risk that information resulting from research genetic testing could be misused for discriminatory purposes. However, state and federal laws provide protections against genetic discrimination. Researchers will need to maintain confidentiality in order to be granted access to genetic information.

2.2.2 Known Potential Benefits

On March 29, 2022, the FDA authorized an additional booster dose (fourth dose) of mRNA vaccine for adults 50 years of age or older and adults with immunocompromising conditions. Subsequent to this authorization, the CDC updated its guidance to permit an additional booster dose for these groups, acknowledging the potential benefit of a second booster for those at risk for severe COVID-19 illnesses as well as for those who received two doses of the Johnson & Johnson vaccine. The exact benefit of this second booster dose is difficult to ascertain given the design of the studies supporting these recommendations. Variant specific mRNA vaccine candidates have been shown to induce similar D614G antibody responses as the prototype mRNA vaccine (i.e. back boost). Therefore, receipt of a variant vaccine in this study is not expected to be inferior to any benefits received from a second booster dose of a Pfizer or Moderna mRNA vaccine obtained outside this trial (i.e. is not inferior to standard care). The Sanofi/GSK COVID-19 vaccine is not yet FDA authorized; the potential benefit of a booster dose is unknown.

There may be benefit to society resulting from insights gained from participation in this study due to the emerging threat of the SARS-CoV-2 variants.

3. OBJECTIVES AND ENDPOINTS

Table 4: Objectives and Endpoints (Outcome Measures)

OBJECTIVES	ENDPOINTS (OUTCOME MEASURES)
Primary	
• To evaluate humoral immune responses of candidate SARS-CoV-2 variant vaccines.	• Response rate, and magnitude of SARS-CoV-2-specific antibody binding and neutralization titers in serum samples as assessed via a range of assays at all timepoints.
Secondary	
• To evaluate the safety of candidate SARS-CoV-2 variant vaccines	• Local and systemic solicited Adverse Events for 7 days following each vaccine dose.<br>• Unsolicited Adverse Events from Dose 1 to 28 days following each vaccine dose.<br>• SAEs, MAAEs, NOCMCs, AESIs, and AEs leading to withdrawal from the study from Dose 1 to 12 months after last vaccine dose.
Exploratory	
• To assess, in at least a subset of samples, the B cell immune response of candidate SARS-CoV-2 variant vaccines.	• Magnitude, phenotype and percentage of SARS-CoV-2 specific B cells, as measured by flow cytometry and targeted B cell subset analysis at selected time points post-vaccination.
• To assess, in at least a subset of samples, the SARS-CoV-2 spike protein-specific T cell responses of candidate SARS-CoV-2 variant vaccines.	• Magnitude, phenotype, and percentage of cytokine producing S protein T cells as measured by flow cytometry at selected time points post-vaccination.
• To assess, in at least a subset of samples, the magnitude, phenotype, and percentage of innate immune cells with candidate SARS-CoV-2 variant vaccines.	• Magnitude, phenotype, and percentage of innate immune cells as measured by flow cytometry at D1 and 14 days after each vaccination.

OBJECTIVES	ENDPOINTS (OUTCOME MEASURES)
• To assess, in at least a subset of samples, the functional potential of SARS-CoV-2 specific antibodies to mediate Fc-effector functions across candidate SARS-CoV-2 variant vaccines.	• Characterization of antigen-specific antibody by a) subclass, b) isotype, c) ability to interact with Fc receptors, d) innate immune receptors, e) lectin-like molecules and f) lectins at different times post-vaccination.<br>• Functional Fc effector assessments which may include quantification of antigen specific antibody-mediated NK cell activation (ADCC-NK) levels, antibody-dependent cellular phagocytosis (ADCP) levels, antibody-dependent complement deposition (ADCD) and/or antibody-dependent neutrophil activation/phagocytosis levels at different times post-vaccination.
• To evaluate breakthrough SARS-CoV-2 infection by sequencing strains for variant spike lineage and assessing anti- nucleocapsid serology.	• Sequence analysis on breakthrough NAAT-confirmed SARS-CoV-2 strains at any time post-vaccination<br>• Anti- nucleocapsid immunoassay at different times post-vaccination.

4. STUDY DESIGN

4.1 Overall Design

This is a Phase 2 randomized, adaptive, open label, non-placebo controlled multi-site, multi-stage clinical trial in individuals, 18 years of age and older, who are in stable state of health, have received a complete authorized/approved vaccine series (primary series + booster either with homologous or heterologous vaccine products) and meet all other eligibility criteria.

Approximately 1500 subjects will be enrolled at up to 30 clinical research sites. At enrollment, subjects will be stratified by i) age (18-64 and ≥ 65 years of age) and ii) confirmed prior SARS-CoV-2 infection, and randomly assigned to one of several study arms of variant vaccines. The study will target the enrollment of approximately 45% of older adults (≥ 65 years of age). Additionally, the study will target to enroll approximately 20% enrollment of participants with a history of confirmed SARS-CoV-2 infection. It is estimated that another 10-30% will have undiagnosed prior infection. Taken together, we anticipate about 40% of the total will have prior infection. The study team will decide, based on latest epidemiology, if enrollment of participants in any particular groups are slowed in order to ensure good representation of previously infected and not previously infected.

The adaptive design of this study will include additional arms, an increase in sample size, or additional vaccine doses or different dosing and provide rapid information about the immunogenicity and the safety of candidate SARS-CoV-2 variant vaccines to inform near term public health policy. This trial will provide proof of concept on how variant vaccines could shift the immune response to become more specific to the VOCs' antigens or broaden the antigenic landscape for emergent variants.

Study arms and stages are described in Section 1.1.

Stages 1 and 2 of the study constitute the highest priority investigation to inform US vaccine policy.

Screening can occur up to 28 days prior to the first dose of study product or on Day 1 prior to administration of Dose 1. Safety will be assessed during the study and blood will be drawn for immunogenicity assays at enrollment and in-person follow-up visits. Swabs will be self-collected or collected by staff at unscheduled illness visits to evaluate breakthrough SARS-CoV-2 infection (symptomatic infection or asymptomatic infection with a positive SARS-CoV-2 test outside the study).

Schedules of assessments are found in Section 1.2.

4.2 Scientific Rationale for Study Design

An open-label study will facilitate the need for rapid review and dissemination of study data for public health reasons. Safety findings are not expected to be dissimilar from Phase 1/2/3 studies conducted for similar vaccine constructs. Randomization will improve comparisons between the different arms. Each stage will contain an arm with the prototype vaccine.

Selected arms will assess 2 study doses of vaccine candidates to assess if 2 doses provide a more durable response and more specific to variant viruses.

We chose the interval of 4 months since a recent study performed by our investigators showed that an interval of 3 months between primary series and boost had acceptable safety and immunogenicity responses.[19]

4.3 Justification for Doses

The dosages of mRNA vaccines selected are those authorized for booster doses under EUA, and previously shown in prior studies to elicit good immune responses with no major safety concerns.

In an open label study of participants in the Moderna Phase 3 clinical trial who received a single 50 mcg booster dose of mRNA-1273 ≥ 6 months after a primary series, a 13 fold rise in antibody titers against D614G was noted from pre-boost levels as well as a 17-fold rise in in titers against the B.1.617.2 variant.[20] This is now the CDC recommended dose for booster vaccinations with the Moderna mRNA-1273 product in immunocompetent adults, to be given at least 5 months from the last inoculation in the primary series.[49] Similarly, in a recent study, boosting with a 50 mcg total antigen dose of a bivalent mRNA-1273.213 vaccine (a 1:1 mix of beta and delta variant mRNA) produced only modestly lower antibody titers than using a 100 mcg booster dose.[21] A separate study evaluating safety and immunogenicity of multiple boost strategies suggests that a heterologous boost strategy generates antibody and neutralizing responses and that 100 mcg of mRNA-1273 (the authorized dose at the time) is likely higher than needed for a booster dose after primary mRNA series.[50] In addition, surveillance data indicate that following an mRNA boost, local and systemic reactions are less common and myocarditis is rarely reported, suggesting that the currently authorized doses are safe.[45] These findings support the evaluation of mRNA-1273, mRNA-1273.351, mRNA-1273.617.2 and mRNA-1273.529 at total dosages of 50 mcg per vaccination.

For BNT162b2, the dose is the same for primary or booster doses in adults. In an open-label clinical study, immunogenicity and safety of a fourth dose of either 30 mcg of BNT162b2 (Pfizer–BioNTech) or 50 mcg of mRNA-1273 (Moderna) administered 4 months after the third dose was assessed in 154 and 120 health care workers, respectively.[25] After the fourth dose, both messenger RNA (mRNA) vaccines increased neutralizing antibody titers to D614G, B.1.617.2 and B.1.1.529 by a factor of 9 to 10 and were slightly higher than those achieved after the third dose.[25] There were no significant adverse events with only mild system and local reactogenicity reported which was comparable with previous doses of mRNA vaccines.

For CoV2 preS dTM-AS03 vaccines, none is currently approved. The dose used is as established in the phase 2 trial and is 5 mcg for any variant vaccine candidate in stage 3.[24] The 5 mcg booster dose increased neutralizing antibody titers when used as a heterologous (18-30 times) or homologous (≥ 84 times) boost.[24] However, no data exist on CoV2 preS dTM-AS03 vaccines as a second boost after primary series with mRNA or adenoviral vectored vaccines.

5. STUDY POPULATION

All participants will have received a complete (primary and booster) US approved/authorized vaccine series. The US population has received either homologous primary series and boost (i.e. Moderna mRNA-1273 for 1st dose, second dose, and booster), or heterologous boosters, and some were infected with SARS-CoV-2 either prior to or after vaccination. This trial will enroll this immunologically diverse population, to ensure any findings are applicable to the larger population.

Based on CDC data about use of primary vaccine or boost (https://covid.cdc.gov/covid-data-tracker/#vaccinations_vacc-total-admin-rate-total), it is estimated that of people in the US that have received a primary vaccine series and booster:

- 53% will have received Pfizer primary series and Pfizer boost,
- 36% will have received Moderna primary series and Moderna boost,
- 4% will have received Pfizer primary series and Moderna boost,
- 3% will have received Moderna primary series and Pfizer boost,
- 1% will have received Janssen primary series and Janssen boost,
- 1% will have received Janssen primary series and Moderna boost,
- 1% will have received Janssen primary series and Pfizer boost.

Additionally, approximately 20% of the US population has been infected with SARS-CoV-2, likely higher with asymptomatic infections.[51]

It is anticipated the enrollment into this trial will be similar to these proportions.

Subject Inclusion and Exclusion Criteria must be confirmed by a study clinician, licensed to make medical diagnoses and listed on the Form FDA 1572. No exemptions are granted on Subject Inclusion or Exclusion Criteria in DMID-sponsored studies.

5.1 Inclusion Criteria

See Inclusion Criteria in Section 1.1.

5.2 Exclusion Criteria

See Exclusion Criteria in Section 1.1.

5.2.1 Exclusion of specific populations

The effects of SARS-CoV-2 vaccines on the developing fetus are not known, though there are data to suggest that mRNA vaccines are safe. Pregnant subjects will not be eligible for the trial as the immune responses to vaccines can be different than non-pregnant subjects. Children will not be included in this trial as presently there are limited safety data in adults for the variant strains.

5.3 Inclusion of Vulnerable Subjects

No vulnerable subjects will be enrolled in this trial.

5.4 Lifestyle Considerations

During this study subjects are asked to:

- Follow public health guidance on preventing SARS-CoV-2 infection.

5.5 Screen Failures

Screening and enrollment visits may be combined. However, the participating site PI or qualified designee is to review the inclusion and exclusion criteria and determine the subject's eligibility for the study. Individuals who do not meet the criteria for participation in the study (screen failure) can be rescreened if the exclusionary condition is considered to be temporary in nature by the investigator.

Only the following information will be collected on screen failures: demographics (age, screen number, sex, ethnicity, and race) and reason for ineligibility. Subjects who are found to be ineligible will be told the reason for ineligibility.

5.5.1 Strategies for Recruitment and Retention

The study will target recruitment of racial and ethnic diversity of the community in which the study will be conducted. Potential subjects will learn about the study via IRB-approved recruitment strategies, including direct mailing, recruitment from an IRB-approved trial registry, press releases, social media and local advertisements/flyers. Screening will begin with a brief approved telephone call from study staff. Information about the study will be presented to potential subjects and questions about their health and ability to comply with the study visit schedule will be asked of potential subjects to presumptively determine eligibility. Appointments will be made at the research clinic for potential subjects who are interested in the study for further screening procedures and additional protocol-specific information.

5.5.2 Retention

Study retention strategies will include education and explanation of the study schedule and procedures during screening and enrollment/baseline visits and restriction of enrollment to persons who can attend all study visits. Participating subjects will be reminded of subsequent visits during each visit, and study staff will contact subjects prior to appointments. Study staff will contact subjects who miss appointments to encourage them to return for completion of safety evaluations.

5.5.3 Compensation Plan for Subjects

Subjects may be compensated for their participation in this trial. Compensation will be in accordance with local IRB requirements, and subject to local IRB approval. Reimbursements will be disbursed at specific timepoints during the study with the amount contingent on completing study procedures.

5.5.4 Costs

There is no cost to subjects for the research tests, procedures/evaluations or study product while taking part in this trial. Procedures and treatment for clinical care may be billed to the subject, subject's insurance or third party.

6. STUDY PRODUCT

6.1 Study Product(s) and Administration

6.1.1 Study Product Description

Products: mRNA-1273 (prototype), mRNA-1273.351 (Beta), mRNA-1273.617.2 (Delta) and mRNA-1273.529 (Omicron)

mRNA-1273 (0.2 mg/mL) is an LNP dispersion containing an mRNA that encodes for the pre fusion stabilized S protein of the Wuhan-Hu-1 strain of SARS-CoV-2. mRNA-1273 consists of an mRNA Drug Substance that is manufactured into LNPs composed of the proprietary ionizable lipid, SM-102, and 3 commercially available lipids, cholesterol, 1,2-distearoyl-sn-glycero-3-phosphocholine (DSPC), and PEG2000 DMG.

mRNA-1273.351 (0.1 mg/mL) is formulated in the same way but contains mRNA that encodes for the prefusion stabilized S protein of the B.1.351 (Beta) variant SARS-CoV-2 strain.

mRNA-1273.617.2 (0.2 mg/mL) is formulated in the same way but contains mRNA that encodes for the prefusion stabilized S protein of the B.1.617.2 (Delta) variant SARS-CoV-2 strain.

mRNA-1273.529 (0.2 mg/mL) is formulated in the same way but contains mRNA that encodes for the prefusion stabilized S protein of the B.1.1.529 (Omicron) variant SARS-CoV-2 strain.

BNT162b2 (wildtype), BNT162b2 (B.1.351) (Beta) and BNT162b2 (B1.1.529) (Omicron)

BNT162b2 (500 mcg/mL) is a preservative-free, sterile dispersion of RNA formulated in LNP in aqueous cryoprotectant buffer. mRNA encodes for the pre fusion stabilized S protein of the ancestral strain of SARS-CoV-2.

BNT162b2 (B.1.351) (500 mcg/mL) is formulated in the same way but contains mRNA that encodes for the prefusion stabilized S protein of the B.1.351 (Beta) variant SARS-CoV-2 strain.

BNT162b2 (B.1.1.529) (500 mcg/mL) is formulated in the same way but contains mRNA that encodes for the prefusion stabilized S protein of the B.1.1.529 (Omicron) variant SARS-CoV-2 strain.

CoV2 preS dTM-AS03 [D614] (prototype), CoV2 preS dTM-AS03 [B.1.351] (Beta), and CoV2 preS dTM-AS03 [D614 + B.1.351] (prototype + Beta)

CoV2 preS dTM-AS03 [D614] (Recombinant COVID-19 Vaccine 20 mcg/mL + AS03) is a liquid formulation made of recombinant protein placed in a formulation buffer and to be mixed with equal volumes of AS03 adjuvant (supplied separately) at the study site before administration. The antigen solution contains the Spike protein sequence of the ancestral strain of SARS-CoV-2. AS03 is an adjuvant system containing α-tocopherol and squalene in an oil/water emulsion. For this formulation, the antigen and AS03 vials are not packaged in a carton box together but are supplied as individual vial.

CoV2 preS dTM-AS03 [B.1.351] (Recombinant COVID-19 Vaccine B1.351 20 mcg/mL+ AS03) is formulated in the same way but contains the Spike protein sequence of the B.1.351 (Beta) variant SARS-CoV-2 strain. Both antigen and AS03 vials are packaged in a carton box.

CoV2 preS dTM-AS03 [D614 + B.1.351] (Recombinant COVID-19 Bivalent Vaccine 10/10 mcg/mL + AS03) is formulated in the same way but contains the Spike protein sequences of the ancestral and B.1.351 (Beta) variant SARS-CoV-2 strains. Both antigen and AS03 vials are packaged in a carton box.**Diluent: 0.9% NaCl for injection, USP**

The USP grade 0.9% NaCl or normal saline for injection is a sterile, nonpyrogenic, isotonic solution; each mL contains NaCl 9 mg. It contains no bacteriostatic agent, antimicrobial agent, preservatives, or added buffer and is supplied only in single-dose containers. This product should be used to dilute the vaccine to the desired concentration.

6.1.2 Dosing and Administration

mRNA-1273, mRNA-1273.351, mRNA-1273.617.2 and mRNA-1273.529

mRNA-1273 (0.2 mg/mL), mRNA-1273.351 (0.1 mg/mL), mRNA-1273.529 (0.2 mg/mL), and mRNA-1273.617.2 (0.2 mg/mL).

Products will be used alone or in combination as specified in Table 1, and will be diluted in 0.9% NaCl for injection, USP to obtain an antigen content of 50 mcg in a 0.5 mL dose. Each dose will be administered via IM injection into the deltoid muscle.

The expiration time of the dosing syringe containing the prepared study vaccine is 8 hours at room temperature after the solution is drawn into the dosing syringe.

When applicable, the second dose of study vaccine can be administered in either arm regardless of the arm that previously administered.

BNT162b2, BNT162b2 (B.1.351) and BNT162b2 (B1.1.529)

BNT162b2, BNT162b2 (B.1.351) and BNT162b2 (B.1.1.529) all at 500 mcg/mL concentration will be used alone or in combination as specified in Table 1 and will be diluted in 0.9% NaCl for injection, USP to obtain an antigen content of 30 mcg in a 0.3 mL dose. Each dose will be administered via IM injection into the deltoid muscle.

The expiration time of the dosing syringe containing the prepared study vaccine is 6 hours at room temperature after the 1st punctured vial..

CoV2 preS dTM-AS03 [D614], CoV2 preS dTM-AS03 [B.1.351], and CoV2 preS dTM-AS03 [D614 + B.1.351]

The multi-dose vial of CoV2 preS dTM antigen, either monovalent or bivalent antigen, will be mixed with the multi-dose vial of AS03 adjuvant prior to administration to obtain an antigen content of 5 mcg in a 0.5 mL dose. Vaccine formulations will be prepared in the CoV2 preS dTM antigen vials by adding an equal volume of adjuvant. A maximum of 10 doses can be used from a single prepared investigational vaccine final product.

After mixing the investigational product, the combined final product can be held for up to 12 hours post preparation at 2 to 8 °C or at controlled room temperatures (up to 23 to 27 °C) and must be protected from light. Syringes containing the final product should be covered to limit exposure to light as well.

For all stages , the pharmacist will prepare a single dose for each subject based on study arm assignment.

See the protocol-specific Manual of Procedures (MOP) for detailed information on the preparation, labeling, storage, and administration of vaccine for each cohort. Vaccine preparation will be performed by the participating site's research pharmacist on the same day of vaccine administration to the subject.

Subjects will be observed in the clinic for at least 30 minutes post each vaccination. Any AEs will be recorded on the appropriate DCF prior to discharge from the clinic

6.1.3 Dose Modifications

Not applicable.

6.2 Accountability/Handling/Storage/Preparation

6.2.1 Acquisition and Accountability

All the vaccines (and diluents as needed) will be provided by the DMID repository:

DMID Clinical Materials Services Contract
Fisher BioServices
20439 Seneca Meadows Parkway
Germantown, MD 20876
Phone: 240-477-1350
Fax: 240-477-1360
Email: DMID.CMS@thermofisher.com

All study products will be shipped to the clinical research site upon request and approval from DMID.

Accountability

The participating site PI is responsible for study product distribution and disposition and has ultimate responsibility for study product accountability. The participating site PI may delegate to the participating site's research pharmacist responsibility for study product accountability. The participating site's research pharmacist will be responsible for maintaining complete records and documentation of study product receipt, accountability, dispensation, storage conditions, and final disposition of the study product(s).

Destruction

After the study treatment period has ended or as appropriate over the course of the study after study product accountability has been performed, disposition of unused and used study product vials should occur as noted:

- Unused and used study product vials:
    - Should be destroyed on-site following applicable site procedures or by the site's selected destruction vendor. Following the site's procedure for the destruction of hazardous material or study product destruction policy/SOP when destroying used and unused items.

- A certificate of destruction or documentation of destruction should be provided to the sponsor and retained in the Pharmacy Binder once completed.

- Used syringes may be destroyed in accordance with site-specific SOPs.

6.2.2 Formulation and Appearance

mRNA-1273, mRNA-1273.351, mRNA-1273.617.2 and mRNA-1273.529 are provided as a sterile liquid for injection, white to off-white suspension in appearance.

BNT162b2, BNT162b2 (B.1.351) and BNT162b2 (B.1.1.529) are provided as a sterile, clear colorless liquid for injection.

CoV2 preS dTM-AS03 [D614], CoV2 preS dTM-AS03 [B.1.351], and CoV2 preS dTM-AS03 [D614 + B.1.351] antigen formulations are provided as a ~~vaccine is a~~ sterile, clear, colorless solution of SARS-CoV-2 prefusion S proteins. The antigen solutions can contain endogenous particles. If present, these light-colored particles are slow sinking and suspended in solution. AS03 adjuvant is a whitish to yellowish homogenous milky liquid emulsion. After mixing, the vaccine is a whitish to yellowish homogeneous milky liquid emulsion

Diluent: 0.9% NaCl for injection, USP

The USP grade 0.9% NaCl or normal saline for injection is a sterile, nonpyrogenic, isotonic solution; each mL contains NaCl 9 mg. It contains no bacteriostatic agent, antimicrobial agent, preservatives, or added buffer and is supplied only in single-dose containers.

Each of the study products will be labeled according to manufacturer specifications and include the statement “Caution: New Drug Limited by Federal Law to Investigational Use.”

6.2.3 Product Storage and Stability

The storage of mRNA-1273,BNT162b2, and CoV2 preS dTM-AS03 vials depend on the presentation (i.e. commercial lots have different storage conditions from investigational lots). For these reasons, refer to the MOP for storage conditions for vaccine candidates as well as storage of diluent.

Prepared doses are stable for clinical in-use for:

- mRNA-1273, mRNA-1273.351, mRNA-1273.617.2 and mRNA-1273.529: up to 8 hours at room temperature
- BNT162b2, BNT162b2 (B.1.351) and BNT162b2 (B.1.1.529): up to 6 hours at room temperature after 1st vial is puntured.
- CoV2 preS dTM-AS03 [D614], CoV2 preS dTM-AS03 [B.1.351], and CoV2 preS dTM-AS03 [D614 + B.1.351]: up to 12 hours post preparation at 2 to 8 °C or at controlled room temperatures (up to 23 to 27 °C) and must be protected from light
-

Study Product Temperature Accountability

The temperature of the storage unit must be manually recorded daily (excluding non-business days and holidays, as applicable) and continuously monitored and recorded during the course of this trial per site-specific SOPs, and documentation will be maintained. If the temperature fluctuates

outside of the required range, the affected study product(s) must be quarantined at the correct storage temperature and labeled as 'Do Not Use' (until further notice).

Study product must be stored in a secure area with limited access (pharmacy staff only) and must be stored as above.

6.2.4 Preparation

Refer to the protocol-specific MOP for details about preparation.

6.3 Measures to Minimize Bias: Randomization and Blinding

6.3.1 Treatment Assignment Procedures

Per International Council for Harmonisation of Technical Requirements for Pharmaceuticals for Human Use (ICH) guideline E6: GCP, screening records will be kept at the participating site to document the reason why an individual was screened, but failed trial entry criteria. The reasons why individuals failed screening will be recorded in Advantage eClinical, the electronic data capture (EDC) system that the Statistical and Data Coordinating Center (SDCC) develops and manages.

Once consented and upon entry of demographic data and confirmation of eligibility for this trial, the subjects will be enrolled.

6.3.2 Randomization and Blinding

Subjects will be randomized to study intervention in an equal allocation ratio. The study will be open label and study sites will administer product to a subject according to which study arm the subject has been assigned. Sites will be asked to recruit for all arms within a stage.

6.3.3 Blinding and Masking Procedures

This study is unblinded.

6.4 Study Intervention Compliance

Each dose of study product will be administered by a member of the clinical research team that is qualified and licensed to administer the study product. Administration and date, time, and location of injection will be recorded on the appropriate DCF.

6.5 Concomitant Therapy

Concomitant medications include prescription medications and vaccinations taken within 28 days of screening or Day 1. Topical, ophthalmic and otic medications, herbal supplements and vitamins will not be collected. This information is asked to assess eligibility. Only COVID-19 vaccination received at any time in the past will be captured in the trial database. After enrollment, immunosuppressant drugs, COVID-19 vaccines received outside of the study as well as COVID-19 related treatment and prophylaxis should be reported at any time during study participation and captured in the trial database. At each study visit, if there are new SAEs, concomitant medications should be recorded on the appropriate DCF and not included in the database unless these are immunosuppressant drugs, COVID-19 vaccines received outside of the study or COVID-19 related treatment and prophylaxis.

6.5.1 Rescue Medicine

Not Applicable.

6.5.2 Non-Research Standard of Care

Not Applicable.

7. STUDY INTERVENTION DISCONTINUATION AND SUBJECT DISCONTINUATION/WITHDRAWAL

7.1 Halting Criteria and Discontinuation of Study Intervention

7.1.1 Halting Criteria

The study will be halted in a given arm if any of the following events occur following any dose:

1- Any subject experiences an SAE after administration of the vaccine that is considered related to vaccine.

2- Any subject experiences laryngospasm, bronchospasm or anaphylaxis within 24 hours after administration of vaccine that is considered related to vaccine.

3- Any subject experiences ulceration, abscess or necrosis at the injection site that is considered related to vaccine administration.

4- Two (2) or more subjects experience an immediate IgE-mediated allergic reaction such as generalized urticaria (defined as occurring at three or more body parts) that is considered related to vaccine.

5- Three (3) or more subjects experience a Grade 3 AE (unsolicited) related to vaccine administration, in the same Preferred Terms based on the Medical Dictionary for Regulatory Activities (MedDRA) coding.

6- One (1) or more participant with myocarditis and/or pericarditis meeting the CDC case definition that is considered related to study vaccine.

7.1.2 Criteria for Continuation of Dosing and Redosing

In the event a halting rule is met:

- An unscheduled safety analysis by the DSMB will be required for approval of further enrollment
- Further administration of any study vaccine boost arms will be suspended until an assessment by the DSMB takes place.

7.1.3 Discontinuation of Study Intervention

For any arm of the study where participants will receive more than a single dose, subjects will need to be reassessed prior to the second dose. The following events constitute contraindications to any further administration of study vaccines. If any of these events occur during the study prior to the next vaccination, the subject will <u>not</u> receive the next vaccination but will be encouraged to continue study participation for safety and immunogenicity evaluations as per the Schedule of Activities for subjects receiving a single dose of vaccine.

- Any clinically significant medical condition that, in the opinion of the participating site PI or appropriate sub-investigator, poses an additional risk to the subject if he/she continues to participate in the study.

- Receipt of SARS-CoV-2 vaccine outside of the study or SARS-CoV-2 immunoglobulin, monoclonal antibody or plasma antibody.
- Confirmed symptomatic or asymptomatic SARS-CoV-2 infection after entry.
- Anaphylaxis or unexpected systemic hypersensitivity reaction following the administration of a prior study vaccination.
- Any SAE judged to be related to vaccine.
- Pregnancy.
- New information becomes available that makes further participation unsafe or interferes with the evaluation of responses.
- Termination of this trial.

7.1.3.1 Delay of Study Vaccination

If any of these events occur at the time scheduled for vaccination, the subject may be vaccinated at a later date.

- Acute moderate or severe infection with or without fever at the time of vaccination.
- Fever, defined as oral temperature ≥38.0°C (100.4°F) at the time of vaccination.

Subjects with a minor illness without fever, as assessed by the participating site PI or appropriate sub-investigator, can be administered vaccines. Subjects with an oral temperature of 38.0°C (100.4°F) or higher will be re-contacted within the window specified in the SOA and re-evaluated.

For subjects for whom a second dose is planned, it is preferred that the vaccination still occur within the window specified in the SOA if possible but delays outside the windows are permitted (would still be a protocol deviation). If intercurrent SARS- CoV-2 infection occurs between the first and prior to the planned second study dose, no second vaccination will be given.

7.1.4 Follow-up for Subjects that Discontinued Study Intervention

Discontinuation of study intervention does not require discontinuation from the study, and the remaining study procedures should be completed. Participants who do not receive a second dose will revert to the schedule of activities (SOA) for single dose participants.

If a clinically significant finding is identified, including, but not limited to, changes from baseline, after enrollment, the participating site PI or qualified designee will determine if any change in subject management is needed (Table 3).

7.1.5 Follow up for Subjects with a Suspicion for Myocarditis and/or Pericarditis

In the event that a subject develops symptoms which in the judgement of the investigator, is consistent with a case of suspected myocarditis and/or pericarditis within 4 weeks from vaccination, the site will conduct an unscheduled illness visit. Additionally, a serum sample will be obtained and stored for potential biomarker testing at a central laboratory. The site will coordinate an appropriate diagnostic workup to make a determination of probable or confirmed myocarditis and/or pericarditis which may include, but is not limited to, an ECG, cardiac

troponin testing (T or I) and referral to a cardiologist (directly, or through the emergency department or primary care clinic). The suspected myocarditis and/or pericarditis case should be reported to the DMID Medical Monitor within 24 hours of site awareness.

The definitions below are intended to serve as a guide to help in the reporting of suspected cases of myocarditis and/or pericarditis; however, the diagnosis of suspected cases is left to the investigator's clinical judgement.

7.1.5.1 Probable Case of Acute Myocarditis

Presence of ≥ 1 new or worsening of the following clinical symptoms:

• Chest pain/pressure/discomfort

• Dyspnea/shortness of breath/pain with breathing

• Palpitations

• Syncope

AND

Presence of ≥ 1 new finding of the following:

• Troponin level above upper limit of normal (any type of troponin)

• Abnormal electrocardiogram (ECG or EKG) or rhythm monitoring findings consistent with myocarditis which includes at least one of the following:

▪ ST segment or T-wave abnormalities

▪ Paroxysmal or sustained atrial, supraventricular, or ventricular arrhythmias

▪ AV nodal conduction delays or intraventricular conduction defects

- Abnormal cardiac function or wall motion abnormalities on echocardiogram
- Cardiac magnetic resonance imaging (cMRI) finding consistent with myocarditis[52]

AND

- No other identifiable cause of the symptoms and findings

7.1.5.2 Confirmed Case of Acute Myocarditis

Meets the case definition for a probable case

AND

• Histopathologic confirmation of myocarditis (using Dallas criteria)[53] OR

• cMRI findings consistent with myocarditis in the presence of troponin level above upper limit of normal (any type of troponin)

AND

• No other identifiable cause of the symptoms and findings

7.1.5.3 Acute Pericarditis Case Definition

Presence of ≥ 2 new or worsening of the following clinical features[54]:

• Acute chest pain (Typically described as pain made worse by lying down, deep inspiration, or cough; and relieved by sitting up or leaning forward, although other types of chest pain may occur)

• Pericardial rub on examination

• New ST-elevation or PR-depression on EKG

• New or worsening pericardial effusion on echocardiogram or magnetic resonance imaging

7.1.5.4 Myopericarditis Case Definition

Participants who meet criteria for both myocarditis and pericarditis may be described under myopericarditis.

7.2 Subject Withdrawal from the Study and Replacement

Subjects are free to withdraw from participation in the study at any time upon request, without any consequence.

A study subject will be discontinued from participation in the study if any of the following reasons occur prior to initial dosing:

- Request by the subject to terminate participation.
- Initial vaccine is not administered.

A subject may be removed from the study for the following reasons post initial dosing; however, whenever possible the subject should be followed for safety and immunogenicity evaluations per protocol:

- Subject becomes pregnant before receiving the additional dose of vaccine.
- Study non-compliance to protocol requirements that in the opinion of the participating site PI or appropriate sub-investigator poses an increased risk or compromises the validity of the data.
- Lost to follow-up.
- If the subject met an exclusion criterion for participation in the study (either newly developed or not previously recognized) that precludes further study participation.
- Request of primary care provider, the IRB, FDA, or NIAID.
- Medical disease or condition, or new clinical finding(s) for which continued participation, in the opinion of the participating site PI or appropriate sub-investigator might compromise the safety of the subject, interferes with the subject's successful completion of this study, or interferes with the evaluation of responses.

- If any AE or situation occurs such that continued participation in the study would not be in the best interest of the subject.
- Any SAE judged to be related to vaccine.

If the subject agrees, every attempt will be made to follow all AEs through resolution or stabilization.

Subjects who withdraw, or are withdrawn from this study, or are lost to follow-up after signing the informed consent form (ICF) and administration of the study product will not be replaced.

Subjects who withdraw, or are withdrawn from this study, or are lost to follow-up after signing the ICF but before administration of the first study product may be replaced.

The reason for subject discontinuation or withdrawal from the study will be recorded on the appropriate DCF.

7.3 Lost to Follow-Up

A subject will be considered lost to follow-up if he or she fails to appear for a follow-up assessment. Extensive effort (i.e., generally three documented contact attempts via telephone calls, e-mail, etc., made on separate occasions) will be made to locate or recall the subject, or at least to determine the subject's health status. These efforts will be documented in the subject's study file.

8. STUDY ASSESSMENTS AND PROCEDURES

8.1 Screening and Immunogenicity Assessments

8.1.1 Screening or Enrollment/Baseline Procedures

There is a small amount of risk to subjects who report that they are in stable state of health but have an unknown health problem at the time of the enrollment/baseline visit. Screening assessments can occur up to 28 days before or at the subject's first vaccination visit (Day 1). At the screening (optional) or enrollment visit, and prior to any other study-related activities, the participating site PI or appropriate designee will provide the subject with detailed study information and will obtain written informed consent.

Some or all of the following assessments are performed during the screening (optional) or enrollment visit to determine eligibility requirements as specified in the inclusion and exclusion criteria:

- Obtain a complete medical history.
- Demographic information.
- Review pre-study medications at screening and record on the appropriate DCF (refer to Section 6.5). Review all licensed/approved and any experimental vaccines (the review of licensed/approved vaccines other than COVID-19 vaccines is limited to 28 days prior to screening/enrollment).
- Review any participation in investigational trials in the last 28 days.
- Measure vital signs (HR, BP, and oral temperature), and height and weight for determination of BMI.
- Targeted physical examination. A targeted exam is one that is targeted to assess any symptoms or medical conditions. The site PI (or designee) determines what exam is needed. An individual with no symptoms and no relevant medical history (or a medical history that is unlikely to reveal anything on medical exam) may not need any physical exam.
- Urine pregnancy test (in subjects of childbearing potential). If urine pregnancy is done at separate screening visit, repeat urine pregnancy test will be done within 24 hours of study vaccine administration.
- Review inclusion and exclusion criteria.

The screening process can be suspended prior to complete assessment at any time if exclusions are identified by the study team.

Study subjects who qualify for inclusion will be contacted and scheduled for enrollment and first vaccination within the window for enrollment unless the screening and vaccination are scheduled on the same day.

If a physiologic parameter, e.g., vital signs, is abnormal and potentially clinically significant, then the measurement may be repeated if, in the judgment of the participating site PI or appropriate designee, the abnormality is the result of an acute, short-term, rapidly reversible condition (e.g.,

stress, anxiety or "white coat syndrome") or other source of error. A physiologic parameter may also be repeated if there is a technical problem with the measurement caused by malfunctioning, or an inappropriate measuring device (i.e., inappropriate-sized BP cuff).

A subject may be re-screened if there is a transient disease status (e.g., subject complained of a "cold or fever" and met a temporary delaying enrollment criterion of acute illness or fever), or if a protocol eligibility criterion that is not met at the initial time of screening, will be met by rescreening at a later date (e.g., a medication taken within exclusionary window at the time of first screening that would not be within exclusionary window at a later rescreen).

Subjects will be provided the results of abnormal clinical findings necessitating follow-up at the discretion of the participating site PI or appropriate designee. Research laboratory results will not be provided to the subject with the exception of urine pregnancy test and SARS-CoV-2 local testing results.

The screening and first vaccination procedures both can be conducted at the enrollment visit.

8.1.2 Immunogenicity Evaluations

Humoral Immunogenicity Assays:

The following humoral immunogenicity assays may be performed:

- IgG ELISA or Multiplex MSD antibody binding assays to SARS-CoV-2 proteins (may include nucleocapsid protein, and multiple variant Receptor binding domains and variant spike proteins).
- Neutralization assays using SARS-CoV-2 variant specific S-pseudotyped viruses.
- Neutralization assay using different strains of live SARS-CoV-2.
- Characterization of antigen-specific antibody by a) subclass, b) isotype, c) ability to interact with Fc receptors, d) innate immune receptors, e) lectin-like molecules and f) lectins at different times post-vaccination.
- Functional Fc effector assays which may include quantification of antigen specific antibody-mediated NK cell activation (ADCC-NK) levels, antibody-dependent cellular phagocytosis (ADCP) activity, Antibody-dependent complement deposition (ADCD) and/or Antibody-dependent neutrophil activation/phagocytosis activity.

Preparation of blood samples and shipping instructions for serological immunogenicity assays are outlined in the protocol-specific MOP. Inability (e.g., failure of venipuncture) to collect all baseline samples on Day 1 will not exclude the subject from further participation in this study as long as a minimum of baseline serum for serological immunogenicity assays is collected (refer to protocol-specific MOP).

Cellular Immunology Assays:

This trial may also investigate innate, B and T cell immune responses using multiparametric flow cytometry. Refer to the protocol-specific immune monitoring plan for details.

Preparation of blood samples and shipping instructions for cellular immunology assays are outlined in the protocol-specific MOP.

The volume of venous blood to be collected for immunogenicity evaluations is presented in Table 2 and Table 3.

8.1.3 Samples for Suspected SARS-CoV-2 infection

In the event that a subject develops symptoms compatible with COVID-19, the site will follow with an unscheduled Illness Visit in person or by phone (with the participant self-collecting the swab). If a participant is asymptomatic but tests positive for COVID-19 outside the study, the site will follow the same procedure. Wide discretion is given to sites for the assessment of COVID-19 illness. Guidance can be found at the CDC website (2021 Case Definition):

Coronavirus Disease 2019 (COVID-19) 2021 Case Definition | CDC

The following intervention will be performed in the event of an illness visit:

- Nasal swabs for PCR and sequencing

One to two nasal swabs will be obtained for the purposes of 1) conducting qualitative analysis to assess for the presence of SARS-CoV-2 virus, and 2) conducting PCR quantitation/sequencing in the event that nasal swab #1 is positive for SARS-CoV-2. If a test outside the study was found to be positive only one swab is needed for sequencing.

The first nasal swab, as applicable, will be processed at the local level with results informing the disposition of the second nasal swab. The sites will freeze and store the second swab (refer to MOP for labeling, storage, and shipping instructions) for potential shipment to the central repository for testing.

8.1.4 Samples for Genetic/Genomic Analysis

8.1.4.1 Genetic/Genomic Analysis

DNA obtained from B-cells may be sequenced to identify B cell receptors and monoclonal antibodies. The DNA data may be used to synthesize antigen-specific antibodies to characterize antibody binding. Secondary research samples may also be used for other genomic analysis, including, but not limited to, single nucleotide polymorphisms (SNP) arrays, human leukocyte antigen (HLA) typing, transcriptomic analysis, evaluation of the immune response to the vaccine, and/or evaluation of any AE from the vaccine.

8.1.4.2 Genetic Privacy and Confidentiality

Any genetic data generated will be kept private. Informed consent permitting data sharing will be part of the consent process. Subjects will be informed that the evolution of genomic technology and analytical methods raises the risk of re-identification, even when specimens are de-identified. No data that may identify specific subjects will be kept with the genetic data.

8.1.4.3 Management of Results

All genetic testing in this protocol will be performed for research purposes only and is not performed in a Clinical Laboratory Improvement Amendments (CLIA) certified laboratory. Therefore, results will not be shared with the subjects.

8.1.5 SARS-CoV-2 infection

Confirmed SARS-CoV-2 infection at any time during the study, defined as a positive RT-PCR test performed by the site or using a non-site testing method (RT-PCR or antigen test), will be documented. While this event would occur on study, it is not a typical AE and therefore captured as an exploratory efficacy assessment.

8.1.6. Retained Samples for Biomarker Testing

For all subjects, sera will be collected at Day 4 and Day 60 (as applicable) and stored for potential biomarker assessment at a central laboratory and comparison to testing of an aliquot of sera obtained at Day 1 and Day 57, respectively

Additionally, in the event that a subject develops symptoms compatible with myocarditis and/or pericarditis or the site is notified of a diagnosis of myocarditis and/or pericarditis, occurring within 4 weeks from vaccination, the site will follow with an in-person unscheduled illness and collect serum for potential biomarker testing at a central laboratory.

The following sample will be collected:

- 4 mL SST collection for potential biomarker testing at a central laboratory

8.2 Safety and Other Assessments

All safety endpoints for this trial are obtained by reporting of adverse events.

- New symptoms will be queried with both broad open-ended questions at the Day 8 telephone call and by specifically asking about symptoms of chest pain, shortness of breath or palpitations that may represent myocarditis and/or pericarditis.
- Data on new medical conditions, doctors' office visits (outside of routine care), emergency room visits, and hospitalizations will be collected.
- Memory Aid: All subjects will complete a Memory Aid from the time of each vaccination through 7 days after each vaccination. Subjects will be asked to confirm completion of the Memory Aid and will be queried on the presence of any grade 3 symptoms. Based on the information provided, subjects may be asked to return to the clinic for evaluation. Fourteen days after each vaccination, Memory Aids are reviewed thoroughly with the subject and confirmed data recorded in the CRF.

As these are similar to licensed vaccines, there are no laboratory assessments for safety that will be done for this trial.

8.3 Adverse Events and Serious Adverse Events

8.3.1 Definition of Adverse Event (AE)

AE means any untoward medical occurrence associated with the use of an intervention in humans, whether or not considered intervention-related [21 CFR 312.32 (a)]. An AE can therefore be any unfavorable and unintended sign, symptom or disease temporally associated with the use of medicinal (investigational) product.

Any medical condition that is present at the time that the subject is screened will be considered as baseline and not reported as an AE. However, if the severity of any pre-existing medical condition increases, it should be recorded as an AE.

AEs can be further divided into solicited AEs and unsolicited AEs. Solicited AEs are those that are described in the package insert as local or systemic reactogenicity occurring in the first 7 days after vaccination. Unsolicited AEs are those events that the subject reports occurring without being queried about the specific event.

AEs will be assessed for severity and relationship to study intervention (Section 8.3.4). Reporting of all AEs, solicited and unsolicited, will occur during the period from study product administration on Day 1 through 28 days after each vaccination. After 28 days post last vaccination through the end of study, only SAEs, Protocol Specified AESIs, MAAEs, NOCMCs, and AEs leading to withdrawal from the study will be reported.

All AEs, solicited and unsolicited, will be captured on the appropriate DCF. Solicited AEs will be regarded as related to the study product and will not require separate entry into the AE log. Information to be collected for unsolicited AEs includes event description, date of onset, assessment of severity, relationship to study product and alternate etiology (assessed only by those with the training and authority to make a diagnosis and listed on the Form FDA 1572 as the participating site PI or appropriate sub-investigator), date of resolution, seriousness, and outcome. AEs occurring during the study-collection and reporting period will be documented appropriately regardless of relationship.

AEs will be followed to resolution or stabilization.

8.3.1.1 Solicited Adverse Events

Solicited AEs are anticipated local and systemic AEs for which consistent collection of information is desired. Study clinicians will follow and collect resolution information for any reactogenicity symptoms that are not resolved within 7 days.

Solicited AEs (i.e., reactogenicity) will be collected using a memory aid and confirmed data recorded on the appropriate DCF from the time of each vaccination through 7 days post each vaccination.

For this study, solicited AEs will be:

- Injection site Pain
- Injection site Erythema
- Injection site Edema/Induration
- Headache
- Fatigue
- Myalgia
- Arthralgia
- Nausea
- Fever
- Chills

8.3.1.2 Unsolicited Adverse Events

All AEs spontaneously reported by the subject and/or in response to an open question from study staff or revealed by observation, physical examination or other diagnostic procedures must be recorded on the appropriate DCF.

Unsolicited AEs of all severities will be reported from the time of study product administration through 28 days post each vaccination.

After 28 days post last vaccination through the end of study, only SAEs, AESIs, NOCMCs, MAAEs, and AEs leading to withdrawal from the study (as detailed in Section 8.3.7 and 8.3.9) will be reported.

8.3.2 Definition of Serious Adverse Event (SAE)

An SAE is defined in 21 CFR 312.32 as follows: "An AE or suspected adverse reaction is considered serious if, in the view of either the participating site PI or appropriate sub-investigator or the sponsor, it results in any of the following outcomes:

- Death,
- a life-threatening AE,
- inpatient hospitalization or prolongation of existing hospitalization,
- a persistent or significant incapacity or substantial disruption of the ability to conduct normal life functions,
- or a congenital anomaly/birth defect.

Important medical events that may not result in death, be life-threatening, or require hospitalization may be considered serious when, based upon appropriate medical judgment, they may jeopardize the patient or subject and may require medical or surgical intervention to prevent one of the outcomes listed in this definition. Examples of such medical events include allergic bronchospasm requiring intensive treatment in an emergency room or at home, blood dyscrasias or convulsions that do not result in inpatient hospitalization, or the development of drug dependency or drug abuse."

"Life-threatening" refers to an AE that at occurrence represents an immediate risk of death to a subject. An event that may cause death if it occurs in a more severe form is not considered life-threatening. Similarly, a hospital admission for an elective procedure is not considered an SAE.

All SAEs, as with any AE, will be assessed for severity and relationship to study intervention.

All SAEs will be recorded on the appropriate SAE DCF.

All SAEs will be followed through resolution or stabilization by a study clinician, licensed to make medical diagnoses and listed on the Form FDA 1572 as the participating site PI or appropriate sub-investigator.

All SAEs will be reviewed and evaluated by DMID and will be sent to the DSMB (for periodic review unless related) and IRB/IEC as needed.

8.3.3 Suspected Unexpected Serious Adverse Reactions (SUSAR)

A SUSAR is any SAE where a causal relationship with the study product is at least reasonably possible but is not listed in the Investigator's Brochure (IB), Package Insert, and/or Summary of Product Characteristics.

8.3.4 Classification of an Adverse Event

The determination of seriousness, severity and causality will be made by an on-site investigator who is qualified (licensed) to diagnose AE information, provide a medical evaluation of AEs and classify AEs based upon medical judgment. This includes, but is not limited to, physicians, physician assistants and nurse practitioners.

8.3.4.1 Severity of Adverse Events

All AEs, AESIs, NOCMCs, MAAEs, SAEs, and AEs leading to withdrawal from the study will be assessed for severity, according to the toxicity grading scales in the FDA "Toxicity Grading Scale for Healthy Adult and Adolescent Volunteers Enrolled in Preventive Vaccine Clinical Trials".

For AEs not included in the FDA-defined grading system, the following guidelines will be used to describe severity.

- Mild (Grade 1): Events that are usually transient and may require only minimal or no treatment or therapeutic intervention and generally do not interfere with the subject's usual activities of daily living.
- Moderate (Grade 2): Events that are usually alleviated with additional specific therapeutic intervention. The event interferes with usual activities of daily living, causing discomfort but poses no significant or permanent risk of harm to the research subject.
- Severe (Grade 3): Events interrupt usual activities of daily living, or significantly affect clinical status, or may require intensive therapeutic intervention. Severe events are usually incapacitating.

AEs characterized as intermittent require documentation of onset and duration of each episode. The start and stop date of each reported AE will be recorded on the appropriate DCF. Changes in the severity of an AE will be documented to allow an assessment of the duration of the event at each level of intensity.

8.3.4.2 Relationship to Study Intervention

For each reported adverse reaction, the participating site PI or qualified designee must assess the relationship of the event to the study product using the following guidelines:

- Related – The AE is known to occur with the study intervention, there is a reasonable possibility that the study intervention caused the AE, or there is a temporal relationship between the study intervention and event. Reasonable possibility means that there is evidence to suggest a causal relationship between the study intervention and the AE.
- Not Related – There is not a reasonable possibility that the administration of the study intervention caused the event, there is no temporal relationship between the study intervention and event onset, or an alternate etiology has been established.

Solicited adverse events reported in the 7 days after each vaccination are considered related to study product unless they are also recorded as an unsolicited event, in which case the relationship to study product will be determined by the PI or qualified designee.

8.3.5 Time Period and Frequency for Event Assessment and Follow-Up

For this study:

- Solicited Adverse Events will be collected for 7 days following each vaccine dose.
- Unsolicited AEs will be collected until 28 days after each vaccination.
- AESIs, NOCMCs, MAAEs, SAEs, and AEs leading to withdrawal from the study will be collected from Day 1 through the end of the study.

8.3.6 Adverse Event Reporting

8.3.6.1 Investigators Reporting of AEs

Information on reportable AEs should be recorded on the appropriate DCF. All clearly related signs, symptoms and results of diagnostic procedures performed because of an AE should be grouped together and recorded as a single diagnosis. If the AE is a clinical laboratory abnormality that is part of a clinical condition or syndrome, it should be recorded as the syndrome or diagnosis rather than the individual clinical laboratory abnormality. Each AE will also be described in terms of duration (start and stop date), severity, association with the study product, action(s) taken, and outcome.

8.3.7 Serious Adverse Event Reporting

8.3.7.1 Investigators Reporting of SAEs

Any AE that meets a protocol-defined criterion as an SAE must be submitted immediately (within 24 hours of site awareness) on an SAE form to the DMID Pharmacovigilance Group, at the following address:

DMID Pharmacovigilance Group
Clinical Research Operations and Management Support (CROMS)
6500 Rock Spring Dr. Suite 650
Bethesda, MD 20817, USA
SAE Hot Line: 1-800-537-9979 (US) or 1-301-897-1709 (outside US)
SAE FAX Number: 1-800-275-7619 (US) or 1-301-897-1710 (outside US)
SAE Email Address: PVG@dmidcroms.com

In addition to the SAE form, select SAE data fields must also be entered into Advantage eClinical. Refer to the protocol-specific MOP for details regarding this procedure.

Other supporting documentation of the event may be requested by the DMID Pharmacovigilance Group and should be provided as soon as possible. The DMID Medical Monitor will review and assess the SAE for regulatory reporting and potential impact on study subject safety and protocol conduct.

At any time after completion of the study, if the participating site PI or appropriate sub-investigator becomes aware of an SAE that is suspected to be related to study product, the participating site PI or appropriate sub-investigator will report the event to the DMID Pharmacovigilance Group.

8.3.7.2 Regulatory Reporting of SAEs

Following notification from the participating site PI or appropriate sub-investigator, DMID, as the IND sponsor, will report any SUSAR in an IND safety report to the FDA and will notify all participating site PIs (i.e., all PIs to whom the sponsor is providing drug under its IND(s) or under any PI's IND(s)) of potential serious risks from clinical studies or any other source, as soon as possible. DMID will report to the FDA any unexpected fatal or life-threatening suspected adverse reaction as soon as possible, but in no case later than 7 calendar days after the sponsor's initial receipt of the information. If the event is not fatal or life-threatening, the IND safety report will be submitted within 15 calendar days after the sponsor determines that the information qualifies for reporting as specified in 21 CFR Part 312.32. Relevant follow-up information to an IND safety report will be submitted as soon as the information is available. Upon request from FDA, DMID will submit to the FDA any additional data or information that the agency deems necessary, as soon as possible, but in no case later than 15 calendar days after receiving the request.

SAEs that are not SUSARs will be reported to the FDA at least annually in a summary format which includes all SAEs.

8.3.8 Reporting Events to Subjects

Subjects will be informed of any major safety event that occurs as part of their participation in this trial.

8.3.9 Adverse Events of Special Interest (AESIs), New Onset of Chronic Medical Conditions (NOCMCs) and Medically Attended Adverse Events (MAAEs)

Adverse Events of Special Interest (AESIs) represent any events for which additional data (besides the standard AE data) are desired. An adverse event of special interest (serious or nonserious) is one of scientific and medical concern specific to the sponsor's product or program, for which ongoing monitoring and rapid communication by the investigator to the sponsor is required. Such an event may require further investigation in order to characterize and understand it. Depending on the nature of the event, rapid communication by the trial sponsor to other parties (e.g., regulators) may also be required. These may be at the request of the regulatory agency, industry partner or DMID, and driven by a regulatory requirement, or known or potential risk from the product or class.

Protocol Specified AESIs are listed in Section 12.

New Onset of Chronic Medical Conditions (NOCMCs) are defined as any new ICD diagnosis (per current International Statistical Classification of Diseases and Related Health Problems) that is applied to the subject during the course of the study, after receipt of the study agent, that is expected to continue for at least 3 months and requires continued health care intervention.

Medically Attended Adverse Events (MAAEs) are defined as a hospitalization < 24 hours, emergency room visit or an otherwise unscheduled visit to or from medical personnel for any reason; and considered related or possibly related to study product.

All AESIs, MAAEs, NOCMCs, are assessed, recorded, and followed as described above under AEs. The following need to be reported to DMID pharmacovigilance:

- All AESIs, MAAEs or NOCMCs that meet SAE criteria (reported within 24 hours);
- Any AESIs even if not SAE (reported within 24 hours);
- MAAEs only when requested by the sponsor;
- NOCMCs only when requested by the sponsor.

As indicated, these should be reported to DMID Pharmacovigilance Group at the following address:

DMID Pharmacovigilance Group
Clinical Research Operations and Management Support (CROMS)
6500 Rock Spring Dr. Suite 650
Bethesda, MD 20817, USA
SAE Hot Line: 1-800-537-9979 (US) or 1-301-897-1709 (outside US)
SAE FAX Number: 1-800-275-7619 (US) or 1-301-897-1710 (outside US)
SAE Email Address: PVG@dmidcroms.com

8.3.10 Reporting of Pregnancy

Pregnancy is not an AE. Pregnancy outcomes will be reported on the appropriate DCF if a new pregnancy occurs within 3 months from last study vaccine dose. If pregnancy is associated with an SAE to the mother or fetus (e.g., miscarriage or preeclampsia), the SAE should be reported to PVG.

8.4 Unanticipated Problems

8.4.1 Definition of Unanticipated Problems (UPs)

The Department of Health and Human Services (DHHS) OHRP considers unanticipated problems involving risks to subjects or others to include, in general, any incident, experience, or outcome that meets all of the following criteria:

- Unexpected in terms of nature, severity, or frequency given (a) the research procedures that are described in the protocol-related documents, such as the IRB-approved research protocol and informed consent document; and (b) the characteristics of the subject population being studied;
- Related or possibly related to participation in the research (“possibly related” means there is a reasonable possibility that the incident, experience, or outcome may have been caused by the procedures involved in the research); and
- Suggests that the research places subjects or others at a greater risk of harm (including physical, psychological, economic, or social harm) than was previously known or recognized.

8.4.2 Unanticipated Problem Reporting

To satisfy the requirement for prompt reporting, UPs will be reported using the following timeline:

- UPs that are SAEs will be reported to the IRB, to the DMID Pharmacovigilance Group, and in the clinical database within 24 hours of the participating site PI or appropriate sub-investigator becoming aware of the event per the above-described SAE reporting process.
- UPs that are SAEs will be collected from Day 1 through the end of the study.
- Any other UP will be reported to the IRB and in the clinical database within 3 days of the participating site PI or appropriate sub-investigator becoming aware of the problem.

8.4.3 Reporting Unanticipated Problems to Subjects

Subjects will be informed of any UPs that occur as part of their participation in this trial.

9. STATISTICAL CONSIDERATIONS

9.1 Statistical Hypotheses

This is a phase 2, open-label, multi-site clinical trial that is not designed to test a specific hypothesis. Rather, it is intended to obtain preliminary estimates in adults of the safety and immunogenicity of additional booster doses of SARS-CoV-2 variant vaccines.

9.2 Sample Size Determination

9.2.1 Sample Size Calculation for the Safety Endpoint

Rare AEs are not demonstrable in a clinical study of this size; however, the probabilities of observing one or more AEs given various true event rates are presented in Table 5. With the assumption that all enrolled subjects will likely complete immunizations and safety visits in this relatively short duration study, the following statistical considerations apply. With approximately 100 subjects in each arm there is a greater than 99.9% chance of observing at least one AE of probability 10%. With approximately 50 subjects in each of the strata subgroups, there is a 99.5% chance of observing at least one AE of probability 10%. With approximately 25 subjects in each of the strata subgroup combinations (assuming equal numbers), there is a 92.8% chance of observing at least one AE of probability 10%. Finally, with approximately 15 subjects in the smallest of the strata subgroup combinations (assuming unequal numbers), there is a 79.4% chance of observing at least one AE of probability 10%. Therefore, if no AEs of a given type occur in a group (or strata), we can be relatively confident that they will occur in fewer than 10% of people if the vaccine is implemented.

Table 5: Probability of Observing one or more Adverse Event for Various Event Rates in one vaccine group (or strata).

N	"True" Event Rate	Probability of Observing ≥ 1 events (%)	N	"True" Event Rate	Probability of Observing ≥ 1 events (%)
100	0.1%	9.5	50	0.1%	4.9
	0.5%	39.4		0.5%	22.2
	1.0%	63.4		1.0%	39.5
	2.0%	86.7		2.0%	63.6
	3.0%	95.2		3.0%	78.2
	4.0%	98.3		4.0%	87
	5.0%	99.4		5.0%	92.3
	10.0%	>99.9		10.0%	99.5
	15.0%	>99.9		15.0%	>99.9
	20.0%	>99.9		20.0%	>99.9
	30.0%	>99.9		30.0%	>99.9

N	"True" Event Rate	Probability of Observing ≥ 1 events (%)	N	"True" Event Rate	Probability of Observing ≥ 1 events (%)
25	0.1%	2.5	15	0.1%	1.5
	0.5%	11.8		0.5%	7.2
	1.0%	22.2		1.0%	14
	2.0%	39.7		2.0%	26.1
	3.0%	53.3		3.0%	36.7
	4.0%	64.0		4.0%	45.8
	5.0%	72.3		5.0%	53.7
	10.0%	92.8		10.0%	79.4
	15.0%	98.3		15.0%	91.3
	20.0%	99.6		20.0%	96.5
	30.0%	>99.9		30.0%	99.5

9.2.2 Sample Size Calculation for the Immunogenicity Endpoints

The primary objective of this study is to evaluate the magnitude and durability of SARS-CoV-2 specific antibody titers in serum samples. This objective is descriptive in nature and will be accomplished by estimating 95% confidence intervals (CI) for the geometric mean titer (GMT) at each timepoint when samples are collected.

The precision with which the GMT can be estimated from observed data depends on the standard deviation (SD) of the measurements, on the logarithmic scale, and the sample size. Table 6 displays two-sided 95% confidence intervals for the GMT for several values of the observed antibody titer. Table 6 also shows results assuming up to 10% attrition.

Table 6: Two-sided 95% confidence intervals based on observing a particular average $\log_e$-antibody titer in subjects' vaccine groups and potential strata sizes

Observed average $\log_e$ antibody titer	SD of $\log_e$ antibody titer	95% confidence interval of GMT in vaccine groups and strata			
		N = 100	N = 50	N = 25	N = 15
$\log_e$ (5)	0.5	(4.5, 5.5)	(4.3, 5.8)	(4.1, 6.1)	(3.8, 6.6)
$\log_e$ (20)		(18.1, 22.1)	(17.4, 23.1)	(16.3, 24.6)	(15.2, 26.4)
$\log_e$ (50)		(45.3, 55.2)	(43.4, 57.6)	(40.7, 61.5)	(37.9, 66)
$\log_e$ (100)		(90.6, 110.4)	(86.8, 115.3)	(81.4, 122.9)	(75.8, 131.9)
$\log_e$ (250)		(226.4, 276.1)	(216.9, 288.2)	(203.4, 307.3)	(189.5, 329.8)
$\log_e$ (500)		(452.8, 552.1)	(433.8, 576.3)	(406.8, 614.6)	(379.1, 659.5)
$\log_e$ (1000)		(905.6, 1104.3)	(867.5, 1152.7)	(813.5, 1229.2)	(758.1, 1319)
$\log_e$ (5)	1.0	(4.1, 6.1)	(3.8, 6.6)	(3.3, 7.6)	(2.9, 8.7)

Observed average $\log_e$ antibody titer	SD of $\log_e$ antibody titer	95% confidence interval of GMT in vaccine groups and strata			
		N = 100	N = 50	N = 25	N = 15
$\log_e$ (20)		(16.4, 24.4)	(15.1, 26.6)	(13.2, 30.2)	(11.5, 34.8)
$\log_e$ (50)		(41, 61)	(37.6, 66.4)	(33.1, 75.6)	(28.7, 87)
$\log_e$ (100)		(82, 121.9)	(75.3, 132.9)	(66.2, 151.1)	(57.5, 174)
$\log_e$ (250)		(205, 304.9)	(188.2, 332.2)	(165.5, 377.8)	(143.7, 435)
$\log_e$ (500)		(410, 609.7)	(376.3, 664.3)	(330.9, 755.5)	(287.4, 869.9)
$\log_e$ (1000)		(820, 1219.5)	(752.6, 1328.7)	(661.8, 1511)	(574.8, 1739.8)

* Assumes approximately 10% attrition.

9.3 Populations for Analyses

The safety analysis population includes all enrolled subjects who received at least one dose of study vaccine. Analyses for the safety population will include safety reported through the end of the study. The modified intent-to-treat (mITT) population includes all subjects who received at least one dose of vaccine and contributed both pre- and at least one post-vaccination venous blood sample for immunogenicity testing for which valid results were reported.

In the final analysis, protocol deviations will be reviewed to determine which protocol deviations may affect the analysis. The per protocol (PP) population will then be defined – and this includes all subjects in the mITT subset with the following exclusions:

- Data from all available visits for subjects found to be ineligible at baseline.
- Data from all visits subsequent to the protocol deviations that are considered to affect the science.
- Data from any visit that occurs substantially out of window.

9.4 Statistical Analyses

Interim analyses of safety and immunologic response data will be done, as needed.

The final analysis will be performed after the final data lock and clinical study report (CSR) completed when all primary safety endpoint data and all secondary immunogenicity endpoint data are available and received by the SDCC. Any available data from the exploratory immunogenicity endpoints may also be included in the CSR. Remaining exploratory immunogenicity endpoint data may be included in an addendum to the CSR, publication of manuscript(s), or other report(s). Abbreviated analysis plans that describe planned analyses to facilitate dissemination of study data for public health reasons, including manuscript publication(s), will be developed by the SDCC. A full statistical analysis plan (SAP) will be developed by the SDCC and finalized prior to the primary data lock.

9.4.1 General Approach

Unless otherwise noted in the SAP, continuous variables will be summarized using the following descriptive statistics: n (non-missing sample size), mean, standard deviation, median, maximum and minimum. The frequency and percentages (based on the non-missing sample size) of observed levels will be reported for all categorical measures.

9.4.2 Analysis of the Primary Endpoint(s)

Descriptive summaries of immunogenicity data will be presented for the mITT population. If there are protocol deviations which may affect the analysis, a per-protocol (PP) analysis may also be performed.

Geometric Mean Titers (GMT) and Geometric Mean Fold Rise (GMFR) from baseline of SARS-CoV-2 specific antibody binding and neutralization titers will be calculated, along with 95% CIs, for all groups, at each timepoint. Summaries will also be displayed graphically. Geometric Mean Ratio is defined as the ratio of a variant of concern (i.e Beta to D614G) will also be reported for all groups, at each timepoint, along with 95% CIs.

9.4.3 Analysis of the Secondary Endpoints

Section 9.4.4 describes the analyses of Safety Endpoints, which is the secondary endpoint of this protocol.

9.4.4 Safety Analyses

Summaries and analysis of safety data will be presented for the Safety Analysis Population.

Solicited non serious AEs will be collected from the time of each vaccination until 7 days after. Solicited AEs will be summarized by severity for each day post vaccination (Days 1-8) and as the maximum severity over all 8 days. Additionally, solicited AEs will be analyzed using standard techniques, such as exact confidence intervals (CI), to summarize the proportion of subjects reporting each symptom, any application site symptom, and any systemic symptom.

Unsolicited non-serious AEs will be collected from the time of first vaccination through 28 days after each vaccination. Unsolicited AEs will be coded by MedDRA for preferred term and system organ class (SOC). SAEs, MAAEs, NOCMCs, AESIs, and AEs leading to withdrawal from the study will be collected from the time of first vaccination through 12 months after the last vaccine dose. The numbers of SAEs, MAAEs, NOCMCS, AESIs, and AEs leading to withdrawal from the study will be reported by detailed listings showing the event description, MedDRA preferred term and SOC, relevant dates (vaccinations and AEs), severity, relatedness, and outcome for each event. Non-serious unsolicited AEs will be summarized as number and percentage of subjects reporting at least one event in each MedDRA preferred term and SOC, cross tabulated by severity and relationship to study product. Additionally, the proportion of subjects and exact 95% CIs of AEs in aggregate and by MedDRA categories will be computed.

9.4.5 Baseline Descriptive Statistics

Summaries of demographic variables such as age, sex, ethnicity, race, and prior infection status will be presented by vaccination arm and overall.

9.4.6 Planned Interim and Early Analyses

Data may be disseminated to public health officials and partners as needed and included in publications and presentations to inform the global scientific community. Early analyses will include safety and immunogenicity as described in Sections 9.4.6.1, 9.4.6.2, and 9.4.6.3. Further, the protocol team will review data periodically to confirm no halting criteria have been met as described in Section 7.1.1.

Cumulative safety information, study status, and primary endpoint results may be published, presented at a public forum, or presented as summaries aggregated by study arm at the discretion of the sponsor while the study is ongoing. None of the interim analyses will include any formal statistical hypothesis testing; therefore, p value adjustment will not be made to any analyses.

9.4.6.1 Interim Safety Analyses

Given the need for rapid review and dissemination of study data for public health reasons, AEs and SAEs may be reviewed as necessary outside of DSMB reviews. The DSMB may not need to meet (unless halting rules are met), and materials will be provided electronically. Documentation of review and any concerns noted will be solicited electronically.

The DSMB will review separate cumulative AE data reports for all subjects within each arm. Given the safety database known for authorized/approved vaccines, there is no routine mandatory review by the DSMB unless halting rules are triggered.

9.4.6.2 Interim Immunogenicity Review

Interim data review of immunogenicity will be performed as often as needed to inform public health decisions.

Statistical analyses of immunogenicity endpoints, by vaccine arm, may be performed when subjects have completed key immunogenicity visits. Immunogenicity reviews may be shared with the DSMB, as determined by DMID.

Data may be disseminated to public health officials and partners as needed and included in publications and presentations to inform the global scientific community.

9.4.6.3 Interim Immunogenicity and Safety Review

Interim analyses of safety, reactogenicity, and immunologic response data may be done, as needed.

9.4.7 Sub-Group Analyses

Subgroup analyses, by age group and infection status, may be performed. Detailed information will be provided in the Statistical Analysis Plan.

9.4.8 Tabulation of Individual Subject Data

In general, all data will be listed, sorted by vaccine arm and subject, and when appropriate by visit number within subject.

9.4.9 Exploratory Analyses

Summaries and analysis of immunologic assay data will be presented for the mITT population. If there are protocol deviations which may affect the analysis, a PP analysis may also be performed.

10. SUPPORTING DOCUMENTATION AND OPERATIONAL CONSIDERATIONS

10.1 Regulatory, Ethical, and Study Oversight Considerations

This study will be conducted in conformity with the principles set forth in The Belmont Report: Ethical Principles and Guidelines for the Protection of Human Subjects of Research (US National Commission for the Protection of Human Subjects of Biomedical and Behavioral Research; April 18, 1979), and the federal policy for the Protection of Human Subjects codified in 45 CFR Part 46, 21 CFR Part 50 (Protection of Human Subjects), and the ICH E6(R2).

An OHRP-registered IRB will review and approve this protocol, associated informed consent documents, recruitment materials, and handouts or surveys intended for the subjects, prior to the recruitment, screening and enrollment of subjects. The IRB review shall be in accordance with 45 CFR 46 and 21 CFR 50, 21 CFR 56 (IRBs), and other federal, state, and local regulations and policies, as applicable.

Each institution engaged in this research will hold an OHRP-approved FWA.

Any amendments to the protocol or informed consent documents will be approved by the IRB before they are implemented. IRB review and approval will occur at least annually throughout the duration of the study. The participating site PI will notify the IRB of deviations from the protocol and reportable SAEs, as applicable to the IRB policy.

DMID must receive the documentation that verifies IRB approval for this protocol, informed consent documents and associated documents, prior to the recruitment, screening and enrollment of subjects, and any IRB approvals for continuing review or amendments as required by the DMID.

10.1.1 Informed Consent Process

Informed consent is a process that is initiated prior to an individual agreeing to participate in a trial and continuing throughout the individual's trial participation. Investigators or designated research staff will obtain a subject's informed consent in accordance with the requirements of 45 CFR 46, 21 CFR 50 and 21 CFR 56 for FDA-regulated studies, state and local regulations and policy, and ICH E6 GCP before any study procedures or data collection are performed. The participating site PI or other study staff may obtain oral or written information for the purpose of screening, recruiting, or determining the eligibility of prospective subjects without the informed consent of the prospective subject if the process is approved by the IRB.

At the first study visit, informed consent will be obtained and documented before any study procedures are performed. Subjects will receive a concise and focused presentation of key information about the clinical trial, verbally and with a written consent form. The key information about the purpose of the study, the procedures and experimental aspects of the study, study interventions/products, risks and discomforts, the expected duration of the subject's participation in the trial, any expected benefits to the subject, and alternative treatments and procedures that may be available to the subject. The explanation will be organized and presented in lay terminology and language that facilitates understanding why one might or might not want to participate.

Subjects will receive an explanation as to whether any compensation and any medical treatments are available if injury occurs, and, if so, what they consist of, or where further information may be obtained. Subjects will be informed of the anticipated financial expenses, if any, to the subject for participating in the trial, as well as any anticipated prorated payments, if any, to the subject for participating in the trial. They will be informed of whom to contact (e.g., the participating site PI) for answers to any questions relating to the research project. Information will also include the foreseeable circumstances and/or reasons under which the subject's participation in the trial may be terminated. The subjects will be informed that participation is voluntary and that they are free to withdraw from the study for any reason at any time without penalty or loss of benefits to which the subject is otherwise entitled. Subjects will be allowed sufficient time to consider participation in this research trial and have the opportunity to discuss this trial with their family, friends or legally authorized representative, or think about it prior to agreeing to participate.

Subjects will be informed that records identifying the subject will be kept confidential, and, to the extent permitted by the applicable laws and/or regulations, will not be made publicly available. If the results of the trial are published, the subject's identity will remain confidential. Subjects will be informed, even if identifiers are removed, that information collected from this research and/or specimens may be used for secondary research, including the sharing of deidentified data.

Subjects will be informed that the monitor(s), auditors(s), IRB, NIAID, and regulatory authority(ies) will be granted direct access to the subject's original medical records for verification of clinical trial procedures and/or data without violating the confidentiality of the subject, to the extent permitted by the applicable laws and regulations, and that, by signing a written ICF, the subject is authorizing such access.

ICFs will be IRB-approved, and subjects will be asked to read and review the consent form. Subjects must sign the ICF prior to starting any study procedures being done specifically for this trial. Once signed, a copy of the ICF will be given to the subject for their records.

New information will be communicated by the participating site PI or designee to subjects who consent to participate in this trial in accordance with IRB requirements. The informed consent document will be updated, and subjects will be re-consented per IRB requirements, if necessary.

10.1.1.1 Requirements for Permission by Parents/Guardians and Assent by Children (in case of a minor)

Not Applicable

10.1.1.2 Other Informed Consent Procedures

The rights and privacy of human subjects who participate in genomic or phenotypic research studies will be protected at all times. The consent process, including relevant language in the ICF, will provide an explanation of the potential risks to the individual study subjects and their families. Clinical metadata, genomic, or other datasets or a subset of the clinical and other metadata that may potentially identify human subjects will not be released in unrestricted databases. Subjects will be informed that the evolution of genomic technology and analytical methods raises the risk of re-identification, even when specimens are de-identified.

Subjects will be asked for consent to collect additional blood, nasal swabs, the use of residual specimens, and the sharing of genetic information and samples for secondary research. This extra/residual blood and corresponding serum, plasma and PBMCs will be used as back-up specimens for PP defined assays or designated for secondary research use and stored indefinitely at a designated storage facility.

Subjects will be asked to consent specifically to genetic testing on primary and secondary research samples, including but not limited to transcriptomics and DNA sequencing. DNA sequencing data will be kept private. DNA data may be used to produce commercial antibody-based therapeutics. Subjects will not share in profits or commercial rights to those products.

If subjects choose not to provide permission for extra blood and secondary research use, they will not be eligible for enrollment into the study.

Collection of extra/residual samples during the course of the study will help facilitate rapid follow-on analyses, if warranted, to provide more comprehensive scientific insights into the impact (safety and immunological) of the vaccine on the host response to vaccination. To maintain statistical power in follow-on analyses it is important that extra blood collection and secondary research use be included in as many subjects as possible, due to the limited sample size per treatment arm.

The stored samples will be labeled with barcodes to maintain confidentiality. Research with identifiable samples and data may occur as needed, however, subject confidentiality will be maintained as described for this protocol and with IRB approval.

Samples designated for secondary research use may be used for additional immunological assessments that may include but are not limited to antibody epitope mapping, B and T cell repertoire determination, non-traditional immune assay development, determination of innate immune factors and the ability of vaccine-induced antibodies to cross-react to different proteins and virus strains. These blood samples might be used in new or different immunological laboratory tests, to provide information for the development of new vaccines or therapeutics, or for the studies of SARS-CoV-2 or other infections. Secondary research using DNA may also be warranted to understand genetic factors involved in vaccination failures.

Samples will not be sold for commercial profit. Although the results of any future research may be patentable or have commercial profit, subjects will have no legal or financial interest in any commercial development resulting from any future research.

There are no direct benefits to the subject for extra specimens collected or from the secondary research. No results from secondary research will be entered into the subject's medical record. Incidental findings will not be shared with the subject, including medically actionable incidental findings, unless required by law.

Risks are associated with the additional volume of blood collected, such as anemia. Risks for loss of privacy and confidentiality are described below.

Subjects may withdraw permission to use samples for secondary use at any time. They will need to contact the participating site and the samples will be removed from the study repository after this study is completed and documentation will be completed that outlines the reason for withdrawal of permission for secondary use of samples. Subjects who withdraw consent before the last visit will not have the extra blood drawn for secondary use.

Human Genetic Testing

The research staff will seek the subjects' consent for extra and residual specimens to be stored and used for secondary research, including genetic research, evaluating human genomic and phenotypic markers. The rights and privacy of human subjects who participate in genomic or phenotypic research studies will be protected at all times.

The consent process will include an explanation of the potential risks to the individual subjects and their families associated with data submitted to an NIH data repository and subsequent sharing. Data that may potentially identify human subjects will not be released in unrestricted databases. Subjects will be informed that the evolution of genomic technology and analytical methods raises the risk of re-identification, even when specimens are de-identified. The consent will include whether individual subject data will be shared through a NIH controlled access data repository. Data for genomic or phenotypic research will be submitted to a controlled access data repository, therefore, informed consent permitting the data sharing must be documented, even if the specimens are de-identified.

10.1.2 Study Termination and Closure

In Section 7, Study Intervention Discontinuation and Subject Discontinuation/Withdrawal, describes the temporary halting of the study.

This study may be prematurely terminated if there is sufficient reasonable cause, including, but not limited to:

- Determination of unexpected, significant, or unacceptable risk to subjects
- Results of interim analysis
- Insufficient compliance to protocol requirements
- Data that are not sufficiently complete and/or not evaluable
- Regulatory authorities

If the study is prematurely terminated, the PI or designee will promptly inform study subjects and the IRB will be notified. The PI will assure appropriate follow-up for the subjects, as necessary.

The sponsor will notify regulatory authorities as applicable.

10.1.3 Confidentiality and Privacy

Subject confidentiality is strictly held in trust by the participating investigators, their staff, and the sponsor(s) and their agents. This confidentiality is extended to cover clinical information relating to subjects, test results of biological samples and genetic tests, and all other information generated during participation in the study. No identifiable information concerning subjects in the study will be released to any unauthorized third party. Subject confidentiality will be maintained when study results are published or discussed in conferences.

The study monitor, other authorized representatives of the sponsor, representatives of the IRB, and/or regulatory agencies may inspect all documents and records required to be maintained by the participating site PI, including, but not limited to, medical records (office, clinic, or hospital)

and pharmacy records for the subjects in this study. The participating site will permit access to such records.

All source records, including electronic data, will be stored in secured systems in accordance with institutional policies and federal regulations.

All study data and research specimens that leave the participating site (including any electronic transmission of data) will be identified only by a coded number that is linked to a subject through a code key maintained at the participating site. Names or readily identifying information will not be released unless DMID approves and it aligns with the consent form, or according to laws for required reporting.

Because it may be possible to re-identify de-identified genomic data, even if access to data is controlled and data security standards are met, confidentiality cannot be guaranteed, and re-identified data could potentially be used to discriminate against or stigmatize subjects, their families, or groups. In addition, there may be unknown risks.

As this research is funded by the NIH, it is covered by NIH policy which effectively issues the research a Certificate of Confidentiality (COC). By this policy, researchers cannot be forced to disclose or provide, in any Federal, State, or local civil, criminal, administrative, legislative, or other proceeding, the name of such individual or any such information, document, or biospecimen that contains identifiable, sensitive information about the individual and that was created or compiled for purposes of the research, unless such disclosure or use is made with the consent of the individual to whom the information, document, or biospecimen pertains.

The Certificate cannot be used to resist a demand for information from personnel of the United States Government that is used for auditing or evaluation of federally funded projects, like this study, or for information that must be released in order to meet the requirements of the FDA.

A COC does not prevent subjects from voluntarily releasing information about themselves or their involvement in this research. If any person or agency obtains a written consent to receive research information, then the researchers may not use the Certificate to withhold that information.

The COC does not prevent the researchers from reporting, without the subject’s consent, information that would identify the subject as a subject in the research project in the case of matters that must be legally reported, including child and elder abuse, sexual abuse, or wanting to harm themselves or others.

The release of individual private information or specimens for other research will only occur if consent was obtained from the individual to whom the information, document, or biospecimen pertains, or that the release is in compliance with applicable Federal regulations governing the protection of human subjects in research.

10.1.4 Secondary Use of Stored Specimens and Data

Secondary Human Subject Research is the re-use of identifiable data or identifiable biospecimens that were collected from some other ‘‘primary’’ or ‘‘initial’’ activity, such as the data and samples collected in this protocol. This section will detail the samples and data available for secondary research. Any use of the secondary sample or data, however, will be presented in a separate protocol and require separate IRB approval.

10.1.4.1 Samples for Secondary Research

The following types of samples will be stored and used for secondary research:

- Residual Research Sample: Any leftover Primary Research Sample after the laboratory testing specified in this protocol is completed will be stored for future studies with the subject's consent.
- Repository Research Sample: Samples will be collected with the subject's consent in this protocol with the intent to store for additional research (i.e., samples collected beyond those needed for primary research) and will be used in future studies. Amendments to this protocol with additional assays may use repository research samples.

Samples will be stored indefinitely at a DMID-designated storage facility. Each sample will be encoded (labeled) only with a barcode and a unique tracking number to protect subject confidentiality. Secondary research with coded samples and data may occur, however, subject confidentiality will be maintained as described for this protocol. An IRB review of the secondary research using coded specimens is required.

Residual/Repository Research Samples, upon written request and approval from DMID and any approvals required by the site or network, may be shared for secondary research with investigators at the participating site, with researchers at other sites or other institutions, or company-designated research laboratories. The samples will not be sold or used directly for production of any commercial product. DMID will authorize shipment from the DMID CMS.

The subject's decision can be changed at any time by notifying the study doctors or nurses in writing. To participate in this study, subjects must consent for storage of samples for secondary use. If the subject subsequently changes his/her decision, the samples will be destroyed if the samples have not been used for research or released for a specific research project.

10.1.4.2 Data Sharing for Secondary Research

Data from this study may be used for secondary research. All of the individual subject data collected during this study will be made available after de-identification. The SAP and Analytic Code will also be made available. Data will be available immediately following publication, with no end date. Upon written request, with provision of a methodologically sound proposal, and approval from DMID and any approvals required by the site or network, data may be shared for secondary research with investigators/researchers. The data will be available for only the purpose outlined in the approved proposal.

For access to genomic data in the NIH designated controlled access database, an investigator (or data requestor) must submit a Data Access Request which certifies adherence to the NIH Security Best Practices for Controlled-Access data subject to the NIH Genomic Data Sharing (GDS) Policy.

The participating site PI may request removal of data on individual study subjects from NIH data repositories in the event that a research subject withdraws or changes his or her consent. However, some data that have been distributed for approved research use cannot be retrieved.

10.1.5 Key Roles and Study Governance

This study is sponsored by DMID. Key Roles are noted in the protocol-specific MOP.

10.1.6 Safety Oversight

10.1.6.1 Data Safety Monitoring Board (DSMB)

The DSMB is an independent group of at least three experts that monitors subject safety and advises DMID. DSMB members will be separate and independent of study staff participating in this trial and should not have scientific, financial, or other conflicts of interest related to this trial. The DSMB will consist of members with appropriate expertise to contribute to the interpretation of data from this trial. A quorum will consist of a simple majority.

The DSMB will hold an organizational meeting or electronic review prior to enrollment. At this meeting, the DSMB will review the charter, protocol, ICF, IBs, and safety report templates.

The DSMB will not need to meet unless halting rules are met. The DSMB will be provided periodic updates on enrollment, safety, and immunogenicity. Documentation of review and any concerns noted will be solicited electronically.

Ad hoc reviews will occur when trial halting criteria are met, or as requested by the sponsor or PI.

Procedures for DSMB reviews/meetings will be defined in the DSMB charter. The DSMB will review applicable data, including, but not limited to, enrollment, demographics, dosing data, clinical laboratory data, and safety data, at scheduled timepoints during this trial as defined in the DSMB charter.

Additional data may be requested by the DSMB, and interim statistical reports may be generated as deemed necessary and appropriate by DMID. As an outcome of each review/meeting, the DSMB will make a recommendation as to the advisability of proceeding with study product administration, and to continue, modify, or terminate this trial.

10.1.7 Clinical Monitoring

Clinical site monitoring is conducted to ensure that the rights and well-being of trial subjects are protected, that the reported trial data are accurate, complete, and verifiable. Clinical Monitoring also ensures conduct of the trial is in compliance with the currently approved protocol/ amendment(s), ICH, GCP, and with applicable regulatory requirement(s) and sponsor requirements. Clinical monitoring will also verify that any critical study procedures are completed following specific instructions in the protocol-specific MOP.

Monitoring for this study will be performed by DMID. Details of clinical site monitoring are documented in a CMP. The CMP describes in detail who will conduct the monitoring, at what frequency monitoring will be done, at what level of detail monitoring will be performed, and the distribution of monitoring reports. Monitoring visits will include, but are not limited to, review of regulatory files, accountability records, electronic case report forms (eCRFs), ICFs, medical and laboratory reports, site study intervention storage records, training records, and protocol and GCP compliance. Site monitors will have access to each participating site, study staff and all study documentation according to the DMID-approved CMP. Study monitors will meet with all participating site PIs to discuss any problems and outstanding issues and will document site visit findings and discussions.

10.1.8 Quality Control (QC) and Quality Assurance (QA)

To ensure the reliability of study data, each participating site will develop a Clinical Quality Management Plan (CQMP). The CQMP will describe:

- routine internal QC and QA activities for the purposes of measuring, documenting and reporting study conduct, protocol adherence, human subjects' protections, and reliability of the protocol-driven data collected and independent of sponsor site monitoring.
- a process for addressing data quality issues (i.e., collecting, recording) or systemic issues (i.e., protocol conduct, non-compliance, human subject protections), and implementation and evaluation of Corrective and Preventative Action Plan (CAPA) procedures as needed.

10.1.9 Data Handling and Record Keeping

10.1.9.1 Data Collection and Management Responsibilities

Data collection is the responsibility of the study staff at the participating site under the supervision of the participating site PI. The participating site PI must maintain complete and accurate source documentation.

Clinical research data from source documentation, including, but not limited to, AEs/SAEs, concomitant medications, medical history, physical assessments, and clinical laboratory data, will be entered by the participating site into eCRFs via a 21 CFR Part 11-compliant internet data entry system provided by Emmes. The data system includes password protection and internal quality checks, such as automatic range checks, to identify data that appear inconsistent, incomplete, or inaccurate. AEs and concomitant medications will be coded according to the most current versions of MedDRA and WhoDrug, respectively.

The SDCC will be responsible for data management, quality review, analysis, and reporting of the study data.

The IND sponsor is responsible for review of data collection tools and processes, and review of data and reports.

AEs will be coded according to the MedDRA dictionary version 23.0 or higher.

A separate study specific Study Data Standardization Plan (SDSP) appendix will be developed which describes the technical recommendations for the submission of human study data and related information in a standardized electronic format throughout product development.

At the end of the study, a copy of all datasets, including annotated CRFs and data dictionary, will be provided to DMID.

10.1.9.2 Study Record Retention

Study-related records, including the regulatory file, study product accountability records, consent forms, subject source documents and electronic records, should be maintained for a period of 2 years following the date a marketing application is approved for the investigational product for the indication for which it is being investigated; or, if no application is to be filed or if the application is not approved for such indication, until 2 years after the investigation is discontinued and FDA is notified. These documents should be retained for a longer period, however, if required by local policies or regulations. No records will be destroyed without the written consent of DMID. Consent forms with specimen retention linked to identifiable specimens will be maintained for as

long as the specimens remain in identifiable format, and a minimum of three years after use of the identifiable specimens in nonexempt human subject research.

10.1.9.3 Source Records

Source data are all information in original records (and certified copies of original records) of clinical findings, observations, or other activities in a clinical trial necessary for the reconstruction and evaluation of the trial. Source data should be attributable, legible, contemporaneous, original, accurate, and complete. Each participating site will maintain appropriate medical and research records for this trial, in compliance with ICH GCP, regulatory, and institutional requirements. Study data will be collected on paper CRFs and entered the eCRF or data will be entered directly into the eCRF. Data recorded in the eCRF derived from source documents should be consistent with the data recorded on the source documents. Data entered directly into the eCRFs will be considered the source document.

Interview of subjects is sufficient for obtaining medical history. Solicitation of medical records from the subject's primary care provider is not required.

10.1.9.4 Protocol Deviations

A protocol deviation is any non-compliance with the clinical trial protocol, any process that is noted in the protocol and refers to details in the protocol-specific MOP or GCP requirements, or any critical study procedures with specific instructions in ancillary documents referenced in the protocol such as a protocol-specific MOP.

The non-compliance may be either on the part of the subject, the participating site PI or the study staff. Following a deviation(s), corrective actions should be developed by the participating site and implemented promptly. All individual protocol deviations will be addressed in subject study records.

It is the responsibility of the participating site PI and study staff to use continuous vigilance to identify and report deviations within five working days of identification of the protocol deviation, or within five working days of the scheduled protocol-required activity. All deviations must be promptly reported to DMID per the protocol deviation reporting procedures. Protocol deviations must be sent to the local IRB/IEC per their guidelines. The participating site PI and study staff are responsible for knowing and adhering to their IRB requirements. A completed copy of the DMID Protocol Deviation Form must be maintained in the Regulatory File, as well as in the subject's chart if the deviation is subject specific.

10.1.9.5 Data Sharing Plan

Data will be available immediately following publication, with no end date, with data sharing at the discretion of the PI.

10.1.9.6 Genomic Data Sharing (GDS) Plan

This study will comply with the NIH GDS Policy, which applies to all NIH-funded research that generates large-scale human or non-human genomic data, as well as the use of these data for subsequent research. Large-scale data include genome-wide association studies (GWAS), SNP arrays, and genome sequence, transcriptomic, epigenomic, and gene expression data.

10.1.9.7 Publication

At intervals throughout the study at the discretion of the sponsor and following completion of the study, the results of this research will be published in a scientific journal. This study will adhere to the NIH Public Access Policy, which ensures that the public has access to the published results of NIH funded research. As such, the final peer-reviewed journal manuscripts will be accessible to the public on PubMed Central no later than 12 months after publication.

10.1.9.8 Conflict of Interest Policy

The independence of this study from any actual or perceived influence, such as by the pharmaceutical industry, is critical. Therefore, any actual conflict of interest of persons who have a role in the design, conduct, analysis, publication, or any aspect of this trial will be disclosed and managed. Furthermore, persons who have a perceived conflict of interest will be required to have such conflicts managed in a way that is appropriate to their participation in the design and conduct of this trial. DMID has established policies and procedures for all study team members to disclose all conflicts of interest and will establish a mechanism for the management of all reported dualities of interest.

10.2 Additional Considerations

10.2.1 Research Related Injuries

For any potential research related injury, the participating site PI or designee will assess the subject. Study staff will try to reduce, control and treat any complications from this trial. Immediate medical treatment may be provided by the participating site, such as giving emergency medications to stop immediate allergic reactions to the vaccine. As needed, referrals to appropriate health care facilities will be provided to the subject. The participating site PI should then determine if an injury occurred as a direct result of the tests or treatments that are done for this trial.

If it is determined by the participating site PI that an injury occurred to a subject as a direct result of the tests or treatments that are done for this trial, then referrals to appropriate health care facilities will be provided to the subject. Study staff will try to reduce, control and treat any complications from this trial. Immediate medical treatment may be provided by the participating site, such as giving emergency medications to stop immediate allergic reactions to the vaccine. No financial compensation will be provided to the subject by NIAID, NIH, the vaccine manufacturer, or the participating site for any injury suffered due to participation in this trial.

For this protocol, the study vaccines are covered under the PREP Act, as described in Section 2.1.1.

10.3 Abbreviations

Table 7: Abbreviations

ACIP	Advisory Committee on Immunization Practices
Ad	Adenovirus

ADCC-NK	Antibody-Mediated Natural Killer
ADCD	Antibody-Dependent Complement Deposition
ADCP	Antibody-Dependent Cellular Phagocytosis
AE	Adverse Event
AESI	Adverse Event of Special Interest
PBMC	Peripheral Blood Mononuclear Cell
BMI	Body Mass Index
BP	Blood Pressure
°C	Degrees Celsius
CAPA	Corrective and Preventative Action Plan
CD4	Cluster of Differentiation 4
CDC	Centers for Disease Control and Prevention
CFR	Code of Federal Regulations
CI	Confidence Interval
CICP	Countermeasures Injury Compensation Program
CLIA	Clinical Laboratory Improvement Amendments
CMP	Clinical Monitoring Plan
CMS	Clinical Material Services
COC	Certificate of Confidentiality
CoV	Coronavirus
COVID-19	Coronavirus Disease 2019
CRF	Case Report Form
CROMS	Clinical Research Operations and Management Support

CSR	Clinical Study Report
CQMP	Clinical Quality Management Plan
DCF	Data Collection Form
DHHS	Department of Health and Human Services
DMID	Division of Microbiology and Infectious Diseases
DNA	Deoxyribonucleic Acid
DSMB	Data and Safety Monitoring Board
DSPC	1,2-distearoyl-*sn*-glycero-3-phosphocholine
ECG	Electrocardiogram
eCRF	Electronic Case Report Form
EDC	Electronic Data Capture
EKG	Electrocardiogram
ELISA	Enzyme-Linked Immunosorbent Assay
EUA	Emergency Use Authorization
°F	Degrees Fahrenheit
FDA	Food and Drug Administration
FWA	Federal Wide Assurance
GCP	Good Clinical Practice
GDS	Genomic Data Sharing
GMFR	Geometric Mean Fold Rise
GMT	Geometric Mean Titer
GWAS	Genome-Wide Association Studies
β-HCG	β Human Chorionic Gonadotropin

HHS	Health and Human Services
HIV	Human Immunodeficiency Virus
HLA	Human Leukocyte Antigen
HR	Heart Rate
HRSA	Health Resources and Services Administration
IB	Investigator's Brochure
ICD	International Classification of Diseases
ICF	Informed Consent Form
ICH	International Council for Harmonisation
IDCRC	Infectious Disease Clinical Research Consortium
IDE	Investigational Device Exemption
IEC	Independent or Institutional Ethics Committee
IM	Intramuscular
IND	Investigational New Drug Application
IRB	Institutional Review Board
IV	Intravenous
kg	Kilogram
LNP	Lipid Nanoparticle
MAAE	Medically Attended Adverse Event
mcg	Microgram
MedDRA	Medical Dictionary for Regulatory Activities
mg	Milligrams
min	Minute

mITT	Modified Intent-To-Treat
mL	Milliliter
MOP	Manual of Procedures
mRNA	Messenger Ribonucleic Acid
MSD	Meso Scale Discovery
N	Number (typically refers to subjects)
NAAT	Nucleic Acid Amplification Test
NaCl	Sodium Chloride
NDA	New Drug Application
NEUT	Neutralizing
NIAID	National Institute of Allergy and Infectious Diseases
NIH	National Institutes of Health
NOCMC	New Onset Chronic Medical Condition
OHRP	Office for Human Research Protections
PBMC	Peripheral Blood Mononuclear Cell
PCR	Polymerase Chain Reaction
PEG	Polyethylene Glycol
PHI	Protected Health Information
PI	Principal Investigator
PP	Per Protocol
PREP Act	Public Readiness and Emergency Preparedness Act
QA	Quality Assurance
QC	Quality Control

RNA	Ribonucleic Acid
RT-PCR	Reverse Transcription Polymerase Chain Reaction
SAE	Serious Adverse Event
SAP	Statistical Analysis Plan
SARS	Severe Acute Respiratory Syndrome
SARS-CoV	SARS Coronavirus
SARS-CoV-2	SARS Coronavirus 2
SD	Standard Deviation
SDCC	Statistical and Data Coordinating Center
SDSP	Study Data Standardization Plan
SNP	Single Nucleotide Polymorphisms
SOA	Schedule of Activities
SOC	System Organ Class
SOP	Standard Operating Procedure
SST	Serum Separator Tube
SUSAR	Suspected Unexpected Serious Adverse Reaction
Th	T helper
TTS	Thrombosis with Thrombocytopenia Syndrome
UP	Unanticipated Problem
US	United States
USP	United States Pharmacopeia
VOC	Variants Of Concern
VRC	Vaccine Research Center

WHO	World Health Organization
WIV1	Chinese Horseshoe Bat Coronavirus WIV1

10.4 Protocol Amendment History

Protocol Amendment History

Version, Date	Section	Description of Change	Brief Rationale
Version 2.0, 15, April 2022	Throughout	Administrative.	Advanced version and date.
	1.2, 7.1.5, 8.1.6	4 cc will be stored and sent to central lab for potential biomarker testing at Day 4 and Day 60 (new visits added), and at unscheduled visit for suspected case myocarditis or pericarditis	To address FDA comment.
	1.1	Exploratory Objective 5 addition of assessing anti-nucleocapsid serology	To assess for asymptomic SARS-CoV2 infections.
	1.1, 4.1 and 5	Changed "35%" of confirmed infection to "20%" confirmed infection.	To reflect a realistic percentage of participants with confirmed infection enrolling in the study based on US serologic data[51]
	1.1	"A history of myocarditis or pericarditis at any time prior to enrollment" added as an exclusion criteria.	To address FDA comment.

	1.2	Blood volume updated for 2 extra cc of sera collected at any in-person scheduled visit or early termination visit.	As the volume of blood per SST is 8.5 cc rather than 8 cc.
	1.2, 7.1.5, 8.1.6	Added information on management of suspected cases of myocarditis and /or pericarditis.	To address FDA comment.
	1.2	Added clarification that pregnancy testing is conducted at screening as well as prior to each vaccination.	Clarification.
	1.2, 8.2, 8.3.1.1	Added clarifications regarding confirmation of information collected in the Memory Aid.	Clarification.
	2.1 and 2.2.2	Added additional information relevant to the second booster currently part of standard of care.	Provided justification on how the receipt of a variant vaccine in the study is not expected to be inferior to any benefits received from a second booster dose of a Pfizer or Moderna mRNA vaccine obtained outside this trial.

	Throughout	"Women" was changed to "subjects".	To maintain gender neutrality.
	6.5 and 8.1.1	Non COVID-19 approved/authorized vaccinations are only recorded if received 28 days prior to first dose.	Clarification.
	7.1.1	Added "immediate IgE mediated" to halting criteria relevant to allergic reactions related to study vaccine.	Clarification.
	7.1.1.	Added halting criteria relevant to myocarditis and /or pericarditis.	To address FDA comment.
	8.1.1	Description provided for targeted physical exam.	Clarification.
	8.1.5 and 8.3.9	SARS-CoV2 is not an AE but an exploratory efficacy assessment.	To address manufacturer comment.
	8.3.9 and 12	Listing of types of AEs requiring reporting to DMID pharmacovigilance.	To address manufacturer comment.
	11	Changes in references.	Updates.
Version 3.0, 29, April 2022	Throughout	Administrative.	Advanced version and date.

	1.1, 2.1	Changes to number of cases and deaths from COVID-19.	Updates.
	1.1	Addition of study arms.	Update to include Pfizer/ BioNTech BNT162b2 vaccine platform.
	2.1	Data on immunogenicity for Pfizer/BioNTech BNT162b2 vaccine against variants of concern and type of variant vaccines available from Pfizer/BioNTech	Addition of information relevant to new arms of Pfizer/BioNTech variant vaccines.
	2.2.1	Information on risks associated with Pfizer/BioNTech BNT162b2 variant vaccine candidates.	Addition of information relevant to new arms of Pfizer/BioNTech variant vaccines.
	2.2.1	Additional systemic adverse effects listed.	Addition of lethargy, decreased appetite, night sweats, diarrhea and malaise.
	4.1	Addition of stage 2.	Update.
	4.3	Addition of language relevant to dosing justification for Pfizer/BioNTech BNT162b2 variant vaccine candidates.	Addition of information relevant to new arms of Pfizer/BioNTech variant vaccines.

	6.1.1, 6.1.2, 6.2.2, 6.2.3	Addition of language relevant to study product description, dosing, administration, appearance, formulation, stability, storage for Pfizer/BioNTech BNT162b2 variant vaccine candidates.	Addition of information relevant to new arms of Pfizer/BioNTech variant vaccines.
Version 4.0, XX, May 2022	Throughout	Administrative.	Advanced version and date.
	1.1	Addition of study arms.	Update to include Sanofi GSK CoV2 preS dTM-AS03 vaccine platform.
	1.1	Update to eligibility criteria	Addition of history of anaphylaxis, urticaria, or other significant adverse reaction requiring medical intervention after receipt of "polysorbate" has been added as an exclusion criteria.
	1.1	Update to eligibility criteria	Exclusion criteria #6 does not apply to stage 3.
	2.1	Data on immunogenicity and efficacy for Sanofi GSK CoV2 preS dTM-AS03 vaccine against variants of concern and type of variant vaccines available	Addition of information relevant to new arms of Sanofi GSK variant vaccines.

	2.2.1	Information on risks associated with Sanofi GSK variant vaccine candidates.	Addition of information relevant to new arms of Sanofi GSK variant vaccines.
	4,3	Addition of language relevant to dosing justification for Sanofi GSK variant vaccine candidates.	Addition of information relevant to new arms of Sanofi GSK variant vaccines
	6.1.1, 6.1.2, 6.2.2, 6.2.3	Addition of language relevant to study product description, dosing, administration, appearance, formulation, stability, storage for Sanofi GSK variant vaccine candidates.	Addition of information relevant to new arms of Sanofi GSK variant vaccines.

11. REFERENCES

1. WHO. World Health Organization, Weekly Operational Update on COVID-19.

2. .JHU. JHU. -19 Dashboard, Center for Systems Science and Engineering (CCSE) at Johns Hopkins University (JHU).

3. Corbett KS, Edwards DK, Leist SR, et al. SARS-CoV-2 mRNA vaccine design enabled by prototype pathogen preparedness. Nature 2020;586:567-71.

4. Bos R, Rutten L, van der Lubbe JEM, et al. Ad26 vector-based COVID-19 vaccine encoding a prefusion-stabilized SARS-CoV-2 Spike immunogen induces potent humoral and cellular immune responses. NPJ Vaccines 2020;5:91.

5. Heath PT, Galiza EP, Baxter DN, et al. Safety and Efficacy of NVX-CoV2373 Covid-19 Vaccine. N Engl J Med 2021;385:1172-83.

6. Dunkle LM, Kotloff KL, Gay CL, et al. Efficacy and Safety of NVX-CoV2373 in Adults in the United States and Mexico. N Engl J Med 2022;386:531-43.

7. Falsey AR, Sobieszczyk ME, Hirsch I, et al. Phase 3 Safety and Efficacy of AZD1222 (ChAdOx1 nCoV-19) Covid-19 Vaccine. N Engl J Med 2021;385:2348-60.

9. Britton A, Jacobs Slifka KM, Edens C, et al. Effectiveness of the Pfizer-BioNTech COVID-19 Vaccine Among Residents of Two Skilled Nursing Facilities Experiencing COVID-19 Outbreaks - Connecticut, December 2020-February 2021. MMWR Morb Mortal Wkly Rep 2021;70:396-401.

10. Chemaitelly H, Tang P, Hasan MR, et al. Waning of BNT162b2 Vaccine Protection against SARS-CoV-2 Infection in Qatar. N Engl J Med 2021;385:e83.

11. Israel A, Merzon E, Schaffer AA, et al. Elapsed time since BNT162b2 vaccine and risk of SARS-CoV-2 infection: test negative design study. BMJ 2021;375:e067873.

12. Goldberg Y, Mandel M, Bar-On YM, et al. Waning Immunity after the BNT162b2 Vaccine in Israel. N Engl J Med 2021;385:e85.

13. Tegally H, Wilkinson E, Giovanetti M, et al. Detection of a SARS-CoV-2 variant of concern in South Africa. Nature 2021;592:438-43.

14. Di Giacomo S, Mercatelli D, Rakhimov A, Giorgi FM. Preliminary report on severe acute respiratory syndrome coronavirus 2 (SARS-CoV-2) Spike mutation T478K. J Med Virol 2021;93:5638-43.

15. Organization WH. Enhancing Readiness for Omicron (B.1.1.529): Technical Brief and Priority Actions for Member States. December 10, 2021.

16. Barda N, Dagan N, Cohen C, et al. Effectiveness of a third dose of the BNT162b2 mRNA COVID-19 vaccine for preventing severe outcomes in Israel: an observational study. Lancet 2021;398:2093-100.

17. Bar-On YM, Goldberg Y, Mandel M, et al. Protection against Covid-19 by BNT162b2 Booster across Age Groups. N Engl J Med 2021;385:2421-30.

18. Patalon T, Gazit S, Pitzer VE, Prunas O, Warren JL, Weinberger DM. Odds of Testing Positive for SARS-CoV-2 Following Receipt of 3 vs 2 Doses of the BNT162b2 mRNA Vaccine. JAMA Intern Med 2022;182:179-84.

19. Atmar RL, Lyke KE, Deming ME, et al. Homologous and Heterologous Covid-19 Booster Vaccinations. N Engl J Med 2022.

20. Miller J. Safety and Immunogenicity of a 50 µg Booster Dose of Moderna COVID-19 Vaccine. Presented at the ACIP Oct 21, 2021. .

21. Pajon R, Doria-Rose NA, Shen X, et al. SARS-CoV-2 Omicron Variant Neutralization after mRNA-1273 Booster Vaccination. N Engl J Med 2022.

22. Choi A, Koch M, Wu K, et al. Safety and immunogenicity of SARS-CoV-2 variant mRNA vaccine boosters in healthy adults: an interim analysis. Nat Med 2021;27:2025-31.

23. Sridhar S, Joaquin A, Bonaparte MI, et al. Safety and immunogenicity of an AS03-adjuvanted SARS-CoV-2 recombinant protein vaccine (CoV2 preS dTM) in healthy adults: interim findings from a phase 2, randomised, dose-finding, multicentre study. Lancet Infect Dis 2022;22:636-48.

24. Sanofi/GSK. Sanofi and GSK to seek regulatory authorization for COVID-19 vaccine. 2022.

25. Regev-Yochay G, Gonen T, Gilboa M, et al. Efficacy of a Fourth Dose of Covid-19 mRNA Vaccine against Omicron. N Engl J Med 2022.

26. Arbel R, Sergienko R, Friger M, Peretz A. Second Booster Vaccine and Covid-19 Mortality in Adults 60 to 100 Years Old. Research Square2022.

27. Gazit S, Saciuk Y, Perez G, Peretz A, Pitzer V, Patalon T. Relative Effectiveness of Four Doses Compared to Three Dose of the BNT162b2 Vaccine in Israel. medRxiv2022.

28. Shen X, Tang H, McDanal C, et al. SARS-CoV-2 variant B.1.1.7 is susceptible to neutralizing antibodies elicited by ancestral Spike vaccines. bioRxiv 2021.

29. Callaway E, Mallapaty S. Novavax offers first evidence that COVID vaccines protect people against variants. Nature 2021;590:17.

30. Wang P, Nair MS, Liu L, et al. Antibody resistance of SARS-CoV-2 variants B.1.351 and B.1.1.7. Nature 2021;593:130-5.

31. Muik A, Lui BG, Wallisch AK, et al. Neutralization of SARS-CoV-2 Omicron by BNT162b2 mRNA vaccine-elicited human sera. Science 2022;375:678-80.

32. Cele S, Jackson L, Khoury DS, et al. Omicron extensively but incompletely escapes Pfizer BNT162b2 neutralization. Nature 2022;602:654-6.

33. Nemet I, Kliker L, Lustig Y, et al. Third BNT162b2 Vaccination Neutralization of SARS-CoV-2 Omicron Infection. N Engl J Med 2022;386:492-4.

34. Pavot V, Berry C, Kishko M, et al. Protein-based SARS-CoV-2 spike vaccine booster increases cross-neutralization against SARS-CoV-2 variants of concern in non-human primates. Nat Commun 2022;13:1699.

35. van der Straten K, Guerra D, van Gils MJ, et al. Mapping the antigenic diversification of SARS-CoV-2. medRxiv 2022:2022.01.03.21268582.

36. COVID-19 Vaccinations in the United States. Centers for Disease Control and Prevention, 2022.

37. Team CC-R, Food, Drug A. Allergic Reactions Including Anaphylaxis After Receipt of the First Dose of Moderna COVID-19 Vaccine - United States, December 21, 2020-January 10, 2021. MMWR Morb Mortal Wkly Rep 2021;70:125-9.

38. McMahon DE, Amerson E, Rosenbach M, et al. Cutaneous reactions reported after Moderna and Pfizer COVID-19 vaccination: A registry-based study of 414 cases. J Am Acad Dermatol 2021;85:46-55.

39. Baden LR, El Sahly HM, Essink B, et al. Efficacy and Safety of the mRNA-1273 SARS-CoV-2 Vaccine. N Engl J Med 2021;384:403-16.

40. Oster ME, Shay DK, Su JR, et al. Myocarditis Cases Reported After mRNA-Based COVID-19 Vaccination in the US From December 2020 to August 2021. JAMA 2022;327:331-40.

41. ACIP Meeting: Evidence to Recommendation Framework. 2022.

42. ACIP Meeting: Update on myocarditis outcomes. 2022.

43. Gilboa M, Mandelboim M, Indenbaum V, et al. Early Immunogenicity and safety of the third dose of BNT162b2 mRNA Covid-19 vaccine among adults older than 60 years; real world experience. J Infect Dis 2021.

44. Buchan SA, Seo CY, Johnson C, et al. Epidemiology of myocarditis and pericarditis following mRNA vaccines in Ontario, Canada: by vaccine product, schedule and interval. medRxiv 2021:2021.12.02.21267156.

45. Hause AM, Baggs J, Marquez P, Myers TR. Safety Monitoring of COVID-19 Vaccine Booster Doses Among Adults — United States, September 22, 2021–February 6, 2022. MMWR Morb Mortal Wkly Rep 2022.

46. Team CC-R, Food, Drug A. Allergic Reactions Including Anaphylaxis After Receipt of the First Dose of Pfizer-BioNTech COVID-19 Vaccine - United States, December 14-23, 2020. MMWR Morb Mortal Wkly Rep 2021;70:46-51.

47. Polack FP, Thomas SJ, Kitchin N, et al. Safety and Efficacy of the BNT162b2 mRNA Covid-19 Vaccine. N Engl J Med 2020;383:2603-15.

48. Agency EM. Assessment report: Pandemrix2016 28 April.

50. Munro APS, Janani L, Cornelius V, et al. Safety and immunogenicity of seven COVID-19 vaccines as a third dose (booster) following two doses of ChAdOx1 nCov-19 or BNT162b2 in

the UK (COV-BOOST): a blinded, multicentre, randomised, controlled, phase 2 trial. Lancet 2021;398:2258-76.

51. Jones JM, Stone M, Sulaeman H, et al. Estimated US Infection- and Vaccine-Induced SARS-CoV-2 Seroprevalence Based on Blood Donations, July 2020-May 2021. JAMA 2021;326:1400-9.

52. Ferreira VM, Schulz-Menger J, Holmvang G, et al. Cardiovascular Magnetic Resonance in Nonischemic Myocardial Inflammation: Expert Recommendations. J Am Coll Cardiol 2018;72:3158-76.

53. Aretz HT. Myocarditis: the Dallas criteria. Hum Pathol 1987;18:619-24.

54. Adler Y, Charron P, Imazio M, et al. 2015 ESC Guidelines for the diagnosis and management of pericardial diseases: The Task Force for the Diagnosis and Management of Pericardial Diseases of the European Society of Cardiology (ESC)Endorsed by: The European Association for Cardio-Thoracic Surgery (EACTS). Eur Heart J 2015;36:2921-64.

12. APPENDIX A: Adverse Events of Special Interest (AESIs) Terms

Investigators should report all events which fall into the following categories as an AESI per the reporting processes specified in the protocol. The following AESIs are medical concepts that may be related to COVID-19 or are of interest in COVID-19 vaccine safety surveillance. Even if the events below occur in the setting of a SARS-CoV-2 infection, the event should still be reported as an AESI if it is one of the medical concepts below. SARS-CoV-2 infection will not be reported using the AESI reporting system. For documentation and medical assessment purposes all AESIs will also be reported on an SAE form and must be submitted immediately (within 24 hours of site awareness) to the DMID Pharmacovigilance Group; however, when applicable, the narrative will indicate that the AESI did not meet SAE criteria.

Medical Concept	Additional Notes
**Anosmia, Ageusia**	• New onset COVID associated or idiopathic events without other etiology excluding congenital etiologies or trauma
**Subacute thyroiditis**	• Including but not limited to events of: atrophic thyroiditis, autoimmune thyroiditis, immune-mediated thyroiditis, silent thyroiditis, thyrotoxicosis and thyroiditis
**Acute pancreatitis**	• Including but not limited to events of: autoimmune pancreatitis, immune-mediated pancreatitis, ischemic pancreatitis, edematous pancreatitis, pancreatitis, acute pancreatitis, hemorrhagic pancreatitis, necrotizing pancreatitis, viral pancreatitis, and subacute pancreatitis<br>• Excluding known etiologic causes of pancreatitis (alcohol, gallstones, trauma, recent invasive procedures)
**Appendicitis**	• Include any event of appendicitis
**Rhabdomyolysis**	• New onset rhabdomyolysis without known etiology such as excessive exercise or trauma
**Acute respiratory distress syndrome (ARDS)**	• Including but not limited to new events of ARDS and respiratory failure
**Coagulation disorders**	• Including but not limited to thromboembolic and bleeding disorders, disseminated intravascular coagulation, pulmonary embolism, deep vein thrombosis

**Acute cardiovascular injury**	• Including but not limited to myocarditis, pericarditis, microangiopathy, coronary artery disease, arrhythmia, stress cardiomyopathy, heart failure, or acute myocardial infarction
**Acute kidney injury**	• Include events with idiopathic or autoimmune etiologies<br>• Exclude events with clear alternate etiology (trauma, infection, tumor, or iatrogenic causes such as medications or radiocontrast etc.)<br>• Include all cases that meet the following criteria:<br>○ Increase in serum creatinine by ≥ 0.3 mg/dl (≥26.5 umol/l) within 48 hours;<br>○ OR Increase in serum creatinine to ≥ 1.5 times baseline, known or presumed to have occurred within prior 7 days<br>○ OR Urine volume ≤0.5 ml/ kg/ hour for 6 hours
**Acute liver injury**	• Include events with idiopathic or autoimmune etiologies<br>• Exclude events with clear alternate etiology (trauma, infection, tumor, etc.)<br>• Include all cases that meet the following criteria<br>○ > 3-fold elevation above the upper normal limit for ALT or AST<br>○ OR • > 2-fold elevation above the upper normal limit for total serum bilirubin or GGT or ALP
**Dermatologic findings**	• Chilblain-like lesions<br>• Single organ cutaneous vasculitis<br>• Erythema multiforme<br>• Bullous rashes<br>• Severe cutaneous adverse reactions including but not limited to: Stevens-Johnson Syndrome (SJS), Toxic Epidermal Necrolysis (TEN), Drug Reaction with Eosinophilia and Systemic Symptoms (DRESS) and fixed drug eruptions
**Multisystem inflammatory disorders**	• Multisystem inflammatory syndrome in adults (MIS-A)<br>• Multisystem inflammatory syndrome in children (MIS-C)<br>• Kawasaki's disease
**Thrombocytopenia and/or Thrombosis with Thrombocytopenia Syndrome (TTS)**	• Platelet counts < 150 x10^9<br>• Thrombotic events: Suspected deep vessel venous or arterial thrombotic events<br>• Including but not limited to TTS (default operative diagnosis if boosted with Ad26.COV2.S), immune thrombocytopenia, platelet production decreased, thrombocytopenia, thrombocytopenic

	purpura, thrombotic thrombocytopenic purpura, or HELLP syndrome
**Acute aseptic arthritis**	• New onset aseptic arthritis without clear alternate etiology (e.g., gout, osteoarthritis, and trauma)
**New onset of or worsening of neurologic disease**	• Including but not limited to:<br>o Guillain-Barre Syndrome<br>o Acute disseminated encephalomyelitis (ADEM)<br>o Peripheral facial nerve palsy (Bell's palsy)<br>o Transverse myelitis<br>o Encephalitis/Encephalomyelitis<br>o Aseptic meningitis<br>o Febrile seizures<br>o Generalized seizures/convulsions<br>o Stroke (Hemorrhagic and non-hemorrhagic)<br>o Narcolepsy
**Anaphylaxis**	• Anaphylaxis is an acute hypersensitivity reaction with multi-organ-system involvement that can present as, or rapidly progress to, a severe life-threatening reaction. It may occur following exposure to allergens from a variety of sources. Anaphylaxis is a clinical syndrome characterized by:<br>o sudden onset AND<br>o rapid progression of signs and symptoms AND<br>o involving two or more organ systems, as follows:<br>o Skin/ mucosal: urticaria (hives), generalized erythema, angioedema, generalized pruritus with skin rash, generalized prickle sensation, red and itchy eyes<br>o Cardiovascular: measured hypotension, clinical diagnosis of uncompensated shock, loss of consciousness or decreased level of consciousness, evidence of reduced peripheral circulation<br>o Respiratory: bilateral wheeze (bronchospasm), difficulty breathing, stridor, upper airway swelling (lip, tongue, throat, uvula, or larynx), respiratory distress, persistent dry cough, hoarse voice, sensation of throat closure, sneezing, rhinorrhea<br>o Gastrointestinal: diarrhea, abdominal pain, nausea, vomiting<br>• Follow reporting procedures in protocol.
**Myocarditis and/or pericarditis**	Symptoms and diagnostic findings include but are not limited to:<br>• Chest pain<br>• Dyspnea<br>• ST or T wave changes on ECG<br>• Elevated cardiac enzymes

	• Abnormal echocardiography or other cardiac imaging.
**Other syndromes**	• Fibromyalgia<br>• Postural Orthostatic Tachycardia Syndrome<br>• Chronic Fatigue Syndrome (Includes Myalgic encephalomyelitis and Post viral fatigue syndrome)<br>• Myasthenia gravis
