# Supplementary material for: Comparison of bivalent and monovalent SARS-CoV-2 variant vaccines: the phase 2 randomized open-label COVAIL trial
Source: Nat Med. 2023 Aug 28;29(9):2334–46. doi: 10.1038/s41591-023-02503-4 (PMC10504073; doi:10.1038/s41591-023-02503-4)
Supplement: Supplementary file 2 — Reporting Summary [file 41591_2023_2503_MOESM2_ESM.pdf]

nature portfolio

Corresponding author(s): Angela Branche, MD
Nadine Rouphael, MD

Last updated by author(s): Jun 28, 2023

Reporting Summary

Nature Portfolio wishes to improve the reproducibility of the work that we publish. This form provides structure for consistency and transparency in reporting. For further information on Nature Portfolio policies, see our Editorial Policies and the Editorial Policy Checklist.

Statistics

For all statistical analyses, confirm that the following items are present in the figure legend, table legend, main text, or Methods section.

n/a	Confirmed	
☐	☒	The exact sample size (*n*) for each experimental group/condition, given as a discrete number and unit of measurement
☐	☒	A statement on whether measurements were taken from distinct samples or whether the same sample was measured repeatedly
☐	☒	The statistical test(s) used AND whether they are one- or two-sided<br>*Only common tests should be described solely by name; describe more complex techniques in the Methods section.*
☐	☒	A description of all covariates tested
☐	☒	A description of any assumptions or corrections, such as tests of normality and adjustment for multiple comparisons
☐	☒	A full description of the statistical parameters including central tendency (e.g. means) or other basic estimates (e.g. regression coefficient) AND variation (e.g. standard deviation) or associated estimates of uncertainty (e.g. confidence intervals)
☒	☐	For null hypothesis testing, the test statistic (e.g. *F*, *t*, *r*) with confidence intervals, effect sizes, degrees of freedom and *P* value noted<br>*Give P values as exact values whenever suitable.*
☒	☐	For Bayesian analysis, information on the choice of priors and Markov chain Monte Carlo settings
☒	☐	For hierarchical and complex designs, identification of the appropriate level for tests and full reporting of outcomes
☐	☒	Estimates of effect sizes (e.g. Cohen's *d*, Pearson's *r*), indicating how they were calculated

Our web collection on statistics for biologists contains articles on many of the points above.

Software and code

Policy information about availability of computer code

Data collection	Data was collected using Emmes' Advantage eClinical platform.
Data analysis	All analyses were done using SAS v9.4 or higher and R v4.2.0 and v4.2.2. Antibody landscapes were constructed using the ablandscape.fit function,6,7 of the ablandscape package (v=1.1.0, R v=4.2.0) with the parameters method = "cone", error.sd = 1, bandwidth = 1, degree = 1, control = list(optimise.cone.slope = TRUE). The code for antibody landscapes and titer line plots is publicly available in a GitHub repository (https://github.com/acorg/branche_et_al2023).

For manuscripts utilizing custom algorithms or software that are central to the research but not yet described in published literature, software must be made available to editors and reviewers. We strongly encourage code deposition in a community repository (e.g. GitHub). See the Nature Portfolio guidelines for submitting code & software for further information.

Data

Policy information about availability of data

All manuscripts must include a data availability statement. This statement should provide the following information, where applicable:

- Accession codes, unique identifiers, or web links for publicly available datasets
- A description of any restrictions on data availability
- For clinical datasets or third party data, please ensure that the statement adheres to our policy

All data is included in the manuscript. The protocol for the study is provided as supplementary materials.

Human research participants

Policy information about studies involving human research participants and Sex and Gender in Research.

Reporting on sex and gender	Sex reporting was based on sex assigned at birth and collected by self-report. No specific analyses were performed stratified by sex assigned at birth due to the small sample size and expected results. Gender was not included in the data collection or analysis.
Population characteristics	Enrollment was stratified to include older adults age greater than or equal to 65 years (~45%). Enrollment was also stratified by self-report of prior history of SARS-CoV-2 infection (~20%).
Recruitment	Participants were recruited from 22 geographically diverse sites in the US with a goal of ensuring at least 25% of the total cohort would be non-white race or Hispanic ethnicity. Sites independently created a recruitment plan which may have included but were not limited to social media strategies, print media ads, radio and television ads, institutional news letters and the use of database registries. These specific methods were not tracked centrally by the sponsor but overseen by the central IRB. There may be inherent biases related to site specific recruitment strategies which might impact which participants were enrolled that could not be accounted for within the scope of the study.
Ethics oversight	The trial was reviewed and initially approved by the Advarra central institutional review board on March 22, 2022 and overseen by an independent Data and Safety Monitoring Board.

Note that full information on the approval of the study protocol must also be provided in the manuscript.

Field-specific reporting

Please select the one below that is the best fit for your research. If you are not sure, read the appropriate sections before making your selection.

☒ Life sciences ☐ Behavioural & social sciences ☐ Ecological, evolutionary & environmental sciences

For a reference copy of the document with all sections, see nature.com/documents/nr-reporting-summary-flat.pdf

Life sciences study design

All studies must disclose on these points even when the disclosure is negative.

Sample size	The primary objective of this study is to evaluate the magnitude and durability of SARS-CoV-2 specific antibody titers in serum samples. This objective is descriptive in nature. As no pre-specified formal hypothesis tests were planned, no specific sample size calculations were performed and the sample size was chosen to provide reasonable precision to the estimation of geometric means. The precision with which the GMT can be estimated from observed data depends on the standard deviation (SD) of the measurements, on the logarithmic scale, and the sample size. A table was developed which estimated two-sided 95% confidence intervals for the GMT for several values of the observed antibody titer assuming up to 10% attrition which provided the basis for confidence in the precision of our estimates.
Data exclusions	Participants with a SARS-CoV-2 infection occurring between vaccination and a pre-specified immunogenicity timepoint were excluded from immunogenicity analysis at that timepoint and thereafter. Subjects with eligibility deviations were also excluded from analyses.
Replication	Sample sizes were chosen to give reasonable precisions to estimates and findings as described in the protocol.<br><br>For the primary endpoint, pseudovirus neutralization assays against different strains were conducted. All dilutions of each sample were tested in duplicate wells. Assays are only repeated if the quality control checks indicate a failure. The assay has been formally validated and meets FDA standards for accuracy, specificity and precision. Multiple parameters of the assay are monitored for quality control at the sample, assay plate and assay run levels, including duplicate well precision, assay plate and assay run positive control values, relative luminescence range, linearity of neutralization curves, and %CV values of cell control and virus control well.
Randomization	Eligible participants were stratified by age (18-64 and ≥ 65 years) and history of confirmed SARS-CoV-2 infection, and randomly assigned across arms within each stage in an equal ratio. Randomization was stratified by previous infection status determined by self-report only, due to the delay in N-Antibody testing. In analyses, previous infection status is determined as either a self-reported infection or testing N-Antibody positive at baseline.
Blinding	No blinding was performed as the immunogenicity endpoints did not require blinding to maintain rigor. Immunogenicity is an independent measurement and not influenced by participant input or study personnel activities which might requiring blinding.

Behavioural & social sciences study design

All studies must disclose on these points even when the disclosure is negative.

Study description	*Briefly describe the study type including whether data are quantitative, qualitative, or mixed-methods (e.g. qualitative cross-sectional, quantitative experimental, mixed-methods case study).*
Research sample	*State the research sample (e.g. Harvard university undergraduates, villagers in rural India) and provide relevant demographic information (e.g. age, sex) and indicate whether the sample is representative. Provide a rationale for the study sample chosen. For studies involving existing datasets, please describe the dataset and source.*
Sampling strategy	*Describe the sampling procedure (e.g. random, snowball, stratified, convenience). Describe the statistical methods that were used to predetermine sample size OR if no sample-size calculation was performed, describe how sample sizes were chosen and provide a rationale for why these sample sizes are sufficient. For qualitative data, please indicate whether data saturation was considered, and what criteria were used to decide that no further sampling was needed.*
Data collection	*Provide details about the data collection procedure, including the instruments or devices used to record the data (e.g. pen and paper, computer, eye tracker, video or audio equipment) whether anyone was present besides the participant(s) and the researcher, and whether the researcher was blind to experimental condition and/or the study hypothesis during data collection.*
Timing	*Indicate the start and stop dates of data collection. If there is a gap between collection periods, state the dates for each sample cohort.*
Data exclusions	*If no data were excluded from the analyses, state so OR if data were excluded, provide the exact number of exclusions and the rationale behind them, indicating whether exclusion criteria were pre-established.*
Non-participation	*State how many participants dropped out/declined participation and the reason(s) given OR provide response rate OR state that no participants dropped out/declined participation.*
Randomization	*If participants were not allocated into experimental groups, state so OR describe how participants were allocated to groups, and if allocation was not random, describe how covariates were controlled.*

Ecological, evolutionary & environmental sciences study design

All studies must disclose on these points even when the disclosure is negative.

Study description	*Briefly describe the study. For quantitative data include treatment factors and interactions, design structure (e.g. factorial, nested, hierarchical), nature and number of experimental units and replicates.*
Research sample	*Describe the research sample (e.g. a group of tagged Passer domesticus, all Stenocereus thurberi within Organ Pipe Cactus National Monument), and provide a rationale for the sample choice. When relevant, describe the organism taxa, source, sex, age range and any manipulations. State what population the sample is meant to represent when applicable. For studies involving existing datasets, describe the data and its source.*
Sampling strategy	*Note the sampling procedure. Describe the statistical methods that were used to predetermine sample size OR if no sample-size calculation was performed, describe how sample sizes were chosen and provide a rationale for why these sample sizes are sufficient.*
Data collection	*Describe the data collection procedure, including who recorded the data and how.*
Timing and spatial scale	*Indicate the start and stop dates of data collection, noting the frequency and periodicity of sampling and providing a rationale for these choices. If there is a gap between collection periods, state the dates for each sample cohort. Specify the spatial scale from which the data are taken*
Data exclusions	*If no data were excluded from the analyses, state so OR if data were excluded, describe the exclusions and the rationale behind them, indicating whether exclusion criteria were pre-established.*
Reproducibility	*Describe the measures taken to verify the reproducibility of experimental findings. For each experiment, note whether any attempts to repeat the experiment failed OR state that all attempts to repeat the experiment were successful.*
Randomization	*Describe how samples/organisms/participants were allocated into groups. If allocation was not random, describe how covariates were controlled. If this is not relevant to your study, explain why.*
Blinding	*Describe the extent of blinding used during data acquisition and analysis. If blinding was not possible, describe why OR explain why blinding was not relevant to your study.*

Did the study involve field work? ☐ Yes ☒ No

Reporting for specific materials, systems and methods

We require information from authors about some types of materials, experimental systems and methods used in many studies. Here, indicate whether each material, system or method listed is relevant to your study. If you are not sure if a list item applies to your research, read the appropriate section before selecting a response.

Materials & experimental systems

n/a	Involved in the study
☒	☐ Antibodies
☒	☐ Eukaryotic cell lines
☒	☐ Palaeontology and archaeology
☒	☐ Animals and other organisms
☐	☒ Clinical data
☒	☐ Dual use research of concern

Methods

n/a	Involved in the study
☒	☐ ChIP-seq
☒	☐ Flow cytometry
☒	☐ MRI-based neuroimaging

Clinical data

Policy information about clinical studies

All manuscripts should comply with the ICMJE guidelines for publication of clinical research and a completed CONSORT checklist must be included with all submissions.

Clinical trial registration: clinicaltrials.gov NCT 05289037

Study protocol: The full study protocol is provided in the supplemental methods.

Data collection: Subjects were recruited at 22 US sites comprised of research clinics associated with academic medical institutions. These sites are listed in the supplementary appendix. All data was collected during scheduled visits. Recruitment occurred from March 2022 to June 2022 and data collection from followup is still ongoing.

Outcomes: The primary outcome of this study was to evaluate the magnitude, breadth and durability of SARS-CoV-2 specific antibody titers in serum samples from participants in each of the prototype/wildtype or variant vaccine arms by estimating 95% confidence intervals (CI) for the geometric mean titer (GMT) at each timepoint when samples are collected. ANCOVA models were used to estimate GMT ratios of variant vaccines compared to Prototype vaccine and included independent variables for vaccination arms, age (18-64 years and ≥ 65 years of age), previous infection history, and baseline titers.

The secondary outcome was to evaluate the safety of candidate SARS-CoV-2 variant vaccines assessed by solicited injection site and systemic adverse events (AEs), which were collected for 7 days after vaccination; unsolicited AEs through Day 29; and serious adverse events (SAEs), new-onset chronic medical conditions (NOCMCs), adverse events of special interest (AESIs), AEs leading to withdrawal, and medically attended adverse events (MAAEs) through the duration of the trial.
